# Machine learning assessment of myocardial ischemia using angiography: Development and retrospective validation

**DOI:** 10.1371/journal.pmed.1002693

**Published:** 2018-11-13

**Authors:** Hyeonyong Hae, Soo-Jin Kang, Won-Jang Kim, So-Yeon Choi, June-Goo Lee, Youngoh Bae, Hyungjoo Cho, Dong Hyun Yang, Joon-Won Kang, Tae-Hwan Lim, Cheol Hyun Lee, Do-Yoon Kang, Pil Hyung Lee, Jung-Min Ahn, Duk-Woo Park, Seung-Whan Lee, Young-Hak Kim, Cheol Whan Lee, Seong-Wook Park, Seung-Jung Park

**Affiliations:** 1 Department of Cardiology, University of Ulsan College of Medicine, Asan Medical Center, Seoul, Korea; 2 Department of Cardiology, CHA Bundang Medical Center, CHA University, Seongnam, Korea; 3 Department of Cardiology, Ajou University, Suwon, Korea; 4 Biomedical Engineering Research Center, Asan Institute for Life Sciences, Seoul, Korea; 5 Department of Radiology, University of Ulsan College of Medicine, Asan Medical Center, Seoul, Korea; Johns Hopkins University, UNITED STATES

## Abstract

**Background:**

Invasive fractional flow reserve (FFR) is a standard tool for identifying ischemia-producing coronary stenosis. However, in clinical practice, over 70% of treatment decisions still rely on visual estimation of angiographic stenosis, which has limited accuracy (about 60%–65%) for the prediction of FFR < 0.80. One of the reasons for the visual–functional mismatch is that myocardial ischemia can be affected by the supplied myocardial size, which is not always evident by coronary angiography. The aims of this study were to develop an angiography-based machine learning (ML) algorithm for predicting the supplied myocardial volume for a stenosis, as measured using coronary computed tomography angiography (CCTA), and then to build an angiography-based classifier for the lesions with an FFR < 0.80 versus ≥ 0.80.

**Methods and findings:**

A retrospective study was conducted using data from 1,132 stable and unstable angina patients with 1,132 intermediate lesions who underwent invasive coronary angiography, FFR, and CCTA at the Asan Medical Center, Seoul, Korea, between 1 May 2012 and 30 November 2015. The mean age was 63 ± 10 years, 76% were men, and 72% of the patients presented with stable angina. Of these, 932 patients (assessed before 31 January 2015) constituted the training set for the algorithm, and 200 patients (assessed after 1 February 2015) served as a test cohort to validate its diagnostic performance. Additionally, external validation with 79 patients from two centers (CHA University, Seongnam, Korea, and Ajou University, Suwon, Korea) was conducted. After automatic contour calibration using the caliber of guiding catheter, quantitative coronary angiography was performed using the edge-detection algorithms (CAAS-5, Pie-Medical). Clinical information was provided by the Asan BiomedicaL Research Environment (ABLE) system. The CCTA-based myocardial segmentation (CAMS)-derived myocardial volume supplied by each vessel (right coronary artery [RCA], left anterior descending [LAD], left circumflex [LCX]) and the myocardial volume subtended to a stenotic segment (CAMS-%*V*_sub_) were measured for labeling. The ML for (1) predicting vessel territories (CAMS-%LAD, CAMS-%LCX, and CAMS-%RCA) and CAMS-%*V*_sub_ and (2) identifying the lesions with an FFR < 0.80 was constructed. Angiography-based ML, employing a light gradient boosting machine (GBM), showed mean absolute errors (MAEs) of 5.42%, 8.57%, and 4.54% for predicting CAMS-%LAD, CAMS-%LCX, and CAMS-%RCA, respectively. The percent myocardial volumes predicted by ML were used to predict the CAMS-%*V*_*sub*_. With 5-fold cross validation, the MAEs between ML-predicted percent myocardial volume subtended to a stenotic segment (ML-%*V*_*sub*_) and CAMS-%*V*_*sub*_ were minimized by the elastic net (6.26% ± 0.55% for LAD, 5.79% ± 0.68% for LCX, and 2.95% ± 0.14% for RCA lesions). Using all attributes (age, sex, involved vessel segment, and angiographic features affecting the myocardial territory and stenosis degree), the ML classifiers (L2 penalized logistic regression, support vector machine, and random forest) predicted an FFR < 0.80 with an accuracy of approximately 80% (area under the curve [AUC] = 0.84–0.87, 95% confidence intervals 0.71–0.94) in the test set, which was greater than that of diameter stenosis (DS) > 53% (66%, AUC = 0.71, 95% confidence intervals 0.65–0.78). The external validation showed 84% accuracy (AUC = 0.89, 95% confidence intervals 0.83–0.95). The retrospective design, single ethnicity, and the lack of clinical outcomes may limit this prediction model’s generalized application.

**Conclusion:**

We found that angiography-based ML is useful to predict subtended myocardial territories and ischemia-producing lesions by mitigating the visual–functional mismatch between angiographic and FFR. Assessment of clinical utility requires further validation in a large, prospective cohort study.

## Abstract 2

Soo-Jin Kang and colleagues present a machine learning–based model for estimating the risk of ischemia resulting from a coronary stenosis. If prospectively validated, the tool may reduce the invasive nature of this diagnosis.

## Author summary

**Why was this study done?:**

- Invasive fractional flow reserve (FFR, defined as the ratio of maximum flow in a diseased artery to the proximal normal maximum flow) has been a standard tool to detect ischemia-producing lesions with FFR < 0.80.
- Although the current guidelines recommend the routine use of FFR for identifying ischemia-producing lesions, the majority of treatment decisions still rely on visual assessment of the degree of angiographic stenosis because of the time and expense associated with FFR-guided decision-making.
- Conventional angiographic parameters cannot predict the presence of ischemia in cases in which this is affected by the size of subtended myocardium.
- Integration and optimization of information about both myocardial territory and stenosis degree are expected to improve the performance of angiographic prediction of low FFR (FFR < 0.80).

**What did the researchers do and find?:**

- A retrospective study was conducted using data from 1,132 angina patients with 1,132 intermediate coronary lesions (932 in the training dataset and 200 in the internal test dataset), who underwent coronary angiography, coronary computed tomography angiography (CCTA), and FFR to evaluate the lesion morphology, subtended myocardial territories, and inducible ischemia, respectively.
- The mean absolute errors between angiography-based machine learning (ML)-derived versus CCTA-derived subtended myocardial volume were 6.26% ± 0.55% for left anterior descending artery, 5.79% ± 0.68% for left circumflex artery, and 2.95% ± 0.14% for right coronary artery lesions.
- Using all clinical and angiographic features, the ML models predicted an FFR < 0.80 with an overall accuracy of approximately 80% in the test set. In the external validation, the overall accuracy for predicting FFR < 0.80 was 84%.

**What do these findings mean?:**

- Angiography-based supervised ML is useful to predict subtended myocardial territories and to identify ischemia-producing lesions by mitigating the visual–functional mismatch between angiography and FFR.
- The data-driven approach may support clinicians in identifying clinically relevant coronary lesions without FFR measurement and in making clinical decisions.
- Performance and clinical utility require further validation in a large, prospective cohort study.

## Introduction

Stratification of cardiovascular risk in patients with stable coronary artery disease is a key to identify high-risk patients who will benefit from percutaneous coronary intervention (PCI). The appropriateness of revascularization has been determined by the presence and extent of myocardial ischemia. A myocardial perfusion imaging study previously suggested that revascularization has a greater survival benefit in patients with a moderate to large degree of ischemic myocardium (≥10% of the total myocardium) [1]. Invasive fractional flow reserve (FFR, defined as the ratio of maximum flow in a diseased artery to the proximal normal maximum flow) has been a standard tool for lesion-specific hemodynamic assessment and treatment decision-making [2–4]. With abundant clinical evidence showing a significant reduction in major adverse cardiac events using FFR-guided PCI (versus angiography-guided PCI), current guidelines recommend FFR measurement when assessing intermediate coronary stenosis. However, in clinical practice, over 70% of treatment decisions still rely on a visual estimation of angiographic stenosis. This may be due to the prolonged procedure time and high short-term costs associated with FFR-guided diagnosis, as well as the need for adenosine-induced hyperemia and the fact that reimbursement systems do not favor this approach [5,6].

Although invasive coronary angiography and intravascular ultrasound (IVUS) are commonly utilized for evaluating coronary anatomy and optimizing PCI, the subjective nature of visual estimation limits the accurate estimation of stenosis severity [7]. In addition, the integration of morphologic and physiologic parameters and the identification of clinically relevant coronary lesions remain challenging [8–10]. In previous studies, the overall diagnostic accuracy of quantitative angiography for predicting FFR < 0.80 was shown to be only 60%–65% [10,11]. One of the reasons for the visual–functional mismatch is that myocardial ischemia is primarily determined by the variable size of the supplied myocardium at risk, as well as by the degree of stenosis [11]. Our previous data suggested that the application of coronary computed tomography angiography (CCTA)-based myocardial segmentation (CAMS)-derived percent myocardial volume subtended to a stenotic segment (CAMS-%*V*_*sub*_) improves the diagnostic accuracy of angiographic indices used to identify ischemia-producing lesions [12,13]. Nonetheless, the necessity of concurrently performing noninvasive CCTA and invasive angiography limited the clinical utility of the mathematical model.

Machine learning (ML) techniques have emerged as highly effective computer algorithms for the identification of patterns in large datasets with a multitude of variables, facilitating the construction of models for data-driven prediction or classification [14–17]. The aims of this study were to develop an angiography-based supervised ML algorithm for predicting the CAMS-%*V*_*sub*_ and to build an angiography-based supervised ML model to classify lesions into those with an FFR < 0.80 and those ≥ 0.80.

## Methods

### Study population

Between 1 May 2012 and 31 January 2015, 5,378 consecutive patients with stable or unstable angina underwent invasive coronary angiography at the Asan Medical Center, Seoul, Korea. Preprocedural FFR and CCTA data for assessing an intermediate coronary lesion (defined as an angiographic stenosis diameter of 30%–80% on visual estimation) were available for 1,143 patients. Among them, 10 patients with tandem lesions, 10 with stented lesions, 17 with in-stent restenosis, 22 with chronic total occlusion, 10 with side branch evaluation, 145 with significant left main coronary artery stenosis, and 5 with scarred myocardium and regional wall motion abnormality on echocardiography were excluded. When FFR was measured in multiple lesions, the lesion with the lowest FFR value was selected. Following exclusions, 932 patients (932 lesions) were used for model training (the training sample). In addition, data from a nonoverlapping population of 200 stable and unstable angina patients (200 lesions) who underwent preprocedural angiography, IVUS, and FFR in a different phase (between 1 February 2015 and 30 November 2015) were used as a test sample to validate the diagnostic performance of the ML models for the prediction of FFR < 0.80 (Table 1). De-identified clinical information, including patient age and sex, was supported by the Asan BiomedicaL Research Environment (ABLE) system. All patients provided written informed consent for the procedures. The protocol of retrospective data analysis (1 January 2017 to approximately 30 November 2017) was approved by the institutional review board of the Asan Medical Center (S1 file), and a waiver for informed consent was granted. This study is reported as per the transparent reporting of a multivariable prediction model for individual prognosis or diagnosis (TRIPOD) guidelines (S1 Checklist).

**Table 1:** Baseline clinical and angiographic characteristics in the training and test samples.

Characteristics	Training sample	Test sample
Patient/lesion number	932/932	200/200
Age, years	63.12 ± 9.81	63.86 ± 9.56
Men	700 (75%)	158 (79%)
Diabetes mellitus	289 (31%)	59 (30%)
Hypertension	592 (64%)	138 (69%)
Current smoker	387 (42%)	86 (43%)
Hyperlipidemia	597 (64%)	134 (67%)
Unstable angina	181 (19%)	30 (15%)
Body mass index, kg/m^2^	24.97 ± 3.21	24.85 ± 3.09
Body surface area, m^2^	1.73 ± 0.18	1.73 ± 0.17
FFR at maximal hyperemia	0.80 ± 0.11	0.80 ± 0.10
Angiographic data		
LAD artery lesion	591 (63%)	127 (64%)
LCX artery lesion	117 (13%)	24 (12%)
RCA lesion	224 (24%)	49 (24%)
DS, %	53.90 ± 11.24	54.90 ± 9.84
MLD, mm	1.49 ± 0.44	1.52 ± 1.19
Lesion length, mm	17.51 ± 9.69	16.14 ± 8.28
Proximal RLD, mm	3.38 ± 0.56	3.34 ± 0.51
Distal RLD, mm	2.94 ± 0.55	2.89 ± 0.51

Data are shown as *n* (%) or mean ± standard deviation; all, *p*-values were >0.05 between training versus test samples.

Abbreviations: DS, diameter stenosis; FFR, fractional flow reserve; LAD, left anterior descending; LCX, left circumflex; MLD, minimal lumen diameter; RCA, right coronary artery; RLD, reference lumen diameter.

The external validation of the ML models was conducted in 79 angina patients (64 patients from CHA University, Seongnam, Korea, and 15 patients from Ajou University, Suwon, Korea) who underwent invasive coronary angiography and FFR to assess an intermediate coronary lesion.

### Computed tomography imaging and CAMS analysis

**Fig 1:**
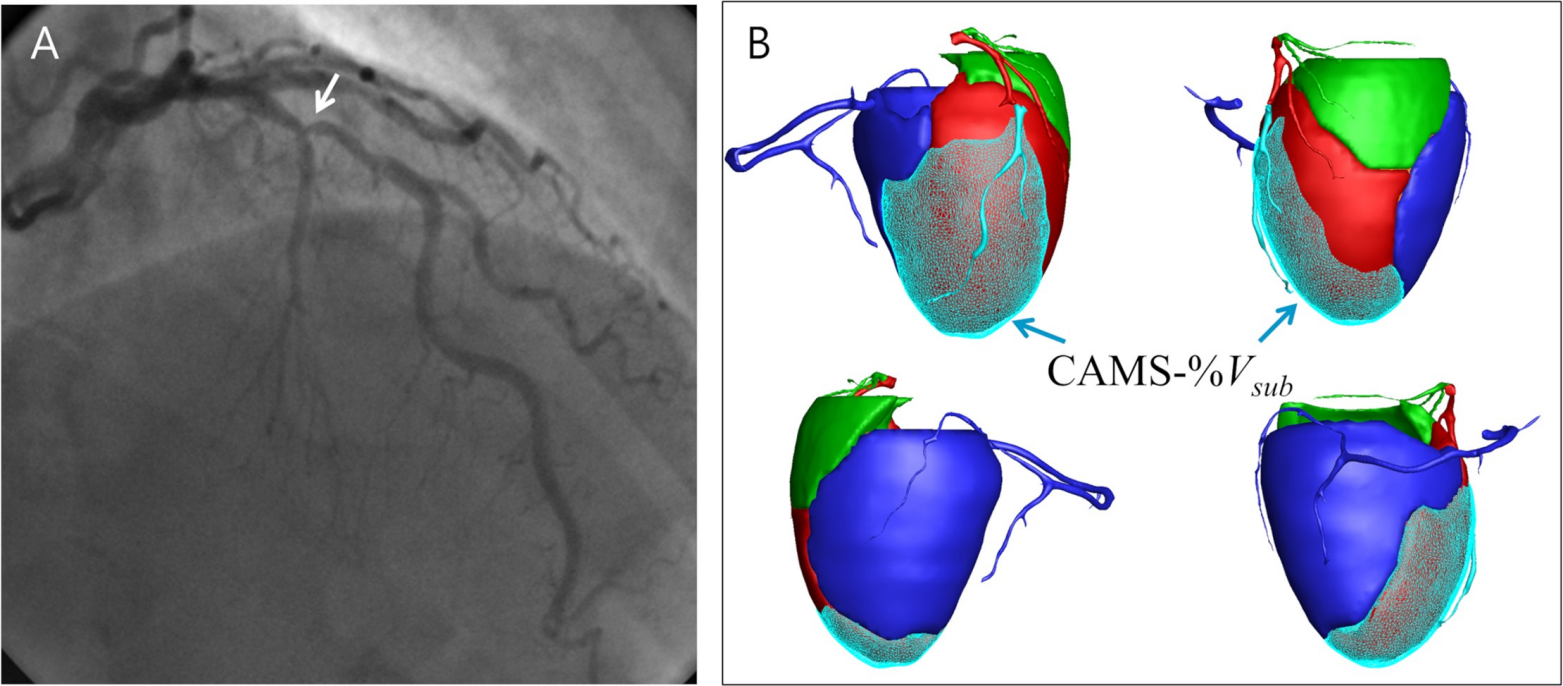
(A) Angiography shows an intermediate stenosis (white arrow) of the mid LAD. (B) The CAMS-derived myocardial volume supplied by LAD was 38.5 cc (shown as red area), and the total left ventricular myocardial volume was 110 cc. The CAMS-%LAD was 35.0%. The myocardial volume subtended to the poststenotic segment was 29.0 cc (blue arrows), and the CAMS-%*V*_*sub*_ was 26.3%. The FFR was 0.82. CAMS, coronary computed tomography angiography–based myocardial segmentation; CAMS-%*V*_*sub*_, CAMS-derived percent myocardial volume subtended to a stenotic segment; FFR, fractional flow reserve; LAD, left anterior descending.

Computed tomography imaging, including CCTA, was performed using first- or second-generation dual-source computed tomography (Definition or Definition Flash, Siemens, Germany). The CCTA data with the fewest motion artifacts and clearest demarcation of the coronary artery were transferred to customized software for CAMS analysis (A-View Cardiac, Asan Medical Center, Korea). After extracting the centerline of each coronary artery and the left ventricular myocardium on the computed tomographic images, the 3D Voronoi algorithm was used to assign the myocardial territories of the major epicardial coronary arteries, including the left anterior descending artery (LAD), left circumflex artery (LCX), and right coronary artery (RCA). In brief, the Voronoi algorithm is a mathematical algorithm that divides the area or space between predetermined points or lines according to the shortest distances from those points or lines [18–20]. The left ventricular myocardial volume was divided into three major epicardial coronary artery territories based on the shortest distance from the coronary artery. The CAMS-%RCA, CAMS-%LCX, and CAMS-%LAD were defined as the percentage ratios of the myocardial volumes supplied by the RCA, LCX, and LAD to the total left ventricular myocardial volume. CAMS-%*V*_sub_ was defined as the percentage ratio of the myocardial volume subtended to a stenotic coronary segment to the total left ventricular myocardial volume. Fig 1 shows an example of the CAMS analysis.

### Angiographic measurements

Quantitative coronary angiography was performed using standard techniques with automated edge-detection algorithms (CAAS-5, Pie-Medical, the Netherlands). After automatic contour calibration by using the known caliber of guiding catheter, angiographic diameter stenosis (DS), minimal lumen diameter (MLD), lesion length, and the proximal and distal reference lumen diameters (RLDs) were measured. Definitions of the angiographic features used for ML training are summarized in Table 2.

**Table 2:** Angiographic features used in the ML models.

Features related to vessel territories	
D_R_ ^#^, mm	Maximal lumen diameter within the 10-mm segment from OS to proximal RCA
D_L_*, mm	Maximal lumen diameter within the 10-mm segment from OS to proximal LAD
D_X_*, mm	Maximal lumen diameter within the 10-mm segment from OS to proximal LCX
D_LM_*, mm	Maximal lumen diameter within left main coronary artery segment
Diminutive RCA	RCA ending prior to giving off the PDA and PL branch
Apex-LAD	LAD runs along the ventricular apex and curves towards the apico-inferior wall
Presence of RI	presence of ramus intermedius
Calculated %RCA^†^	estimated percent myocardial volume supplied by the RCA
Calculated %LAD^†^	estimated percent myocardial volume supplied by the LAD
Calculated %LCX^†^	estimated percent myocardial volume supplied by the LCX
Features related to myocardial volume subtended to a stenotic segment	
Distance to OS, mm	distance between the OS to the narrowest site
Proximal RLD, mm	proximal RLD
Distal RLD, mm	distal RLD
Averaged RLD, mm	average of proximal and distal RLDs
Proximal segment	disease involvement of proximal segment
Mid segment	disease involvement of mid segment
Distal segment	disease involvement of distal segment
D1^‡^, mm	diameter of the uppermost diagonal branch above the stenosis
D2^‡^, mm	diameter of the lower diagonal branch above the stenosis
S1^‡^, mm	diameter of the largest septal branch above the stenosis
D3^‡^, mm	diameter of the uppermost diagonal branch below the stenosis
D4^‡^, mm	diameter of the lower diagonal branch below the stenosis
S2^‡^, mm	diameter of the largest septal branch below the stenosis
D1 + D2, mm	sum of diagonal branch diameters above the stenosis
D1 + D2 + S1, mm	sum of all branch diameters above the stenosis
D3 + D4, mm	sum of diagonal branch diameters below the stenosis
D3 + D4 + S2, mm	sum of all branch diameters below the stenosis
D3^‡^, mm	diameter of the uppermost diagonal branch below the stenosis
D4^‡^, mm	diameter of the lower diagonal branch below the stenosis
First OM	the lesion located at the first OM
Second OM	the lesion located at the second OM
SB1, mm	diameter of the largest branch above the stenosis
SB2, mm	diameter of the uppermost branch below the stenosis
SB3, mm	diameter of the lower branch below the stenosis
SB2 + SB3, mm	sum of all branch diameters below the stenosis (SB2 and SB3)
Features related to lesion severity	
MLD	minimal lumen diameter
%DS	DS = (averaged RLD–MLD)/ averaged RLD × 100
lesion length	length of stenosis

^#^ measured by using LAO view

*measured by using LAO caudal view

^†^ calculated %RCA = 106.1 × D_R_ / (D_L_ + D_X_ + D_R_)– 9.02; calculated %LCX = 140.9 × D_X_ / (D_L_ + D_X_ + D_R_)– 18.24; calculated %LAD = 100 –calculated %RCA–calculated %LCX

^‡^ Only side branches with lumen diameter > 1.5 mm were included.

Abbreviations: DS, diameter stenosis; LAD, left anterior descending artery; LAO, left anterior oblique; LCX, left circumflex artery; ML, machine learning; OM, obtus marginalis; OS, ostium; PDA, posterior descending artery; PL, posterolateral; RCA, right coronary artery; RI, ramus intermedius; RLD, reference lumen diameter.

### FFR measurement

FFR is defined as the ratio of the mean distal coronary pressure (Pd, measured with the pressure wire) to the mean aortic pressure (Pa, measured simultaneously with the guiding catheter) at maximum hyperemia. First, “Equalizing” was performed with the guidewire sensor positioned at the guiding catheter tip. A 0.014-inch FFR pressure guidewire (Radi, St. Jude Medical, Uppsala, Sweden) was then advanced distal to the stenosis. The FFR was measured at the maximum hyperemia induced by an intravenous infusion of adenosine administered through a central vein at 140 μg/kg/min increasing to 200 μg/kg/min, to enhance detection of hemodynamically relevant stenoses. Hyperemic pressure pullback recordings were performed. A stenosis was considered functionally significant when the FFR was <0.80 [3,4].

### IVUS analysis

After intracoronary administration of 0.2 mg nitroglycerin, IVUS imaging was routinely performed using motorized transducer pullback (0.5 mm/s) and a commercial scanner (Boston Scientific Scimed, Minneapolis, MN, United States) with a rotating 40-MHz transducer within a 3.2-French imaging sheath. For the 630 patients for whom preprocedural IVUS data were available, the IVUS-derived minimum lumen area (IVUS-MLA) within a stenotic segment was obtained using computerized software (EchoPlaque 3.0, Indec Systems, Mountain View, CA, USA).

### ML model for predicting CAMS-*V*_*sub*_

**Fig 2:**
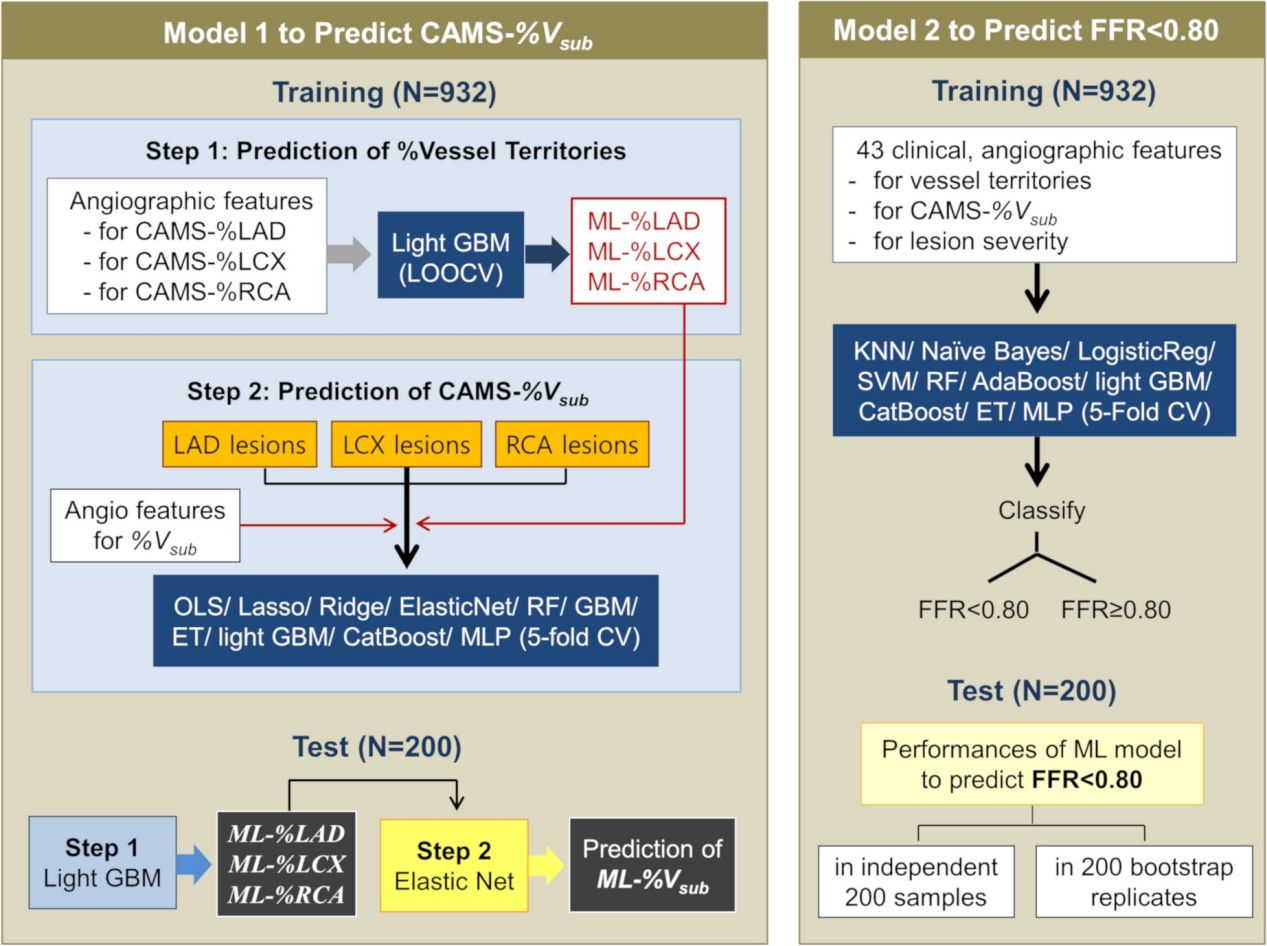
Workflow for the ML. CAMS-%RCA, CAMS-%LCX, and CAMS-%LAD are CCTA-measured percent ratios of the myocardial volumes supplied by the RCA, LCX, and LAD to the total left ventricular myocardial volume. ML-%RCA, ML-%LCX, and ML-%LAD are ML-predicted percent ratios of the myocardial volumes supplied by the RCA, LCX, and LAD to the total left ventricular myocardial volume. CAMS, CCTA-based myocardial segmentation; CAMS-%*V*_*sub*_, CAMS-derived percent myocardial volume subtended to a stenotic segment; CCTA, coronary computer tomography angiography; CV, cross-validation; ET, extra tree; FFR, fractional flow reserve; GBM, gradient boosting machine; KNN, K-nearest neighbor; LAD, left anterior descending; LCX, left circumflex; LOOCV, leave-one-out cross-validation; ML, machine learning; ML-%*V*_*sub*_, ML-predicted percent myocardial volume subtended to a stenotic segment; MLP, multilayer perceptron; OLS, ordinary least squares; RCA, right coronary artery; RF, random forest; SVM, support vector machine.

The overall flow of the supervised ML models using the angiographic features is shown in Fig 2. A theoretical overview and summary of the ML algorithms and technical details are described in the supporting information (S1 Text).

First, a light gradient boosting machine (GBM) with leave-one-out cross-validation was applied to predict the CAMS-derived percent myocardial volume supplied by each coronary artery (CAMS-%RCA, CAMS-%LCX, and CAMS-%LAD). The angiographic attributes affecting each vessel territory are summarized in Table 2. The variables estimated in our pilot data on the basis of the lumen diameters of LAD, RCA, and LCX (calculated %RCA, %LCX, and %LAD) were also included as attributes (see method in S1 Text). Then, the percent myocardial volumes supplied by each coronary artery (ML-%RCA, ML-%LCX, and ML-%LAD), as predicted by the algorithm, were used for the next step.

The second step was to build a model to predict the CAMS-*%V*_*sub*_ values. The ML algorithms evaluated were ordinary least squares (OLSs), ridge and lasso regressions, elastic net, random forests, extra trees, GBM, light GBM, CatBoost, and multilayer perceptrons. A 5-fold cross-validation scheme divided the training sample into five nonoverlapping partitions (method described in S1 Text). Each partition was rotated to be the validation set, with the remaining partitions being used as the training set (S1 Fig). As attributes, the ML-%RCA, ML-%LCX, and ML-%LAD were added to the angiographic features affecting the CAMS-*%V*_*sub*_ (Table 2). The mean absolute errors (MAEs) and mean squared errors (MSEs) were the metrics used to evaluate the performance of the models for predicting the CAMS-*%V*_*sub*_ values.

### ML model for predicting FFR < 0.80

To develop the binary classifiers to separate the lesions with an FFR < 0.80 from those ≥0.80, 43 clinical and angiographic features including age, sex, involved segment (proximal LAD, mid LAD, distal LAD, proximal RCA, mid RCA, distal RCA, proximal LCX, distal LCX, first and second obtus marginalis), and the angiographic features affecting vessel territories, CAMS*-%V*_*sub*_, and lesion severity were used and summarized in S2 Table. The evaluated algorithms were K-nearest neighbor, binary class L2 penalized logistic regression, support vector machine, random forest, extra tree, AdaBoost, light GBM, CatBoost, Gaussian naïve Bayes, and multilayer perceptron (S1 Text). The receiver operating curve (ROC), which was based on the relative performances considering the whole range of possible probability thresholds (from 0 to 1), has an area that ranges from 0.5 for classifiers without any prediction capability to 1 for perfectly classifying algorithms. Analyses based on precision-recall curves were also conducted. Using a 5-fold cross-validation scheme (S1 Fig), the accuracy was calculated by averaging the accuracies over the five tests performed in the multiple rounds of cross-validation. For a nonbiased assessment of the performance for identifying lesions with an FFR of <0.80, the classifiers that had been previously built on the training samples were applied to a completely independent test set of 200 lesions enrolled in the different phase.

In the training set, the algorithms were independently trained on the 200 train-validation random splits with a 3:1 ratio by bootstrap, and the average performances and 95% confidence intervals were calculated. In the 200 bootstrap replicates obtained by random sampling of 50 out of the 200 test samples, the average performance and bootstrap confidence intervals were also calculated.

### Statistical analysis

The statistical analyses for evaluating patient and lesion characteristics at baseline were performed using SPSS (version 10.0, SPSS, Chicago, IL, USA). All values are expressed as means ± 1 standard deviation (continuous variables) or as counts and percentages (categorical variables). Continuous variables were compared using unpaired *t* tests; categorical variables were compared using χ^2^ statistics. A *p*-value < 0.05 was considered statistically significant. ROCs were analyzed using MedCalc Software (Mariakerke, Belgium) to assess the best cutoff for angiographic DS or IVUS-measured lumen area to predict FFR < 0.80 with maximal accuracy.

## Results

### Clinical and lesion characteristics

The clinical characteristics and angiographic data of the patients in the training and test sets are summarized in Table 1. The mean age was 63 ± 10 years, and 76% were men. The evaluated vessels were LAD in 63%. The overall CAMS-%RCA, CAMS-%LAD, and CAMS-%LCX were 27.2% ± 9.3%, 42.4% ± 7.2%, and 27.7% ± 9.6%, respectively. The CAMS-%*V*_*sub*_ was 31.1% ± 10.2%. FFR < 0.80 was shown in 41.6% of the lesions.

### Angiographic prediction of vessel territory

By applying the light GBM to the training sample, the feature importance metrics for determining the CAMS-derived percent myocardial volume subtended to each coronary artery were determined, with the values being summarized in S1 Table. The estimated percent myocardial volumes (calculated %RCA, %LCX, and %LAD) based on the proximal vessel diameters were the most important features for predicting the CAMS-%RCA, CAMS-%LCX, and CAMS-%LAD, respectively. Additionally, the presence of ramus intermedius, a diminutive RCA, and an apical LAD curve affected the vessel territories. With the light GBM and leave-one-out cross-validation, the MAEs and MSEs were 5.42% and 7.10%, respectively, for predicting CAMS-%LAD, 8.57% and 11.28% for predicting CAMS-%LCX, and 4.54% and 6.51% for predicting CAMS-%RCA.

### Angiographic prediction of percent myocardial volume subtended to stenotic segments

Table 3 summarizes the diagnostic performances of the various ML models used to predict CAMS-%*V*_*sub*_. Among the models, the MAEs between the ML-predicted percent myocardial volume subtended to a stenotic segment (ML-%*V*_*sub*_) and the CAMS-%*V*_*sub*_ were minimal with the use of the elastic net algorithm (6.26% ± 0.55% for LAD lesions, 5.79% ± 0.68% for LCX lesions, and 2.95% ± 0.14% for RCA lesions). When the elastic net algorithm was applied to all cases, the overall MAE was 5.39%. Table 4 shows the feature importance metrics by elastic net for the prediction of CAMS-%*V*_*sub*_.

**Table 3:** Diagnostic performances of ML models for predicting the CAMS-%*V*_*sub*_.

Model	OLSs	Lasso	Ridge	Elastic net	Random forest	Extra tree	GBMs	Light GBMs	CatBoost	MLPs
LAD lesion										
MAE, SD	6.26, 0.56	6.26, 0.56	6.27, 0.55	6.26, 0.55	6.54, 0.42	6.65, 0.34	6.43, 0.51	6.63, 0.42	6.41, 0.32	6.29, 0.57
MSE, SD	7.89, 0.72	7.87, 0.72	7.88, 0.72	7.89, 0.69	8.17, 0.50	8.27, 0.41	8.07, 0.64	8.29, 0.50	8.14, 0.39	7.95, 0.69
LCX lesion										
MAE, SD	5.77, 0.67	4.77, 0.70	5.84, 0.73	5.79, 0.68	6.69, 0.55	6.43, 0.59	6.31, 0.75	6.39, 0.36	6.28, 0.43	6.41, 0.62
MSE, SD	7.60, 0.92	7.62, 0.90	7.65, 0.98	7.66, 0.93	8.84, 0.86	8.51, 0.98	8.46, 1.01	8.53, 0.69	8.42, 0.92	8.52, 0.73
RCA lesion										
MAE, SD	3.01, 0.16	2.96, 0.12	2.98, 0.16	2.95, 0.14	3.43, 0.44	3.47, 0.40	3.27, 0.43	3.45, 0.49	3.26, 0.28	4.26, 0.62
MSE, SD	4.23, 0.21	4.19, 0.18	4.20, 0.20	4.19, 0.19	4.62, 0.56	4.63, 0.57	4.51, 0.47	4.87, 0.49	4.41, 0.42	5.80, 0.43

Abbreviations: CAMS-%*V*_*sub*_, coronary computed tomography angiography–based myocardial segmentation–derived percent myocardial volume subtended to a stenotic segment; GBM, gradient boosting machine; LAD, left anterior descending artery; LCX, left circumflex artery; MAE, mean absolute error; ML, machine learning; MLP, multilayer perceptron; MSE, mean squared error; OLS, ordinary least square; RCA, right coronary artery; SD, standard deviation.

**Table 4:** Ranked angiographic features for predicting CAMS-%*V*_*sub*_ and FFR < 0.80.

Rank	Predictors of CAMS-*%V*_*sub*_ by Elastic Net*			Predictors of FFR by CatBoost^#^
	LAD lesion	LCX lesion	RCA lesion	
First	proximal LAD (3.07)	distal RLD (2.77)	ML-%RCA (2.04)	MLD (12.1%)
Second	distal RLD (1.15)	proximal LCX (2.75)	distal RLD (0.13)	%DS (6.1%)
Third	ML-%LAD (1.12)	SB2 + SB3 (2.05)	averaged RLD (0.12)	Age (5.9%)
Fourth	distal LAD (−1.06)	SB2 (0.75)	proximal RLD (0.10)	D_LM_ (5.4%)
Fifth	S1 (−1.04)	SB1 (0.68)	distance to OS (−0.02)	calculated %LCX (4.7%)
Sixth	averaged RLD (0.84)	averaged RLD (0.68)	proximal RCA (0.00)	lesion length (4.5%)
Seventh	D3 + D4 (0.82)	ML-%LCX (0.63)	mid RCA (0.00)	D_RCA_ (4.4%)
Eighth	proximal RLD (0.52)	first OM (−0.59)	distal RCA (0.00)	calculated %LAD (4.3%)
Ninth	D3 (0.44)	SB3 (0.31)		distance to OS (4.3%)
10th	D2 (0.20)	distance to OS (0.00)		D_LCX_ (4.3%)
11th	S2 (0.06)	proximal RLD (0.00)		D_LAD_ (4.1%)
12th	distance to OS (0.05)	distal LCX (0.00)		proximal LAD (4.0%)

*coefficient (by Elastic Net)

^#^feature importance (by CatBoost)

Abbreviations: CAMS-%*V*_*sub*_, coronary computed tomography angiography–based percent myocardial segmentation–derived myocardial volume subtended to a stenotic segment; DS, diameter stenosis; FFR, fractional flow reserve; LAD, left anterior descending artery; LCX, left circumflex artery; MLD, minimal lumen diameter; OM, obtus marginalis; OS, ostium; RCA, right coronary artery; RLD, reference lumen diameter.

### Prediction of FFR < 0.80 in the training sample

**Fig 3:**
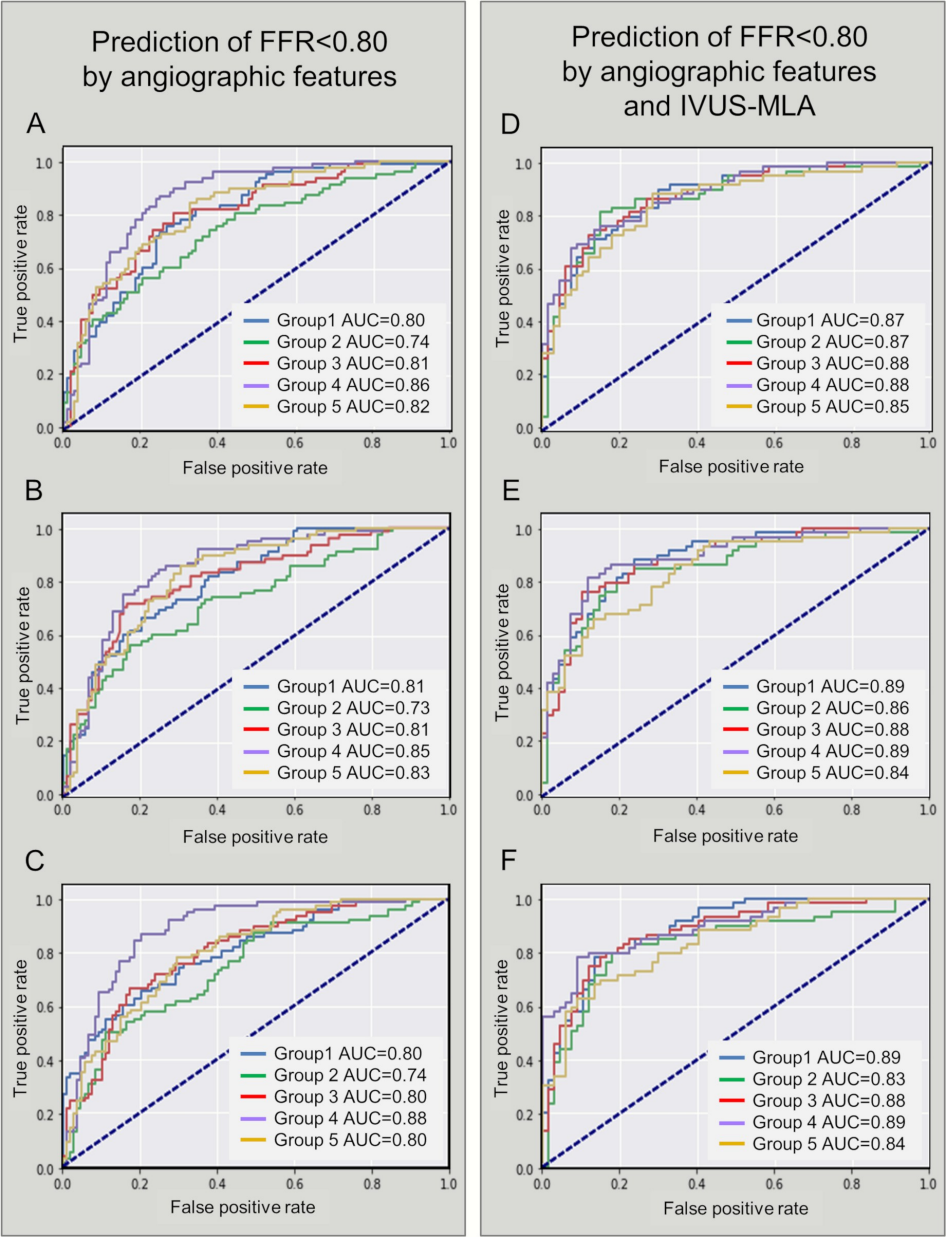
ROCs for predicting FFR < 0.80 in the training sample (*N* = 932). (A) L2 penalized logistic regression using angiographic features. (B) Random forest using angiographic features. (C) CatBoost using angiographic features. (D) L2 penalized logistic regression using angiographic features and IVUS-MLA. (E) Random forest using angiographic features and IVUS-MLA. (F) CatBoost using angiographic features and IVUS-MLA. AUC, area under the curve; FFR, fractional flow reserve; IVUS-MLA, intravascular ultrasound–derived minimal lumen area; ROC, receiver operating curve.

To classify the lesions into those with an FFR < 0.80 versus ≥ 0.80, 43 clinical and angiographic features affecting vessel territories, CAMS*-%V*_*sub*_, and lesion severity were used for ML (S2 Table). Based on the feature importance metrics by CatBoost, the top-12 features for determining the FFR were identified (Table 4). The ROC-based diagnostic performances of the ML algorithms are shown in Table 5, S3 Table, and Fig 3. In addition, the diagnostic performances based on the precision-recall curves are shown in S4 Table.

**Table 5:** Angiographic prediction of FFR < 0.80.

	Threshold of predictive score	AUC	Sensitivity	Specificity	PPV	NPV	Overall accuracy
Prediction of FFR < 0.80 in the training sample (*N* = 932)							
L2 penalized logistic regression*	0.41 ± 0.03 (0.37–0.45)	0.81 ± 0.04 (0.75–0.86)	0.74 ± 0.05 (0.67–0.8)	0.74 ± 0.04 (0.67–0.79)	0.67 ± 0.05 (0.59–0.72)	0.80 ± 0.04 (0.74–0.85)	0.74 ± 0.04 (0.67–0.79)
Support vector machine*	0.42 ± 0.01 (0.40–0.44)	0.81 ± 0.04 (0.74–0.85)	0.73 ± 0.05 (0.65–0.8)	0.74 ± 0.04 (0.68–0.78)	0.67 ± 0.05 (0.59–0.72)	0.79 ± 0.04 (0.73–0.85)	0.74 ± 0.04 (0.67–0.79)
Random forest*	0.43 ± 0.02 (0.40–0.47)	0.81 ± 0.05 (0.75–0.88)	0.72 ± 0.04 (0.65–0.77)	0.72 ± 0.05 (0.66–0.81)	0.65 ± 0.06 (0.57–0.74)	0.78 ± 0.04 (0.72–0.83)	0.72 ± 0.05 (0.65–0.79)
AdaBoost*	0.50 ± 0.00 (0.50–0.50)	0.75 ± 0.05 (0.67–0.82)	0.70 ± 0.03 (0.64–0.74)	0.70 ± 0.04 (0.64–0.76)	0.62 ± 0.04 (0.56–0.68)	0.76 ± 0.03 (0.72–0.8)	0.70 ± 0.04 (0.64–0.75)
CatBoost*	0.37 ± 0.06 (0.27–0.45)	0.78 ± 0.05 (0.73–0.86)	0.71 ± 0.03 (0.67–0.76)	0.72 ± 0.00 (0.65–0.78)	0.64 ± 0.05 (0.58–0.71)	0.77 ± 0.03 (0.73–0.82)	0.71 ± 0.04 (0.66–0.77)
Prediction of FFR < 0.80 in the test sample (*N* = 200)							
L2 penalized logistic regression	0.41	0.86	0.79	0.81	0.78	0.82	0.80
Support vector machine	0.38	0.87	0.80	0.8	0.77	0.83	0.80
Random forest	0.44	0.84	0.78	0.81	0.77	0.81	0.80
AdaBoost	0.50	0.80	0.73	0.76	0.72	0.77	0.74
CatBoost	0.40	0.83	0.75	0.78	0.74	0.79	0.76
External validation cohort (*N* = 79)							
L2 penalized logistic regression	0.33	0.91	0.84	0.8	0.66	0.91	0.81
Support vector machine	0.35	0.89	0.84	0.81	0.68	0.92	0.82
Random forest	0.37	0.89	0.84	0.81	0.68	0.92	0.82
AdaBoost	0.5	0.84	0.76	0.8	0.63	0.88	0.78
CatBoost	0.3	0.89	0.8	0.85	0.71	0.9	0.84

*average of 5-fold cross-validation results shown by mean ± standard deviation.

Abbreviations: AUC, area under the curve; FFR, fractional flow reserve; NPV, negative predictive value; PPV, positive predictive value.

In the subgroup that included the 630 patients with available preprocedural IVUS data, the IVUS-MLA was 2.77 ± 1.32 mm^2^. When the IVUS-MLA was added as an attribute, the classifiers using L2 penalized logistic regression, random forest, and support vector machine showed an overall accuracy of 78%–80% (area under the curve [AUC] = 0.87) to predict an FFR < 0.80 that is used as a hemodynamic index requiring revascularization (Fig 3 and S5 Table).

### Performance in test sample

The test samples including the 200 lesions that were not utilized during the training showed no significant differences in clinical and lesion characteristics in comparison with the training sample (Table 1). In the identification of lesions with an FFR < 0.80, angiographic DS > 53% as the cutoff derived from an ROC analysis showed a sensitivity of 74%, a specificity of 61%, and an overall accuracy of 66% (AUC = 0.71). In addition, an IVUS-MLA < 2.34 mm^2^ had a sensitivity of 53%, a specificity of 79%, and an overall accuracy of 67% (AUC = 0.72).

Using clinical and angiographic features, the overall diagnostic accuracies of the ML classifiers (L2 penalized logistic regression, support vector machine, and random forest) in the test set were approximately 80% for predicting an FFR < 0.80 (AUC = 0.84–0.87, Table 5 and S6 Table). Table 6 summarizes the performances with bootstrap confidence intervals in the 200 bootstrap replicates for each of the training and test sets.

**Table 6:** Angiographic prediction of FFR < 0.80 in 200 bootstrap replicates.

	Threshold of predictive score	AUC	Sensitivity	Specificity	PPV	NPV	Overall accuracy
200 bootstrap replicates in the training set							
L2 penalized logistic regression*	0.42 ± 0.03 [0.37–0.48]	0.81 ± 0.03 [0.76–0.86]	0.74 ± 0.03 [0.69–0.78]	0.74 ± 0.03 [0.68–0.79]	0.67 ± 0.03 [0.61–0.72]	0.80 ± 0.02 [0.76–0.84]	0.74 ± 0.03 [0.69–0.79]
Support vector machine*	0.42 ± 0.02 [0.37–0.46]	0.80 ± 0.02 [0.76–0.85]	0.73 ± 0.03 [0.68–0.78]	0.73 ± 0.03 [0.68–0.79]	0.66 ± 0.03 [0.61–0.73]	0.79 ± 0.02 [0.75–0.84]	0.73 ± 0.03 [0.69–0.79]
Random forest*	0.43 ± 0.02 [0.4–0.47]	0.81 ± 0.02 [0.76–0.85]	0.73 ± 0.03 [0.67–0.8]	0.73 ± 0.03 [0.67–0.78]	0.66 ± 0.03 [0.59–0.72]	0.79 ± 0.02 [0.74–0.84]	0.73 ± 0.03 [0.67–0.79]
AdaBoost*	0.50 ± 0.00 [0.50–0.50]	0.75 ± 0.03 [0.7–0.8]	0.69 ± 0.03 [0.64–0.74]	0.69 ± 0.03 [0.64–0.74]	0.62 ± 0.03 [0.56–0.67]	0.76 ± 0.02 [0.72–0.80]	0.69 ± 0.03 [0.64–0.74]
CatBoost*	0.38 ± 0.06 [0.28–0.49]	0.78 ± 0.03 [0.73–0.83]	0.71 ± 0.03 [0.66–0.76]	0.71 ± 0.03 [0.65–0.76]	0.64 ± 0.03 [0.58–0.69]	0.78 ± 0.02 [0.74–0.82]	0.71 ± 0.03 [0.67–0.76]
200 bootstrap replicates in the test set							
L2 penalized logistic regression*	0.47 ± 0.10 [0.27–0.65]	0.83 ± 0.06 [0.71–0.94]	0.75 ± 0.07 [0.65–0.87]	0.76 ± 0.07 [0.63–0.89]	0.73 ± 0.07 [0.61–0.86]	0.78 ± 0.05 [0.68–0.88]	0.76 ± 0.06 [0.66–0.86]
Support vector machine*	0.47 ± 0.08 [0.34–0.61]	0.85 ± 0.05 [0.76–0.94]	0.78 ± 0.07 [0.65–0.87]	0.78 ± 0.07 [0.67–0.93]	0.76 ± 0.07 [0.63–0.9]	0.81 ± 0.05 [0.70–0.90]	0.78 ± 0.05 [0.68–0.88]
Random forest*	0.46 ± 0.05 [0.38–0.56]	0.81 ± 0.05 [0.71–0.91]	0.74 ± 0.07 [0.61–0.87]	0.74 ± 0.06 [0.63–0.85]	0.71 ± 0.06 [0.6–0.83]	0.77 ± 0.05 [0.67–0.88]	0.74 ± 0.05 [0.64–0.84]
AdaBoost*	0.50 ± 0.01 [0.48–0.52]	0.77 ± 0.06 [0.65–0.88]	0.71 ± 0.08 [0.52–0.87]	0.71 ± 0.07 [0.56–0.81]	0.68 ± 0.07 [0.54–0.8]	0.74 ± 0.06 [0.62–0.87]	0.71 ± 0.06 [0.58–0.82]
CatBoost*	0.46 ± 0.17 [0.16–0.79]	0.80 ± 0.05 [0.69–0.89]	0.73 ± 0.07 [0.57–0.87]	0.74 ± 0.07 [0.59–0.85]	0.70 ± 0.06 [0.58–0.82]	0.76 ± 0.06 [0.66–0.88]	0.73 ± 0.06 [0.62–0.84]

*average of 200 bootstrap replicates shown by mean ± standard deviation

[value] = bootstrap confidence intervals.

Abbreviations: AUC, area under the curve; FFR, fractional flow reserve; NPV, negative predictive value; PPV, positive predictive value.

**Fig 4:**
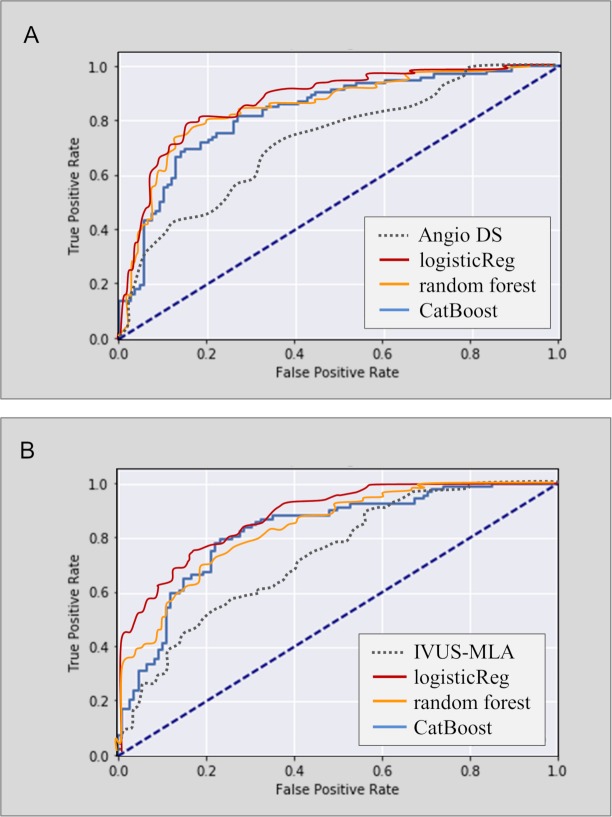
ROC analyses for predicting FFR < 0.80 in the test sample (*N* = 200). (A) The ML models using angiographic features showed greater AUCs (0.83–0.86) than did the angiographic DS alone (AUC = 0.71). (B) The ML models using both angiographic features and IVUS-MLA showed larger AUC (0.82–0.87) than did the IVUS-MLA alone (AUC = 0.72). AUC, area under the curve; DS, diameter stenosis; FFR, fractional flow reserve; IVUS-MLA, intravascular ultrasound–derived minimal lumen area; ML, machine learning; ROC, receiver operating curve.

By adding the IVUS-MLA, the classifiers using L2 penalized logistic regression and support vector machine achieved an overall accuracy of 78%–79% in the test set (AUC = 0.86–0.87, Fig 4 and S5 Table).

### External validation

In the external validation cohort including 79 patients, the age was 59.6 ± 9.0 years, and 58 (73.4%) were men. An FFR < 0.8 was seen in 25 (31.6%) lesions. The angiographic DS and MLD were 48.3% ± 8.0% and 1.64 ± 0.39 mm, respectively. The performances of the ML models for the prediction of FFR < 0.8 were shown in Table 5.

## Discussion

This study demonstrated that (1) angiography-based ML predicted the CAMS-%*V*_*sub*_ with an MAE of 6.26%, 5.79%, and 2.95% for LAD, LCX, and RCA lesions, respectively, and (2) for the identification of ischemia-producing lesions with a FFR < 0.80, the ML classifiers (L2 penalized logistic regression, support vector machine, and random forest) using the angiographic features showed an overall diagnostic accuracy of 80% (maximal AUC = 0.87), which was greater than that of angiographic DS criterion (66%, AUC = 0.71) or even that of the IVUS-MLA threshold (67%, AUC = 0.72).

Assessment of the myocardial mass at risk is of great importance because the presence and extent of ischemic myocardium determines the clinical relevance of revascularization [21–22]. A recent meta-analysis suggested that, in comparison with medical therapy, PCI significantly reduces mortality in patients with objective ischemia documented by functional tests [21]. Moreover, myocardial perfusion imaging suggests that revascularization has a greater survival benefit in patients with a moderate to large degree of ischemic myocardium [1]. These data have provided an insight into a higher-risk population that may benefit from an approach that incorporates ischemia-guided revascularization.

In daily practice, lesion-specific FFR is used to identify ischemia-producing lesions and to decide whether or not to treat it [2–4]. Although coronary angiography and IVUS have been commonly utilized to assess lesion severity, the diagnostic accuracy for predicting an FFR < 0.80 by angiographic DS or IVUS-MLA alone is <60%–70%, which restricts their clinical utility in treatment decision-making [8–10]. Similarly, the current study showed poor diagnostic accuracies for the detection of FFR < 0.80 using angiographic DS > 53% and IVUS-MLA < 2.34 mm^2^ based on the ROC analysis (66% and 67%, respectively). One of the reasons for the visual–functional mismatch is that myocardial ischemia is also determined by the variable size of the supplied myocardium, as well as the degree of stenosis [11].

In our previous study, the use of CAMS-*V*_*sub*_ improved the diagnostic performance of angiographic MLD and/or IVUS-MLA for the prediction of FFR < 0.80 [12,13]. Although a mathematical model using *V*_sub_/MLD^4^ > 6.26 increased the accuracy to 82%, it could be applied only when the patient underwent noninvasive CCTA prior to catheterization. The current angiography-based ML model showed an overall MAE of 5.39% for predicting the CCTA-measured *%V*_*sub*_. During the procedure, the angiographic prediction of the amount of supplied myocardium supports clinicians by confirming the clinical relevance of revascularization treatment in lesions with a large area of myocardium at risk and by precisely identifying the ischemia-producing lesions by reducing the discrepancy between anatomical and functional severity.

Several approaches for an FFR approximation of FFR using angiography-based models have recently been introduced [23–26]. A virtual functional assessment index and quantitative flow ratio based on computational fluid dynamics have shown the overall accuracies of 80%–86% in predicting an FFR < 0.80. Those approaches require a 3D reconstruction of at least two angiographic projections without foreshortening or overlapping vessels and the subsequent computational analyses. Using the clinical and 2D angiographic features affecting the subtended myocardial mass and degree of stenosis, our current ML classifiers predicted an FFR < 0.80 with an overall diagnostic accuracy of 80%. Therefore, the ML models not only reduce procedural expense by avoiding FFR testing but also provide information on the subtended myocardial territory that cannot be predicted by the FFR value. Ultimately, this data-driven approach extends the role of angiography in decision-making for the management of intermediate coronary stenosis.

Although traditional statistical methods validate the association between specific features and an endpoint, the development of a prediction model remains challenging, particularly in the setting of a nonlinear relationship between a factor and an outcome, interactions among variables, and the presence of many predictor variables. ML, an application of artificial intelligence, provides the ability to automatically learn a task without being explicitly programmed [14–17]. The algorithms attempt to balance two competing interests, “bias and variance,” which are summarized by loss functions to optimize a prediction model. Using angiographic features, both regression and decision tree models showed good performance in the prediction of CAMS-%*V*_*sub*_, which led to greater detection of ischemia-producing lesions with reduced FFR.

The current study demonstrated the impact of the individual variables according to metrics (feature importance). For the prediction of CAMS-%*V*_*sub*_, the important features were seen to be proximal segment involvement, RLD, ML-predicted territory of each vessel, the sum of the distal branch diameters, and the distance between the ostium and the narrowest site. Moreover, the key features for predicting FFR < 0.80 were MLD; %DS; age, proximal vessel size of LAD, LCX, and RCA; lesion length; distance between the ostium and MLD site; and the involvement of the proximal LAD, which suggested the importance of the impact of the angiographic determinant for stenosis degree and vascular territory on the FFR value. Although the rank in each algorithm is specific to the ML model, the approach may be hypothesis generating, suggesting which features are valuable for inclusion in future studies.

### Limitations

This study may be subject to selection bias. As the analysis included the single ethnicity and excluded significant left main disease, side branch, and diffuse and tandem lesions, the model cannot be applied generally. Although the developed models were validated in the historical test set and the external validation cohort, the possibility of overfitting cannot be completely excluded. This model did not include the computational fluid dynamics for estimating the anatomical severity. A large prospective trial is required to validate whether the models allow clinicians to dispense with FFR measurement and therefore change the current clinical practice. Finally, prespecified angiographic features were used for ML; an image-based deep learning strategy using big data is worthy of investigation to achieve optimal diagnostic performance for clinical use.

### Conclusion

Angiography-based ML models were useful for the prediction of CAMS-%*V*_*sub*_ and for improving the detection of ischemia-producing lesions. The data-driven approach may support clinicians in the identification of clinically relevant coronary lesions and in treatment decision-making.

## Supporting information

S1 Checklist TRIPOD statement. TRIPOD, transparent reporting of a multivariable prediction model for individual prognosis or diagnosis.
(PDF)

S1 Text Definition of angina, descriptions of pilot study, 5-fold cross-validation, CAMS measurements, and ML algorithms. CAMS, coronary computed tomography angiography–based myocardial segmentation; ML, machine learning.
(DOC)

S1 Table Ranked angiographic features to predict CAMS-derived percent myocardial volume subtended to each coronary artery. CAMS, coronary computed tomography angiography–based myocardial segmentation.
(DOC)

S2 Table Clinical and angiographic features used in ML for predicting FFR < 0.80. FFR, fractional flow reserve; ML, machine learning.
(DOC)

S3 Table Angiographic prediction of FFR < 0.80 in the training sample. FFR, fractional flow reserve.
(DOC)

S4 Table Precision-recall-based performances for predicting FFR < 0.80. FFR, fractional flow reserve.
(DOC)

S5 Table Prediction of FFR < 0.80 using angiographic features and IVUS-MLA. FFR, fractional flow reserve; IVUS-MLA, intravascular ultrasound–derived minimum lumen area.
(DOC)

S6 Table Angiographic prediction of FFR < 0.80 in test sample. FFR, flow fractional reserve.
(DOC)

S1 Fig Five-fold cross-validation. (TIF)

S1 File Brief summary of study protocol approved by IRB. IRB, institutional review board.
(DOCX)

S1 Data Database and codes. (ZIP)

## Abbreviations

ABLE: Asan BiomedicaL Research Environment
AUC: area under the curve
CAMS: CCTA-based myocardial segmentation
CAMS-%*V*_*sub*_: CAMS-derived percent myocardial volume subtended to a stenotic segment CCTA, coronary computed tomography angiography
DS: diameter stenosis
FFR: fractional flow reserve
GBM: gradient boosting machine
IVUS: intravascular ultrasound
IVUS-MLA: IVUS-derived minimum lumen area
KNN: K-nearest neighbor
LAD: left anterior descending
LAO: left anterior oblique
LCX: left circumflex
MAE: mean absolute error
ML: machine learning
MLD: minimal lumen diameter
ML-%*V*_*sub*_: ML-predicted percent myocardial volume subtended to a stenotic segment
MSE: mean squared error
NPV: negative predictive value
OLS: ordinary least square
Pa: aortic pressure
PCI: percutaneous coronary intervention
Pd: distal coronary pressure
PDA: posterior descending artery
PL: posterolateral
PPV: positive predictive value
RCA: right coronary artery
RLD: reference lumen diameter
ROC: receiver operating curve
TRIPOD: transparent reporting of a multivariable prediction model for individual prognosis or diagnosis
