# Supplementary material for: Machine learning assessment of myocardial ischemia using angiography: Development and retrospective validation
Source: PLoS Med. 2018 Nov 13;15(11):e1002693. doi: 10.1371/journal.pmed.1002693 (PMC6233920; doi:10.1371/journal.pmed.1002693)
Supplement: S1 Text — CAMS, coronary computed tomography angiography–based myocardial segmentation; ML, machine learning. (DOC) [file pmed.1002693.s002.doc]

**S1 Text**

**Expanded Methods and Results**

**Definition of stable and unstable angina**

Both training and test sets included patients who underwent coronary angiography for a clinical indication. Patients with stable (CCS class 1 to 4) or unstable angina pectoris (Braunwald class IB, IC, IIB, IIC, IIIB, IIIC) were evaluated.

Acute coronary syndrome was defined as the group of clinical symptoms, electrocardiographic changes, or elevation of cardiac biomarkers that is compatible with acute myocardial ischemia and categories as unstable angina, non–ST-segment elevation myocardial infarction, and ST-segment elevation myocardial infarction (1). Among them, the cases without an elevation of cardiac biomarkers (CK-MB or troponin) were classified as ‘unstable angina’.

**Pilot study.** To determine the angiographic measurements affecting myocardial volume supplied by each epicardial coronary artery, linear regression analyses were previously performed in 302 patients with angiography and coronary computed tomography angiography (CCTA) data (separate cohort). By angiography, D_L_, D_X_ and D_R_ were defined as the maximal lumen diameters within 10-mm segment of ostium–proximal lle anterior descending artery (LAD), ostium–proximal left circumflex artery (LCX) and ostium–proximal right coronary artery (RCA), respectively. There was a significant correlation between D_R_ / (D_L_ + D_X_ + D_R_) and CAMS-measured %myocardial volume supplied by RCA (r=0.708, p<0.001). In addition, a significant correlation between D_X_ / (D_L_ + D_X_ + D_R_) and CAMS-measured %myocardial volume supplied by LCX was found (r=0.678, p<0.001). The angiographic measurements and CAMS-measured %myocardial volume supplied by each vessel were used as the explanatory and the dependent variables, respectively. For predicting the CAMS-measured %myocardial volume supplied by each vessel, by using slope and intercept on each linear regression line, calculated %RCA, calculated %LCX and calculated %LAD were estimated by following, and then used in the current model as attributes.

calculated %RCA= 106.1 x D_R_ / (D_L_ + D_X_ + D_R_) – 9.02

calculated %LCX= 140.9 x D_X_ / (D_L_ + D_X_ + D_R_) – 18.24

calculated %LAD= 100 – calculated %RCA – calculated %LCX

**5-fold cross validation tests**. The 5-fold cross validation scheme divided the training sample into non-overlapped five partitions (Supplemental Figure 1). Each partition was rotated to be the test set and the rests are used as training data. The accuracy was calculated by averaging the accuracies over five tests. To reduce variability, multiple rounds of cross-validation were performed and averaged.

**Machine learning algorithms.**

1. Ordinary Least Squares:

Ordinary Least Squares (OLS) regression is a generalized linear modeling technique that may be used to model a single response variable which has been recorded on at least an interval scale (2). At a very basic level, the relationship between a continuous response variable (Y) and a continuous explanatory variable (X) may be represented using a line of best-fit, where Y is predicted, at least to some extent, by X. If this relationship is linear, it may be appropriately represented mathematically using the straight line equation 'Y = α + βx'. This can be extended to include multiple explanatory variables by simply adding additional variables to the equation. The form of the model is the same as above with a single response variable (Y), but this time Y is predicted by multiple explanatory variables (X_1_ to X_3_).

Y= α+ β_1_ X_1_+ β_2_ X_2_+ β_3_ X_3_

The interpretation of the parameters (α and β) from the above model is basically the same as for the simple regression model above. α indicates the value of Y when all vales of the explanatory variables are zero. Each β parameter indicates the average change in Y that is associated with a unit change in X, whilst controlling for the other explanatory variables in the model. Model-fit can be assessed through comparing deviance measures of nested models.

1. Ridge

To solve usual regression problem, the ordinary least squares (OLS) estimates are obtained by minimizing the residual squared error (3,4). When we have training data are pairs (x_i_,y_i_) for i=1,…,n. The response y is linear in the predictor variables, taking the form μ+x'β. However, if the β_j_s are unconstrained, they might explode and be susceptible to very high variance. In ridge regression, a criterion is added to the residual squared error term to control the variance.


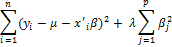


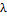
 is a ridge parameter and ranges through [0, ∞]. Adding the penalty reduces the variance of the estimate
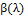
 while introducing a bias. The intercept
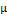
 does not appear in the quadratic penalty term. Defining
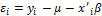
, ridge regression minimizes
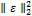
 +
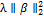
. Ridge regression can also be described via penalization. If we were to minimize
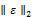
 subject to an upper bound constraint on
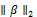
 we would get the same path, though each point on it might correspond to a different
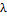
 value.

1. Lasso

Lasso which is 'least absolute shrinkage and selection operator' also has a similar function to ridge in regression problem (3,4). Lasso shrinks some coefficients and sets others to 0, and hence tries to retain the good features for selecting both subsets. The lasso modifies the criterion to


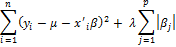


The lasso replaces the L2 penalty
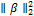
 by an L_1_ penalty
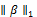
. The main benefit of the lasso is that it can find sparse solutions, ones in which some or even most of the β_j_ are zero. Sparsity is desirable for interpretation.

1. Elastic net

Lasso has some limitations, for example, in the "large p, small n" case (high-dimensional data with few examples). It selects at most n variables before it saturates. Also if there is a group of highly correlated variables, then the Lasso tends to select one variable from a group and ignore the others. To overcome these limitations, the elastic net adds a quadratic part to the penalty (
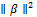
), which when used alone is ridge regression (5). The estimates from the elastic net method are defined by


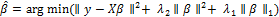
.

The quadratic penalty term makes the loss function strictly convex, and it therefore has a unique minimum.

1. Binary class L2 penalized logistic regression

As an optimization problem, binary class L2 penalized logistic regression minimizes the following cost function:


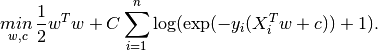


1. Random forest

Random forests add an additional layer of randomness to bagging (6). In addition to constructing each tree using a different bootstrap sample of the data, random forests change how the classification or regression trees are constructed. In standard trees, each node is split using the best split among all variables. In a random forest, each node is split using the best among a subset of predictors randomly chosen at that node. This somewhat counterintuitive strategy turns out to perform very well compared to many other classifiers, including discriminant analysis, support vector machines and neural networks, and is robust against overfitting. The random forests algorithm (for both classification and regression) is as follows:

- Draw
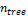
 bootstrap samples from the original data.
- For each of the bootstrap samples, grow an *unpruned* classification or regression tree, with the following modification: at each node, rather than choosing the best split among all predictors, randomly sample
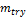
 of the predictors and choose the best split from among those variables. (Bagging can be thought of as the special case of random forests obtained when
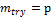
, the number of predictors.)
- Predict new data by aggregating the predictions of the
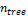
 trees (i.e., majority votes for classification, average for regression).

An estimate of the error rate is obtained, based on the training data, by the following:

- At each bootstrap iteration, predict the data not in the bootstrap sample (what Breiman calls “out-of-bag”, or OOB, data) using the tree grown with the sample.
- Aggregate the OOB predictions. (On the average, each data point would be out-of-bag around 36% of the times, so aggregate these predictions.) Calculate the error rate, and call it the OOB estimate of error rate.

1. Extra-trees

Extremely randomized trees (Extra-trees) algorithm builds an ensemble of unpruned decision or regression trees according to the classical top-down procedure (7). Two main differences from other tree-based ensemble methods are that it splits nodes by choosing cut-points fully at random and that it uses the whole learning sample (rather than a bootstrap replica) to grow the trees. The Extra-Trees splitting procedure for numerical attributes is summarized below. From the bias-variance point of view, the rationale behind the Extra-Trees method is that the explicit randomization of the cut-point and attribute combined with ensemble averaging should be able to reduce variance more strongly than the weaker randomization schemes used by other methods. The usage of the full original learning sample rather than bootstrap replicas is motivated in order to minimize bias. From the computational point of view, the complexity of the tree growing procedure is, assuming balanced trees, on the order of N log N with respect to learning sample size, like other tree growing procedures. The parameters K,
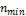
 and M have different effects: K determines the strength of the attribute selection process,
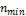
 the strength of averaging output noise, and M the strength of the variance reduction of the ensemble model aggregation. These parameters could be adapted to the problem specifics in a manual or an automatic way (e.g. by cross-validation).

Extra-Tress splitting algorithm (for numerical attributes)


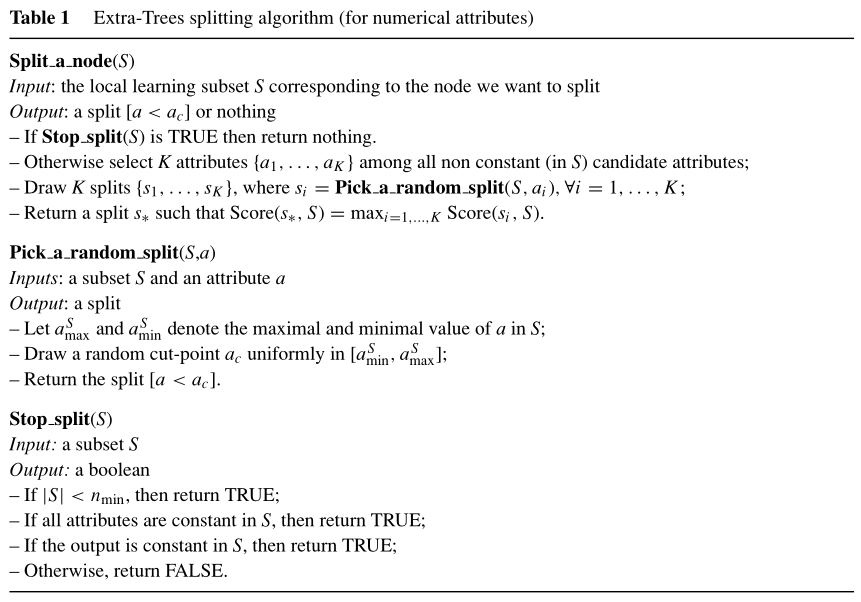


1. Gradient boosting machines

In gradient boosting machines (GBMs), the learning procedure consecutively fits new models to provide a more accurate estimate of the response variable (8,9). The principle idea behind this algorithm is to construct the new base-learners to be maximally correlated with the negative gradient of the loss function, associated with the whole ensemble. The loss functions applied can be arbitrary, but to give a better intuition, if the error function is the classic squared-error loss, the learning procedure would result in consecutive error-fitting. In general, the choice of the loss function is up to the researcher, with both a rich variety of loss functions derived so far and with the possibility of implementing one’s own task-specific loss. This high flexibility makes the GBMs highly customizable to any particular data-driven task. It introduces a lot of freedom into the model design thus making the choice of the most appropriate loss function a matter of trial and error.


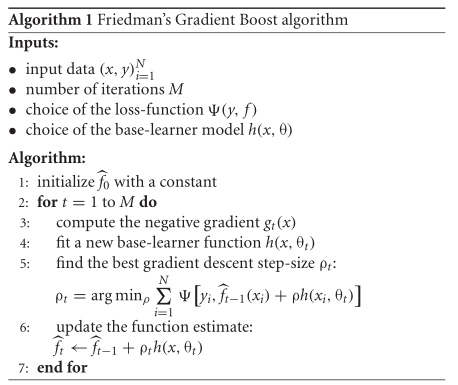


1. Light gradient boosting machines

Light Gradient Boosting Machine (Light GBM) is a fast, distributed, high-performance gradient boosting framework based on decision tree algorithm, used for ranking, classification and many other machine learning tasks (10). Based on decision tree algorithms, it splits the tree leaf wise with the best fit whereas other boosting algorithms split the tree depth wise or level wise rather than leaf-wise.


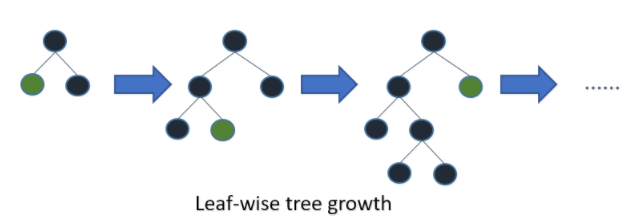


As growing on the same leaf in Light GBM, the leaf-wise algorithm can reduce more loss than the level-wise algorithm and hence results in much better accuracy which can rarely be achieved by any of the existing boosting algorithms. Leaf lead to increase in complexity and may lead to overfitting and it can be overcome by specifying another parameter max-depth which specifies the depth to which splitting will occur.

1. CatBoost

Catboost is a recently open-sourced machine learning method based on gradient boosting over decision trees from Yandex (11,12). The name, “Catboost” comes from two words “Category” and “Boosting”. Catboost is the successor of the MatrixNet algorithm that is widely used within the company for ranking tasks, forecasting and making recommendations. It is universal and can be applied across a wide range of areas and to a variety of problems. Catboost can work with diverse data types to help solve a wide range of problems that businesses face today. To top it up, it provides best-in-class accuracy. It yields state-of-the-art results without extensive data training typically required by other machine learning methods. In addition, Catboost reduces the need for extensive hyper-parameter tuning and lower the chances of overfitting also which leads to more generalized models. Although, CatBoost has multiple parameters to tune and it contains parameters like the number of trees, learning rate, regularization, tree depth, fold size, bagging temperature and others. Lastly, Catboost does not require conversion of data set to any specific format like LightGBM. It converts categorical values into numbers using various statistics on combinations of categorical features and combinations of categorical and numerical features

1. Multi-layer perceptrons

Multi-layer perceptrons (MLP) form one type of neural network (13,14). The MLP consists of a system of simple interconnected neurons, or nodes, which is a model representing a nonlinear mapping between an input vector and an output vector.


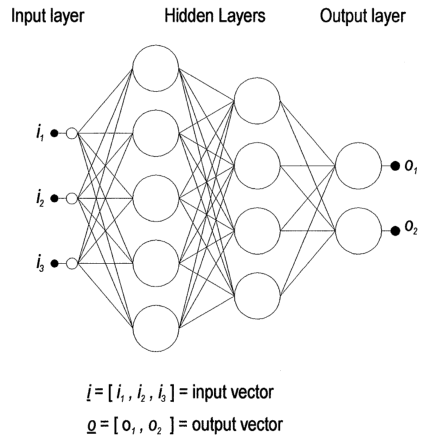


The nodes are connected by weights and output signals which are a function of the sum of the inputs to the node modified by a simple nonlinear transfer, or activation, function. It is the superposition of many simple nonlinear transfer functions that enables the MLP to approximate extremely non-linear functions. If the transfer function was linear then the MLP would only be able to model linear functions. The output of a node is scaled by the connecting weight and fed forward to be an input to the nodes in the next layer of the network. This implies a direction of information processing, hence the MLP is known as a feed-forward neural network.

1. K-nearest-neighbor

K-nearest-neighbor (KNN) classification is one of the most fundamental and simple classification methods and should be one of the first choices for a classification study when there is little or no prior knowledge about the distribution of the data (15,16). The KNN classifier is commonly based on the Euclidean distance between a test sample and the specified training samples. Let
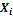
 be an input sample with p features
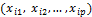
, n be the total number of input samples (i=1,2,…,n) and p the total number of features (j=1,2,…,p). The Euclidean distance between sample
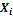
 and
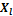
 (l=1,2,…,n) is defined as


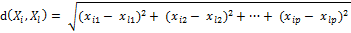


The Voronoi tessellation reflects two characteristics of the example 2-dimensional coordinate system: i) all possible points within a sample's Voronoi cell are the nearest neighboring points for that sample, and ii) for any sample, the nearest sample is determined by the closest Voronoi cell edge. Using the latter characteristic, the KNN classification rule is to assign to a test sample the majority category label of its k nearest training samples. In practice, k is usually chosen to be odd, so as to avoid ties.

1. Support vector machine

Support vector machine (SVM) is a relatively new type of learning algorithm which shows remarkably robust performance with respect to sparse and noisy data (17,18). When used for classification, they separate a given set of binary labeled training data with a hyper-plane that is maximally distant from them (known as ‘the maximal margin hyper-plane’). For cases in which no linear separation is possible, they can work in combination with the technique of ‘kernels’ that automatically realizes a non-linear mapping to a feature space. The hyper-plane found by the SVM in feature space corresponds to a non-linear decision boundary in the input space. The SVM combines 3 ideas: the solution technique from optimal hyperplanes (that allows for an expansion of the solution vector on support vectors), the idea of convolution of the dot-product (that extends the solution surfaces from linear to non-linear), and the notion of soft margins (to allow for errors on the training set).

1. Gaussian Naïve Bayes

Naive Bayes is the simplest form of Bayesian network, in which all attributes are independent given the value of the class variable (19,20). Typically, an example E is represented by a tuple of attribute values (
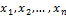
), where
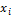
 is the value of attribute
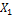
. Let C represent the classification variable, and let c be the value of C. Let’s assume that there are only two classes: + (the positive class) or − (the negative class). A classifier is a function that assigns a class label to an example. From the probability perspective, according to Bayes Rule, the probability of an example E = (
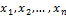
) being class c is


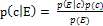


E is classified as the class C = + if and only if


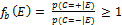
,

where
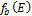
 is called a Bayesian classifier.

Assume that all attributes are independent given the value of the class variable; that is,


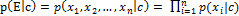
,

The resulting naive Bayesian classifier is then:


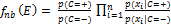
.

1. AdaBoost

The adaptive boosting algorithm, Adaboost is a machine learning meta-algorithm which has been shown to be a very accurate learning procedure (21,22). Adaboost works by generating a set of classifiers and then voting them to classify test examples. The algorithm maintains a probability distribution ω over the training examples. This distribution is initially uniform. The algorithm proceeds in a series of T trials. In each trial, a sample of size m (the size of the training set) is drawn with replacement according to the current probability distribution. This sample is then given to the inner (weak) learning algorithm. The resulting classifier is applied to classify each example in the training set, and the training set probabilities are updated to reduce the probability for correctly-classified examples and increase the probability for misclassified examples. A classifier weight β is computed (for each trial), which is used in the final weighted vote. If a classifier gas an error rate great than 1/2 in a trial, then we reset the training set weights to the uniform distribution and continue drawing samples.

**Coronary CT angiography-based myocardial segmentation.** Despite the emphasis on the impact of the size of the supplied myocardium, the traditional AHA segmentation method with its fixed assignment of coronary territories does not accurately link the culprit vessel with its corresponding myocardial territory.^23-25^ Using coronary computed tomography angiography, a semi-automated, coronary artery-based myocardial segmentation (CAMS) method was recently developed to quantify the myocardium subtended by a specific stenotic coronary segment.^26-28^ The CAMS has recently been developed as a novel, semi-automated approach to quantify the myocardium at risk subtended by individual coronary segments. A validation study using a pig model demonstrated that the CAMS method showed a higher percentage of matched columns than the AHA method (95% vs. 76%). While the AHA method underestimates the ischemic territory of LAD stenosis, the CAMS more precisely identified corresponding coronary territory.^29,30^

Perfusion imaging was performed using second generation dual-source computed coronary tomography (Definition Flash, Siemens, Germany). Data with fewest motion artifacts and clearest demarcation of coronary artery segments were transferred to customized software for CAMS analysis (A-View Cardiac, Asan Medical Center, Korea). After extracting the centerline of each coronary artery and the left ventricular myocardium on the computed tomographic images, a 3-dimensional Voronoi algorithm was used to assign the myocardial territories to the 3 major epicardial coronary arteries with the CAMS program. Briefly, the Voronoi algorithm is a mathematical algorithm that divides the area or space between predetermined points or lines according to the shortest distances from those points or lines.^26-28^ The left ventricular myocardial volume (*V*_total_) was divided into three major epicardial coronary artery territories based on the shortest distance from the coronary artery. The *V*_sub_ was defined as the volume of the myocardium subtended by the stenotic coronary segment.

**References**

1. Anderson L, Adams D, Antman M, et al. ACC/AHA 2007 guidelines for the management of patients with unstable angina/non ST-elevation myocardial infarction: a report of the American College of Cardiology/American Heart Association Task Force on Practice Guidelines (Writing Committee to Revise the 2002 Guidelines for the Management of Patients With Unstable Angina/Non ST-Elevation Myocardial Infarction): developed in collaboration with the American College of Emergency Physicians, the Society for Cardiovascular Angiography and Interventions, and the Society of Thoracic Surgeons: endorsed by the American Association of Cardiovascular and Pulmonary Rehabilitation and the Society for Academic Emergency Medicine. Circulation 2007;116:e148-304.
2. Craven, B. D., and Sardar MN Islam. Ordinary least squares regression. Sage Publications, 2011.
3. Tibshirani, Robert. "Regression shrinkage and selection via the lasso." Journal of the Royal Statistical Society. Series B (Methodological);1996:267-88.
4. Owen, Art B. "A robust hybrid of lasso and ridge regression." Contemporary Mathematics 2007;443: 59-72.
5. Zou, Hui, and Trevor Hastie. "Regularization and variable selection via the elastic net." Journal of the Royal Statistical Society: Series B (Statistical Methodology) 2005;67.2: 301-20.
6. Liaw, Andy, and Matthew Wiener. "Classification and regression by randomForest." R news 2002;2.3: 18-22.
7. Geurts, Pierre, Damien Ernst, and Louis Wehenkel. "Extremely randomized trees." Machine learning 2006;63.1: 3-42.
8. Friedman, Jerome H. "Greedy function approximation: a gradient boosting machine." Annals of statistics 2001: 1189-1232.
9. Natekin, Alexey, and Alois Knoll. "Gradient boosting machines, a tutorial." Frontiers in neurorobotics 7 (2013).
10. Khandelwal, Pranjal. “Which algorithm takes the crown: Light GBM vs XGBOOST?” Analytics Vidhya 2017.
11. “CatBoost is an open-source gradient boosting library with categorical features support”, Yandex, 2017.
12. Ray, Sunil. “CatBoost: A machine learning library to handle categorical (CAT) data automatically”, Analytics Vidhya, 2017.
13. Gardner, Matt W., and S. R. Dorling. "Artificial neural networks (the multilayer perceptron)—a review of applications in the atmospheric sciences." Atmospheric environment 32.14 (1998): 2627-36.
14. Jain, Anil K., Jianchang Mao, and K. Moidin Mohiuddin. "Artificial neural networks: A tutorial." Computer 29.3 (1996): 31-44.
15. Keller, James M., Michael R. Gray, and James A. Givens. "A fuzzy k-nearest neighbor algorithm." IEEE transactions on systems, man, and cybernetics 4 (1985): 580-585.
16. Peterson, Leif E. "K-nearest neighbor." Scholarpedia 4.2 (2009): 1883.
17. Cortes, Corinna, and Vladimir Vapnik. "Support-vector networks." Machine learning 20.3 (1995): 273-297.
18. Furey, Terrence S., et al. "Support vector machine classification and validation of cancer tissue samples using microarray expression data." Bioinformatics 16.10 (2000): 906-914.
19. Zhang, Harry. "The optimality of naive Bayes." AA 1.2 (2004): 3.
20. Lou, Wangchao, et al. "Sequence based prediction of DNA-binding proteins based on hybrid feature selection using random forest and Gaussian naive Bayes." PLoS One 9.1 (2014): e86703.
21. Breiman, Leo. "Bagging predictors." Machine learning 24.2 (1996): 123-140.
22. Margineantu, Dragos D., and Thomas G. Dietterich. "Pruning adaptive boosting." ICML. Vol. 97. 1997
23. Ortiz-Perez JT, Rodriguez J, Meyers SN, Lee DC, Davidson C, Wu E. Correspondence between the 17-segment model and coronary arterial anatomy using contrast-enhanced cardiac magnetic resonance imaging. JACC Cardiovasc Imaging 2008;1:282-293.
24. Donato P, Coelho P, Santos C, Bernardes A, Caseiro-Alves F. Correspondence between left ventricular 17 myocardial segments and coronary anatomy obtained by multi-detector computed tomography: An ex vivo contribution. Surg Radiol Anat 2012;34:805-810.
25. Pereztol-Valdes O, Candell-Riera J, Santana-Boado C, Angel J, Aguade-Bruix S, Castell-Conesa J, Garcia EV, Soler-Soler J. Correspondence between left ventricular 17 myocardial segments and coronary arteries. Eur Heart J 2005;26:2637-2643.
26. Kurata A, Kono A, Sakamoto T, Kido T, Mochizuki T, Higashino H, Abe M, Coenen A, Saru-Chelu RG, de Feyter PJ, Krestin GP, Nieman K. Quantification of the myocardial area at risk using coronary ct angiography and voronoi algorithm-based myocardial segmentation. Eur Radiol 2015;25:49-57.
27. Termeer M, Bescós JO, Breeuwer M, Vilanova A, Gerritsen F, Groller ME, Nagel E. Patient-specific mappings between myocardial and coronary anatomy. Dagstuhl Follow-Ups 2010;1:196-209.
28. Karch R, Neumann F, Neumann M, Szawlowski P, Schreiner W. Voronoi polyhedra analysis of optimized arterial tree models. Ann Biomed Eng 2003;31:548-563.
29. Chung MS, Yang DH, Kim YH, et al. Myocardial segmentation based on coronary anatomy using coronary computed tomography angiography: Development and validation in a pig model. Eur Radiol. 2017:10.1007/s00330-017-4793-0.
30. Kang SJ, Kweon J, Yang DH, et al. Mathematically Derived Criteria for Detecting Functionally Significant Stenoses Using Coronary Computed Tomographic Angiography-Based Myocardial Segmentation and Intravascular Ultrasound-Measured Minimal Lumen Area. Am J Cardiol 2016;118:170-6.
