# Supplementary material for: Machine learning assessment of myocardial ischemia using angiography: Development and retrospective validation
Source: PLoS Med. 2018 Nov 13;15(11):e1002693. doi: 10.1371/journal.pmed.1002693 (PMC6233920; doi:10.1371/journal.pmed.1002693)
Supplement: S1 Table — CAMS, coronary computed tomography angiography–based myocardial segmentation. (DOC) [file pmed.1002693.s003.doc]

**S1 Table. Ranked angiographic features to predict CAMS-derived %myocardial volume subtended to each coronary artery**

| For predicting  CAMS-%LAD* | | For predicting  CAMS-%LCX* | | For predicting  CAMS-%RCA* | |
| --- | --- | --- | --- | --- | --- |
| calculated %LAD | 72 | calculated %LCX | 82 | calculated %RCA | 58 |
| D_LM_ | 47 | D_R_ | 47 | D_LM_ | 51 |
| calculated %RCA | 41 | D_L_ | 33 | calculated %LCX | 41 |
| D_L_ | 30 | D_X_ | 32 | D_R_ | 33 |
| calculated %LCX | 29 | D_LM_ | 28 | D_X_ | 31 |
| D_R_ | 27 | calculated %RCA | 26 | D_L_ | 27 |
| D_X_ | 21 | RI presence | 25 | calculated %LAD | 26 |
| RI presence | 17 | calculated %LAD | 15 | apex curve | 22 |
| apex curve | 16 | diminutive | 7 | diminutive | 7 |
| diminutive | 0 | apex curve | 5 | RI presence | 4 |

Feature importance by using Light GBM

D_R_: Maximal lumen diameter within 10-mm segment from ostium to proximal RCA

D_L_: Maximal lumen diameter within 10-mm segment from ostium to proximal LAD

D_X_ :Maximal lumen diameter within 10-mm segment from ostium to proximal LCX

D_LM_: Maximal lumen diameter within left main coronary artery

CAMS-%RCA, CAMS-%LCX and CAMS-%LAD: % ratios of the myocardial volumes supplied by the RCA, LCX and LAD to the total left ventricular myocardial volume

calculated %RCA= 106.1 x D_R_ / (D_L_ + D_X_ + D_R_) – 9.02

calculated %LCX= 140.9 x D_X_ / (D_L_ + D_X_ + D_R_) – 18.24

calculated %LAD= 100 – calculated %RCA – calculated %LCX
