# Supplementary material for: Machine learning assessment of myocardial ischemia using angiography: Development and retrospective validation
Source: PLoS Med. 2018 Nov 13;15(11):e1002693. doi: 10.1371/journal.pmed.1002693 (PMC6233920; doi:10.1371/journal.pmed.1002693)
Supplement: S2 Table — FFR, fractional flow reserve; ML, machine learning. (DOC) [file pmed.1002693.s004.doc]

**S2 Table. Clinical and angiographic features used in ML for predicting FFR<0.80**

| *Angiographic features related to vessel territories* | |
| --- | --- |
| D_R_ ^#^, mm | Maximal lumen diameter within 10-mm segment from ostium to proximal RCA |
| D_L_*, mm | Maximal lumen diameter within 10-mm segment from ostium to proximal LAD |
| D_X_*, mm | Maximal lumen diameter within 10-mm segment of from ostium to proximal LCX |
| D_LM_*, mm | Maximal lumen diameter within left main coronary artery segment |
| diminutive RCA | RCA ending prior to giving off the PDA and PL branch |
| apex-LAD | LAD runs along the ventricular apex and curved towards the apicoinferior wall |
| presence of RI | presence of ramus intermedius |
| calculated %RCA^†^ | estimated %myocardial volume supplied by RCA |
| calculated %LAD^†^ | estimated %myocardial volume supplied by LAD |
| calculated %LCX^†^ | estimated %myocardial volume supplied by LCX |
| *Angiographic features related to myocardial volume subtended to a stenotic segment* | |
| involved segments  (10 attributes) | Proximal LAD, mid LAD, distal LAD, proximal RCA, mid RCA, distal RCA, proximal LCX, distal LCX, 1^st^ OM, 2^nd^ OM |
| distance to OS, mm | distance between the ostium to the narrowest site |
| proximal RLD, mm | proximal reference lumen diameter |
| distal RLD, mm | distal reference lumen diameter |
| averaged RLD, mm | average of proximal and distal RLDs |
| D1^‡^, mm | diameter of the uppermost diagonal branch above the stenosis |
| D2^‡^, mm | diameter of the lower diagonal branch above the stenosis |
| S1^‡^, mm | diameter of the largest septal branch above the stenosis |
| D3^‡^, mm | diameter of the uppermost diagonal branch below the stenosis |
| D4^‡^, mm | diameter of the lower diagonal branch below the stenosis |
| S2^‡^, mm | diameter of the largest septal branch below the stenosis |
| D1+D2, mm | sum of diagonal branch diameters above the stenosis |
| D1+D2+S1, mm | sum of all branch diameters above the stenosis |
| D3+D4, mm | sum of diagonal branch diameters below the stenosis |
| D3+D4+S2, mm | sum of all branch diameters below the stenosis |
| SB1, mm | diameter of the largest branch above the stenosis |
| SB2, mm | diameter of the uppermost branch below the stenosis |
| SB3, mm | diameter of the lower branch below the stenosis |
| SB2+SB3, mm | sum of all branch diameters below the stenosis (SB2 and SB3) |
| *Angiographic features related to lesion severity* | |
| MLD | minimal lumen diameter |
| %DS | diameter stenosis = (averaged RLD – MLD)/ averaged RLDx100 |
| lesion length | length of stenosis |
| *Clinical features* | |
| age | patient age |
| male | male gender |

^#^ measured by using LAO view, *measured by using LAO caudal view

^†^ calculated %RCA= 106.1 × D_R_ / (D_L_ + D_X_ + D_R_) – 9.02

calculated %LCX= 140.9 × D_X_ / (D_L_ + D_X_ + D_R_) – 18.24

calculated %LAD= 100 – calculated %RCA – calculated %LCX

^‡^ Only sidebranches with lumen diameter >1.5mm were included
