# Supplementary material for: Machine learning assessment of myocardial ischemia using angiography: Development and retrospective validation
Source: PLoS Med. 2018 Nov 13;15(11):e1002693. doi: 10.1371/journal.pmed.1002693 (PMC6233920; doi:10.1371/journal.pmed.1002693)
Supplement: S3 Table — FFR, fractional flow reserve. (DOC) [file pmed.1002693.s005.doc]

**S3 Table. Angiographic prediction of FFR<0.80 in the training sample (N=932)^#^**

|  | threshold of predictive score* | Area under curve* | sensitivity* | specificity* | PPV* | NPV* | Overall accuracy* |
| --- | --- | --- | --- | --- | --- | --- | --- |
| K-nearest neighbor | 0.33 | 0.65 | 84% | 32% | 47% | 74% | 54% |
| L2 penalized logistic regression | 0.41 | 0.81 | 74% | 74% | 67% | 80% | 74% |
| Support vector machine | 0.42 | 0.81 | 73% | 74% | 67% | 79% | 74% |
| Random forest | 0.43 | 0.81 | 72% | 72% | 65% | 78% | 72% |
| Extra Tree | 0.44 | 0.79 | 71% | 73% | 65% | 78% | 72% |
| AdaBoost | 0.50 | 0.75 | 70% | 70% | 62% | 76% | 70% |
| Light GBM | 0.39 | 0.75 | 71% | 72% | 62% | 77% | 70% |
| CatBoost | 0.37 | 0.78 | 71% | 72% | 64% | 77% | 71% |
| Gaussian Naïve Bayes | 0.47 | 0.79 | 72% | 72% | 64% | 78% | 72% |
| Multi-layer perceptron | 0.47 | 0.73 | 67% | 66% | 59% | 74% | 66% |

*average of 5-fold cross-validation results, ^#^use of angiographic features as attributes

GBM= gradient boosting machines, PPV= positive predictive value, NPV= negative predictive value
