# Supplementary material for: Machine learning assessment of myocardial ischemia using angiography: Development and retrospective validation
Source: PLoS Med. 2018 Nov 13;15(11):e1002693. doi: 10.1371/journal.pmed.1002693 (PMC6233920; doi:10.1371/journal.pmed.1002693)
Supplement: S4 Table — FFR, fractional flow reserve. (DOC) [file pmed.1002693.s006.doc]

**S4 Table. Precision-recall based performances for predicting FFR<0.80**

|  | threshold of predictive score | Area under curve | sensitivity | specificity | PPV | NPV | Overall accuracy |
| --- | --- | --- | --- | --- | --- | --- | --- |
| *Prediction of FFR<0.80 in the training sample (N=932)* | | | | | | | |
| L2 penalized logistic regression* | 0.46 (0.02)  [0.42-0.5] | 0.73 (0.02) [0.69-0.78] | 0.69 (0.05) [0.58-0.79] | 0.76 (0.04) [0.69-0.84] | 0.68 (0.05) [0.57-0.78] | 0.77 (0.04) [0.69-0.85] | 0.73 (0.04) [0.64-0.82] |
| Support vector machine* | 0.45 (0.03)  [0.39-0.51] | 0.73 (0.02) [0.68-0.78] | 0.7 (0.05) [0.6-0.8] | 0.77 (0.04) [0.69-0.84] | 0.68 (0.05) [0.58-0.78] | 0.78 (0.04) [0.71-0.86] | 0.74 (0.04) [0.65-0.83] |
| Random forest* | 0.45 (0.02)  [0.41-0.5] | 0.74 (0.05) [0.64-0.83] | 0.69 (0.05) [0.58-0.79] | 0.76 (0.04) [0.68-0.84] | 0.67 (0.05) [0.57-0.77] | 0.77 (0.04) [0.7-0.85] | 0.73 (0.04) [0.64-0.82] |
| AdaBoost* | 0.5 (0.0)  [0.5-0.5] | 0.66 (0.04) [0.58-0.73] | 0.65 (0.06) [0.54-0.76] | 0.74 (0.04) [0.66-0.81] | 0.64 (0.05) [0.53-0.74] | 0.75 (0.04) [0.67-0.83] | 0.7 (0.04) [0.61-0.79] |
| CatBoost* | 0.43 (0.06)  [0.32-0.54] | 0.71 (0.04) [0.63-0.79] | 0.68 (0.04) [0.6-0.76] | 0.76 (0.03) [0.7-0.82] | 0.67 (0.04) [0.59-0.74] | 0.77 (0.03) [0.71-0.83] | 0.73 (0.03) [0.66-0.79] |
| *Prediction of FFR<0.80 in the test sample (N=200)* | | | | | | | |
| L2 penalized logistic regression | 0.41 | 0.82 | 0.79 | 0.81 | 0.78 | 0.82 | 0.8 |
| Support vector machine | 0.39 | 0.82 | 0.78 | 0.81 | 0.78 | 0.81 | 0.8 |
| Random forest | 0.43 | 0.8 | 0.79 | 0.81 | 0.78 | 0.82 | 0.8 |
| AdaBoost | 0.5 | 0.74 | 0.74 | 0.76 | 0.72 | 0.77 | 0.75 |
| CatBoost | 0.38 | 0.79 | 0.75 | 0.77 | 0.73 | 0.78 | 0.76 |
| *200 bootstrap replicates in the training set* | |  |  |  |  |  |  |
| L2 penalized logistic regression^#^ | 0.46 (0.03)  [0.4-0.51] | 0.73 (0.04) [0.65-0.8] | 0.7 (0.03) [0.64-0.76] | 0.77 (0.02) [0.73-0.81] | 0.69 (0.03) [0.63-0.74] | 0.78 (0.02) [0.74-0.82] | 0.74 (0.02) [0.69-0.79] |
| Support vector machine^#^ | 0.45 (0.03)  [0.4-0.51] | 0.72 (0.04) [0.65-0.8] | 0.69 (0.03) [0.63-0.75] | 0.77 (0.02) [0.72-0.81] | 0.68 (0.03) [0.62-0.74] | 0.78 (0.02) [0.73-0.82] | 0.74 (0.03) [0.69-0.79] |
| Random forest^#^ | 0.45 (0.02)  [0.42-0.49] | 0.74 (0.04) [0.67-0.82] | 0.69 (0.03) [0.63-0.74] | 0.77 (0.02) [0.73-0.8] | 0.68 (0.03) [0.62-0.73] | 0.77 (0.02) [0.73-0.82] | 0.73 (0.02) [0.69-0.78] |
| AdaBoost^#^ | 0.5 (0.0)  [0.5-0.5] | 0.66 (0.04) [0.58-0.74] | 0.64 (0.03) [0.57-0.71] | 0.73 (0.03) [0.68-0.79] | 0.63 (0.03) [0.56-0.7] | 0.74 (0.02) [0.69-0.79] | 0.7 (0.03) [0.64-0.75] |
| CatBoost^#^ | 0.43 (0.06)  [0.32-0.55] | 0.71 (0.04) [0.64-0.78] | 0.67 (0.03) [0.61-0.73] | 0.75 (0.02) [0.71-0.79] | 0.66 (0.03) [0.6-0.72] | 0.76 (0.02) [0.72-0.8] | 0.72 (0.02) [0.67-0.77] |
| *200 bootstrap replicates in the test set* | |  |  |  |  |  |  |
| L2 penalized logistic regression^#^ | 0.45 (0.08)  [0.29-0.61] | 0.8 (0.06) [0.67-0.92] | 0.77 (0.06) [0.64-0.9] | 0.75 (0.05) [0.64-0.85] | 0.72 (0.06) [0.61-0.83] | 0.79 (0.06) [0.68-0.9] | 0.76 (0.06) [0.65-0.87] |
| Support vector machine^#^ | 0.46 (0.07)  [0.32-0.59] | 0.83 (0.07) [0.7-0.96] | 0.8 (0.06) [0.68-0.92] | 0.78 (0.05) [0.68-0.88] | 0.76 (0.05) [0.65-0.87] | 0.82 (0.05) [0.72-0.93] | 0.79 (0.05) [0.69-0.89] |
| Random forest^#^ | 0.45 (0.04)  [0.37-0.54] | 0.79 (0.07) [0.65-0.92] | 0.75 (0.06) [0.62-0.87] | 0.72 (0.05) [0.62-0.83] | 0.7 (0.06) [0.58-0.81] | 0.77 (0.05) [0.66-0.88] | 0.73 (0.06) [0.62-0.85] |
| AdaBoost^#^ | 0.5 (0.01)  [0.48-0.52] | 0.71 (0.08) [0.56-0.87] | 0.7 (0.07) [0.57-0.83] | 0.69 (0.06) [0.57-0.81] | 0.66 (0.06) [0.53-0.78] | 0.73 (0.06) [0.61-0.84] | 0.69 (0.06) [0.58-0.81] |
| CatBoost^#^ | 0.42 (0.14)  [0.14-0.69] | 0.76 (0.07) [0.61-0.91] | 0.74 (0.07) [0.61-0.87] | 0.72 (0.06) [0.61-0.84] | 0.7 (0.06) [0.57-0.82] | 0.77 (0.06) [0.65-0.88] | 0.73 (0.06) [0.61-0.85] |

*average of 5-fold cross-validation results, by mean (standard deviation), PPV= positive predictive value, NPV= negative predictive value, [value]=95% confidence intervals

^#^average of 200 bootstrap replicates shown by mean (standard deviation), [value]=bootstrap confidence intervals
