# Supplementary material for: Machine learning assessment of myocardial ischemia using angiography: Development and retrospective validation
Source: PLoS Med. 2018 Nov 13;15(11):e1002693. doi: 10.1371/journal.pmed.1002693 (PMC6233920; doi:10.1371/journal.pmed.1002693)
Supplement: S5 Table — FFR, fractional flow reserve; IVUS-MLA, intravascular ultrasound–derived minimum lumen area. (DOC) [file pmed.1002693.s007.doc]

**S5 Table. Prediction of FFR<0.80 using angiographic features and IVUS-MLA**

|  | threshold of predictive score | Area under curve* | sensitivity | specificity | PPV | NPV | Overall accuracy |
| --- | --- | --- | --- | --- | --- | --- | --- |
| *In the training set (N=630)* |  |  |  |  |  |  |  |
| L2 penalized logistic regression | 0.46 (0.07)  [0.38-0.57] | 0.87 (0.01)  [0.85-0.88] | 0.78 (0.03)  [0.75-0.83] | 0.79 (0.02)  [0.76-0.82] | 0.77 (0.02)  [0.74-0.80] | 0.80 (0.02)  [0.77-0.84] | 0.78 (0.02)  [0.76-0.82] |
| Support vector machine | 0.48 (0.04)  [0.44-0.55] | 0.87 (0.02)  [0.84-0.89] | 0.79 (0.04)  [0.72-0.84] | 0.80 (0.03)  [0.75-0.84] | 0.78 (0.04)  [0.72-0.82] | 0.81 (0.04)  [0.75-0.86] | 0.80 (0.04)  [0.74-0.84] |
| Random forest | 0.47 (0.04)  [0.42-0.52] | 0.87 (0.02)  [0.83-0.89] | 0.79 (0.03)  [0.74-0.81] | 0.79 (0.02)  [0.76-0.82] | 0.77 (0.02)  [0.73-0.80] | 0.81 (0.02)  [0.77-0.83] | 0.79 (0.02)  [0.75-0.81] |
| AdaBoost | 0.50 (0.00)  [0.49-0.50] | 0.84 (0.03)  [0.80-0.88] | 0.78 (0.02)  [0.75-0.79] | 0.74 (0.02)  [0.72-0.76] | 0.73 (0.01)  [0.71-0.74] | 0.79 (0.01)  [0.77-0.81] | 0.76 (0.01)  [0.75-0.77] |
| CatBoost | 0.44 (0.19)  [0.26-0.73] | 0.86 (0.02)  [0.85-0.89] | 0.79 (0.02)  [0.76-0.81] | 0.79 (0.03)  [0.76-0.83] | 0.77 (0.02)  [0.75-0.80] | 0.81 (0.01)  [0.80-0.83] | 0.79 (0.01)  [0.78-0.80] |
| *In the test set (N=200)* |  |  |  |  |  |  |  |
| L2 penalized logistic regression | 0.40 | 0.87 | 0.77 | 0.81 | 0.77 | 0.81 | 0.79 |
| Support vector machine | 0.37 | 0.86 | 0.77 | 0.79 | 0.76 | 0.80 | 0.78 |
| Random forest | 0.41 | 0.83 | 0.74 | 0.75 | 0.72 | 0.77 | 0.74 |
| AdaBoost | 0.49 | 0.83 | 0.73 | 0.74 | 0.70 | 0.77 | 0.74 |
| CatBoost | 0.18 | 0.82 | 0.78 | 0.77 | 0.74 | 0.81 | 0.78 |

*average of 5-fold cross-validation results, PPV= positive predictive value, NPV= negative predictive value

^#^by using angiographic features and IVUS-derived minimal lumen area in 630 patients with available IVUS data
