# Supplementary material for: Machine learning assessment of myocardial ischemia using angiography: Development and retrospective validation
Source: PLoS Med. 2018 Nov 13;15(11):e1002693. doi: 10.1371/journal.pmed.1002693 (PMC6233920; doi:10.1371/journal.pmed.1002693)
Supplement: S6 Table — FFR, flow fractional reserve. (DOC) [file pmed.1002693.s008.doc]

**S4 Table. Angiographic prediction of FFR<0.80 in test sample (N=200)**

|  | threshold of predictive score | Area under curve | sensitivity | specificity | PPV | NPV | Overall accuracy |
| --- | --- | --- | --- | --- | --- | --- | --- |
| ***Using angiographic features*** | |  |  |  |  |  |  |
| K-nearest neighbor | 0.33 | 0.74 | 88% | 51% | 60% | 83% | 68% |
| L2 penalized logistic regression | 0.41 | 0.86 | 79% | 81% | 78% | 82% | 80% |
| Support vector machine | 0.38 | 0.87 | 80% | 80% | 77% | 83% | 80% |
| Random forest | 0.44 | 0.84 | 78% | 81% | 77% | 81% | 80% |
| Extra Tree | 0.43 | 0.84 | 75% | 76% | 73% | 78% | 76% |
| AdaBoost | 0.50 | 0.81 | 73% | 76% | 72% | 77% | 74% |
| Light GBM | 0.25 | 0.79 | 73% | 70% | 68% | 75% | 72% |
| CatBoost | 0.40 | 0.83 | 75% | 78% | 74% | 79% | 76% |
| Gaussian Naïve Bayes | 0.39 | 0.83 | 75% | 73% | 70% | 77% | 74% |
| Multi-layer perceptron | 0.38 | 0.81 | 73% | 70% | 67% | 75% | 72% |

PPV= positive predictive value, NPV= negative predictive value
