# Supplementary figures and images for: Machine learning assessment of myocardial ischemia using angiography: Development and retrospective validation
Source: PLoS Med. 2018 Nov 13;15(11):e1002693. doi: 10.1371/journal.pmed.1002693 (PMC6233920; doi:10.1371/journal.pmed.1002693)

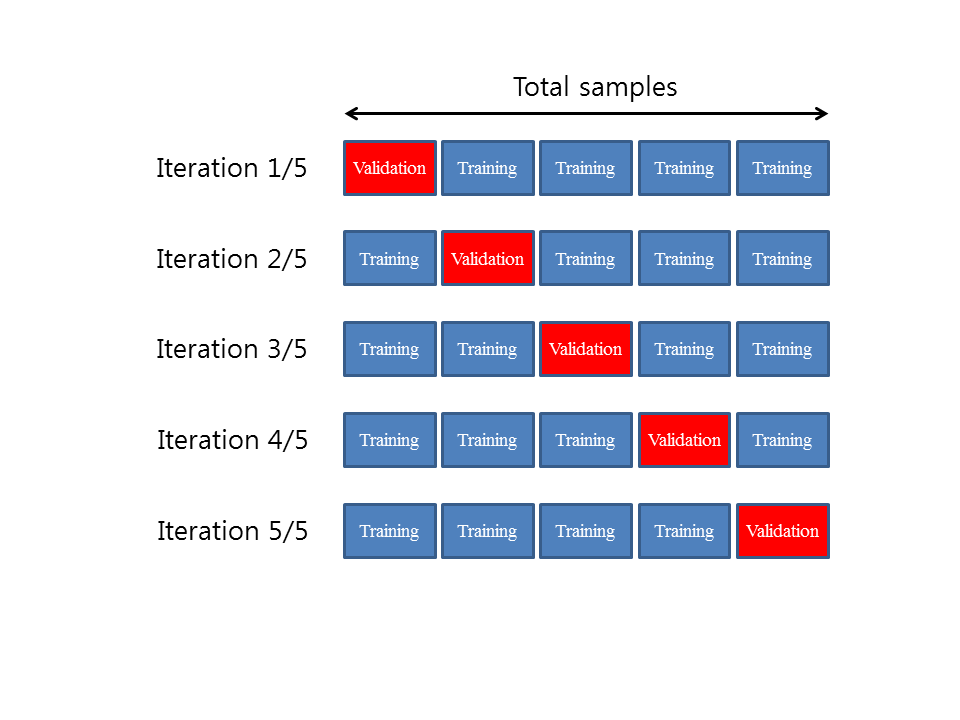

Supplement: S1 Fig — (TIF) [file pmed.1002693.s009.tif]
