# Supplementary material for: Machine learning assessment of myocardial ischemia using angiography: Development and retrospective validation
Source: PLoS Med. 2018 Nov 13;15(11):e1002693. doi: 10.1371/journal.pmed.1002693 (PMC6233920; doi:10.1371/journal.pmed.1002693)
Supplement: S1 File — IRB, institutional review board. (DOCX) [file pmed.1002693.s010.docx]

**Development of prediction models of cardiovascular risk by**

**coronary imaging-based machine learning.**

Principal Investigator: Soo-Jin Kang, M.D. Ph.D

Associate Professor of Medicine

Asan Medical Center

88, Olympic-ro 43-Gil, Songpa-gu, Seoul, Korea 138-736

Phone: 82-2-3010-3157

Fax: 82-2-3010-8634

E-mail: sjkang[@amc.seoul.kr](mailto:sjpark@amc.seoul.kr)

**1. Background**

Stratification of cardiovascular risk in patient with stable coronary artery disease is a key to identify high-risk patients who will benefit from percutaneous coronary intervention. Although previous studies suggested the cardiovascular risk predictors such as the presence of inducible ischemia with an FFR<0.80, the extent of ischemic myocardium, morphological characteristics (small lumen area, large plaque burden, thin-cap fibroatheroma), either diagnosing algorithm of vulnerable plaque or prediction model for cardiovascular events has not yet been provided. With a visual-functional mismatch, there are many debates to determine when and how to integrate the diagnostic modalities for morphological and physiological information, which leads to increasing the expenses for decision making. Moreover, it is challenging to perform real-time interpretation during procedure because it requires accurate lumen segmentation that is a time-consuming and labor-intensive process. Recently developed artificial-intelligence platform showed a high degree of concordance with clinical recommendation, while its application to the patients with coronary artery disease is still limited by the presence of a complex hierarchy of imaging-based decision making and the lack of risk scoring system per vessel or per patient.

**2. Study Objectives**

1) To develop angiography and/or intravascular ultrasound-based machine learning algorithms for predicting the presence of ischemia, subtended myocardial territory, and vulnerable plaques.

2) To develop the data-deriven prediction model for risk scoring of future cardiovascular events, 3) To validate the performances of the prediction models in ABLE-derived retrospective cohort.

**3. Enrollment**

ABLE-derived 10000 patients who underwent coronary angiography and/or pre-procedural intravascular ultrasound will be included.

Exclusion criteria: patients with acute myocardial infarction, history of coronary artery bypass surgery, COPD, the lesions with TIMI<3, left ventricular ejection fraction<40%, variant angina, life expectancy shorter than 2 years.

**3. Study Design**

**3.1 Development of prediction models**

- Angiography/intravascular ultrasound-based machine learning for classifying the lesions with FFR<0.80 vs. ≥0.80 – using logistric regression, eXtream Gradient Boosting, random forest, extra tree, KNN, multi-layer perceptron, artificial neural network, etc.
- Intravascular ultrasound-based deep learning for predicting thin-cap fibroatheroma –suing convolutional neural network.
- Angiography-based algorithm to predict the subtended myocardial volume obtained by CT angiography-based myocardial segmentation.
- Development of risk scoring system to stratify 1-year cardiovascular risk per patient and per lesion using 1)-3)

**3.2 Development of machine leaning algorithm**

**IVUS/NIRS Training**

Feature extraction

ML Parameters

**IVUS/NIRS Testing**

Feature extraction

FA

Non-FA

*Training*

**Offline**

**Classifier**

**Online**

**Classifier**

Classification result

Feature selection

Feature selection

1) Co-registration of 2-mm long histological sections with every 120th IVUS and NIRS sections

2) Each IVUS image is labeled by histologically–defined FA vs. non-FA (TCFA vs. non-TCFA)


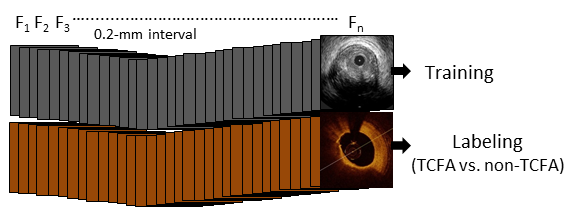

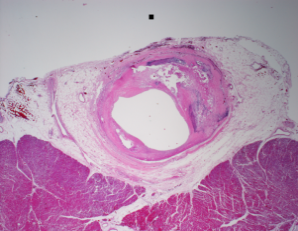


Labeled by

FA vs. non-FA

IVUS data


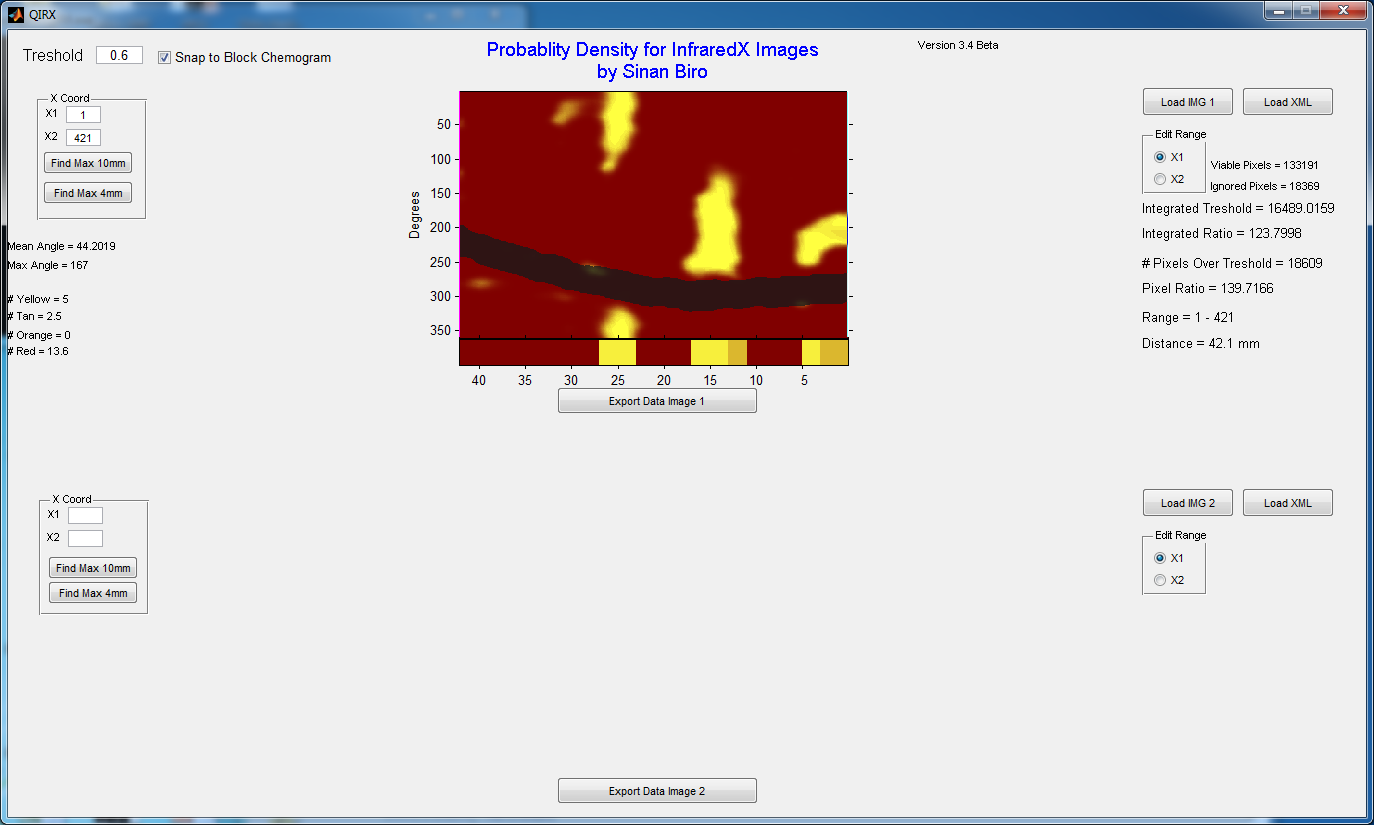

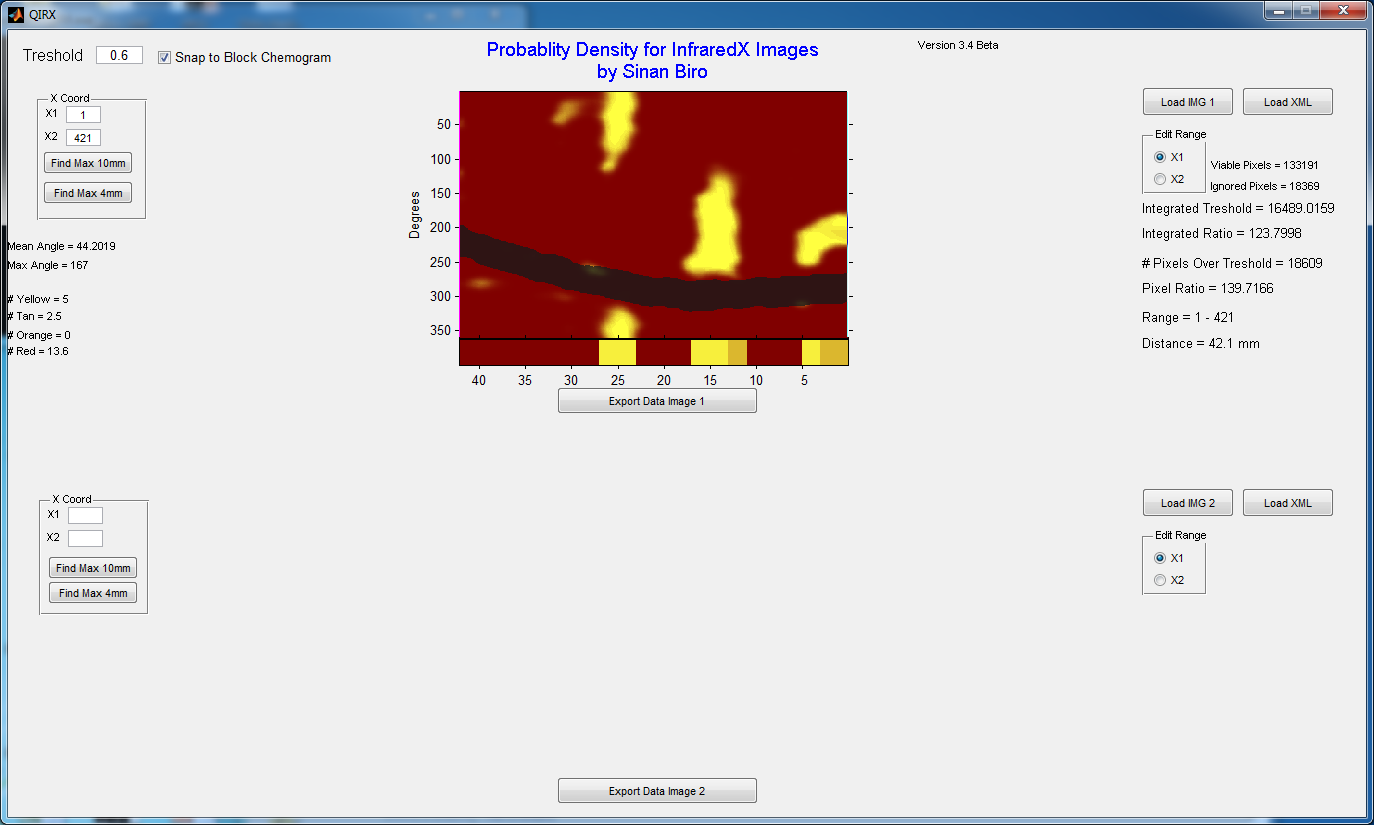


NIRS-LCBI

3) Cross-validation with training set: test set = 4:1

4) IVUS pre-processing by lumen and external elastic membrane (EEM) segmentation using Medical Imaging Interaction Toolkit (MITK)

5) ROI extraction

6) IVUS feature extraction

7) IVUS feature selection by univariable feature selection

8) Supervised machine learning to classify FA vs. non-FA (or TCFA vs. non-TCFA)

: with vs. without stacking of NIRS-LCBI

- K-nearest neighbor

- Support vector machine

- Random forest

- Deep feed=forward neural network

9) Deep learning to classify FA vs. non-FA (or TCFA vs. non-TCFA)

: with vs. without stacking of NIRS-LCBI

- CNN, using inception (Google), ResNet (Microsoft)


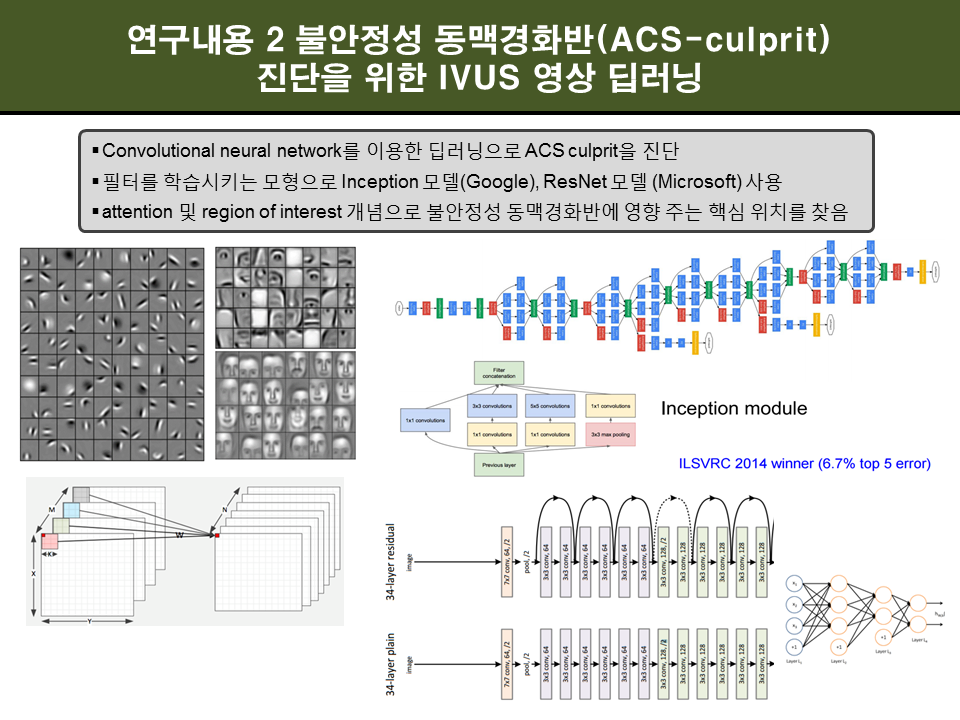

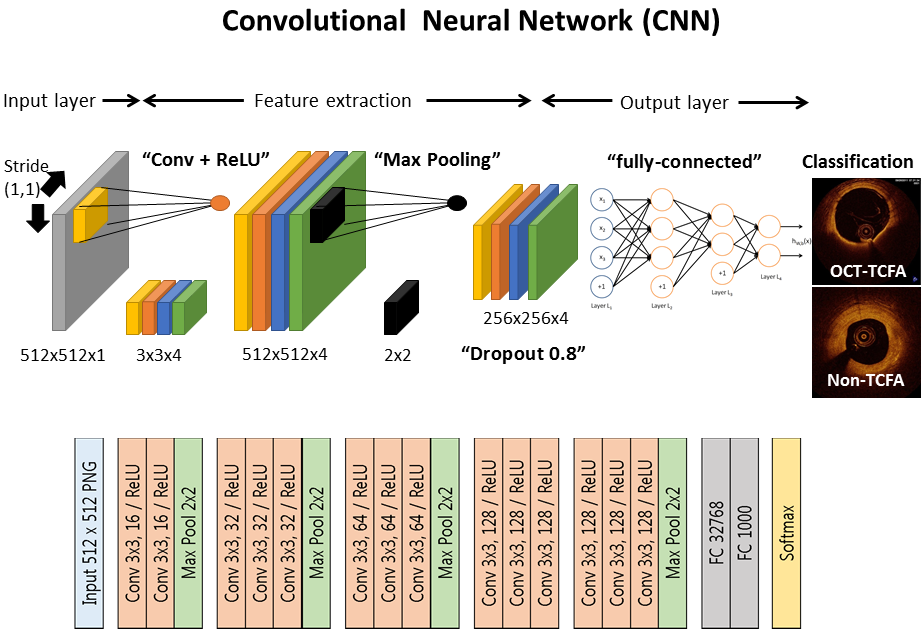


10) Comparison of the performance between the machine/deep learning algorithms of IVUS-NIRS vs. IVUS alone

11) Comparison of the performance of IVUS-NIRS algorithms with IVUS-attenuation, echo lucent plaque, NIRS-LCBI, etc.

**3.3 Clinical validation of the prediction models**

- In ABLE-based 10000 patients

**3.4 Use of clinical information**

- EMR-based clinical information (body weight, height, clinical presentations, symptoms and signs, physical findings, electrocardiography, complete blood count, serum BUN/ creatinine, liver function test, lipid profiles
- Fractional flow reserve
- History of medication
- Coronary CT angiographic findings

**4. End points**

- CT angiography-measured myocardial volume (myocardial territory)
- Fractional flow reserve<0.80 (ischemia-producing lesion)
- OCT-derived thin-cap fibroatheroma (prototype of vulnerable plaque)
- 1-year major adverse cardiac events including cardiac death, myocardial infarction, target-vessel revascularization

**5. Statistical methods**

The statistical analyses for evaluating patient and lesion characteristics at baseline were performed using SPSS (version 10.0, SPSS Inc., Chicago, IL, USA). All values are expressed as means ± 1 standard deviation (continuous variables) or as counts and percentages (categorical variables). Continuous variables were compared using unpaired t-tests; categorical variables were compared using χ2 statistics. A p value <0.05 was considered statistically significant. ROCs were analyzed using MedCalc Software (Mariakerke, Belgium) to assess the best cut-off for angiographic DS or IVUS-measured lumen area to predict FFR <0.80 with maximal accuracy.
