# Supplementary material for: Machine learning assessment of myocardial ischemia using angiography: Development and retrospective validation
Source: PLoS Med. 2018 Nov 13;15(11):e1002693. doi: 10.1371/journal.pmed.1002693 (PMC6233920; doi:10.1371/journal.pmed.1002693)
Supplement: S1 Data — (ZIP) [file pmed.1002693.s011.zip › S1_Data/180827_final_900_sp_ffr_unstack.html]

180827\_final\_900\_sp\_ffr\_unstack


In [1]:

```
import numpy as np
import pandas as pd
from sklearn.metrics import mean_absolute_error,mean_squared_error,median_absolute_error
from sklearn.model_selection import train_test_split, StratifiedKFold, KFold

from sklearn.linear_model import LassoCV, RidgeCV, ElasticNetCV, LinearRegression
from sklearn.ensemble import RandomForestRegressor, ExtraTreesRegressor, GradientBoostingRegressor

from sklearn.metrics import mean_absolute_error,mean_squared_error,median_absolute_error
```

In [2]:

```
!pip install pandas_ml
```

```
Requirement already satisfied: pandas_ml in c:\anaconda3\lib\site-packages (0.5.0)
Requirement already satisfied: pandas>=0.17.0 in c:\anaconda3\lib\site-packages (from pandas_ml) (0.21.0)
Requirement already satisfied: enum34 in c:\anaconda3\lib\site-packages (from pandas_ml) (1.1.6)
Requirement already satisfied: python-dateutil>=2 in c:\anaconda3\lib\site-packages (from pandas>=0.17.0->pandas_ml) (2.6.1)
Requirement already satisfied: numpy>=1.9.0 in c:\anaconda3\lib\site-packages (from pandas>=0.17.0->pandas_ml) (1.14.0)
Requirement already satisfied: pytz>=2011k in c:\anaconda3\lib\site-packages (from pandas>=0.17.0->pandas_ml) (2017.3)
Requirement already satisfied: six>=1.5 in c:\anaconda3\lib\site-packages (from python-dateutil>=2->pandas>=0.17.0->pandas_ml) (1.11.0)
```

```
thrift-sasl 0.2.1 requires sasl>=0.2.1, which is not installed.
pyhs2 0.6.0 requires sasl, which is not installed.
pyhs2 0.6.0 requires thrift, which is not installed.
tensorflow-tensorboard 1.5.1 has requirement bleach==1.5.0, but you'll have bleach 2.1.3 which is incompatible.
tensorflow-tensorboard 1.5.1 has requirement html5lib==0.9999999, but you'll have html5lib 1.0.1 which is incompatible.
crowdai 1.0.10 has requirement Jinja2<2.10,>=2.9.6, but you'll have jinja2 2.10 which is incompatible.
crowdai 1.0.10 has requirement six<1.11,>=1.10, but you'll have six 1.11.0 which is incompatible.
crowdai 1.0.10 has requirement Werkzeug<0.13,>=0.12.1, but you'll have werkzeug 0.14.1 which is incompatible.
docker-py 1.10.3 has requirement requests<2.11,>=2.5.2, but you'll have requests 2.14.2 which is incompatible.
You are using pip version 10.0.1, however version 18.0 is available.
You should consider upgrading via the 'python -m pip install --upgrade pip' command.
```

In [3]:

```
!pip install tqdm
```

```
Requirement already satisfied: tqdm in c:\anaconda3\lib\site-packages (4.11.2)
```

```
thrift-sasl 0.2.1 requires sasl>=0.2.1, which is not installed.
pyhs2 0.6.0 requires sasl, which is not installed.
pyhs2 0.6.0 requires thrift, which is not installed.
tensorflow-tensorboard 1.5.1 has requirement bleach==1.5.0, but you'll have bleach 2.1.3 which is incompatible.
tensorflow-tensorboard 1.5.1 has requirement html5lib==0.9999999, but you'll have html5lib 1.0.1 which is incompatible.
crowdai 1.0.10 has requirement Jinja2<2.10,>=2.9.6, but you'll have jinja2 2.10 which is incompatible.
crowdai 1.0.10 has requirement six<1.11,>=1.10, but you'll have six 1.11.0 which is incompatible.
crowdai 1.0.10 has requirement Werkzeug<0.13,>=0.12.1, but you'll have werkzeug 0.14.1 which is incompatible.
docker-py 1.10.3 has requirement requests<2.11,>=2.5.2, but you'll have requests 2.14.2 which is incompatible.
You are using pip version 10.0.1, however version 18.0 is available.
You should consider upgrading via the 'python -m pip install --upgrade pip' command.
```

In [4]:

```
final_900 = pd.read_csv('subterritory_final_aug28.csv',encoding='cp949')
final_900 = pd.concat([pd.get_dummies(final_900['segment']),final_900],axis=1)
final_900.drop('segment',axis=1,inplace=True)

test_200 = pd.read_csv('./test200.csv')
test_200 = pd.concat([pd.get_dummies(test_200['segment']),test_200],axis=1)
test_200.drop('segment',axis=1,inplace=True)
# test set에는 1st OM 더미변수가 없었기 때문에 전부 0으로 단순히 만들어주었습니다.
test_200['1st OM'] = 0
```

In [5]:

```
features = ['diminutive', 'diam_SB3', 'sp_D_pLAD', 'diam_SB2', 'diam_D2', 'calc_sp_RCA', 'calc_sp_LCX','calc_sp_LAD',
 '%DS', 'Sum_D3+D4+S2', 'Mid LAD', '1st OM', 'sp_D_pLCX', 'Proximal LAD', 'sp_D_LM', 'Apex curve',
 'disal_MLD', 'diam_D1', 'Proximal LCX', 'Proximal RCA', 'Distal RCA', 'diam_SB1', 'diam_D4',
 'Sum_D1+D2+S1', 'Sum_SB2+SB3', 'diam_D3', 'diam_S1', 'aver_MLD', 'age', 'Sum_D1+D2',
 'prox_MLD', 'Mid RCA', 'Distal LAD', 'RI presence', 'lesion_L', 'D_mRCA', 'Distal LCX', 'distance_os_MLD', 'MLD', 'gender', 'diam_S2', 'Sum_D3+D4']
```

# 결측값이 있는 변수¶

In [6]:

```
train_features = final_900[features]
train_features['Sum_D1+D2'] = train_features['diam_D1']+train_features['diam_D2']
train_features['Sum_D1+D2+S1'] = train_features['diam_D1']+train_features['diam_D2']+train_features['diam_S1']
train_features['Sum_D3+D4'] = train_features['diam_D3']+train_features['diam_D4']
train_features['Sum_D3+D4+S2'] = train_features['diam_D3']+train_features['diam_D4']+train_features['diam_S2']
train_features['Sum_SB2+SB3'] = train_features['diam_SB2']+train_features['diam_SB3']
```

```
C:\Anaconda3\lib\site-packages\ipykernel\__main__.py:2: SettingWithCopyWarning: 
A value is trying to be set on a copy of a slice from a DataFrame.
Try using .loc[row_indexer,col_indexer] = value instead

See the caveats in the documentation: http://pandas.pydata.org/pandas-docs/stable/indexing.html#indexing-view-versus-copy
  from ipykernel import kernelapp as app
C:\Anaconda3\lib\site-packages\ipykernel\__main__.py:3: SettingWithCopyWarning: 
A value is trying to be set on a copy of a slice from a DataFrame.
Try using .loc[row_indexer,col_indexer] = value instead

See the caveats in the documentation: http://pandas.pydata.org/pandas-docs/stable/indexing.html#indexing-view-versus-copy
  app.launch_new_instance()
C:\Anaconda3\lib\site-packages\ipykernel\__main__.py:4: SettingWithCopyWarning: 
A value is trying to be set on a copy of a slice from a DataFrame.
Try using .loc[row_indexer,col_indexer] = value instead

See the caveats in the documentation: http://pandas.pydata.org/pandas-docs/stable/indexing.html#indexing-view-versus-copy
C:\Anaconda3\lib\site-packages\ipykernel\__main__.py:5: SettingWithCopyWarning: 
A value is trying to be set on a copy of a slice from a DataFrame.
Try using .loc[row_indexer,col_indexer] = value instead

See the caveats in the documentation: http://pandas.pydata.org/pandas-docs/stable/indexing.html#indexing-view-versus-copy
C:\Anaconda3\lib\site-packages\ipykernel\__main__.py:6: SettingWithCopyWarning: 
A value is trying to be set on a copy of a slice from a DataFrame.
Try using .loc[row_indexer,col_indexer] = value instead

See the caveats in the documentation: http://pandas.pydata.org/pandas-docs/stable/indexing.html#indexing-view-versus-copy
```

In [7]:

```
test_features = test_200[features]
test_features['Sum_D1+D2'] = test_features['diam_D1']+test_features['diam_D2']
test_features['Sum_D1+D2+S1'] = test_features['diam_D1']+test_features['diam_D2']+test_features['diam_S1']
test_features['Sum_D3+D4'] = test_features['diam_D3']+test_features['diam_D4']
test_features['Sum_D3+D4+S2'] = test_features['diam_D3']+test_features['diam_D4']+test_features['diam_S2']
test_features['Sum_SB2+SB3'] = test_features['diam_SB2']+test_features['diam_SB3']
```

```
C:\Anaconda3\lib\site-packages\ipykernel\__main__.py:2: SettingWithCopyWarning: 
A value is trying to be set on a copy of a slice from a DataFrame.
Try using .loc[row_indexer,col_indexer] = value instead

See the caveats in the documentation: http://pandas.pydata.org/pandas-docs/stable/indexing.html#indexing-view-versus-copy
  from ipykernel import kernelapp as app
C:\Anaconda3\lib\site-packages\ipykernel\__main__.py:3: SettingWithCopyWarning: 
A value is trying to be set on a copy of a slice from a DataFrame.
Try using .loc[row_indexer,col_indexer] = value instead

See the caveats in the documentation: http://pandas.pydata.org/pandas-docs/stable/indexing.html#indexing-view-versus-copy
  app.launch_new_instance()
C:\Anaconda3\lib\site-packages\ipykernel\__main__.py:4: SettingWithCopyWarning: 
A value is trying to be set on a copy of a slice from a DataFrame.
Try using .loc[row_indexer,col_indexer] = value instead

See the caveats in the documentation: http://pandas.pydata.org/pandas-docs/stable/indexing.html#indexing-view-versus-copy
C:\Anaconda3\lib\site-packages\ipykernel\__main__.py:5: SettingWithCopyWarning: 
A value is trying to be set on a copy of a slice from a DataFrame.
Try using .loc[row_indexer,col_indexer] = value instead

See the caveats in the documentation: http://pandas.pydata.org/pandas-docs/stable/indexing.html#indexing-view-versus-copy
C:\Anaconda3\lib\site-packages\ipykernel\__main__.py:6: SettingWithCopyWarning: 
A value is trying to be set on a copy of a slice from a DataFrame.
Try using .loc[row_indexer,col_indexer] = value instead

See the caveats in the documentation: http://pandas.pydata.org/pandas-docs/stable/indexing.html#indexing-view-versus-copy
```

In [8]:

```
for col in train_features.columns:
    if train_features[col].isna().sum() > 0:
        print('결측값 있는 변수 : \t',col)
```

```
결측값 있는 변수 : 	 diam_SB3
결측값 있는 변수 : 	 diam_SB2
결측값 있는 변수 : 	 diam_D2
결측값 있는 변수 : 	 Sum_D3+D4+S2
결측값 있는 변수 : 	 diam_D1
결측값 있는 변수 : 	 diam_SB1
결측값 있는 변수 : 	 diam_D4
결측값 있는 변수 : 	 Sum_D1+D2+S1
결측값 있는 변수 : 	 Sum_SB2+SB3
결측값 있는 변수 : 	 diam_D3
결측값 있는 변수 : 	 diam_S1
결측값 있는 변수 : 	 Sum_D1+D2
결측값 있는 변수 : 	 diam_S2
결측값 있는 변수 : 	 Sum_D3+D4
```

In [9]:

```
for col in test_features.columns:
    if test_features[col].isna().sum() > 0:
        print('결측값 있는 변수 : \t',col)
```

```
결측값 있는 변수 : 	 diam_SB3
결측값 있는 변수 : 	 diam_SB2
결측값 있는 변수 : 	 diam_D2
결측값 있는 변수 : 	 Sum_D3+D4+S2
결측값 있는 변수 : 	 diam_D1
결측값 있는 변수 : 	 diam_SB1
결측값 있는 변수 : 	 diam_D4
결측값 있는 변수 : 	 Sum_D1+D2+S1
결측값 있는 변수 : 	 Sum_SB2+SB3
결측값 있는 변수 : 	 diam_D3
결측값 있는 변수 : 	 diam_S1
결측값 있는 변수 : 	 Sum_D1+D2
결측값 있는 변수 : 	 diam_S2
결측값 있는 변수 : 	 Sum_D3+D4
```

In [10]:

```
target = (final_900['FFR']<0.8).astype(int)
test_target = (test_200['FFR']<0.8).astype(int)
```

In [11]:

```
from sklearn.linear_model import LogisticRegressionCV
from sklearn.neural_network import MLPClassifier
from sklearn.neighbors import KNeighborsClassifier
from sklearn.svm import SVC
from sklearn.gaussian_process import GaussianProcessClassifier
from sklearn.gaussian_process.kernels import RBF
from sklearn.tree import DecisionTreeClassifier
from sklearn.ensemble import RandomForestClassifier, AdaBoostClassifier, ExtraTreesClassifier
from sklearn.naive_bayes import GaussianNB
from sklearn.discriminant_analysis import QuadraticDiscriminantAnalysis
import catboost as cgb
import lightgbm as lgb
```

In [12]:

```
classifiers = [
    LogisticRegressionCV(refit=False,n_jobs=-1),
    SVC(kernel="linear", C=0.025,probability=True),
    RandomForestClassifier(max_depth=5, n_estimators=500,n_jobs=-1,random_state=4321),
    AdaBoostClassifier(),
    cgb.CatBoostClassifier(verbose=False,random_seed =4321)
    ]
```

In [13]:

```
import sklearn.metrics as metrics
import matplotlib.pyplot as plt
import pandas_ml as pdml
%matplotlib inline
```

결측값 0으로 대체

In [14]:

```
train_features.fillna(0,inplace=True)
test_features.fillna(0,inplace=True)
```

```
C:\Anaconda3\lib\site-packages\pandas\core\frame.py:3035: SettingWithCopyWarning: 
A value is trying to be set on a copy of a slice from a DataFrame

See the caveats in the documentation: http://pandas.pydata.org/pandas-docs/stable/indexing.html#indexing-view-versus-copy
  downcast=downcast, **kwargs)
```

# test set¶

In [15]:

```
for clf in classifiers:
    print(str(clf))
    clf.fit(train_features, target)
    pred_score = clf.predict_proba(test_features)
    
    fpr, tpr, thresholds = metrics.roc_curve(test_target, pred_score[:,1])
    i = np.arange(len(tpr))
    roc = pd.DataFrame({'fpr' : pd.Series(fpr, index=i),'tpr' : pd.Series(tpr, index = i), '1-fpr' : pd.Series(1-fpr, index = i), 'tf' : pd.Series(tpr - (1-fpr), index = i), 'cutoff value' : pd.Series(thresholds, index = i)})
    print()
    print(roc.ix[(roc.tf-0).abs().argsort()[1]])
    threshold = roc.ix[(roc.tf-0).abs().argsort()[1]]['cutoff value']
    print()
    roc_auc = metrics.auc(fpr, tpr)
    pred = np.where(pred_score>=threshold,1,0)
    stats = pdml.ConfusionMatrix(test_target,pred[:,1]).stats()
    print('{} {} {} {} {} {} {}'.format(threshold.round(2), roc_auc.round(2), stats['TPR'].round(2), stats['TNR'].round(2), 
                                        stats['PPV'].round(2), stats['NPV'].round(2), stats['ACC'].round(2)))
    print()
    plt.plot(fpr, tpr, lw=2, label="ROC curve (area = {roc_auc:.2f})".format(roc_auc=roc_auc))
    plt.plot([0, 1], [0, 1], color='navy', lw=2, linestyle='--')
    plt.xlim([0.0, 1.0])
    plt.ylim([0.0, 1.05])
    plt.xlabel('False Positive Rate')
    plt.ylabel('True Positive Rate')
    plt.title('Receiver operating characteristic')
    plt.legend(loc="lower right")
    plt.show()
    print('----------------------------------------------------------------------------------')
```

```
LogisticRegressionCV(Cs=10, class_weight=None, cv=None, dual=False,
           fit_intercept=True, intercept_scaling=1.0, max_iter=100,
           multi_class='ovr', n_jobs=-1, penalty='l2', random_state=None,
           refit=False, scoring=None, solver='lbfgs', tol=0.0001,
           verbose=0)

1-fpr           0.805556
cutoff value    0.408425
fpr             0.194444
tf             -0.012077
tpr             0.793478
Name: 27, dtype: float64

0.41 0.86 0.79 0.81 0.78 0.82 0.8
```

```
C:\Anaconda3\lib\site-packages\ipykernel\__main__.py:10: DeprecationWarning: 
.ix is deprecated. Please use
.loc for label based indexing or
.iloc for positional indexing

See the documentation here:
http://pandas.pydata.org/pandas-docs/stable/indexing.html#ix-indexer-is-deprecated
```

```
----------------------------------------------------------------------------------
SVC(C=0.025, cache_size=200, class_weight=None, coef0=0.0,
  decision_function_shape='ovr', degree=3, gamma='auto', kernel='linear',
  max_iter=-1, probability=True, random_state=None, shrinking=True,
  tol=0.001, verbose=False)

1-fpr           0.796296
cutoff value    0.379559
fpr             0.203704
tf              0.008052
tpr             0.804348
Name: 31, dtype: float64

0.38 0.87 0.8 0.8 0.77 0.83 0.8
```

```
C:\Anaconda3\lib\site-packages\ipykernel\__main__.py:10: DeprecationWarning: 
.ix is deprecated. Please use
.loc for label based indexing or
.iloc for positional indexing

See the documentation here:
http://pandas.pydata.org/pandas-docs/stable/indexing.html#ix-indexer-is-deprecated
```

```
----------------------------------------------------------------------------------
RandomForestClassifier(bootstrap=True, class_weight=None, criterion='gini',
            max_depth=5, max_features='auto', max_leaf_nodes=None,
            min_impurity_decrease=0.0, min_impurity_split=None,
            min_samples_leaf=1, min_samples_split=2,
            min_weight_fraction_leaf=0.0, n_estimators=500, n_jobs=-1,
            oob_score=False, random_state=4321, verbose=0,
            warm_start=False)

1-fpr           0.805556
cutoff value    0.435541
fpr             0.194444
tf             -0.022947
tpr             0.782609
Name: 36, dtype: float64

0.44 0.84 0.78 0.81 0.77 0.81 0.8
```

```
C:\Anaconda3\lib\site-packages\ipykernel\__main__.py:10: DeprecationWarning: 
.ix is deprecated. Please use
.loc for label based indexing or
.iloc for positional indexing

See the documentation here:
http://pandas.pydata.org/pandas-docs/stable/indexing.html#ix-indexer-is-deprecated
```

```
----------------------------------------------------------------------------------
AdaBoostClassifier(algorithm='SAMME.R', base_estimator=None,
          learning_rate=1.0, n_estimators=50, random_state=None)

1-fpr           0.759259
cutoff value    0.498362
fpr             0.240741
tf             -0.030998
tpr             0.728261
Name: 33, dtype: float64

0.5 0.8 0.73 0.76 0.72 0.77 0.74
```

```
C:\Anaconda3\lib\site-packages\ipykernel\__main__.py:10: DeprecationWarning: 
.ix is deprecated. Please use
.loc for label based indexing or
.iloc for positional indexing

See the documentation here:
http://pandas.pydata.org/pandas-docs/stable/indexing.html#ix-indexer-is-deprecated
```

```
----------------------------------------------------------------------------------
<catboost.core.CatBoostClassifier object at 0x0000021620E03D68>

1-fpr           0.777778
cutoff value    0.397499
fpr             0.222222
tf             -0.027778
tpr             0.750000
Name: 35, dtype: float64

0.4 0.83 0.75 0.78 0.74 0.79 0.76
```

```
----------------------------------------------------------------------------------
```

In [16]:

```
# catboost feature importance
pd.DataFrame(list(zip(classifiers[4].feature_importances_,features))).sort_values(0,ascending=False)
```

Out[16]:

|  | 0 | 1 |
| --- | --- | --- |
| 38 | 12.052636 | MLD |
| 8 | 6.063492 | %DS |
| 28 | 5.860971 | age |
| 14 | 5.383061 | sp\_D\_LM |
| 6 | 4.731414 | calc\_sp\_LCX |
| 34 | 4.550703 | lesion\_L |
| 35 | 4.434396 | D\_mRCA |
| 7 | 4.351088 | calc\_sp\_LAD |
| 37 | 4.346357 | distance\_os\_MLD |
| 12 | 4.327562 | sp\_D\_pLCX |
| 2 | 4.111699 | sp\_D\_pLAD |
| 13 | 4.019445 | Proximal LAD |
| 30 | 3.827336 | prox\_MLD |
| 16 | 3.519124 | disal\_MLD |
| 5 | 3.403391 | calc\_sp\_RCA |
| 27 | 2.898069 | aver\_MLD |
| 25 | 2.736945 | diam\_D3 |
| 9 | 2.316247 | Sum\_D3+D4+S2 |
| 17 | 2.238470 | diam\_D1 |
| 29 | 1.804880 | Sum\_D1+D2 |
| 23 | 1.772796 | Sum\_D1+D2+S1 |
| 41 | 1.561191 | Sum\_D3+D4 |
| 31 | 1.527006 | Mid RCA |
| 39 | 1.481498 | gender |
| 40 | 1.038804 | diam\_S2 |
| 26 | 0.955335 | diam\_S1 |
| 22 | 0.570189 | diam\_D4 |
| 10 | 0.560142 | Mid LAD |
| 4 | 0.494462 | diam\_D2 |
| 0 | 0.407860 | diminutive |
| 19 | 0.381618 | Proximal RCA |
| 21 | 0.380859 | diam\_SB1 |
| 33 | 0.374341 | RI presence |
| 20 | 0.331138 | Distal RCA |
| 3 | 0.303834 | diam\_SB2 |
| 36 | 0.263832 | Distal LCX |
| 24 | 0.249807 | Sum\_SB2+SB3 |
| 15 | 0.126630 | Apex curve |
| 18 | 0.103230 | Proximal LCX |
| 32 | 0.072420 | Distal LAD |
| 11 | 0.044744 | 1st OM |
| 1 | 0.020975 | diam\_SB3 |

# top feature + accuracy¶

In [17]:

```
fi = pd.DataFrame(list(zip(classifiers[4].feature_importances_,features))).sort_values(0,ascending=False)
clf = LogisticRegressionCV(refit=False)
for i in range(1,len(fi[1].values)+1):
    print('top', len(fi[1][:i].values), 'features : \t', fi[1][:i].values)
    clf.fit(train_features[fi[1][:i].values], target)
    pred_score = clf.predict_proba(test_features[fi[1][:i].values])
    pred = np.where(pred_score>=threshold,1,0)
    print("정확도", metrics.accuracy_score(test_target,pred[:,1]))
    print('-'*50)
```

```
top 1 features : 	 ['MLD']
정확도 0.645
--------------------------------------------------
top 2 features : 	 ['MLD' '%DS']
정확도 0.68
--------------------------------------------------
top 3 features : 	 ['MLD' '%DS' 'age']
정확도 0.665
--------------------------------------------------
top 4 features : 	 ['MLD' '%DS' 'age' 'sp_D_LM']
정확도 0.655
--------------------------------------------------
top 5 features : 	 ['MLD' '%DS' 'age' 'sp_D_LM' 'calc_sp_LCX']
정확도 0.655
--------------------------------------------------
top 6 features : 	 ['MLD' '%DS' 'age' 'sp_D_LM' 'calc_sp_LCX' 'lesion_L']
정확도 0.665
--------------------------------------------------
top 7 features : 	 ['MLD' '%DS' 'age' 'sp_D_LM' 'calc_sp_LCX' 'lesion_L' 'D_mRCA']
정확도 0.685
--------------------------------------------------
top 8 features : 	 ['MLD' '%DS' 'age' 'sp_D_LM' 'calc_sp_LCX' 'lesion_L' 'D_mRCA'
 'calc_sp_LAD']
정확도 0.71
--------------------------------------------------
top 9 features : 	 ['MLD' '%DS' 'age' 'sp_D_LM' 'calc_sp_LCX' 'lesion_L' 'D_mRCA'
 'calc_sp_LAD' 'distance_os_MLD']
정확도 0.745
--------------------------------------------------
top 10 features : 	 ['MLD' '%DS' 'age' 'sp_D_LM' 'calc_sp_LCX' 'lesion_L' 'D_mRCA'
 'calc_sp_LAD' 'distance_os_MLD' 'sp_D_pLCX']
정확도 0.745
--------------------------------------------------
top 11 features : 	 ['MLD' '%DS' 'age' 'sp_D_LM' 'calc_sp_LCX' 'lesion_L' 'D_mRCA'
 'calc_sp_LAD' 'distance_os_MLD' 'sp_D_pLCX' 'sp_D_pLAD']
정확도 0.74
--------------------------------------------------
top 12 features : 	 ['MLD' '%DS' 'age' 'sp_D_LM' 'calc_sp_LCX' 'lesion_L' 'D_mRCA'
 'calc_sp_LAD' 'distance_os_MLD' 'sp_D_pLCX' 'sp_D_pLAD' 'Proximal LAD']
정확도 0.805
--------------------------------------------------
top 13 features : 	 ['MLD' '%DS' 'age' 'sp_D_LM' 'calc_sp_LCX' 'lesion_L' 'D_mRCA'
 'calc_sp_LAD' 'distance_os_MLD' 'sp_D_pLCX' 'sp_D_pLAD' 'Proximal LAD'
 'prox_MLD']
정확도 0.8
--------------------------------------------------
top 14 features : 	 ['MLD' '%DS' 'age' 'sp_D_LM' 'calc_sp_LCX' 'lesion_L' 'D_mRCA'
 'calc_sp_LAD' 'distance_os_MLD' 'sp_D_pLCX' 'sp_D_pLAD' 'Proximal LAD'
 'prox_MLD' 'disal_MLD']
정확도 0.805
--------------------------------------------------
top 15 features : 	 ['MLD' '%DS' 'age' 'sp_D_LM' 'calc_sp_LCX' 'lesion_L' 'D_mRCA'
 'calc_sp_LAD' 'distance_os_MLD' 'sp_D_pLCX' 'sp_D_pLAD' 'Proximal LAD'
 'prox_MLD' 'disal_MLD' 'calc_sp_RCA']
정확도 0.8
--------------------------------------------------
top 16 features : 	 ['MLD' '%DS' 'age' 'sp_D_LM' 'calc_sp_LCX' 'lesion_L' 'D_mRCA'
 'calc_sp_LAD' 'distance_os_MLD' 'sp_D_pLCX' 'sp_D_pLAD' 'Proximal LAD'
 'prox_MLD' 'disal_MLD' 'calc_sp_RCA' 'aver_MLD']
정확도 0.8
--------------------------------------------------
top 17 features : 	 ['MLD' '%DS' 'age' 'sp_D_LM' 'calc_sp_LCX' 'lesion_L' 'D_mRCA'
 'calc_sp_LAD' 'distance_os_MLD' 'sp_D_pLCX' 'sp_D_pLAD' 'Proximal LAD'
 'prox_MLD' 'disal_MLD' 'calc_sp_RCA' 'aver_MLD' 'diam_D3']
정확도 0.8
--------------------------------------------------
top 18 features : 	 ['MLD' '%DS' 'age' 'sp_D_LM' 'calc_sp_LCX' 'lesion_L' 'D_mRCA'
 'calc_sp_LAD' 'distance_os_MLD' 'sp_D_pLCX' 'sp_D_pLAD' 'Proximal LAD'
 'prox_MLD' 'disal_MLD' 'calc_sp_RCA' 'aver_MLD' 'diam_D3' 'Sum_D3+D4+S2']
정확도 0.785
--------------------------------------------------
top 19 features : 	 ['MLD' '%DS' 'age' 'sp_D_LM' 'calc_sp_LCX' 'lesion_L' 'D_mRCA'
 'calc_sp_LAD' 'distance_os_MLD' 'sp_D_pLCX' 'sp_D_pLAD' 'Proximal LAD'
 'prox_MLD' 'disal_MLD' 'calc_sp_RCA' 'aver_MLD' 'diam_D3' 'Sum_D3+D4+S2'
 'diam_D1']
정확도 0.79
--------------------------------------------------
top 20 features : 	 ['MLD' '%DS' 'age' 'sp_D_LM' 'calc_sp_LCX' 'lesion_L' 'D_mRCA'
 'calc_sp_LAD' 'distance_os_MLD' 'sp_D_pLCX' 'sp_D_pLAD' 'Proximal LAD'
 'prox_MLD' 'disal_MLD' 'calc_sp_RCA' 'aver_MLD' 'diam_D3' 'Sum_D3+D4+S2'
 'diam_D1' 'Sum_D1+D2']
정확도 0.795
--------------------------------------------------
top 21 features : 	 ['MLD' '%DS' 'age' 'sp_D_LM' 'calc_sp_LCX' 'lesion_L' 'D_mRCA'
 'calc_sp_LAD' 'distance_os_MLD' 'sp_D_pLCX' 'sp_D_pLAD' 'Proximal LAD'
 'prox_MLD' 'disal_MLD' 'calc_sp_RCA' 'aver_MLD' 'diam_D3' 'Sum_D3+D4+S2'
 'diam_D1' 'Sum_D1+D2' 'Sum_D1+D2+S1']
정확도 0.81
--------------------------------------------------
top 22 features : 	 ['MLD' '%DS' 'age' 'sp_D_LM' 'calc_sp_LCX' 'lesion_L' 'D_mRCA'
 'calc_sp_LAD' 'distance_os_MLD' 'sp_D_pLCX' 'sp_D_pLAD' 'Proximal LAD'
 'prox_MLD' 'disal_MLD' 'calc_sp_RCA' 'aver_MLD' 'diam_D3' 'Sum_D3+D4+S2'
 'diam_D1' 'Sum_D1+D2' 'Sum_D1+D2+S1' 'Sum_D3+D4']
정확도 0.815
--------------------------------------------------
top 23 features : 	 ['MLD' '%DS' 'age' 'sp_D_LM' 'calc_sp_LCX' 'lesion_L' 'D_mRCA'
 'calc_sp_LAD' 'distance_os_MLD' 'sp_D_pLCX' 'sp_D_pLAD' 'Proximal LAD'
 'prox_MLD' 'disal_MLD' 'calc_sp_RCA' 'aver_MLD' 'diam_D3' 'Sum_D3+D4+S2'
 'diam_D1' 'Sum_D1+D2' 'Sum_D1+D2+S1' 'Sum_D3+D4' 'Mid RCA']
정확도 0.805
--------------------------------------------------
top 24 features : 	 ['MLD' '%DS' 'age' 'sp_D_LM' 'calc_sp_LCX' 'lesion_L' 'D_mRCA'
 'calc_sp_LAD' 'distance_os_MLD' 'sp_D_pLCX' 'sp_D_pLAD' 'Proximal LAD'
 'prox_MLD' 'disal_MLD' 'calc_sp_RCA' 'aver_MLD' 'diam_D3' 'Sum_D3+D4+S2'
 'diam_D1' 'Sum_D1+D2' 'Sum_D1+D2+S1' 'Sum_D3+D4' 'Mid RCA' 'gender']
정확도 0.815
--------------------------------------------------
top 25 features : 	 ['MLD' '%DS' 'age' 'sp_D_LM' 'calc_sp_LCX' 'lesion_L' 'D_mRCA'
 'calc_sp_LAD' 'distance_os_MLD' 'sp_D_pLCX' 'sp_D_pLAD' 'Proximal LAD'
 'prox_MLD' 'disal_MLD' 'calc_sp_RCA' 'aver_MLD' 'diam_D3' 'Sum_D3+D4+S2'
 'diam_D1' 'Sum_D1+D2' 'Sum_D1+D2+S1' 'Sum_D3+D4' 'Mid RCA' 'gender'
 'diam_S2']
정확도 0.81
--------------------------------------------------
top 26 features : 	 ['MLD' '%DS' 'age' 'sp_D_LM' 'calc_sp_LCX' 'lesion_L' 'D_mRCA'
 'calc_sp_LAD' 'distance_os_MLD' 'sp_D_pLCX' 'sp_D_pLAD' 'Proximal LAD'
 'prox_MLD' 'disal_MLD' 'calc_sp_RCA' 'aver_MLD' 'diam_D3' 'Sum_D3+D4+S2'
 'diam_D1' 'Sum_D1+D2' 'Sum_D1+D2+S1' 'Sum_D3+D4' 'Mid RCA' 'gender'
 'diam_S2' 'diam_S1']
정확도 0.79
--------------------------------------------------
top 27 features : 	 ['MLD' '%DS' 'age' 'sp_D_LM' 'calc_sp_LCX' 'lesion_L' 'D_mRCA'
 'calc_sp_LAD' 'distance_os_MLD' 'sp_D_pLCX' 'sp_D_pLAD' 'Proximal LAD'
 'prox_MLD' 'disal_MLD' 'calc_sp_RCA' 'aver_MLD' 'diam_D3' 'Sum_D3+D4+S2'
 'diam_D1' 'Sum_D1+D2' 'Sum_D1+D2+S1' 'Sum_D3+D4' 'Mid RCA' 'gender'
 'diam_S2' 'diam_S1' 'diam_D4']
정확도 0.815
--------------------------------------------------
top 28 features : 	 ['MLD' '%DS' 'age' 'sp_D_LM' 'calc_sp_LCX' 'lesion_L' 'D_mRCA'
 'calc_sp_LAD' 'distance_os_MLD' 'sp_D_pLCX' 'sp_D_pLAD' 'Proximal LAD'
 'prox_MLD' 'disal_MLD' 'calc_sp_RCA' 'aver_MLD' 'diam_D3' 'Sum_D3+D4+S2'
 'diam_D1' 'Sum_D1+D2' 'Sum_D1+D2+S1' 'Sum_D3+D4' 'Mid RCA' 'gender'
 'diam_S2' 'diam_S1' 'diam_D4' 'Mid LAD']
정확도 0.775
--------------------------------------------------
top 29 features : 	 ['MLD' '%DS' 'age' 'sp_D_LM' 'calc_sp_LCX' 'lesion_L' 'D_mRCA'
 'calc_sp_LAD' 'distance_os_MLD' 'sp_D_pLCX' 'sp_D_pLAD' 'Proximal LAD'
 'prox_MLD' 'disal_MLD' 'calc_sp_RCA' 'aver_MLD' 'diam_D3' 'Sum_D3+D4+S2'
 'diam_D1' 'Sum_D1+D2' 'Sum_D1+D2+S1' 'Sum_D3+D4' 'Mid RCA' 'gender'
 'diam_S2' 'diam_S1' 'diam_D4' 'Mid LAD' 'diam_D2']
정확도 0.775
--------------------------------------------------
top 30 features : 	 ['MLD' '%DS' 'age' 'sp_D_LM' 'calc_sp_LCX' 'lesion_L' 'D_mRCA'
 'calc_sp_LAD' 'distance_os_MLD' 'sp_D_pLCX' 'sp_D_pLAD' 'Proximal LAD'
 'prox_MLD' 'disal_MLD' 'calc_sp_RCA' 'aver_MLD' 'diam_D3' 'Sum_D3+D4+S2'
 'diam_D1' 'Sum_D1+D2' 'Sum_D1+D2+S1' 'Sum_D3+D4' 'Mid RCA' 'gender'
 'diam_S2' 'diam_S1' 'diam_D4' 'Mid LAD' 'diam_D2' 'diminutive']
정확도 0.79
--------------------------------------------------
top 31 features : 	 ['MLD' '%DS' 'age' 'sp_D_LM' 'calc_sp_LCX' 'lesion_L' 'D_mRCA'
 'calc_sp_LAD' 'distance_os_MLD' 'sp_D_pLCX' 'sp_D_pLAD' 'Proximal LAD'
 'prox_MLD' 'disal_MLD' 'calc_sp_RCA' 'aver_MLD' 'diam_D3' 'Sum_D3+D4+S2'
 'diam_D1' 'Sum_D1+D2' 'Sum_D1+D2+S1' 'Sum_D3+D4' 'Mid RCA' 'gender'
 'diam_S2' 'diam_S1' 'diam_D4' 'Mid LAD' 'diam_D2' 'diminutive'
 'Proximal RCA']
정확도 0.795
--------------------------------------------------
top 32 features : 	 ['MLD' '%DS' 'age' 'sp_D_LM' 'calc_sp_LCX' 'lesion_L' 'D_mRCA'
 'calc_sp_LAD' 'distance_os_MLD' 'sp_D_pLCX' 'sp_D_pLAD' 'Proximal LAD'
 'prox_MLD' 'disal_MLD' 'calc_sp_RCA' 'aver_MLD' 'diam_D3' 'Sum_D3+D4+S2'
 'diam_D1' 'Sum_D1+D2' 'Sum_D1+D2+S1' 'Sum_D3+D4' 'Mid RCA' 'gender'
 'diam_S2' 'diam_S1' 'diam_D4' 'Mid LAD' 'diam_D2' 'diminutive'
 'Proximal RCA' 'diam_SB1']
정확도 0.795
--------------------------------------------------
top 33 features : 	 ['MLD' '%DS' 'age' 'sp_D_LM' 'calc_sp_LCX' 'lesion_L' 'D_mRCA'
 'calc_sp_LAD' 'distance_os_MLD' 'sp_D_pLCX' 'sp_D_pLAD' 'Proximal LAD'
 'prox_MLD' 'disal_MLD' 'calc_sp_RCA' 'aver_MLD' 'diam_D3' 'Sum_D3+D4+S2'
 'diam_D1' 'Sum_D1+D2' 'Sum_D1+D2+S1' 'Sum_D3+D4' 'Mid RCA' 'gender'
 'diam_S2' 'diam_S1' 'diam_D4' 'Mid LAD' 'diam_D2' 'diminutive'
 'Proximal RCA' 'diam_SB1' 'RI presence']
정확도 0.79
--------------------------------------------------
top 34 features : 	 ['MLD' '%DS' 'age' 'sp_D_LM' 'calc_sp_LCX' 'lesion_L' 'D_mRCA'
 'calc_sp_LAD' 'distance_os_MLD' 'sp_D_pLCX' 'sp_D_pLAD' 'Proximal LAD'
 'prox_MLD' 'disal_MLD' 'calc_sp_RCA' 'aver_MLD' 'diam_D3' 'Sum_D3+D4+S2'
 'diam_D1' 'Sum_D1+D2' 'Sum_D1+D2+S1' 'Sum_D3+D4' 'Mid RCA' 'gender'
 'diam_S2' 'diam_S1' 'diam_D4' 'Mid LAD' 'diam_D2' 'diminutive'
 'Proximal RCA' 'diam_SB1' 'RI presence' 'Distal RCA']
정확도 0.805
--------------------------------------------------
top 35 features : 	 ['MLD' '%DS' 'age' 'sp_D_LM' 'calc_sp_LCX' 'lesion_L' 'D_mRCA'
 'calc_sp_LAD' 'distance_os_MLD' 'sp_D_pLCX' 'sp_D_pLAD' 'Proximal LAD'
 'prox_MLD' 'disal_MLD' 'calc_sp_RCA' 'aver_MLD' 'diam_D3' 'Sum_D3+D4+S2'
 'diam_D1' 'Sum_D1+D2' 'Sum_D1+D2+S1' 'Sum_D3+D4' 'Mid RCA' 'gender'
 'diam_S2' 'diam_S1' 'diam_D4' 'Mid LAD' 'diam_D2' 'diminutive'
 'Proximal RCA' 'diam_SB1' 'RI presence' 'Distal RCA' 'diam_SB2']
정확도 0.79
--------------------------------------------------
top 36 features : 	 ['MLD' '%DS' 'age' 'sp_D_LM' 'calc_sp_LCX' 'lesion_L' 'D_mRCA'
 'calc_sp_LAD' 'distance_os_MLD' 'sp_D_pLCX' 'sp_D_pLAD' 'Proximal LAD'
 'prox_MLD' 'disal_MLD' 'calc_sp_RCA' 'aver_MLD' 'diam_D3' 'Sum_D3+D4+S2'
 'diam_D1' 'Sum_D1+D2' 'Sum_D1+D2+S1' 'Sum_D3+D4' 'Mid RCA' 'gender'
 'diam_S2' 'diam_S1' 'diam_D4' 'Mid LAD' 'diam_D2' 'diminutive'
 'Proximal RCA' 'diam_SB1' 'RI presence' 'Distal RCA' 'diam_SB2'
 'Distal LCX']
정확도 0.795
--------------------------------------------------
top 37 features : 	 ['MLD' '%DS' 'age' 'sp_D_LM' 'calc_sp_LCX' 'lesion_L' 'D_mRCA'
 'calc_sp_LAD' 'distance_os_MLD' 'sp_D_pLCX' 'sp_D_pLAD' 'Proximal LAD'
 'prox_MLD' 'disal_MLD' 'calc_sp_RCA' 'aver_MLD' 'diam_D3' 'Sum_D3+D4+S2'
 'diam_D1' 'Sum_D1+D2' 'Sum_D1+D2+S1' 'Sum_D3+D4' 'Mid RCA' 'gender'
 'diam_S2' 'diam_S1' 'diam_D4' 'Mid LAD' 'diam_D2' 'diminutive'
 'Proximal RCA' 'diam_SB1' 'RI presence' 'Distal RCA' 'diam_SB2'
 'Distal LCX' 'Sum_SB2+SB3']
정확도 0.79
--------------------------------------------------
top 38 features : 	 ['MLD' '%DS' 'age' 'sp_D_LM' 'calc_sp_LCX' 'lesion_L' 'D_mRCA'
 'calc_sp_LAD' 'distance_os_MLD' 'sp_D_pLCX' 'sp_D_pLAD' 'Proximal LAD'
 'prox_MLD' 'disal_MLD' 'calc_sp_RCA' 'aver_MLD' 'diam_D3' 'Sum_D3+D4+S2'
 'diam_D1' 'Sum_D1+D2' 'Sum_D1+D2+S1' 'Sum_D3+D4' 'Mid RCA' 'gender'
 'diam_S2' 'diam_S1' 'diam_D4' 'Mid LAD' 'diam_D2' 'diminutive'
 'Proximal RCA' 'diam_SB1' 'RI presence' 'Distal RCA' 'diam_SB2'
 'Distal LCX' 'Sum_SB2+SB3' 'Apex curve']
정확도 0.79
--------------------------------------------------
top 39 features : 	 ['MLD' '%DS' 'age' 'sp_D_LM' 'calc_sp_LCX' 'lesion_L' 'D_mRCA'
 'calc_sp_LAD' 'distance_os_MLD' 'sp_D_pLCX' 'sp_D_pLAD' 'Proximal LAD'
 'prox_MLD' 'disal_MLD' 'calc_sp_RCA' 'aver_MLD' 'diam_D3' 'Sum_D3+D4+S2'
 'diam_D1' 'Sum_D1+D2' 'Sum_D1+D2+S1' 'Sum_D3+D4' 'Mid RCA' 'gender'
 'diam_S2' 'diam_S1' 'diam_D4' 'Mid LAD' 'diam_D2' 'diminutive'
 'Proximal RCA' 'diam_SB1' 'RI presence' 'Distal RCA' 'diam_SB2'
 'Distal LCX' 'Sum_SB2+SB3' 'Apex curve' 'Proximal LCX']
정확도 0.8
--------------------------------------------------
top 40 features : 	 ['MLD' '%DS' 'age' 'sp_D_LM' 'calc_sp_LCX' 'lesion_L' 'D_mRCA'
 'calc_sp_LAD' 'distance_os_MLD' 'sp_D_pLCX' 'sp_D_pLAD' 'Proximal LAD'
 'prox_MLD' 'disal_MLD' 'calc_sp_RCA' 'aver_MLD' 'diam_D3' 'Sum_D3+D4+S2'
 'diam_D1' 'Sum_D1+D2' 'Sum_D1+D2+S1' 'Sum_D3+D4' 'Mid RCA' 'gender'
 'diam_S2' 'diam_S1' 'diam_D4' 'Mid LAD' 'diam_D2' 'diminutive'
 'Proximal RCA' 'diam_SB1' 'RI presence' 'Distal RCA' 'diam_SB2'
 'Distal LCX' 'Sum_SB2+SB3' 'Apex curve' 'Proximal LCX' 'Distal LAD']
정확도 0.79
--------------------------------------------------
top 41 features : 	 ['MLD' '%DS' 'age' 'sp_D_LM' 'calc_sp_LCX' 'lesion_L' 'D_mRCA'
 'calc_sp_LAD' 'distance_os_MLD' 'sp_D_pLCX' 'sp_D_pLAD' 'Proximal LAD'
 'prox_MLD' 'disal_MLD' 'calc_sp_RCA' 'aver_MLD' 'diam_D3' 'Sum_D3+D4+S2'
 'diam_D1' 'Sum_D1+D2' 'Sum_D1+D2+S1' 'Sum_D3+D4' 'Mid RCA' 'gender'
 'diam_S2' 'diam_S1' 'diam_D4' 'Mid LAD' 'diam_D2' 'diminutive'
 'Proximal RCA' 'diam_SB1' 'RI presence' 'Distal RCA' 'diam_SB2'
 'Distal LCX' 'Sum_SB2+SB3' 'Apex curve' 'Proximal LCX' 'Distal LAD'
 '1st OM']
정확도 0.795
--------------------------------------------------
top 42 features : 	 ['MLD' '%DS' 'age' 'sp_D_LM' 'calc_sp_LCX' 'lesion_L' 'D_mRCA'
 'calc_sp_LAD' 'distance_os_MLD' 'sp_D_pLCX' 'sp_D_pLAD' 'Proximal LAD'
 'prox_MLD' 'disal_MLD' 'calc_sp_RCA' 'aver_MLD' 'diam_D3' 'Sum_D3+D4+S2'
 'diam_D1' 'Sum_D1+D2' 'Sum_D1+D2+S1' 'Sum_D3+D4' 'Mid RCA' 'gender'
 'diam_S2' 'diam_S1' 'diam_D4' 'Mid LAD' 'diam_D2' 'diminutive'
 'Proximal RCA' 'diam_SB1' 'RI presence' 'Distal RCA' 'diam_SB2'
 'Distal LCX' 'Sum_SB2+SB3' 'Apex curve' 'Proximal LCX' 'Distal LAD'
 '1st OM' 'diam_SB3']
정확도 0.79
--------------------------------------------------
```

# train set cv¶

In [18]:

```
from sklearn.model_selection import StratifiedKFold
skf = StratifiedKFold(n_splits=5,random_state=4321,shuffle=True)
```

In [19]:

```
for clf in classifiers:
    t = 1
    print(str(clf))
    ci = {'accuracy':[],'sensitivity':[],'specificity':[],'PPV':[],'NPV':[],'auc':[],'threshold':[]}
    for train_index, test_index in skf.split(train_features, target):
        print("그룹 %s" % t)
        X_train = train_features.values[train_index]
        X_test = train_features.values[test_index]
        y_train = target.values[train_index]
        y_test = target.values[test_index]
        clf.fit(X_train, y_train)
        pred_score = clf.predict_proba(X_test)
        fpr, tpr, thresholds = metrics.roc_curve(y_test, pred_score[:,1])
        i = np.arange(len(tpr))
        roc = pd.DataFrame({'fpr' : pd.Series(fpr, index=i),'tpr' : pd.Series(tpr, index = i), '1-fpr' : pd.Series(1-fpr, index = i), 'tf' : pd.Series(tpr - (1-fpr), index = i), 'cutoff value' : pd.Series(thresholds, index = i)})
        print()
        print(roc.iloc[(roc.tf-0).abs().argsort()[1]])
        threshold = roc.iloc[(roc.tf-0).abs().argsort()[1]]['cutoff value']
        ci['threshold'].append(threshold)
        print()
        roc_auc = metrics.auc(fpr, tpr)
        pred = np.where(pred_score>=threshold,1,0)
        ci['auc'].append(roc_auc)
        stats = pdml.ConfusionMatrix(y_test,pred[:,1]).stats()
        ci['sensitivity'].append(stats['TPR'])
        ci['specificity'].append(stats['TNR'])
        ci['PPV'].append(stats['PPV'])
        ci['NPV'].append(stats['NPV'])
        ci['accuracy'].append(stats['ACC'])
        print(pdml.ConfusionMatrix(y_test,pred[:,1]).print_stats())
        print()
        plt.plot(fpr, tpr, lw=2, label="Group {t} ROC curve (area = {roc_auc:.2f})".format(t=t,roc_auc=roc_auc))
        plt.plot([0, 1], [0, 1], color='navy', lw=2, linestyle='--')
        plt.xlim([0.0, 1.0])
        plt.ylim([0.0, 1.05])
        plt.xlabel('False Positive Rate')
        plt.ylabel('True Positive Rate')
        plt.title('Receiver operating characteristic')
        plt.legend(loc="lower right")
        t += 1
    plt.show()
    print("평균적인 threshold, {} ({}) [{}-{}]".format(np.mean(ci['threshold']).round(2), np.std(ci['threshold']).round(2), 
          np.percentile(ci['threshold'],2.5).round(2), np.percentile(ci['threshold'],97.5).round(2)))
    print("평균적인 auc, {} ({}) [{}-{}]".format(np.mean(ci['auc']).round(2), np.std(ci['auc']).round(2), 
          np.percentile(ci['auc'],2.5).round(2), np.percentile(ci['auc'],97.5).round(2)))
    print("평균적인 sensitivity, {} ({}) [{}-{}]".format(np.mean(ci['sensitivity']).round(2), np.std(ci['sensitivity']).round(2), 
          np.percentile(ci['sensitivity'],2.5).round(2), np.percentile(ci['sensitivity'],97.5).round(2)))
    print("평균적인 specificity, {} ({}) [{}-{}]".format(np.mean(ci['specificity']).round(2), np.std(ci['specificity']).round(2), 
          np.percentile(ci['specificity'],2.5).round(2), np.percentile(ci['specificity'],97.5).round(2)))
    print("평균적인 ppv, {} ({}) [{}-{}]".format(np.mean(ci['PPV']).round(2), np.std(ci['PPV']).round(2), 
          np.percentile(ci['PPV'],2.5).round(2), np.percentile(ci['PPV'],97.5).round(2)))
    print("평균적인 npv, {} ({}) [{}-{}]".format(np.mean(ci['NPV']).round(2), np.std(ci['NPV']).round(2), 
          np.percentile(ci['NPV'],2.5).round(2), np.percentile(ci['NPV'],97.5).round(2)))
    print("평균적인 accuracy, {} ({}) [{}-{}]".format(np.mean(ci['accuracy']).round(2), np.std(ci['accuracy']).round(2), 
          np.percentile(ci['accuracy'],2.5).round(2), np.percentile(ci['accuracy'],97.5).round(2)))
    print('----------------------------------------------------------------------------------')
```

```
LogisticRegressionCV(Cs=10, class_weight=None, cv=None, dual=False,
           fit_intercept=True, intercept_scaling=1.0, max_iter=100,
           multi_class='ovr', n_jobs=-1, penalty='l2', random_state=None,
           refit=False, scoring=None, solver='lbfgs', tol=0.0001,
           verbose=0)
그룹 1

1-fpr           0.752294
cutoff value    0.438741
fpr             0.247706
tf             -0.021524
tpr             0.730769
Name: 37, dtype: float64

population: 187
P: 78
N: 109
PositiveTest: 84
NegativeTest: 103
TP: 57
TN: 82
FP: 27
FN: 21
TPR: 0.7307692307692307
TNR: 0.7522935779816514
PPV: 0.6785714285714286
NPV: 0.7961165048543689
FPR: 0.24770642201834864
FDR: 0.32142857142857145
FNR: 0.2692307692307692
ACC: 0.7433155080213903
F1_score: 0.7037037037037037
MCC: 0.47885706259886934
informedness: 0.48306280875088214
markedness: 0.4746879334257974
prevalence: 0.41711229946524064
LRP: 2.95014245014245
LRN: 0.3578799249530957
DOR: 8.243386243386244
FOR: 0.20388349514563106
None

그룹 2

1-fpr           0.660550
cutoff value    0.379125
fpr             0.339450
tf              0.006116
tpr             0.666667
Name: 44, dtype: float64

population: 187
P: 78
N: 109
PositiveTest: 89
NegativeTest: 98
TP: 52
TN: 72
FP: 37
FN: 26
TPR: 0.6666666666666666
TNR: 0.6605504587155964
PPV: 0.5842696629213483
NPV: 0.7346938775510204
FPR: 0.3394495412844037
FDR: 0.4157303370786517
FNR: 0.3333333333333333
ACC: 0.6631016042780749
F1_score: 0.6227544910179641
MCC: 0.32306397635006845
informedness: 0.3272171253822629
markedness: 0.31896354047236875
prevalence: 0.41711229946524064
LRP: 1.9639639639639637
LRN: 0.5046296296296295
DOR: 3.891891891891892
FOR: 0.2653061224489796
None

그룹 3

1-fpr           0.743119
cutoff value    0.368210
fpr             0.256881
tf              0.026112
tpr             0.769231
Name: 32, dtype: float64

population: 187
P: 78
N: 109
PositiveTest: 88
NegativeTest: 99
TP: 60
TN: 81
FP: 28
FN: 18
TPR: 0.7692307692307693
TNR: 0.7431192660550459
PPV: 0.6818181818181818
NPV: 0.8181818181818182
FPR: 0.25688073394495414
FDR: 0.3181818181818182
FNR: 0.23076923076923078
ACC: 0.7540106951871658
F1_score: 0.7228915662650602
MCC: 0.5061373505708777
informedness: 0.5123500352858152
markedness: 0.5
prevalence: 0.41711229946524064
LRP: 2.9945054945054945
LRN: 0.31054131054131057
DOR: 9.642857142857142
FOR: 0.18181818181818182
None

그룹 4

1-fpr           0.788991
cutoff value    0.445724
fpr             0.211009
tf              0.016204
tpr             0.805195
Name: 32, dtype: float64

population: 186
P: 77
N: 109
PositiveTest: 85
NegativeTest: 101
TP: 62
TN: 86
FP: 23
FN: 15
TPR: 0.8051948051948052
TNR: 0.7889908256880734
PPV: 0.7294117647058823
NPV: 0.8514851485148515
FPR: 0.21100917431192662
FDR: 0.27058823529411763
FNR: 0.19480519480519481
ACC: 0.7956989247311828
F1_score: 0.7654320987654321
MCC: 0.5875037011457702
informedness: 0.5941856308828788
markedness: 0.5808969132207338
prevalence: 0.41397849462365593
LRP: 3.815923207227555
LRN: 0.2469042585321655
DOR: 15.455072463768117
FOR: 0.1485148514851485
None

그룹 5

1-fpr           0.750000
cutoff value    0.429523
fpr             0.250000
tf             -0.022727
tpr             0.727273
Name: 40, dtype: float64

population: 185
P: 77
N: 108
PositiveTest: 83
NegativeTest: 102
TP: 56
TN: 81
FP: 27
FN: 21
TPR: 0.7272727272727273
TNR: 0.75
PPV: 0.6746987951807228
NPV: 0.7941176470588235
FPR: 0.25
FDR: 0.3253012048192771
FNR: 0.2727272727272727
ACC: 0.7405405405405405
F1_score: 0.7
MCC: 0.47302568849689897
informedness: 0.4772727272727273
markedness: 0.4688164422395462
prevalence: 0.41621621621621624
LRP: 2.909090909090909
LRN: 0.3636363636363636
DOR: 8.000000000000002
FOR: 0.20588235294117646
None
```

```
평균적인 threshold, 0.41 (0.03) [0.37-0.45]
평균적인 auc, 0.81 (0.04) [0.75-0.86]
평균적인 sensitivity, 0.74 (0.05) [0.67-0.8]
평균적인 specificity, 0.74 (0.04) [0.67-0.79]
평균적인 ppv, 0.67 (0.05) [0.59-0.72]
평균적인 npv, 0.8 (0.04) [0.74-0.85]
평균적인 accuracy, 0.74 (0.04) [0.67-0.79]
----------------------------------------------------------------------------------
SVC(C=0.025, cache_size=200, class_weight=None, coef0=0.0,
  decision_function_shape='ovr', degree=3, gamma='auto', kernel='linear',
  max_iter=-1, probability=True, random_state=None, shrinking=True,
  tol=0.001, verbose=False)
그룹 1

1-fpr           0.715596
cutoff value    0.441650
fpr             0.284404
tf             -0.010468
tpr             0.705128
Name: 39, dtype: float64

population: 187
P: 78
N: 109
PositiveTest: 86
NegativeTest: 101
TP: 55
TN: 78
FP: 31
FN: 23
TPR: 0.7051282051282052
TNR: 0.7155963302752294
PPV: 0.6395348837209303
NPV: 0.7722772277227723
FPR: 0.28440366972477066
FDR: 0.36046511627906974
FNR: 0.2948717948717949
ACC: 0.7112299465240641
F1_score: 0.6707317073170732
MCC: 0.4162444705466478
informedness: 0.4207245354034346
markedness: 0.41181211144370256
prevalence: 0.41711229946524064
LRP: 2.479321753515302
LRN: 0.41206443129520054
DOR: 6.016830294530154
FOR: 0.22772277227722773
None

그룹 2

1-fpr           0.678899
cutoff value    0.400457
fpr             0.321101
tf             -0.037873
tpr             0.641026
Name: 39, dtype: float64

population: 187
P: 78
N: 109
PositiveTest: 85
NegativeTest: 102
TP: 50
TN: 74
FP: 35
FN: 28
TPR: 0.6410256410256411
TNR: 0.6788990825688074
PPV: 0.5882352941176471
NPV: 0.7254901960784313
FPR: 0.3211009174311927
FDR: 0.4117647058823529
FNR: 0.358974358974359
ACC: 0.6631016042780749
F1_score: 0.6134969325153374
MCC: 0.31680994418659464
informedness: 0.31992472359444846
markedness: 0.3137254901960784
prevalence: 0.41711229946524064
LRP: 1.9963369963369964
LRN: 0.5287595287595287
DOR: 3.775510204081633
FOR: 0.27450980392156865
None

그룹 3

1-fpr           0.770642
cutoff value    0.411999
fpr             0.229358
tf             -0.027052
tpr             0.743590
Name: 31, dtype: float64

population: 187
P: 78
N: 109
PositiveTest: 83
NegativeTest: 104
TP: 58
TN: 84
FP: 25
FN: 20
TPR: 0.7435897435897436
TNR: 0.7706422018348624
PPV: 0.6987951807228916
NPV: 0.8076923076923077
FPR: 0.22935779816513763
FDR: 0.30120481927710846
FNR: 0.2564102564102564
ACC: 0.7593582887700535
F1_score: 0.7204968944099379
MCC: 0.5103450269190154
informedness: 0.514231945424606
markedness: 0.5064874884151993
prevalence: 0.41711229946524064
LRP: 3.242051282051282
LRN: 0.33272283272283265
DOR: 9.744000000000002
FOR: 0.19230769230769232
None

그룹 4

1-fpr           0.779817
cutoff value    0.434847
fpr             0.220183
tf              0.025378
tpr             0.805195
Name: 30, dtype: float64

population: 186
P: 77
N: 109
PositiveTest: 86
NegativeTest: 100
TP: 62
TN: 85
FP: 24
FN: 15
TPR: 0.8051948051948052
TNR: 0.7798165137614679
PPV: 0.7209302325581395
NPV: 0.85
FPR: 0.22018348623853212
FDR: 0.27906976744186046
FNR: 0.19480519480519481
ACC: 0.7903225806451613
F1_score: 0.7607361963190185
MCC: 0.5779278920253365
informedness: 0.5850113189562731
markedness: 0.5709302325581396
prevalence: 0.41397849462365593
LRP: 3.656926406926407
LRN: 0.24980901451489687
DOR: 14.63888888888889
FOR: 0.15
None

그룹 5

1-fpr           0.768519
cutoff value    0.425650
fpr             0.231481
tf             -0.028259
tpr             0.740260
Name: 34, dtype: float64

population: 185
P: 77
N: 108
PositiveTest: 82
NegativeTest: 103
TP: 57
TN: 83
FP: 25
FN: 20
TPR: 0.7402597402597403
TNR: 0.7685185185185185
PPV: 0.6951219512195121
NPV: 0.8058252427184466
FPR: 0.23148148148148148
FDR: 0.3048780487804878
FNR: 0.2597402597402597
ACC: 0.7567567567567568
F1_score: 0.7169811320754716
MCC: 0.5048475424042485
informedness: 0.5087782587782588
markedness: 0.5009471939379586
prevalence: 0.41621621621621624
LRP: 3.197922077922078
LRN: 0.33797527773431385
DOR: 9.462
FOR: 0.1941747572815534
None
```

```
평균적인 threshold, 0.42 (0.01) [0.4-0.44]
평균적인 auc, 0.81 (0.04) [0.74-0.85]
평균적인 sensitivity, 0.73 (0.05) [0.65-0.8]
평균적인 specificity, 0.74 (0.04) [0.68-0.78]
평균적인 ppv, 0.67 (0.05) [0.59-0.72]
평균적인 npv, 0.79 (0.04) [0.73-0.85]
평균적인 accuracy, 0.74 (0.04) [0.67-0.79]
----------------------------------------------------------------------------------
RandomForestClassifier(bootstrap=True, class_weight=None, criterion='gini',
            max_depth=5, max_features='auto', max_leaf_nodes=None,
            min_impurity_decrease=0.0, min_impurity_split=None,
            min_samples_leaf=1, min_samples_split=2,
            min_weight_fraction_leaf=0.0, n_estimators=500, n_jobs=-1,
            oob_score=False, random_state=4321, verbose=0,
            warm_start=False)
그룹 1

1-fpr           0.697248
cutoff value    0.452134
fpr             0.302752
tf              0.020701
tpr             0.717949
Name: 34, dtype: float64

population: 187
P: 78
N: 109
PositiveTest: 89
NegativeTest: 98
TP: 56
TN: 76
FP: 33
FN: 22
TPR: 0.717948717948718
TNR: 0.6972477064220184
PPV: 0.6292134831460674
NPV: 0.7755102040816326
FPR: 0.30275229357798167
FDR: 0.3707865168539326
FNR: 0.28205128205128205
ACC: 0.7058823529411765
F1_score: 0.6706586826347305
MCC: 0.40992661269437153
informedness: 0.41519642437073623
markedness: 0.4047236872277
prevalence: 0.41711229946524064
LRP: 2.3714063714063713
LRN: 0.4045209176788124
DOR: 5.862258953168045
FOR: 0.22448979591836735
None

그룹 2

1-fpr           0.651376
cutoff value    0.399567
fpr             0.348624
tf             -0.010351
tpr             0.641026
Name: 38, dtype: float64

population: 187
P: 78
N: 109
PositiveTest: 88
NegativeTest: 99
TP: 50
TN: 71
FP: 38
FN: 28
TPR: 0.6410256410256411
TNR: 0.6513761467889908
PPV: 0.5681818181818182
NPV: 0.7171717171717171
FPR: 0.3486238532110092
FDR: 0.4318181818181818
FNR: 0.358974358974359
ACC: 0.6470588235294118
F1_score: 0.6024096385542169
MCC: 0.28885616471974335
informedness: 0.2924017878146319
markedness: 0.28535353535353547
prevalence: 0.41711229946524064
LRP: 1.8387314439946019
LRN: 0.5511014806789455
DOR: 3.3364661654135337
FOR: 0.2828282828282828
None

그룹 3

1-fpr           0.715596
cutoff value    0.414137
fpr             0.284404
tf              0.015173
tpr             0.730769
Name: 35, dtype: float64

population: 187
P: 78
N: 109
PositiveTest: 88
NegativeTest: 99
TP: 57
TN: 78
FP: 31
FN: 21
TPR: 0.7307692307692307
TNR: 0.7155963302752294
PPV: 0.6477272727272727
NPV: 0.7878787878787878
FPR: 0.28440366972477066
FDR: 0.3522727272727273
FNR: 0.2692307692307692
ACC: 0.7219251336898396
F1_score: 0.6867469879518072
MCC: 0.4409529948155374
informedness: 0.4463655610444601
markedness: 0.43560606060606055
prevalence: 0.41711229946524064
LRP: 2.5694789081885854
LRN: 0.376232741617357
DOR: 6.829493087557603
FOR: 0.21212121212121213
None

그룹 4

1-fpr           0.816514
cutoff value    0.468386
fpr             0.183486
tf             -0.037293
tpr             0.779221
Name: 27, dtype: float64

population: 186
P: 77
N: 109
PositiveTest: 80
NegativeTest: 106
TP: 60
TN: 89
FP: 20
FN: 17
TPR: 0.7792207792207793
TNR: 0.8165137614678899
PPV: 0.75
NPV: 0.839622641509434
FPR: 0.1834862385321101
FDR: 0.25
FNR: 0.22077922077922077
ACC: 0.8010752688172043
F1_score: 0.7643312101910829
MCC: 0.592670712553997
informedness: 0.5957345406886692
markedness: 0.5896226415094339
prevalence: 0.41397849462365593
LRP: 4.246753246753246
LRN: 0.2703925288194951
DOR: 15.705882352941176
FOR: 0.16037735849056603
None

그룹 5

1-fpr           0.731481
cutoff value    0.438591
fpr             0.268519
tf             -0.017196
tpr             0.714286
Name: 37, dtype: float64

population: 185
P: 77
N: 108
PositiveTest: 84
NegativeTest: 101
TP: 55
TN: 79
FP: 29
FN: 22
TPR: 0.7142857142857143
TNR: 0.7314814814814815
PPV: 0.6547619047619048
NPV: 0.7821782178217822
FPR: 0.26851851851851855
FDR: 0.34523809523809523
FNR: 0.2857142857142857
ACC: 0.7243243243243244
F1_score: 0.6832298136645962
MCC: 0.44133159094076296
informedness: 0.4457671957671958
markedness: 0.4369401225836871
prevalence: 0.41621621621621624
LRP: 2.6600985221674875
LRN: 0.3905967450271247
DOR: 6.810344827586207
FOR: 0.21782178217821782
None
```

```
평균적인 threshold, 0.43 (0.02) [0.4-0.47]
평균적인 auc, 0.81 (0.05) [0.75-0.88]
평균적인 sensitivity, 0.72 (0.04) [0.65-0.77]
평균적인 specificity, 0.72 (0.05) [0.66-0.81]
평균적인 ppv, 0.65 (0.06) [0.57-0.74]
평균적인 npv, 0.78 (0.04) [0.72-0.83]
평균적인 accuracy, 0.72 (0.05) [0.65-0.79]
----------------------------------------------------------------------------------
AdaBoostClassifier(algorithm='SAMME.R', base_estimator=None,
          learning_rate=1.0, n_estimators=50, random_state=None)
그룹 1

1-fpr           0.688073
cutoff value    0.497806
fpr             0.311927
tf             -0.008586
tpr             0.679487
Name: 47, dtype: float64

population: 187
P: 78
N: 109
PositiveTest: 87
NegativeTest: 100
TP: 53
TN: 75
FP: 34
FN: 25
TPR: 0.6794871794871795
TNR: 0.6880733944954128
PPV: 0.6091954022988506
NPV: 0.75
FPR: 0.3119266055045872
FDR: 0.39080459770114945
FNR: 0.32051282051282054
ACC: 0.6844919786096256
F1_score: 0.6424242424242425
MCC: 0.36335391595643174
informedness: 0.36756057398259223
markedness: 0.3591954022988506
prevalence: 0.41711229946524064
LRP: 2.1783559577677223
LRN: 0.4658119658119659
DOR: 4.6764705882352935
FOR: 0.25
None

그룹 2

1-fpr           0.633028
cutoff value    0.497441
fpr             0.366972
tf              0.007998
tpr             0.641026
Name: 43, dtype: float64

population: 187
P: 78
N: 109
PositiveTest: 90
NegativeTest: 97
TP: 50
TN: 69
FP: 40
FN: 28
TPR: 0.6410256410256411
TNR: 0.6330275229357798
PPV: 0.5555555555555556
NPV: 0.711340206185567
FPR: 0.3669724770642202
FDR: 0.4444444444444444
FNR: 0.358974358974359
ACC: 0.6363636363636364
F1_score: 0.5952380952380952
MCC: 0.27045078656392957
informedness: 0.27405316396142076
markedness: 0.2668957617411225
prevalence: 0.41711229946524064
LRP: 1.7467948717948718
LRN: 0.5670754366406541
DOR: 3.080357142857143
FOR: 0.28865979381443296
None

그룹 3

1-fpr           0.688073
cutoff value    0.497132
fpr             0.311927
tf              0.029875
tpr             0.717949
Name: 37, dtype: float64

population: 187
P: 78
N: 109
PositiveTest: 90
NegativeTest: 97
TP: 56
TN: 75
FP: 34
FN: 22
TPR: 0.717948717948718
TNR: 0.6880733944954128
PPV: 0.6222222222222222
NPV: 0.7731958762886598
FPR: 0.3119266055045872
FDR: 0.37777777777777777
FNR: 0.28205128205128205
ACC: 0.7005347593582888
F1_score: 0.6666666666666666
MCC: 0.40068502799085187
informedness: 0.4060221124441308
markedness: 0.39541809851088194
prevalence: 0.41711229946524064
LRP: 2.3016591251885368
LRN: 0.4099145299145299
DOR: 5.614973262032085
FOR: 0.2268041237113402
None

그룹 4

1-fpr           0.761468
cutoff value    0.500124
fpr             0.238532
tf             -0.021208
tpr             0.740260
Name: 37, dtype: float64

population: 186
P: 77
N: 109
PositiveTest: 83
NegativeTest: 103
TP: 57
TN: 83
FP: 26
FN: 20
TPR: 0.7402597402597403
TNR: 0.7614678899082569
PPV: 0.6867469879518072
NPV: 0.8058252427184466
FPR: 0.23853211009174313
FDR: 0.3132530120481928
FNR: 0.2597402597402597
ACC: 0.7526881720430108
F1_score: 0.7125
MCC: 0.497128854504293
informedness: 0.5017276301679972
markedness: 0.4925722306702538
prevalence: 0.41397849462365593
LRP: 3.1033966033966034
LRN: 0.341104678454076
DOR: 9.098076923076924
FOR: 0.1941747572815534
None

그룹 5

1-fpr           0.722222
cutoff value    0.497213
fpr             0.277778
tf             -0.007937
tpr             0.714286
Name: 39, dtype: float64

population: 185
P: 77
N: 108
PositiveTest: 85
NegativeTest: 100
TP: 55
TN: 78
FP: 30
FN: 22
TPR: 0.7142857142857143
TNR: 0.7222222222222222
PPV: 0.6470588235294118
NPV: 0.78
FPR: 0.2777777777777778
FDR: 0.35294117647058826
FNR: 0.2857142857142857
ACC: 0.7189189189189189
F1_score: 0.6790123456790124
MCC: 0.4317575312908051
informedness: 0.4365079365079365
markedness: 0.42705882352941194
prevalence: 0.41621621621621624
LRP: 2.571428571428571
LRN: 0.3956043956043956
DOR: 6.5
FOR: 0.22
None
```

```
평균적인 threshold, 0.5 (0.0) [0.5-0.5]
평균적인 auc, 0.75 (0.05) [0.67-0.82]
평균적인 sensitivity, 0.7 (0.03) [0.64-0.74]
평균적인 specificity, 0.7 (0.04) [0.64-0.76]
평균적인 ppv, 0.62 (0.04) [0.56-0.68]
평균적인 npv, 0.76 (0.03) [0.72-0.8]
평균적인 accuracy, 0.7 (0.04) [0.64-0.75]
----------------------------------------------------------------------------------
<catboost.core.CatBoostClassifier object at 0x0000021620E03D68>
그룹 1

1-fpr           0.715596
cutoff value    0.399768
fpr             0.284404
tf             -0.010468
tpr             0.705128
Name: 36, dtype: float64

population: 187
P: 78
N: 109
PositiveTest: 86
NegativeTest: 101
TP: 55
TN: 78
FP: 31
FN: 23
TPR: 0.7051282051282052
TNR: 0.7155963302752294
PPV: 0.6395348837209303
NPV: 0.7722772277227723
FPR: 0.28440366972477066
FDR: 0.36046511627906974
FNR: 0.2948717948717949
ACC: 0.7112299465240641
F1_score: 0.6707317073170732
MCC: 0.4162444705466478
informedness: 0.4207245354034346
markedness: 0.41181211144370256
prevalence: 0.41711229946524064
LRP: 2.479321753515302
LRN: 0.41206443129520054
DOR: 6.016830294530154
FOR: 0.22772277227722773
None

그룹 2

1-fpr           0.642202
cutoff value    0.268862
fpr             0.357798
tf              0.024465
tpr             0.666667
Name: 43, dtype: float64

population: 187
P: 78
N: 109
PositiveTest: 91
NegativeTest: 96
TP: 52
TN: 70
FP: 39
FN: 26
TPR: 0.6666666666666666
TNR: 0.6422018348623854
PPV: 0.5714285714285714
NPV: 0.7291666666666666
FPR: 0.3577981651376147
FDR: 0.42857142857142855
FNR: 0.3333333333333333
ACC: 0.6524064171122995
F1_score: 0.6153846153846154
MCC: 0.3047037918327319
informedness: 0.308868501529052
markedness: 0.30059523809523814
prevalence: 0.41711229946524064
LRP: 1.863247863247863
LRN: 0.519047619047619
DOR: 3.58974358974359
FOR: 0.2708333333333333
None

그룹 3

1-fpr           0.752294
cutoff value    0.325852
fpr             0.247706
tf             -0.034345
tpr             0.717949
Name: 32, dtype: float64

population: 187
P: 78
N: 109
PositiveTest: 83
NegativeTest: 104
TP: 56
TN: 82
FP: 27
FN: 22
TPR: 0.717948717948718
TNR: 0.7522935779816514
PPV: 0.6746987951807228
NPV: 0.7884615384615384
FPR: 0.24770642201834864
FDR: 0.3253012048192771
FNR: 0.28205128205128205
ACC: 0.7379679144385026
F1_score: 0.6956521739130435
MCC: 0.4666878814323475
informedness: 0.47024229593036937
markedness: 0.46316033364226117
prevalence: 0.41711229946524064
LRP: 2.8983855650522314
LRN: 0.3749218261413383
DOR: 7.730639730639731
FOR: 0.21153846153846154
None

그룹 4

1-fpr           0.779817
cutoff value    0.458200
fpr             0.220183
tf             -0.013583
tpr             0.766234
Name: 31, dtype: float64

population: 186
P: 77
N: 109
PositiveTest: 83
NegativeTest: 103
TP: 59
TN: 85
FP: 24
FN: 18
TPR: 0.7662337662337663
TNR: 0.7798165137614679
PPV: 0.7108433734939759
NPV: 0.8252427184466019
FPR: 0.22018348623853212
FDR: 0.2891566265060241
FNR: 0.23376623376623376
ACC: 0.7741935483870968
F1_score: 0.7375
MCC: 0.5410452482054559
informedness: 0.5460502799952343
markedness: 0.5360860919405779
prevalence: 0.41397849462365593
LRP: 3.4799783549783547
LRN: 0.29977081741787626
DOR: 11.608796296296294
FOR: 0.17475728155339806
None

그룹 5

1-fpr           0.694444
cutoff value    0.373459
fpr             0.305556
tf             -0.006133
tpr             0.688312
Name: 47, dtype: float64

population: 185
P: 77
N: 108
PositiveTest: 86
NegativeTest: 99
TP: 53
TN: 75
FP: 33
FN: 24
TPR: 0.6883116883116883
TNR: 0.6944444444444444
PPV: 0.6162790697674418
NPV: 0.7575757575757576
FPR: 0.3055555555555556
FDR: 0.38372093023255816
FNR: 0.3116883116883117
ACC: 0.6918918918918919
F1_score: 0.6503067484662577
MCC: 0.3782792988336723
informedness: 0.38275613275613285
markedness: 0.3738548273431994
prevalence: 0.41621621621621624
LRP: 2.2526564344746163
LRN: 0.44883116883116886
DOR: 5.018939393939394
FOR: 0.24242424242424243
None
```

```
평균적인 threshold, 0.37 (0.06) [0.27-0.45]
평균적인 auc, 0.78 (0.05) [0.73-0.86]
평균적인 sensitivity, 0.71 (0.03) [0.67-0.76]
평균적인 specificity, 0.72 (0.05) [0.65-0.78]
평균적인 ppv, 0.64 (0.05) [0.58-0.71]
평균적인 npv, 0.77 (0.03) [0.73-0.82]
평균적인 accuracy, 0.71 (0.04) [0.66-0.77]
----------------------------------------------------------------------------------
```

# auc maximize, 200 times bootstrap¶

In [20]:

```
from sklearn.model_selection import train_test_split
from tqdm import tqdm
```

## training set ci¶

In [21]:

```
for clf in classifiers:
    t = 1
    print(str(clf))
    ci = {'accuracy':[],'sensitivity':[],'specificity':[],'PPV':[],'NPV':[],'auc':[],'threshold':[]}
    for t in tqdm(range(200)):
        X_train, X_test, y_train, y_test = train_test_split(train_features.values, target.values,stratify = target.values)
        clf.fit(X_train, y_train)
        pred_score = clf.predict_proba(X_test)
        fpr, tpr, thresholds = metrics.roc_curve(y_test, pred_score[:,1])
        i = np.arange(len(tpr))
        roc = pd.DataFrame({'fpr' : pd.Series(fpr, index=i),'tpr' : pd.Series(tpr, index = i), '1-fpr' : pd.Series(1-fpr, index = i), 'tf' : pd.Series(tpr - (1-fpr), index = i), 'cutoff value' : pd.Series(thresholds, index = i)})
        threshold = roc.iloc[(roc.tf-0).abs().argsort()[1]]['cutoff value']
        ci['threshold'].append(threshold)
        roc_auc = metrics.auc(fpr, tpr)
        pred = np.where(pred_score>=threshold,1,0)
        ci['auc'].append(roc_auc)
        stats = pdml.ConfusionMatrix(y_test,pred[:,1]).stats()
        ci['sensitivity'].append(stats['TPR'])
        ci['specificity'].append(stats['TNR'])
        ci['PPV'].append(stats['PPV'])
        ci['NPV'].append(stats['NPV'])
        ci['accuracy'].append(stats['ACC'])
    print("평균적인 threshold, {} ({}) [{}-{}]".format(np.mean(ci['threshold']).round(2), np.std(ci['threshold']).round(2), 
          np.percentile(ci['threshold'],2.5).round(2), np.percentile(ci['threshold'],97.5).round(2)))
    print("평균적인 auc, {} ({}) [{}-{}]".format(np.mean(ci['auc']).round(2), np.std(ci['auc']).round(2), 
          np.percentile(ci['auc'],2.5).round(2), np.percentile(ci['auc'],97.5).round(2)))
    print("평균적인 sensitivity, {} ({}) [{}-{}]".format(np.mean(ci['sensitivity']).round(2), np.std(ci['sensitivity']).round(2), 
          np.percentile(ci['sensitivity'],2.5).round(2), np.percentile(ci['sensitivity'],97.5).round(2)))
    print("평균적인 specificity, {} ({}) [{}-{}]".format(np.mean(ci['specificity']).round(2), np.std(ci['specificity']).round(2), 
          np.percentile(ci['specificity'],2.5).round(2), np.percentile(ci['specificity'],97.5).round(2)))
    print("평균적인 ppv, {} ({}) [{}-{}]".format(np.mean(ci['PPV']).round(2), np.std(ci['PPV']).round(2), 
          np.percentile(ci['PPV'],2.5).round(2), np.percentile(ci['PPV'],97.5).round(2)))
    print("평균적인 npv, {} ({}) [{}-{}]".format(np.mean(ci['NPV']).round(2), np.std(ci['NPV']).round(2), 
          np.percentile(ci['NPV'],2.5).round(2), np.percentile(ci['NPV'],97.5).round(2)))
    print("평균적인 accuracy, {} ({}) [{}-{}]".format(np.mean(ci['accuracy']).round(2), np.std(ci['accuracy']).round(2), 
          np.percentile(ci['accuracy'],2.5).round(2), np.percentile(ci['accuracy'],97.5).round(2)))
    print('----------------------------------------------------------------------------------')
```

```
LogisticRegressionCV(Cs=10, class_weight=None, cv=None, dual=False,
           fit_intercept=True, intercept_scaling=1.0, max_iter=100,
           multi_class='ovr', n_jobs=-1, penalty='l2', random_state=None,
           refit=False, scoring=None, solver='lbfgs', tol=0.0001,
           verbose=0)
```

```
100%|████████████| 200/200 [21:02<00:00,  5.89s/it]
```

```
평균적인 threshold, 0.42 (0.03) [0.37-0.48]
평균적인 auc, 0.81 (0.03) [0.76-0.86]
평균적인 sensitivity, 0.74 (0.03) [0.69-0.78]
평균적인 specificity, 0.74 (0.03) [0.68-0.79]
평균적인 ppv, 0.67 (0.03) [0.61-0.72]
평균적인 npv, 0.8 (0.02) [0.76-0.84]
평균적인 accuracy, 0.74 (0.03) [0.69-0.79]
----------------------------------------------------------------------------------
SVC(C=0.025, cache_size=200, class_weight=None, coef0=0.0,
  decision_function_shape='ovr', degree=3, gamma='auto', kernel='linear',
  max_iter=-1, probability=True, random_state=None, shrinking=True,
  tol=0.001, verbose=False)
```

```
100%|████████████| 200/200 [00:31<00:00,  6.23it/s]
```

```
평균적인 threshold, 0.42 (0.02) [0.37-0.46]
평균적인 auc, 0.8 (0.02) [0.76-0.85]
평균적인 sensitivity, 0.73 (0.03) [0.68-0.78]
평균적인 specificity, 0.73 (0.03) [0.68-0.79]
평균적인 ppv, 0.66 (0.03) [0.61-0.73]
평균적인 npv, 0.79 (0.02) [0.75-0.84]
평균적인 accuracy, 0.73 (0.03) [0.69-0.79]
----------------------------------------------------------------------------------
RandomForestClassifier(bootstrap=True, class_weight=None, criterion='gini',
            max_depth=5, max_features='auto', max_leaf_nodes=None,
            min_impurity_decrease=0.0, min_impurity_split=None,
            min_samples_leaf=1, min_samples_split=2,
            min_weight_fraction_leaf=0.0, n_estimators=500, n_jobs=-1,
            oob_score=False, random_state=4321, verbose=0,
            warm_start=False)
```

```
100%|████████████| 200/200 [02:59<00:00,  1.12s/it]
```

```
평균적인 threshold, 0.43 (0.02) [0.4-0.47]
평균적인 auc, 0.81 (0.02) [0.76-0.85]
평균적인 sensitivity, 0.73 (0.03) [0.67-0.8]
평균적인 specificity, 0.73 (0.03) [0.67-0.78]
평균적인 ppv, 0.66 (0.03) [0.59-0.72]
평균적인 npv, 0.79 (0.02) [0.74-0.84]
평균적인 accuracy, 0.73 (0.03) [0.67-0.79]
----------------------------------------------------------------------------------
AdaBoostClassifier(algorithm='SAMME.R', base_estimator=None,
          learning_rate=1.0, n_estimators=50, random_state=None)
```

```
100%|████████████| 200/200 [00:34<00:00,  6.31it/s]
```

```
평균적인 threshold, 0.5 (0.0) [0.5-0.5]
평균적인 auc, 0.75 (0.03) [0.7-0.8]
평균적인 sensitivity, 0.69 (0.03) [0.64-0.74]
평균적인 specificity, 0.69 (0.03) [0.64-0.74]
평균적인 ppv, 0.62 (0.03) [0.56-0.67]
평균적인 npv, 0.76 (0.02) [0.72-0.8]
평균적인 accuracy, 0.69 (0.03) [0.64-0.74]
----------------------------------------------------------------------------------
<catboost.core.CatBoostClassifier object at 0x0000021620E03D68>
```

```
100%|██████████| 200/200 [1:47:39<00:00, 36.37s/it]
```

```
평균적인 threshold, 0.38 (0.06) [0.28-0.49]
평균적인 auc, 0.78 (0.03) [0.73-0.83]
평균적인 sensitivity, 0.71 (0.03) [0.66-0.76]
평균적인 specificity, 0.71 (0.03) [0.65-0.76]
평균적인 ppv, 0.64 (0.03) [0.58-0.69]
평균적인 npv, 0.78 (0.02) [0.74-0.82]
평균적인 accuracy, 0.71 (0.03) [0.67-0.76]
----------------------------------------------------------------------------------
```

# test set ci¶

In [22]:

```
for clf in classifiers:
    t = 1
    print(str(clf))
    ci = {'accuracy':[],'sensitivity':[],'specificity':[],'PPV':[],'NPV':[],'auc':[],'threshold':[]}
    for t in tqdm(range(200)):
        X_train, X_test, y_train, y_test = train_test_split(test_features.values, test_target.values,stratify = test_target.values)
        clf.fit(X_train, y_train)
        pred_score = clf.predict_proba(X_test)
        fpr, tpr, thresholds = metrics.roc_curve(y_test, pred_score[:,1])
        i = np.arange(len(tpr))
        roc = pd.DataFrame({'fpr' : pd.Series(fpr, index=i),'tpr' : pd.Series(tpr, index = i), '1-fpr' : pd.Series(1-fpr, index = i), 'tf' : pd.Series(tpr - (1-fpr), index = i), 'cutoff value' : pd.Series(thresholds, index = i)})
        threshold = roc.iloc[(roc.tf-0).abs().argsort()[1]]['cutoff value']
        ci['threshold'].append(threshold)
        roc_auc = metrics.auc(fpr, tpr)
        pred = np.where(pred_score>=threshold,1,0)
        ci['auc'].append(roc_auc)
        stats = pdml.ConfusionMatrix(y_test,pred[:,1]).stats()
        ci['sensitivity'].append(stats['TPR'])
        ci['specificity'].append(stats['TNR'])
        ci['PPV'].append(stats['PPV'])
        ci['NPV'].append(stats['NPV'])
        ci['accuracy'].append(stats['ACC'])
    print("평균적인 threshold, {} ({}) [{}-{}]".format(np.mean(ci['threshold']).round(2), np.std(ci['threshold']).round(2), 
          np.percentile(ci['threshold'],2.5).round(2), np.percentile(ci['threshold'],97.5).round(2)))
    print("평균적인 auc, {} ({}) [{}-{}]".format(np.mean(ci['auc']).round(2), np.std(ci['auc']).round(2), 
          np.percentile(ci['auc'],2.5).round(2), np.percentile(ci['auc'],97.5).round(2)))
    print("평균적인 sensitivity, {} ({}) [{}-{}]".format(np.mean(ci['sensitivity']).round(2), np.std(ci['sensitivity']).round(2), 
          np.percentile(ci['sensitivity'],2.5).round(2), np.percentile(ci['sensitivity'],97.5).round(2)))
    print("평균적인 specificity, {} ({}) [{}-{}]".format(np.mean(ci['specificity']).round(2), np.std(ci['specificity']).round(2), 
          np.percentile(ci['specificity'],2.5).round(2), np.percentile(ci['specificity'],97.5).round(2)))
    print("평균적인 ppv, {} ({}) [{}-{}]".format(np.mean(ci['PPV']).round(2), np.std(ci['PPV']).round(2), 
          np.percentile(ci['PPV'],2.5).round(2), np.percentile(ci['PPV'],97.5).round(2)))
    print("평균적인 npv, {} ({}) [{}-{}]".format(np.mean(ci['NPV']).round(2), np.std(ci['NPV']).round(2), 
          np.percentile(ci['NPV'],2.5).round(2), np.percentile(ci['NPV'],97.5).round(2)))
    print("평균적인 accuracy, {} ({}) [{}-{}]".format(np.mean(ci['accuracy']).round(2), np.std(ci['accuracy']).round(2), 
          np.percentile(ci['accuracy'],2.5).round(2), np.percentile(ci['accuracy'],97.5).round(2)))
    print('----------------------------------------------------------------------------------')
```

```
LogisticRegressionCV(Cs=10, class_weight=None, cv=None, dual=False,
           fit_intercept=True, intercept_scaling=1.0, max_iter=100,
           multi_class='ovr', n_jobs=-1, penalty='l2', random_state=None,
           refit=False, scoring=None, solver='lbfgs', tol=0.0001,
           verbose=0)
```

```
  0%|                      | 0/200 [00:00<?, ?it/s]C:\Anaconda3\lib\site-packages\ipykernel\__main__.py:12: DeprecationWarning: 
.ix is deprecated. Please use
.loc for label based indexing or
.iloc for positional indexing

See the documentation here:
http://pandas.pydata.org/pandas-docs/stable/indexing.html#ix-indexer-is-deprecated
100%|████████████| 200/200 [20:01<00:00,  5.84s/it]
```

```
평균적인 threshold, 0.47 (0.1) [0.27-0.65]
평균적인 auc, 0.83 (0.06) [0.71-0.94]
평균적인 sensitivity, 0.75 (0.07) [0.65-0.87]
평균적인 specificity, 0.76 (0.07) [0.63-0.89]
평균적인 ppv, 0.73 (0.07) [0.61-0.86]
평균적인 npv, 0.78 (0.05) [0.68-0.88]
평균적인 accuracy, 0.76 (0.06) [0.66-0.86]
----------------------------------------------------------------------------------
SVC(C=0.025, cache_size=200, class_weight=None, coef0=0.0,
  decision_function_shape='ovr', degree=3, gamma='auto', kernel='linear',
  max_iter=-1, probability=True, random_state=None, shrinking=True,
  tol=0.001, verbose=False)
```

```
  0%|                      | 0/200 [00:00<?, ?it/s]C:\Anaconda3\lib\site-packages\ipykernel\__main__.py:12: DeprecationWarning: 
.ix is deprecated. Please use
.loc for label based indexing or
.iloc for positional indexing

See the documentation here:
http://pandas.pydata.org/pandas-docs/stable/indexing.html#ix-indexer-is-deprecated
100%|████████████| 200/200 [00:06<00:00, 29.30it/s]
```

```
평균적인 threshold, 0.47 (0.08) [0.34-0.61]
평균적인 auc, 0.85 (0.05) [0.76-0.94]
평균적인 sensitivity, 0.78 (0.07) [0.65-0.87]
평균적인 specificity, 0.78 (0.07) [0.67-0.93]
평균적인 ppv, 0.76 (0.07) [0.63-0.9]
평균적인 npv, 0.81 (0.05) [0.7-0.9]
평균적인 accuracy, 0.78 (0.05) [0.68-0.88]
----------------------------------------------------------------------------------
RandomForestClassifier(bootstrap=True, class_weight=None, criterion='gini',
            max_depth=5, max_features='auto', max_leaf_nodes=None,
            min_impurity_decrease=0.0, min_impurity_split=None,
            min_samples_leaf=1, min_samples_split=2,
            min_weight_fraction_leaf=0.0, n_estimators=500, n_jobs=-1,
            oob_score=False, random_state=4321, verbose=0,
            warm_start=False)
```

```
  0%|                      | 0/200 [00:00<?, ?it/s]C:\Anaconda3\lib\site-packages\ipykernel\__main__.py:12: DeprecationWarning: 
.ix is deprecated. Please use
.loc for label based indexing or
.iloc for positional indexing

See the documentation here:
http://pandas.pydata.org/pandas-docs/stable/indexing.html#ix-indexer-is-deprecated
  0%|              | 1/200 [00:00<02:37,  1.27it/s]C:\Anaconda3\lib\site-packages\ipykernel\__main__.py:12: DeprecationWarning: 
.ix is deprecated. Please use
.loc for label based indexing or
.iloc for positional indexing

See the documentation here:
http://pandas.pydata.org/pandas-docs/stable/indexing.html#ix-indexer-is-deprecated
  1%|▏             | 2/200 [00:01<02:40,  1.23it/s]C:\Anaconda3\lib\site-packages\ipykernel\__main__.py:12: DeprecationWarning: 
.ix is deprecated. Please use
.loc for label based indexing or
.iloc for positional indexing

See the documentation here:
http://pandas.pydata.org/pandas-docs/stable/indexing.html#ix-indexer-is-deprecated
  2%|▏             | 3/200 [00:02<02:40,  1.23it/s]C:\Anaconda3\lib\site-packages\ipykernel\__main__.py:12: DeprecationWarning: 
.ix is deprecated. Please use
.loc for label based indexing or
.iloc for positional indexing

See the documentation here:
http://pandas.pydata.org/pandas-docs/stable/indexing.html#ix-indexer-is-deprecated
  2%|▎             | 4/200 [00:03<02:41,  1.22it/s]C:\Anaconda3\lib\site-packages\ipykernel\__main__.py:12: DeprecationWarning: 
.ix is deprecated. Please use
.loc for label based indexing or
.iloc for positional indexing

See the documentation here:
http://pandas.pydata.org/pandas-docs/stable/indexing.html#ix-indexer-is-deprecated
  2%|▎             | 5/200 [00:04<02:38,  1.23it/s]C:\Anaconda3\lib\site-packages\ipykernel\__main__.py:12: DeprecationWarning: 
.ix is deprecated. Please use
.loc for label based indexing or
.iloc for positional indexing

See the documentation here:
http://pandas.pydata.org/pandas-docs/stable/indexing.html#ix-indexer-is-deprecated
  3%|▍             | 6/200 [00:04<02:36,  1.24it/s]C:\Anaconda3\lib\site-packages\ipykernel\__main__.py:12: DeprecationWarning: 
.ix is deprecated. Please use
.loc for label based indexing or
.iloc for positional indexing

See the documentation here:
http://pandas.pydata.org/pandas-docs/stable/indexing.html#ix-indexer-is-deprecated
  4%|▍             | 7/200 [00:05<02:35,  1.24it/s]C:\Anaconda3\lib\site-packages\ipykernel\__main__.py:12: DeprecationWarning: 
.ix is deprecated. Please use
.loc for label based indexing or
.iloc for positional indexing

See the documentation here:
http://pandas.pydata.org/pandas-docs/stable/indexing.html#ix-indexer-is-deprecated
  4%|▌             | 8/200 [00:06<02:34,  1.25it/s]C:\Anaconda3\lib\site-packages\ipykernel\__main__.py:12: DeprecationWarning: 
.ix is deprecated. Please use
.loc for label based indexing or
.iloc for positional indexing

See the documentation here:
http://pandas.pydata.org/pandas-docs/stable/indexing.html#ix-indexer-is-deprecated
  4%|▋             | 9/200 [00:07<02:32,  1.25it/s]C:\Anaconda3\lib\site-packages\ipykernel\__main__.py:12: DeprecationWarning: 
.ix is deprecated. Please use
.loc for label based indexing or
.iloc for positional indexing

See the documentation here:
http://pandas.pydata.org/pandas-docs/stable/indexing.html#ix-indexer-is-deprecated
  5%|▋            | 10/200 [00:08<02:31,  1.25it/s]C:\Anaconda3\lib\site-packages\ipykernel\__main__.py:12: DeprecationWarning: 
.ix is deprecated. Please use
.loc for label based indexing or
.iloc for positional indexing

See the documentation here:
http://pandas.pydata.org/pandas-docs/stable/indexing.html#ix-indexer-is-deprecated
  6%|▋            | 11/200 [00:08<02:33,  1.23it/s]C:\Anaconda3\lib\site-packages\ipykernel\__main__.py:12: DeprecationWarning: 
.ix is deprecated. Please use
.loc for label based indexing or
.iloc for positional indexing

See the documentation here:
http://pandas.pydata.org/pandas-docs/stable/indexing.html#ix-indexer-is-deprecated
  6%|▊            | 12/200 [00:09<02:34,  1.21it/s]C:\Anaconda3\lib\site-packages\ipykernel\__main__.py:12: DeprecationWarning: 
.ix is deprecated. Please use
.loc for label based indexing or
.iloc for positional indexing

See the documentation here:
http://pandas.pydata.org/pandas-docs/stable/indexing.html#ix-indexer-is-deprecated
  6%|▊            | 13/200 [00:10<02:35,  1.21it/s]C:\Anaconda3\lib\site-packages\ipykernel\__main__.py:12: DeprecationWarning: 
.ix is deprecated. Please use
.loc for label based indexing or
.iloc for positional indexing

See the documentation here:
http://pandas.pydata.org/pandas-docs/stable/indexing.html#ix-indexer-is-deprecated
  7%|▉            | 14/200 [00:11<02:32,  1.22it/s]C:\Anaconda3\lib\site-packages\ipykernel\__main__.py:12: DeprecationWarning: 
.ix is deprecated. Please use
.loc for label based indexing or
.iloc for positional indexing

See the documentation here:
http://pandas.pydata.org/pandas-docs/stable/indexing.html#ix-indexer-is-deprecated
  8%|▉            | 15/200 [00:12<02:31,  1.22it/s]C:\Anaconda3\lib\site-packages\ipykernel\__main__.py:12: DeprecationWarning: 
.ix is deprecated. Please use
.loc for label based indexing or
.iloc for positional indexing

See the documentation here:
http://pandas.pydata.org/pandas-docs/stable/indexing.html#ix-indexer-is-deprecated
  8%|█            | 16/200 [00:13<02:31,  1.21it/s]C:\Anaconda3\lib\site-packages\ipykernel\__main__.py:12: DeprecationWarning: 
.ix is deprecated. Please use
.loc for label based indexing or
.iloc for positional indexing

See the documentation here:
http://pandas.pydata.org/pandas-docs/stable/indexing.html#ix-indexer-is-deprecated
  8%|█            | 17/200 [00:13<02:28,  1.23it/s]C:\Anaconda3\lib\site-packages\ipykernel\__main__.py:12: DeprecationWarning: 
.ix is deprecated. Please use
.loc for label based indexing or
.iloc for positional indexing

See the documentation here:
http://pandas.pydata.org/pandas-docs/stable/indexing.html#ix-indexer-is-deprecated
  9%|█▏           | 18/200 [00:14<02:26,  1.24it/s]C:\Anaconda3\lib\site-packages\ipykernel\__main__.py:12: DeprecationWarning: 
.ix is deprecated. Please use
.loc for label based indexing or
.iloc for positional indexing

See the documentation here:
http://pandas.pydata.org/pandas-docs/stable/indexing.html#ix-indexer-is-deprecated
 10%|█▏           | 19/200 [00:15<02:26,  1.24it/s]C:\Anaconda3\lib\site-packages\ipykernel\__main__.py:12: DeprecationWarning: 
.ix is deprecated. Please use
.loc for label based indexing or
.iloc for positional indexing

See the documentation here:
http://pandas.pydata.org/pandas-docs/stable/indexing.html#ix-indexer-is-deprecated
 10%|█▎           | 20/200 [00:16<02:25,  1.24it/s]C:\Anaconda3\lib\site-packages\ipykernel\__main__.py:12: DeprecationWarning: 
.ix is deprecated. Please use
.loc for label based indexing or
.iloc for positional indexing

See the documentation here:
http://pandas.pydata.org/pandas-docs/stable/indexing.html#ix-indexer-is-deprecated
 10%|█▎           | 21/200 [00:17<02:23,  1.25it/s]C:\Anaconda3\lib\site-packages\ipykernel\__main__.py:12: DeprecationWarning: 
.ix is deprecated. Please use
.loc for label based indexing or
.iloc for positional indexing

See the documentation here:
http://pandas.pydata.org/pandas-docs/stable/indexing.html#ix-indexer-is-deprecated
 11%|█▍           | 22/200 [00:17<02:21,  1.26it/s]C:\Anaconda3\lib\site-packages\ipykernel\__main__.py:12: DeprecationWarning: 
.ix is deprecated. Please use
.loc for label based indexing or
.iloc for positional indexing

See the documentation here:
http://pandas.pydata.org/pandas-docs/stable/indexing.html#ix-indexer-is-deprecated
 12%|█▍           | 23/200 [00:18<02:20,  1.26it/s]C:\Anaconda3\lib\site-packages\ipykernel\__main__.py:12: DeprecationWarning: 
.ix is deprecated. Please use
.loc for label based indexing or
.iloc for positional indexing

See the documentation here:
http://pandas.pydata.org/pandas-docs/stable/indexing.html#ix-indexer-is-deprecated
 12%|█▌           | 24/200 [00:19<02:19,  1.26it/s]C:\Anaconda3\lib\site-packages\ipykernel\__main__.py:12: DeprecationWarning: 
.ix is deprecated. Please use
.loc for label based indexing or
.iloc for positional indexing

See the documentation here:
http://pandas.pydata.org/pandas-docs/stable/indexing.html#ix-indexer-is-deprecated
 12%|█▋           | 25/200 [00:20<02:18,  1.26it/s]C:\Anaconda3\lib\site-packages\ipykernel\__main__.py:12: DeprecationWarning: 
.ix is deprecated. Please use
.loc for label based indexing or
.iloc for positional indexing

See the documentation here:
http://pandas.pydata.org/pandas-docs/stable/indexing.html#ix-indexer-is-deprecated
 13%|█▋           | 26/200 [00:20<02:17,  1.27it/s]C:\Anaconda3\lib\site-packages\ipykernel\__main__.py:12: DeprecationWarning: 
.ix is deprecated. Please use
.loc for label based indexing or
.iloc for positional indexing

See the documentation here:
http://pandas.pydata.org/pandas-docs/stable/indexing.html#ix-indexer-is-deprecated
 14%|█▊           | 27/200 [00:21<02:15,  1.28it/s]C:\Anaconda3\lib\site-packages\ipykernel\__main__.py:12: DeprecationWarning: 
.ix is deprecated. Please use
.loc for label based indexing or
.iloc for positional indexing

See the documentation here:
http://pandas.pydata.org/pandas-docs/stable/indexing.html#ix-indexer-is-deprecated
 14%|█▊           | 28/200 [00:22<02:16,  1.26it/s]C:\Anaconda3\lib\site-packages\ipykernel\__main__.py:12: DeprecationWarning: 
.ix is deprecated. Please use
.loc for label based indexing or
.iloc for positional indexing

See the documentation here:
http://pandas.pydata.org/pandas-docs/stable/indexing.html#ix-indexer-is-deprecated
 14%|█▉           | 29/200 [00:23<02:16,  1.25it/s]C:\Anaconda3\lib\site-packages\ipykernel\__main__.py:12: DeprecationWarning: 
.ix is deprecated. Please use
.loc for label based indexing or
.iloc for positional indexing

See the documentation here:
http://pandas.pydata.org/pandas-docs/stable/indexing.html#ix-indexer-is-deprecated
 15%|█▉           | 30/200 [00:24<02:15,  1.26it/s]C:\Anaconda3\lib\site-packages\ipykernel\__main__.py:12: DeprecationWarning: 
.ix is deprecated. Please use
.loc for label based indexing or
.iloc for positional indexing

See the documentation here:
http://pandas.pydata.org/pandas-docs/stable/indexing.html#ix-indexer-is-deprecated
 16%|██           | 31/200 [00:24<02:14,  1.26it/s]C:\Anaconda3\lib\site-packages\ipykernel\__main__.py:12: DeprecationWarning: 
.ix is deprecated. Please use
.loc for label based indexing or
.iloc for positional indexing

See the documentation here:
http://pandas.pydata.org/pandas-docs/stable/indexing.html#ix-indexer-is-deprecated
 16%|██           | 32/200 [00:25<02:14,  1.25it/s]C:\Anaconda3\lib\site-packages\ipykernel\__main__.py:12: DeprecationWarning: 
.ix is deprecated. Please use
.loc for label based indexing or
.iloc for positional indexing

See the documentation here:
http://pandas.pydata.org/pandas-docs/stable/indexing.html#ix-indexer-is-deprecated
 16%|██▏          | 33/200 [00:26<02:14,  1.24it/s]C:\Anaconda3\lib\site-packages\ipykernel\__main__.py:12: DeprecationWarning: 
.ix is deprecated. Please use
.loc for label based indexing or
.iloc for positional indexing

See the documentation here:
http://pandas.pydata.org/pandas-docs/stable/indexing.html#ix-indexer-is-deprecated
 17%|██▏          | 34/200 [00:27<02:14,  1.23it/s]C:\Anaconda3\lib\site-packages\ipykernel\__main__.py:12: DeprecationWarning: 
.ix is deprecated. Please use
.loc for label based indexing or
.iloc for positional indexing

See the documentation here:
http://pandas.pydata.org/pandas-docs/stable/indexing.html#ix-indexer-is-deprecated
 18%|██▎          | 35/200 [00:28<02:13,  1.24it/s]C:\Anaconda3\lib\site-packages\ipykernel\__main__.py:12: DeprecationWarning: 
.ix is deprecated. Please use
.loc for label based indexing or
.iloc for positional indexing

See the documentation here:
http://pandas.pydata.org/pandas-docs/stable/indexing.html#ix-indexer-is-deprecated
 18%|██▎          | 36/200 [00:29<02:12,  1.24it/s]C:\Anaconda3\lib\site-packages\ipykernel\__main__.py:12: DeprecationWarning: 
.ix is deprecated. Please use
.loc for label based indexing or
.iloc for positional indexing

See the documentation here:
http://pandas.pydata.org/pandas-docs/stable/indexing.html#ix-indexer-is-deprecated
 18%|██▍          | 37/200 [00:29<02:12,  1.23it/s]C:\Anaconda3\lib\site-packages\ipykernel\__main__.py:12: DeprecationWarning: 
.ix is deprecated. Please use
.loc for label based indexing or
.iloc for positional indexing

See the documentation here:
http://pandas.pydata.org/pandas-docs/stable/indexing.html#ix-indexer-is-deprecated
 19%|██▍          | 38/200 [00:30<02:11,  1.23it/s]C:\Anaconda3\lib\site-packages\ipykernel\__main__.py:12: DeprecationWarning: 
.ix is deprecated. Please use
.loc for label based indexing or
.iloc for positional indexing

See the documentation here:
http://pandas.pydata.org/pandas-docs/stable/indexing.html#ix-indexer-is-deprecated
 20%|██▌          | 39/200 [00:31<02:10,  1.23it/s]C:\Anaconda3\lib\site-packages\ipykernel\__main__.py:12: DeprecationWarning: 
.ix is deprecated. Please use
.loc for label based indexing or
.iloc for positional indexing

See the documentation here:
http://pandas.pydata.org/pandas-docs/stable/indexing.html#ix-indexer-is-deprecated
 20%|██▌          | 40/200 [00:32<02:09,  1.24it/s]C:\Anaconda3\lib\site-packages\ipykernel\__main__.py:12: DeprecationWarning: 
.ix is deprecated. Please use
.loc for label based indexing or
.iloc for positional indexing

See the documentation here:
http://pandas.pydata.org/pandas-docs/stable/indexing.html#ix-indexer-is-deprecated
 20%|██▋          | 41/200 [00:33<02:07,  1.25it/s]C:\Anaconda3\lib\site-packages\ipykernel\__main__.py:12: DeprecationWarning: 
.ix is deprecated. Please use
.loc for label based indexing or
.iloc for positional indexing

See the documentation here:
http://pandas.pydata.org/pandas-docs/stable/indexing.html#ix-indexer-is-deprecated
 21%|██▋          | 42/200 [00:33<02:06,  1.25it/s]C:\Anaconda3\lib\site-packages\ipykernel\__main__.py:12: DeprecationWarning: 
.ix is deprecated. Please use
.loc for label based indexing or
.iloc for positional indexing

See the documentation here:
http://pandas.pydata.org/pandas-docs/stable/indexing.html#ix-indexer-is-deprecated
 22%|██▊          | 43/200 [00:34<02:06,  1.24it/s]C:\Anaconda3\lib\site-packages\ipykernel\__main__.py:12: DeprecationWarning: 
.ix is deprecated. Please use
.loc for label based indexing or
.iloc for positional indexing

See the documentation here:
http://pandas.pydata.org/pandas-docs/stable/indexing.html#ix-indexer-is-deprecated
 22%|██▊          | 44/200 [00:35<02:06,  1.24it/s]C:\Anaconda3\lib\site-packages\ipykernel\__main__.py:12: DeprecationWarning: 
.ix is deprecated. Please use
.loc for label based indexing or
.iloc for positional indexing

See the documentation here:
http://pandas.pydata.org/pandas-docs/stable/indexing.html#ix-indexer-is-deprecated
 22%|██▉          | 45/200 [00:36<02:04,  1.24it/s]C:\Anaconda3\lib\site-packages\ipykernel\__main__.py:12: DeprecationWarning: 
.ix is deprecated. Please use
.loc for label based indexing or
.iloc for positional indexing

See the documentation here:
http://pandas.pydata.org/pandas-docs/stable/indexing.html#ix-indexer-is-deprecated
 23%|██▉          | 46/200 [00:37<02:02,  1.25it/s]C:\Anaconda3\lib\site-packages\ipykernel\__main__.py:12: DeprecationWarning: 
.ix is deprecated. Please use
.loc for label based indexing or
.iloc for positional indexing

See the documentation here:
http://pandas.pydata.org/pandas-docs/stable/indexing.html#ix-indexer-is-deprecated
 24%|███          | 47/200 [00:37<02:02,  1.25it/s]C:\Anaconda3\lib\site-packages\ipykernel\__main__.py:12: DeprecationWarning: 
.ix is deprecated. Please use
.loc for label based indexing or
.iloc for positional indexing

See the documentation here:
http://pandas.pydata.org/pandas-docs/stable/indexing.html#ix-indexer-is-deprecated
 24%|███          | 48/200 [00:38<02:00,  1.26it/s]C:\Anaconda3\lib\site-packages\ipykernel\__main__.py:12: DeprecationWarning: 
.ix is deprecated. Please use
.loc for label based indexing or
.iloc for positional indexing

See the documentation here:
http://pandas.pydata.org/pandas-docs/stable/indexing.html#ix-indexer-is-deprecated
 24%|███▏         | 49/200 [00:39<02:00,  1.26it/s]C:\Anaconda3\lib\site-packages\ipykernel\__main__.py:12: DeprecationWarning: 
.ix is deprecated. Please use
.loc for label based indexing or
.iloc for positional indexing

See the documentation here:
http://pandas.pydata.org/pandas-docs/stable/indexing.html#ix-indexer-is-deprecated
 25%|███▎         | 50/200 [00:40<02:00,  1.25it/s]C:\Anaconda3\lib\site-packages\ipykernel\__main__.py:12: DeprecationWarning: 
.ix is deprecated. Please use
.loc for label based indexing or
.iloc for positional indexing

See the documentation here:
http://pandas.pydata.org/pandas-docs/stable/indexing.html#ix-indexer-is-deprecated
 26%|███▎         | 51/200 [00:41<02:00,  1.23it/s]C:\Anaconda3\lib\site-packages\ipykernel\__main__.py:12: DeprecationWarning: 
.ix is deprecated. Please use
.loc for label based indexing or
.iloc for positional indexing

See the documentation here:
http://pandas.pydata.org/pandas-docs/stable/indexing.html#ix-indexer-is-deprecated
 26%|███▍         | 52/200 [00:41<01:59,  1.24it/s]C:\Anaconda3\lib\site-packages\ipykernel\__main__.py:12: DeprecationWarning: 
.ix is deprecated. Please use
.loc for label based indexing or
.iloc for positional indexing

See the documentation here:
http://pandas.pydata.org/pandas-docs/stable/indexing.html#ix-indexer-is-deprecated
 26%|███▍         | 53/200 [00:42<01:58,  1.24it/s]C:\Anaconda3\lib\site-packages\ipykernel\__main__.py:12: DeprecationWarning: 
.ix is deprecated. Please use
.loc for label based indexing or
.iloc for positional indexing

See the documentation here:
http://pandas.pydata.org/pandas-docs/stable/indexing.html#ix-indexer-is-deprecated
 27%|███▌         | 54/200 [00:43<01:57,  1.24it/s]C:\Anaconda3\lib\site-packages\ipykernel\__main__.py:12: DeprecationWarning: 
.ix is deprecated. Please use
.loc for label based indexing or
.iloc for positional indexing

See the documentation here:
http://pandas.pydata.org/pandas-docs/stable/indexing.html#ix-indexer-is-deprecated
 28%|███▌         | 55/200 [00:44<01:57,  1.24it/s]C:\Anaconda3\lib\site-packages\ipykernel\__main__.py:12: DeprecationWarning: 
.ix is deprecated. Please use
.loc for label based indexing or
.iloc for positional indexing

See the documentation here:
http://pandas.pydata.org/pandas-docs/stable/indexing.html#ix-indexer-is-deprecated
 28%|███▋         | 56/200 [00:45<01:56,  1.24it/s]C:\Anaconda3\lib\site-packages\ipykernel\__main__.py:12: DeprecationWarning: 
.ix is deprecated. Please use
.loc for label based indexing or
.iloc for positional indexing

See the documentation here:
http://pandas.pydata.org/pandas-docs/stable/indexing.html#ix-indexer-is-deprecated
 28%|███▋         | 57/200 [00:45<01:56,  1.23it/s]C:\Anaconda3\lib\site-packages\ipykernel\__main__.py:12: DeprecationWarning: 
.ix is deprecated. Please use
.loc for label based indexing or
.iloc for positional indexing

See the documentation here:
http://pandas.pydata.org/pandas-docs/stable/indexing.html#ix-indexer-is-deprecated
 29%|███▊         | 58/200 [00:46<01:54,  1.24it/s]C:\Anaconda3\lib\site-packages\ipykernel\__main__.py:12: DeprecationWarning: 
.ix is deprecated. Please use
.loc for label based indexing or
.iloc for positional indexing

See the documentation here:
http://pandas.pydata.org/pandas-docs/stable/indexing.html#ix-indexer-is-deprecated
 30%|███▊         | 59/200 [00:47<01:53,  1.24it/s]C:\Anaconda3\lib\site-packages\ipykernel\__main__.py:12: DeprecationWarning: 
.ix is deprecated. Please use
.loc for label based indexing or
.iloc for positional indexing

See the documentation here:
http://pandas.pydata.org/pandas-docs/stable/indexing.html#ix-indexer-is-deprecated
 30%|███▉         | 60/200 [00:48<01:53,  1.24it/s]C:\Anaconda3\lib\site-packages\ipykernel\__main__.py:12: DeprecationWarning: 
.ix is deprecated. Please use
.loc for label based indexing or
.iloc for positional indexing

See the documentation here:
http://pandas.pydata.org/pandas-docs/stable/indexing.html#ix-indexer-is-deprecated
 30%|███▉         | 61/200 [00:49<01:52,  1.24it/s]C:\Anaconda3\lib\site-packages\ipykernel\__main__.py:12: DeprecationWarning: 
.ix is deprecated. Please use
.loc for label based indexing or
.iloc for positional indexing

See the documentation here:
http://pandas.pydata.org/pandas-docs/stable/indexing.html#ix-indexer-is-deprecated
 31%|████         | 62/200 [00:49<01:51,  1.24it/s]C:\Anaconda3\lib\site-packages\ipykernel\__main__.py:12: DeprecationWarning: 
.ix is deprecated. Please use
.loc for label based indexing or
.iloc for positional indexing

See the documentation here:
http://pandas.pydata.org/pandas-docs/stable/indexing.html#ix-indexer-is-deprecated
 32%|████         | 63/200 [00:50<01:51,  1.23it/s]C:\Anaconda3\lib\site-packages\ipykernel\__main__.py:12: DeprecationWarning: 
.ix is deprecated. Please use
.loc for label based indexing or
.iloc for positional indexing

See the documentation here:
http://pandas.pydata.org/pandas-docs/stable/indexing.html#ix-indexer-is-deprecated
 32%|████▏        | 64/200 [00:51<01:49,  1.24it/s]C:\Anaconda3\lib\site-packages\ipykernel\__main__.py:12: DeprecationWarning: 
.ix is deprecated. Please use
.loc for label based indexing or
.iloc for positional indexing

See the documentation here:
http://pandas.pydata.org/pandas-docs/stable/indexing.html#ix-indexer-is-deprecated
 32%|████▏        | 65/200 [00:52<01:48,  1.25it/s]C:\Anaconda3\lib\site-packages\ipykernel\__main__.py:12: DeprecationWarning: 
.ix is deprecated. Please use
.loc for label based indexing or
.iloc for positional indexing

See the documentation here:
http://pandas.pydata.org/pandas-docs/stable/indexing.html#ix-indexer-is-deprecated
 33%|████▎        | 66/200 [00:53<01:46,  1.25it/s]C:\Anaconda3\lib\site-packages\ipykernel\__main__.py:12: DeprecationWarning: 
.ix is deprecated. Please use
.loc for label based indexing or
.iloc for positional indexing

See the documentation here:
http://pandas.pydata.org/pandas-docs/stable/indexing.html#ix-indexer-is-deprecated
 34%|████▎        | 67/200 [00:53<01:46,  1.25it/s]C:\Anaconda3\lib\site-packages\ipykernel\__main__.py:12: DeprecationWarning: 
.ix is deprecated. Please use
.loc for label based indexing or
.iloc for positional indexing

See the documentation here:
http://pandas.pydata.org/pandas-docs/stable/indexing.html#ix-indexer-is-deprecated
 34%|████▍        | 68/200 [00:54<01:46,  1.24it/s]C:\Anaconda3\lib\site-packages\ipykernel\__main__.py:12: DeprecationWarning: 
.ix is deprecated. Please use
.loc for label based indexing or
.iloc for positional indexing

See the documentation here:
http://pandas.pydata.org/pandas-docs/stable/indexing.html#ix-indexer-is-deprecated
 34%|████▍        | 69/200 [00:55<01:45,  1.24it/s]C:\Anaconda3\lib\site-packages\ipykernel\__main__.py:12: DeprecationWarning: 
.ix is deprecated. Please use
.loc for label based indexing or
.iloc for positional indexing

See the documentation here:
http://pandas.pydata.org/pandas-docs/stable/indexing.html#ix-indexer-is-deprecated
 35%|████▌        | 70/200 [00:56<01:44,  1.24it/s]C:\Anaconda3\lib\site-packages\ipykernel\__main__.py:12: DeprecationWarning: 
.ix is deprecated. Please use
.loc for label based indexing or
.iloc for positional indexing

See the documentation here:
http://pandas.pydata.org/pandas-docs/stable/indexing.html#ix-indexer-is-deprecated
 36%|████▌        | 71/200 [00:57<01:42,  1.26it/s]C:\Anaconda3\lib\site-packages\ipykernel\__main__.py:12: DeprecationWarning: 
.ix is deprecated. Please use
.loc for label based indexing or
.iloc for positional indexing

See the documentation here:
http://pandas.pydata.org/pandas-docs/stable/indexing.html#ix-indexer-is-deprecated
 36%|████▋        | 72/200 [00:58<01:42,  1.25it/s]C:\Anaconda3\lib\site-packages\ipykernel\__main__.py:12: DeprecationWarning: 
.ix is deprecated. Please use
.loc for label based indexing or
.iloc for positional indexing

See the documentation here:
http://pandas.pydata.org/pandas-docs/stable/indexing.html#ix-indexer-is-deprecated
 36%|████▋        | 73/200 [00:58<01:42,  1.24it/s]C:\Anaconda3\lib\site-packages\ipykernel\__main__.py:12: DeprecationWarning: 
.ix is deprecated. Please use
.loc for label based indexing or
.iloc for positional indexing

See the documentation here:
http://pandas.pydata.org/pandas-docs/stable/indexing.html#ix-indexer-is-deprecated
 37%|████▊        | 74/200 [00:59<01:41,  1.24it/s]C:\Anaconda3\lib\site-packages\ipykernel\__main__.py:12: DeprecationWarning: 
.ix is deprecated. Please use
.loc for label based indexing or
.iloc for positional indexing

See the documentation here:
http://pandas.pydata.org/pandas-docs/stable/indexing.html#ix-indexer-is-deprecated
 38%|████▉        | 75/200 [01:00<01:40,  1.24it/s]C:\Anaconda3\lib\site-packages\ipykernel\__main__.py:12: DeprecationWarning: 
.ix is deprecated. Please use
.loc for label based indexing or
.iloc for positional indexing

See the documentation here:
http://pandas.pydata.org/pandas-docs/stable/indexing.html#ix-indexer-is-deprecated
 38%|████▉        | 76/200 [01:01<01:41,  1.23it/s]C:\Anaconda3\lib\site-packages\ipykernel\__main__.py:12: DeprecationWarning: 
.ix is deprecated. Please use
.loc for label based indexing or
.iloc for positional indexing

See the documentation here:
http://pandas.pydata.org/pandas-docs/stable/indexing.html#ix-indexer-is-deprecated
 38%|█████        | 77/200 [01:02<01:39,  1.24it/s]C:\Anaconda3\lib\site-packages\ipykernel\__main__.py:12: DeprecationWarning: 
.ix is deprecated. Please use
.loc for label based indexing or
.iloc for positional indexing

See the documentation here:
http://pandas.pydata.org/pandas-docs/stable/indexing.html#ix-indexer-is-deprecated
 39%|█████        | 78/200 [01:02<01:37,  1.25it/s]C:\Anaconda3\lib\site-packages\ipykernel\__main__.py:12: DeprecationWarning: 
.ix is deprecated. Please use
.loc for label based indexing or
.iloc for positional indexing

See the documentation here:
http://pandas.pydata.org/pandas-docs/stable/indexing.html#ix-indexer-is-deprecated
 40%|█████▏       | 79/200 [01:03<01:37,  1.25it/s]C:\Anaconda3\lib\site-packages\ipykernel\__main__.py:12: DeprecationWarning: 
.ix is deprecated. Please use
.loc for label based indexing or
.iloc for positional indexing

See the documentation here:
http://pandas.pydata.org/pandas-docs/stable/indexing.html#ix-indexer-is-deprecated
 40%|█████▏       | 80/200 [01:04<01:37,  1.23it/s]C:\Anaconda3\lib\site-packages\ipykernel\__main__.py:12: DeprecationWarning: 
.ix is deprecated. Please use
.loc for label based indexing or
.iloc for positional indexing

See the documentation here:
http://pandas.pydata.org/pandas-docs/stable/indexing.html#ix-indexer-is-deprecated
 40%|█████▎       | 81/200 [01:05<01:35,  1.25it/s]C:\Anaconda3\lib\site-packages\ipykernel\__main__.py:12: DeprecationWarning: 
.ix is deprecated. Please use
.loc for label based indexing or
.iloc for positional indexing

See the documentation here:
http://pandas.pydata.org/pandas-docs/stable/indexing.html#ix-indexer-is-deprecated
 41%|█████▎       | 82/200 [01:06<01:34,  1.25it/s]C:\Anaconda3\lib\site-packages\ipykernel\__main__.py:12: DeprecationWarning: 
.ix is deprecated. Please use
.loc for label based indexing or
.iloc for positional indexing

See the documentation here:
http://pandas.pydata.org/pandas-docs/stable/indexing.html#ix-indexer-is-deprecated
 42%|█████▍       | 83/200 [01:06<01:34,  1.24it/s]C:\Anaconda3\lib\site-packages\ipykernel\__main__.py:12: DeprecationWarning: 
.ix is deprecated. Please use
.loc for label based indexing or
.iloc for positional indexing

See the documentation here:
http://pandas.pydata.org/pandas-docs/stable/indexing.html#ix-indexer-is-deprecated
 42%|█████▍       | 84/200 [01:07<01:32,  1.25it/s]C:\Anaconda3\lib\site-packages\ipykernel\__main__.py:12: DeprecationWarning: 
.ix is deprecated. Please use
.loc for label based indexing or
.iloc for positional indexing

See the documentation here:
http://pandas.pydata.org/pandas-docs/stable/indexing.html#ix-indexer-is-deprecated
 42%|█████▌       | 85/200 [01:08<01:31,  1.26it/s]C:\Anaconda3\lib\site-packages\ipykernel\__main__.py:12: DeprecationWarning: 
.ix is deprecated. Please use
.loc for label based indexing or
.iloc for positional indexing

See the documentation here:
http://pandas.pydata.org/pandas-docs/stable/indexing.html#ix-indexer-is-deprecated
 43%|█████▌       | 86/200 [01:09<01:31,  1.25it/s]C:\Anaconda3\lib\site-packages\ipykernel\__main__.py:12: DeprecationWarning: 
.ix is deprecated. Please use
.loc for label based indexing or
.iloc for positional indexing

See the documentation here:
http://pandas.pydata.org/pandas-docs/stable/indexing.html#ix-indexer-is-deprecated
 44%|█████▋       | 87/200 [01:10<01:30,  1.25it/s]C:\Anaconda3\lib\site-packages\ipykernel\__main__.py:12: DeprecationWarning: 
.ix is deprecated. Please use
.loc for label based indexing or
.iloc for positional indexing

See the documentation here:
http://pandas.pydata.org/pandas-docs/stable/indexing.html#ix-indexer-is-deprecated
 44%|█████▋       | 88/200 [01:10<01:32,  1.22it/s]C:\Anaconda3\lib\site-packages\ipykernel\__main__.py:12: DeprecationWarning: 
.ix is deprecated. Please use
.loc for label based indexing or
.iloc for positional indexing

See the documentation here:
http://pandas.pydata.org/pandas-docs/stable/indexing.html#ix-indexer-is-deprecated
 44%|█████▊       | 89/200 [01:11<01:36,  1.15it/s]C:\Anaconda3\lib\site-packages\ipykernel\__main__.py:12: DeprecationWarning: 
.ix is deprecated. Please use
.loc for label based indexing or
.iloc for positional indexing

See the documentation here:
http://pandas.pydata.org/pandas-docs/stable/indexing.html#ix-indexer-is-deprecated
 45%|█████▊       | 90/200 [01:12<01:33,  1.18it/s]C:\Anaconda3\lib\site-packages\ipykernel\__main__.py:12: DeprecationWarning: 
.ix is deprecated. Please use
.loc for label based indexing or
.iloc for positional indexing

See the documentation here:
http://pandas.pydata.org/pandas-docs/stable/indexing.html#ix-indexer-is-deprecated
 46%|█████▉       | 91/200 [01:13<01:33,  1.16it/s]C:\Anaconda3\lib\site-packages\ipykernel\__main__.py:12: DeprecationWarning: 
.ix is deprecated. Please use
.loc for label based indexing or
.iloc for positional indexing

See the documentation here:
http://pandas.pydata.org/pandas-docs/stable/indexing.html#ix-indexer-is-deprecated
 46%|█████▉       | 92/200 [01:14<01:31,  1.19it/s]C:\Anaconda3\lib\site-packages\ipykernel\__main__.py:12: DeprecationWarning: 
.ix is deprecated. Please use
.loc for label based indexing or
.iloc for positional indexing

See the documentation here:
http://pandas.pydata.org/pandas-docs/stable/indexing.html#ix-indexer-is-deprecated
 46%|██████       | 93/200 [01:15<01:31,  1.17it/s]C:\Anaconda3\lib\site-packages\ipykernel\__main__.py:12: DeprecationWarning: 
.ix is deprecated. Please use
.loc for label based indexing or
.iloc for positional indexing

See the documentation here:
http://pandas.pydata.org/pandas-docs/stable/indexing.html#ix-indexer-is-deprecated
 47%|██████       | 94/200 [01:16<01:29,  1.18it/s]C:\Anaconda3\lib\site-packages\ipykernel\__main__.py:12: DeprecationWarning: 
.ix is deprecated. Please use
.loc for label based indexing or
.iloc for positional indexing

See the documentation here:
http://pandas.pydata.org/pandas-docs/stable/indexing.html#ix-indexer-is-deprecated
 48%|██████▏      | 95/200 [01:16<01:27,  1.20it/s]C:\Anaconda3\lib\site-packages\ipykernel\__main__.py:12: DeprecationWarning: 
.ix is deprecated. Please use
.loc for label based indexing or
.iloc for positional indexing

See the documentation here:
http://pandas.pydata.org/pandas-docs/stable/indexing.html#ix-indexer-is-deprecated
 48%|██████▏      | 96/200 [01:17<01:25,  1.22it/s]C:\Anaconda3\lib\site-packages\ipykernel\__main__.py:12: DeprecationWarning: 
.ix is deprecated. Please use
.loc for label based indexing or
.iloc for positional indexing

See the documentation here:
http://pandas.pydata.org/pandas-docs/stable/indexing.html#ix-indexer-is-deprecated
 48%|██████▎      | 97/200 [01:18<01:23,  1.23it/s]C:\Anaconda3\lib\site-packages\ipykernel\__main__.py:12: DeprecationWarning: 
.ix is deprecated. Please use
.loc for label based indexing or
.iloc for positional indexing

See the documentation here:
http://pandas.pydata.org/pandas-docs/stable/indexing.html#ix-indexer-is-deprecated
 49%|██████▎      | 98/200 [01:19<01:22,  1.24it/s]C:\Anaconda3\lib\site-packages\ipykernel\__main__.py:12: DeprecationWarning: 
.ix is deprecated. Please use
.loc for label based indexing or
.iloc for positional indexing

See the documentation here:
http://pandas.pydata.org/pandas-docs/stable/indexing.html#ix-indexer-is-deprecated
 50%|██████▍      | 99/200 [01:20<01:20,  1.25it/s]C:\Anaconda3\lib\site-packages\ipykernel\__main__.py:12: DeprecationWarning: 
.ix is deprecated. Please use
.loc for label based indexing or
.iloc for positional indexing

See the documentation here:
http://pandas.pydata.org/pandas-docs/stable/indexing.html#ix-indexer-is-deprecated
 50%|██████      | 100/200 [01:20<01:19,  1.25it/s]C:\Anaconda3\lib\site-packages\ipykernel\__main__.py:12: DeprecationWarning: 
.ix is deprecated. Please use
.loc for label based indexing or
.iloc for positional indexing

See the documentation here:
http://pandas.pydata.org/pandas-docs/stable/indexing.html#ix-indexer-is-deprecated
 50%|██████      | 101/200 [01:21<01:19,  1.25it/s]C:\Anaconda3\lib\site-packages\ipykernel\__main__.py:12: DeprecationWarning: 
.ix is deprecated. Please use
.loc for label based indexing or
.iloc for positional indexing

See the documentation here:
http://pandas.pydata.org/pandas-docs/stable/indexing.html#ix-indexer-is-deprecated
 51%|██████      | 102/200 [01:22<01:18,  1.25it/s]C:\Anaconda3\lib\site-packages\ipykernel\__main__.py:12: DeprecationWarning: 
.ix is deprecated. Please use
.loc for label based indexing or
.iloc for positional indexing

See the documentation here:
http://pandas.pydata.org/pandas-docs/stable/indexing.html#ix-indexer-is-deprecated
 52%|██████▏     | 103/200 [01:23<01:17,  1.25it/s]C:\Anaconda3\lib\site-packages\ipykernel\__main__.py:12: DeprecationWarning: 
.ix is deprecated. Please use
.loc for label based indexing or
.iloc for positional indexing

See the documentation here:
http://pandas.pydata.org/pandas-docs/stable/indexing.html#ix-indexer-is-deprecated
 52%|██████▏     | 104/200 [01:24<01:17,  1.24it/s]C:\Anaconda3\lib\site-packages\ipykernel\__main__.py:12: DeprecationWarning: 
.ix is deprecated. Please use
.loc for label based indexing or
.iloc for positional indexing

See the documentation here:
http://pandas.pydata.org/pandas-docs/stable/indexing.html#ix-indexer-is-deprecated
 52%|██████▎     | 105/200 [01:24<01:17,  1.23it/s]C:\Anaconda3\lib\site-packages\ipykernel\__main__.py:12: DeprecationWarning: 
.ix is deprecated. Please use
.loc for label based indexing or
.iloc for positional indexing

See the documentation here:
http://pandas.pydata.org/pandas-docs/stable/indexing.html#ix-indexer-is-deprecated
 53%|██████▎     | 106/200 [01:25<01:17,  1.21it/s]C:\Anaconda3\lib\site-packages\ipykernel\__main__.py:12: DeprecationWarning: 
.ix is deprecated. Please use
.loc for label based indexing or
.iloc for positional indexing

See the documentation here:
http://pandas.pydata.org/pandas-docs/stable/indexing.html#ix-indexer-is-deprecated
 54%|██████▍     | 107/200 [01:26<01:16,  1.22it/s]C:\Anaconda3\lib\site-packages\ipykernel\__main__.py:12: DeprecationWarning: 
.ix is deprecated. Please use
.loc for label based indexing or
.iloc for positional indexing

See the documentation here:
http://pandas.pydata.org/pandas-docs/stable/indexing.html#ix-indexer-is-deprecated
 54%|██████▍     | 108/200 [01:27<01:15,  1.22it/s]C:\Anaconda3\lib\site-packages\ipykernel\__main__.py:12: DeprecationWarning: 
.ix is deprecated. Please use
.loc for label based indexing or
.iloc for positional indexing

See the documentation here:
http://pandas.pydata.org/pandas-docs/stable/indexing.html#ix-indexer-is-deprecated
 55%|██████▌     | 109/200 [01:28<01:14,  1.23it/s]C:\Anaconda3\lib\site-packages\ipykernel\__main__.py:12: DeprecationWarning: 
.ix is deprecated. Please use
.loc for label based indexing or
.iloc for positional indexing

See the documentation here:
http://pandas.pydata.org/pandas-docs/stable/indexing.html#ix-indexer-is-deprecated
 55%|██████▌     | 110/200 [01:29<01:15,  1.20it/s]C:\Anaconda3\lib\site-packages\ipykernel\__main__.py:12: DeprecationWarning: 
.ix is deprecated. Please use
.loc for label based indexing or
.iloc for positional indexing

See the documentation here:
http://pandas.pydata.org/pandas-docs/stable/indexing.html#ix-indexer-is-deprecated
 56%|██████▋     | 111/200 [01:29<01:13,  1.22it/s]C:\Anaconda3\lib\site-packages\ipykernel\__main__.py:12: DeprecationWarning: 
.ix is deprecated. Please use
.loc for label based indexing or
.iloc for positional indexing

See the documentation here:
http://pandas.pydata.org/pandas-docs/stable/indexing.html#ix-indexer-is-deprecated
 56%|██████▋     | 112/200 [01:30<01:12,  1.22it/s]C:\Anaconda3\lib\site-packages\ipykernel\__main__.py:12: DeprecationWarning: 
.ix is deprecated. Please use
.loc for label based indexing or
.iloc for positional indexing

See the documentation here:
http://pandas.pydata.org/pandas-docs/stable/indexing.html#ix-indexer-is-deprecated
 56%|██████▊     | 113/200 [01:31<01:10,  1.23it/s]C:\Anaconda3\lib\site-packages\ipykernel\__main__.py:12: DeprecationWarning: 
.ix is deprecated. Please use
.loc for label based indexing or
.iloc for positional indexing

See the documentation here:
http://pandas.pydata.org/pandas-docs/stable/indexing.html#ix-indexer-is-deprecated
 57%|██████▊     | 114/200 [01:32<01:09,  1.24it/s]C:\Anaconda3\lib\site-packages\ipykernel\__main__.py:12: DeprecationWarning: 
.ix is deprecated. Please use
.loc for label based indexing or
.iloc for positional indexing

See the documentation here:
http://pandas.pydata.org/pandas-docs/stable/indexing.html#ix-indexer-is-deprecated
 57%|██████▉     | 115/200 [01:33<01:08,  1.25it/s]C:\Anaconda3\lib\site-packages\ipykernel\__main__.py:12: DeprecationWarning: 
.ix is deprecated. Please use
.loc for label based indexing or
.iloc for positional indexing

See the documentation here:
http://pandas.pydata.org/pandas-docs/stable/indexing.html#ix-indexer-is-deprecated
 58%|██████▉     | 116/200 [01:33<01:07,  1.24it/s]C:\Anaconda3\lib\site-packages\ipykernel\__main__.py:12: DeprecationWarning: 
.ix is deprecated. Please use
.loc for label based indexing or
.iloc for positional indexing

See the documentation here:
http://pandas.pydata.org/pandas-docs/stable/indexing.html#ix-indexer-is-deprecated
 58%|███████     | 117/200 [01:34<01:06,  1.25it/s]C:\Anaconda3\lib\site-packages\ipykernel\__main__.py:12: DeprecationWarning: 
.ix is deprecated. Please use
.loc for label based indexing or
.iloc for positional indexing

See the documentation here:
http://pandas.pydata.org/pandas-docs/stable/indexing.html#ix-indexer-is-deprecated
 59%|███████     | 118/200 [01:35<01:07,  1.22it/s]C:\Anaconda3\lib\site-packages\ipykernel\__main__.py:12: DeprecationWarning: 
.ix is deprecated. Please use
.loc for label based indexing or
.iloc for positional indexing

See the documentation here:
http://pandas.pydata.org/pandas-docs/stable/indexing.html#ix-indexer-is-deprecated
 60%|███████▏    | 119/200 [01:36<01:05,  1.23it/s]C:\Anaconda3\lib\site-packages\ipykernel\__main__.py:12: DeprecationWarning: 
.ix is deprecated. Please use
.loc for label based indexing or
.iloc for positional indexing

See the documentation here:
http://pandas.pydata.org/pandas-docs/stable/indexing.html#ix-indexer-is-deprecated
 60%|███████▏    | 120/200 [01:37<01:05,  1.22it/s]C:\Anaconda3\lib\site-packages\ipykernel\__main__.py:12: DeprecationWarning: 
.ix is deprecated. Please use
.loc for label based indexing or
.iloc for positional indexing

See the documentation here:
http://pandas.pydata.org/pandas-docs/stable/indexing.html#ix-indexer-is-deprecated
 60%|███████▎    | 121/200 [01:37<01:04,  1.23it/s]C:\Anaconda3\lib\site-packages\ipykernel\__main__.py:12: DeprecationWarning: 
.ix is deprecated. Please use
.loc for label based indexing or
.iloc for positional indexing

See the documentation here:
http://pandas.pydata.org/pandas-docs/stable/indexing.html#ix-indexer-is-deprecated
 61%|███████▎    | 122/200 [01:38<01:04,  1.22it/s]C:\Anaconda3\lib\site-packages\ipykernel\__main__.py:12: DeprecationWarning: 
.ix is deprecated. Please use
.loc for label based indexing or
.iloc for positional indexing

See the documentation here:
http://pandas.pydata.org/pandas-docs/stable/indexing.html#ix-indexer-is-deprecated
 62%|███████▍    | 123/200 [01:39<01:03,  1.21it/s]C:\Anaconda3\lib\site-packages\ipykernel\__main__.py:12: DeprecationWarning: 
.ix is deprecated. Please use
.loc for label based indexing or
.iloc for positional indexing

See the documentation here:
http://pandas.pydata.org/pandas-docs/stable/indexing.html#ix-indexer-is-deprecated
 62%|███████▍    | 124/200 [01:40<01:03,  1.19it/s]C:\Anaconda3\lib\site-packages\ipykernel\__main__.py:12: DeprecationWarning: 
.ix is deprecated. Please use
.loc for label based indexing or
.iloc for positional indexing

See the documentation here:
http://pandas.pydata.org/pandas-docs/stable/indexing.html#ix-indexer-is-deprecated
 62%|███████▌    | 125/200 [01:41<01:02,  1.20it/s]C:\Anaconda3\lib\site-packages\ipykernel\__main__.py:12: DeprecationWarning: 
.ix is deprecated. Please use
.loc for label based indexing or
.iloc for positional indexing

See the documentation here:
http://pandas.pydata.org/pandas-docs/stable/indexing.html#ix-indexer-is-deprecated
 63%|███████▌    | 126/200 [01:42<01:00,  1.22it/s]C:\Anaconda3\lib\site-packages\ipykernel\__main__.py:12: DeprecationWarning: 
.ix is deprecated. Please use
.loc for label based indexing or
.iloc for positional indexing

See the documentation here:
http://pandas.pydata.org/pandas-docs/stable/indexing.html#ix-indexer-is-deprecated
 64%|███████▌    | 127/200 [01:42<01:00,  1.22it/s]C:\Anaconda3\lib\site-packages\ipykernel\__main__.py:12: DeprecationWarning: 
.ix is deprecated. Please use
.loc for label based indexing or
.iloc for positional indexing

See the documentation here:
http://pandas.pydata.org/pandas-docs/stable/indexing.html#ix-indexer-is-deprecated
 64%|███████▋    | 128/200 [01:43<01:00,  1.20it/s]C:\Anaconda3\lib\site-packages\ipykernel\__main__.py:12: DeprecationWarning: 
.ix is deprecated. Please use
.loc for label based indexing or
.iloc for positional indexing

See the documentation here:
http://pandas.pydata.org/pandas-docs/stable/indexing.html#ix-indexer-is-deprecated
 64%|███████▋    | 129/200 [01:44<00:58,  1.22it/s]C:\Anaconda3\lib\site-packages\ipykernel\__main__.py:12: DeprecationWarning: 
.ix is deprecated. Please use
.loc for label based indexing or
.iloc for positional indexing

See the documentation here:
http://pandas.pydata.org/pandas-docs/stable/indexing.html#ix-indexer-is-deprecated
 65%|███████▊    | 130/200 [01:45<00:57,  1.22it/s]C:\Anaconda3\lib\site-packages\ipykernel\__main__.py:12: DeprecationWarning: 
.ix is deprecated. Please use
.loc for label based indexing or
.iloc for positional indexing

See the documentation here:
http://pandas.pydata.org/pandas-docs/stable/indexing.html#ix-indexer-is-deprecated
 66%|███████▊    | 131/200 [01:46<00:55,  1.24it/s]C:\Anaconda3\lib\site-packages\ipykernel\__main__.py:12: DeprecationWarning: 
.ix is deprecated. Please use
.loc for label based indexing or
.iloc for positional indexing

See the documentation here:
http://pandas.pydata.org/pandas-docs/stable/indexing.html#ix-indexer-is-deprecated
 66%|███████▉    | 132/200 [01:46<00:54,  1.25it/s]C:\Anaconda3\lib\site-packages\ipykernel\__main__.py:12: DeprecationWarning: 
.ix is deprecated. Please use
.loc for label based indexing or
.iloc for positional indexing

See the documentation here:
http://pandas.pydata.org/pandas-docs/stable/indexing.html#ix-indexer-is-deprecated
 66%|███████▉    | 133/200 [01:47<00:53,  1.25it/s]C:\Anaconda3\lib\site-packages\ipykernel\__main__.py:12: DeprecationWarning: 
.ix is deprecated. Please use
.loc for label based indexing or
.iloc for positional indexing

See the documentation here:
http://pandas.pydata.org/pandas-docs/stable/indexing.html#ix-indexer-is-deprecated
 67%|████████    | 134/200 [01:48<00:52,  1.25it/s]C:\Anaconda3\lib\site-packages\ipykernel\__main__.py:12: DeprecationWarning: 
.ix is deprecated. Please use
.loc for label based indexing or
.iloc for positional indexing

See the documentation here:
http://pandas.pydata.org/pandas-docs/stable/indexing.html#ix-indexer-is-deprecated
 68%|████████    | 135/200 [01:49<00:52,  1.25it/s]C:\Anaconda3\lib\site-packages\ipykernel\__main__.py:12: DeprecationWarning: 
.ix is deprecated. Please use
.loc for label based indexing or
.iloc for positional indexing

See the documentation here:
http://pandas.pydata.org/pandas-docs/stable/indexing.html#ix-indexer-is-deprecated
 68%|████████▏   | 136/200 [01:50<00:51,  1.24it/s]C:\Anaconda3\lib\site-packages\ipykernel\__main__.py:12: DeprecationWarning: 
.ix is deprecated. Please use
.loc for label based indexing or
.iloc for positional indexing

See the documentation here:
http://pandas.pydata.org/pandas-docs/stable/indexing.html#ix-indexer-is-deprecated
 68%|████████▏   | 137/200 [01:50<00:50,  1.25it/s]C:\Anaconda3\lib\site-packages\ipykernel\__main__.py:12: DeprecationWarning: 
.ix is deprecated. Please use
.loc for label based indexing or
.iloc for positional indexing

See the documentation here:
http://pandas.pydata.org/pandas-docs/stable/indexing.html#ix-indexer-is-deprecated
 69%|████████▎   | 138/200 [01:51<00:49,  1.26it/s]C:\Anaconda3\lib\site-packages\ipykernel\__main__.py:12: DeprecationWarning: 
.ix is deprecated. Please use
.loc for label based indexing or
.iloc for positional indexing

See the documentation here:
http://pandas.pydata.org/pandas-docs/stable/indexing.html#ix-indexer-is-deprecated
 70%|████████▎   | 139/200 [01:52<00:48,  1.26it/s]C:\Anaconda3\lib\site-packages\ipykernel\__main__.py:12: DeprecationWarning: 
.ix is deprecated. Please use
.loc for label based indexing or
.iloc for positional indexing

See the documentation here:
http://pandas.pydata.org/pandas-docs/stable/indexing.html#ix-indexer-is-deprecated
 70%|████████▍   | 140/200 [01:53<00:48,  1.25it/s]C:\Anaconda3\lib\site-packages\ipykernel\__main__.py:12: DeprecationWarning: 
.ix is deprecated. Please use
.loc for label based indexing or
.iloc for positional indexing

See the documentation here:
http://pandas.pydata.org/pandas-docs/stable/indexing.html#ix-indexer-is-deprecated
 70%|████████▍   | 141/200 [01:54<00:47,  1.25it/s]C:\Anaconda3\lib\site-packages\ipykernel\__main__.py:12: DeprecationWarning: 
.ix is deprecated. Please use
.loc for label based indexing or
.iloc for positional indexing

See the documentation here:
http://pandas.pydata.org/pandas-docs/stable/indexing.html#ix-indexer-is-deprecated
 71%|████████▌   | 142/200 [01:55<00:47,  1.21it/s]C:\Anaconda3\lib\site-packages\ipykernel\__main__.py:12: DeprecationWarning: 
.ix is deprecated. Please use
.loc for label based indexing or
.iloc for positional indexing

See the documentation here:
http://pandas.pydata.org/pandas-docs/stable/indexing.html#ix-indexer-is-deprecated
 72%|████████▌   | 143/200 [01:55<00:46,  1.22it/s]C:\Anaconda3\lib\site-packages\ipykernel\__main__.py:12: DeprecationWarning: 
.ix is deprecated. Please use
.loc for label based indexing or
.iloc for positional indexing

See the documentation here:
http://pandas.pydata.org/pandas-docs/stable/indexing.html#ix-indexer-is-deprecated
 72%|████████▋   | 144/200 [01:56<00:45,  1.22it/s]C:\Anaconda3\lib\site-packages\ipykernel\__main__.py:12: DeprecationWarning: 
.ix is deprecated. Please use
.loc for label based indexing or
.iloc for positional indexing

See the documentation here:
http://pandas.pydata.org/pandas-docs/stable/indexing.html#ix-indexer-is-deprecated
 72%|████████▋   | 145/200 [01:57<00:45,  1.21it/s]C:\Anaconda3\lib\site-packages\ipykernel\__main__.py:12: DeprecationWarning: 
.ix is deprecated. Please use
.loc for label based indexing or
.iloc for positional indexing

See the documentation here:
http://pandas.pydata.org/pandas-docs/stable/indexing.html#ix-indexer-is-deprecated
 73%|████████▊   | 146/200 [01:58<00:44,  1.22it/s]C:\Anaconda3\lib\site-packages\ipykernel\__main__.py:12: DeprecationWarning: 
.ix is deprecated. Please use
.loc for label based indexing or
.iloc for positional indexing

See the documentation here:
http://pandas.pydata.org/pandas-docs/stable/indexing.html#ix-indexer-is-deprecated
 74%|████████▊   | 147/200 [01:59<00:43,  1.23it/s]C:\Anaconda3\lib\site-packages\ipykernel\__main__.py:12: DeprecationWarning: 
.ix is deprecated. Please use
.loc for label based indexing or
.iloc for positional indexing

See the documentation here:
http://pandas.pydata.org/pandas-docs/stable/indexing.html#ix-indexer-is-deprecated
 74%|████████▉   | 148/200 [01:59<00:42,  1.23it/s]C:\Anaconda3\lib\site-packages\ipykernel\__main__.py:12: DeprecationWarning: 
.ix is deprecated. Please use
.loc for label based indexing or
.iloc for positional indexing

See the documentation here:
http://pandas.pydata.org/pandas-docs/stable/indexing.html#ix-indexer-is-deprecated
 74%|████████▉   | 149/200 [02:00<00:41,  1.24it/s]C:\Anaconda3\lib\site-packages\ipykernel\__main__.py:12: DeprecationWarning: 
.ix is deprecated. Please use
.loc for label based indexing or
.iloc for positional indexing

See the documentation here:
http://pandas.pydata.org/pandas-docs/stable/indexing.html#ix-indexer-is-deprecated
 75%|█████████   | 150/200 [02:01<00:40,  1.24it/s]C:\Anaconda3\lib\site-packages\ipykernel\__main__.py:12: DeprecationWarning: 
.ix is deprecated. Please use
.loc for label based indexing or
.iloc for positional indexing

See the documentation here:
http://pandas.pydata.org/pandas-docs/stable/indexing.html#ix-indexer-is-deprecated
 76%|█████████   | 151/200 [02:02<00:39,  1.24it/s]C:\Anaconda3\lib\site-packages\ipykernel\__main__.py:12: DeprecationWarning: 
.ix is deprecated. Please use
.loc for label based indexing or
.iloc for positional indexing

See the documentation here:
http://pandas.pydata.org/pandas-docs/stable/indexing.html#ix-indexer-is-deprecated
 76%|█████████   | 152/200 [02:03<00:38,  1.24it/s]C:\Anaconda3\lib\site-packages\ipykernel\__main__.py:12: DeprecationWarning: 
.ix is deprecated. Please use
.loc for label based indexing or
.iloc for positional indexing

See the documentation here:
http://pandas.pydata.org/pandas-docs/stable/indexing.html#ix-indexer-is-deprecated
 76%|█████████▏  | 153/200 [02:03<00:37,  1.24it/s]C:\Anaconda3\lib\site-packages\ipykernel\__main__.py:12: DeprecationWarning: 
.ix is deprecated. Please use
.loc for label based indexing or
.iloc for positional indexing

See the documentation here:
http://pandas.pydata.org/pandas-docs/stable/indexing.html#ix-indexer-is-deprecated
 77%|█████████▏  | 154/200 [02:04<00:37,  1.23it/s]C:\Anaconda3\lib\site-packages\ipykernel\__main__.py:12: DeprecationWarning: 
.ix is deprecated. Please use
.loc for label based indexing or
.iloc for positional indexing

See the documentation here:
http://pandas.pydata.org/pandas-docs/stable/indexing.html#ix-indexer-is-deprecated
 78%|█████████▎  | 155/200 [02:05<00:36,  1.24it/s]C:\Anaconda3\lib\site-packages\ipykernel\__main__.py:12: DeprecationWarning: 
.ix is deprecated. Please use
.loc for label based indexing or
.iloc for positional indexing

See the documentation here:
http://pandas.pydata.org/pandas-docs/stable/indexing.html#ix-indexer-is-deprecated
 78%|█████████▎  | 156/200 [02:06<00:36,  1.21it/s]C:\Anaconda3\lib\site-packages\ipykernel\__main__.py:12: DeprecationWarning: 
.ix is deprecated. Please use
.loc for label based indexing or
.iloc for positional indexing

See the documentation here:
http://pandas.pydata.org/pandas-docs/stable/indexing.html#ix-indexer-is-deprecated
 78%|█████████▍  | 157/200 [02:07<00:35,  1.22it/s]C:\Anaconda3\lib\site-packages\ipykernel\__main__.py:12: DeprecationWarning: 
.ix is deprecated. Please use
.loc for label based indexing or
.iloc for positional indexing

See the documentation here:
http://pandas.pydata.org/pandas-docs/stable/indexing.html#ix-indexer-is-deprecated
 79%|█████████▍  | 158/200 [02:08<00:34,  1.23it/s]C:\Anaconda3\lib\site-packages\ipykernel\__main__.py:12: DeprecationWarning: 
.ix is deprecated. Please use
.loc for label based indexing or
.iloc for positional indexing

See the documentation here:
http://pandas.pydata.org/pandas-docs/stable/indexing.html#ix-indexer-is-deprecated
 80%|█████████▌  | 159/200 [02:08<00:33,  1.24it/s]C:\Anaconda3\lib\site-packages\ipykernel\__main__.py:12: DeprecationWarning: 
.ix is deprecated. Please use
.loc for label based indexing or
.iloc for positional indexing

See the documentation here:
http://pandas.pydata.org/pandas-docs/stable/indexing.html#ix-indexer-is-deprecated
 80%|█████████▌  | 160/200 [02:09<00:32,  1.23it/s]C:\Anaconda3\lib\site-packages\ipykernel\__main__.py:12: DeprecationWarning: 
.ix is deprecated. Please use
.loc for label based indexing or
.iloc for positional indexing

See the documentation here:
http://pandas.pydata.org/pandas-docs/stable/indexing.html#ix-indexer-is-deprecated
 80%|█████████▋  | 161/200 [02:10<00:31,  1.24it/s]C:\Anaconda3\lib\site-packages\ipykernel\__main__.py:12: DeprecationWarning: 
.ix is deprecated. Please use
.loc for label based indexing or
.iloc for positional indexing

See the documentation here:
http://pandas.pydata.org/pandas-docs/stable/indexing.html#ix-indexer-is-deprecated
 81%|█████████▋  | 162/200 [02:11<00:30,  1.26it/s]C:\Anaconda3\lib\site-packages\ipykernel\__main__.py:12: DeprecationWarning: 
.ix is deprecated. Please use
.loc for label based indexing or
.iloc for positional indexing

See the documentation here:
http://pandas.pydata.org/pandas-docs/stable/indexing.html#ix-indexer-is-deprecated
 82%|█████████▊  | 163/200 [02:12<00:30,  1.22it/s]C:\Anaconda3\lib\site-packages\ipykernel\__main__.py:12: DeprecationWarning: 
.ix is deprecated. Please use
.loc for label based indexing or
.iloc for positional indexing

See the documentation here:
http://pandas.pydata.org/pandas-docs/stable/indexing.html#ix-indexer-is-deprecated
 82%|█████████▊  | 164/200 [02:13<00:30,  1.17it/s]C:\Anaconda3\lib\site-packages\ipykernel\__main__.py:12: DeprecationWarning: 
.ix is deprecated. Please use
.loc for label based indexing or
.iloc for positional indexing

See the documentation here:
http://pandas.pydata.org/pandas-docs/stable/indexing.html#ix-indexer-is-deprecated
 82%|█████████▉  | 165/200 [02:14<00:30,  1.13it/s]C:\Anaconda3\lib\site-packages\ipykernel\__main__.py:12: DeprecationWarning: 
.ix is deprecated. Please use
.loc for label based indexing or
.iloc for positional indexing

See the documentation here:
http://pandas.pydata.org/pandas-docs/stable/indexing.html#ix-indexer-is-deprecated
 83%|█████████▉  | 166/200 [02:14<00:29,  1.15it/s]C:\Anaconda3\lib\site-packages\ipykernel\__main__.py:12: DeprecationWarning: 
.ix is deprecated. Please use
.loc for label based indexing or
.iloc for positional indexing

See the documentation here:
http://pandas.pydata.org/pandas-docs/stable/indexing.html#ix-indexer-is-deprecated
 84%|██████████  | 167/200 [02:15<00:27,  1.18it/s]C:\Anaconda3\lib\site-packages\ipykernel\__main__.py:12: DeprecationWarning: 
.ix is deprecated. Please use
.loc for label based indexing or
.iloc for positional indexing

See the documentation here:
http://pandas.pydata.org/pandas-docs/stable/indexing.html#ix-indexer-is-deprecated
 84%|██████████  | 168/200 [02:16<00:26,  1.19it/s]C:\Anaconda3\lib\site-packages\ipykernel\__main__.py:12: DeprecationWarning: 
.ix is deprecated. Please use
.loc for label based indexing or
.iloc for positional indexing

See the documentation here:
http://pandas.pydata.org/pandas-docs/stable/indexing.html#ix-indexer-is-deprecated
 84%|██████████▏ | 169/200 [02:17<00:25,  1.20it/s]C:\Anaconda3\lib\site-packages\ipykernel\__main__.py:12: DeprecationWarning: 
.ix is deprecated. Please use
.loc for label based indexing or
.iloc for positional indexing

See the documentation here:
http://pandas.pydata.org/pandas-docs/stable/indexing.html#ix-indexer-is-deprecated
 85%|██████████▏ | 170/200 [02:18<00:25,  1.17it/s]C:\Anaconda3\lib\site-packages\ipykernel\__main__.py:12: DeprecationWarning: 
.ix is deprecated. Please use
.loc for label based indexing or
.iloc for positional indexing

See the documentation here:
http://pandas.pydata.org/pandas-docs/stable/indexing.html#ix-indexer-is-deprecated
 86%|██████████▎ | 171/200 [02:18<00:24,  1.19it/s]C:\Anaconda3\lib\site-packages\ipykernel\__main__.py:12: DeprecationWarning: 
.ix is deprecated. Please use
.loc for label based indexing or
.iloc for positional indexing

See the documentation here:
http://pandas.pydata.org/pandas-docs/stable/indexing.html#ix-indexer-is-deprecated
 86%|██████████▎ | 172/200 [02:19<00:24,  1.16it/s]C:\Anaconda3\lib\site-packages\ipykernel\__main__.py:12: DeprecationWarning: 
.ix is deprecated. Please use
.loc for label based indexing or
.iloc for positional indexing

See the documentation here:
http://pandas.pydata.org/pandas-docs/stable/indexing.html#ix-indexer-is-deprecated
 86%|██████████▍ | 173/200 [02:20<00:23,  1.17it/s]C:\Anaconda3\lib\site-packages\ipykernel\__main__.py:12: DeprecationWarning: 
.ix is deprecated. Please use
.loc for label based indexing or
.iloc for positional indexing

See the documentation here:
http://pandas.pydata.org/pandas-docs/stable/indexing.html#ix-indexer-is-deprecated
 87%|██████████▍ | 174/200 [02:21<00:21,  1.19it/s]C:\Anaconda3\lib\site-packages\ipykernel\__main__.py:12: DeprecationWarning: 
.ix is deprecated. Please use
.loc for label based indexing or
.iloc for positional indexing

See the documentation here:
http://pandas.pydata.org/pandas-docs/stable/indexing.html#ix-indexer-is-deprecated
 88%|██████████▌ | 175/200 [02:22<00:20,  1.21it/s]C:\Anaconda3\lib\site-packages\ipykernel\__main__.py:12: DeprecationWarning: 
.ix is deprecated. Please use
.loc for label based indexing or
.iloc for positional indexing

See the documentation here:
http://pandas.pydata.org/pandas-docs/stable/indexing.html#ix-indexer-is-deprecated
 88%|██████████▌ | 176/200 [02:23<00:19,  1.23it/s]C:\Anaconda3\lib\site-packages\ipykernel\__main__.py:12: DeprecationWarning: 
.ix is deprecated. Please use
.loc for label based indexing or
.iloc for positional indexing

See the documentation here:
http://pandas.pydata.org/pandas-docs/stable/indexing.html#ix-indexer-is-deprecated
 88%|██████████▌ | 177/200 [02:23<00:18,  1.24it/s]C:\Anaconda3\lib\site-packages\ipykernel\__main__.py:12: DeprecationWarning: 
.ix is deprecated. Please use
.loc for label based indexing or
.iloc for positional indexing

See the documentation here:
http://pandas.pydata.org/pandas-docs/stable/indexing.html#ix-indexer-is-deprecated
 89%|██████████▋ | 178/200 [02:24<00:17,  1.24it/s]C:\Anaconda3\lib\site-packages\ipykernel\__main__.py:12: DeprecationWarning: 
.ix is deprecated. Please use
.loc for label based indexing or
.iloc for positional indexing

See the documentation here:
http://pandas.pydata.org/pandas-docs/stable/indexing.html#ix-indexer-is-deprecated
 90%|██████████▋ | 179/200 [02:25<00:16,  1.25it/s]C:\Anaconda3\lib\site-packages\ipykernel\__main__.py:12: DeprecationWarning: 
.ix is deprecated. Please use
.loc for label based indexing or
.iloc for positional indexing

See the documentation here:
http://pandas.pydata.org/pandas-docs/stable/indexing.html#ix-indexer-is-deprecated
 90%|██████████▊ | 180/200 [02:26<00:17,  1.13it/s]C:\Anaconda3\lib\site-packages\ipykernel\__main__.py:12: DeprecationWarning: 
.ix is deprecated. Please use
.loc for label based indexing or
.iloc for positional indexing

See the documentation here:
http://pandas.pydata.org/pandas-docs/stable/indexing.html#ix-indexer-is-deprecated
 90%|██████████▊ | 181/200 [02:27<00:17,  1.10it/s]C:\Anaconda3\lib\site-packages\ipykernel\__main__.py:12: DeprecationWarning: 
.ix is deprecated. Please use
.loc for label based indexing or
.iloc for positional indexing

See the documentation here:
http://pandas.pydata.org/pandas-docs/stable/indexing.html#ix-indexer-is-deprecated
 91%|██████████▉ | 182/200 [02:28<00:16,  1.12it/s]C:\Anaconda3\lib\site-packages\ipykernel\__main__.py:12: DeprecationWarning: 
.ix is deprecated. Please use
.loc for label based indexing or
.iloc for positional indexing

See the documentation here:
http://pandas.pydata.org/pandas-docs/stable/indexing.html#ix-indexer-is-deprecated
 92%|██████████▉ | 183/200 [02:29<00:14,  1.15it/s]C:\Anaconda3\lib\site-packages\ipykernel\__main__.py:12: DeprecationWarning: 
.ix is deprecated. Please use
.loc for label based indexing or
.iloc for positional indexing

See the documentation here:
http://pandas.pydata.org/pandas-docs/stable/indexing.html#ix-indexer-is-deprecated
 92%|███████████ | 184/200 [02:30<00:13,  1.17it/s]C:\Anaconda3\lib\site-packages\ipykernel\__main__.py:12: DeprecationWarning: 
.ix is deprecated. Please use
.loc for label based indexing or
.iloc for positional indexing

See the documentation here:
http://pandas.pydata.org/pandas-docs/stable/indexing.html#ix-indexer-is-deprecated
 92%|███████████ | 185/200 [02:30<00:12,  1.17it/s]C:\Anaconda3\lib\site-packages\ipykernel\__main__.py:12: DeprecationWarning: 
.ix is deprecated. Please use
.loc for label based indexing or
.iloc for positional indexing

See the documentation here:
http://pandas.pydata.org/pandas-docs/stable/indexing.html#ix-indexer-is-deprecated
 93%|███████████▏| 186/200 [02:31<00:11,  1.20it/s]C:\Anaconda3\lib\site-packages\ipykernel\__main__.py:12: DeprecationWarning: 
.ix is deprecated. Please use
.loc for label based indexing or
.iloc for positional indexing

See the documentation here:
http://pandas.pydata.org/pandas-docs/stable/indexing.html#ix-indexer-is-deprecated
 94%|███████████▏| 187/200 [02:32<00:10,  1.21it/s]C:\Anaconda3\lib\site-packages\ipykernel\__main__.py:12: DeprecationWarning: 
.ix is deprecated. Please use
.loc for label based indexing or
.iloc for positional indexing

See the documentation here:
http://pandas.pydata.org/pandas-docs/stable/indexing.html#ix-indexer-is-deprecated
 94%|███████████▎| 188/200 [02:33<00:09,  1.22it/s]C:\Anaconda3\lib\site-packages\ipykernel\__main__.py:12: DeprecationWarning: 
.ix is deprecated. Please use
.loc for label based indexing or
.iloc for positional indexing

See the documentation here:
http://pandas.pydata.org/pandas-docs/stable/indexing.html#ix-indexer-is-deprecated
 94%|███████████▎| 189/200 [02:34<00:09,  1.22it/s]C:\Anaconda3\lib\site-packages\ipykernel\__main__.py:12: DeprecationWarning: 
.ix is deprecated. Please use
.loc for label based indexing or
.iloc for positional indexing

See the documentation here:
http://pandas.pydata.org/pandas-docs/stable/indexing.html#ix-indexer-is-deprecated
 95%|███████████▍| 190/200 [02:34<00:08,  1.23it/s]C:\Anaconda3\lib\site-packages\ipykernel\__main__.py:12: DeprecationWarning: 
.ix is deprecated. Please use
.loc for label based indexing or
.iloc for positional indexing

See the documentation here:
http://pandas.pydata.org/pandas-docs/stable/indexing.html#ix-indexer-is-deprecated
 96%|███████████▍| 191/200 [02:35<00:07,  1.24it/s]C:\Anaconda3\lib\site-packages\ipykernel\__main__.py:12: DeprecationWarning: 
.ix is deprecated. Please use
.loc for label based indexing or
.iloc for positional indexing

See the documentation here:
http://pandas.pydata.org/pandas-docs/stable/indexing.html#ix-indexer-is-deprecated
 96%|███████████▌| 192/200 [02:36<00:06,  1.23it/s]C:\Anaconda3\lib\site-packages\ipykernel\__main__.py:12: DeprecationWarning: 
.ix is deprecated. Please use
.loc for label based indexing or
.iloc for positional indexing

See the documentation here:
http://pandas.pydata.org/pandas-docs/stable/indexing.html#ix-indexer-is-deprecated
 96%|███████████▌| 193/200 [02:37<00:05,  1.21it/s]C:\Anaconda3\lib\site-packages\ipykernel\__main__.py:12: DeprecationWarning: 
.ix is deprecated. Please use
.loc for label based indexing or
.iloc for positional indexing

See the documentation here:
http://pandas.pydata.org/pandas-docs/stable/indexing.html#ix-indexer-is-deprecated
 97%|███████████▋| 194/200 [02:38<00:05,  1.16it/s]C:\Anaconda3\lib\site-packages\ipykernel\__main__.py:12: DeprecationWarning: 
.ix is deprecated. Please use
.loc for label based indexing or
.iloc for positional indexing

See the documentation here:
http://pandas.pydata.org/pandas-docs/stable/indexing.html#ix-indexer-is-deprecated
 98%|███████████▋| 195/200 [02:39<00:04,  1.17it/s]C:\Anaconda3\lib\site-packages\ipykernel\__main__.py:12: DeprecationWarning: 
.ix is deprecated. Please use
.loc for label based indexing or
.iloc for positional indexing

See the documentation here:
http://pandas.pydata.org/pandas-docs/stable/indexing.html#ix-indexer-is-deprecated
 98%|███████████▊| 196/200 [02:39<00:03,  1.19it/s]C:\Anaconda3\lib\site-packages\ipykernel\__main__.py:12: DeprecationWarning: 
.ix is deprecated. Please use
.loc for label based indexing or
.iloc for positional indexing

See the documentation here:
http://pandas.pydata.org/pandas-docs/stable/indexing.html#ix-indexer-is-deprecated
 98%|███████████▊| 197/200 [02:40<00:02,  1.21it/s]C:\Anaconda3\lib\site-packages\ipykernel\__main__.py:12: DeprecationWarning: 
.ix is deprecated. Please use
.loc for label based indexing or
.iloc for positional indexing

See the documentation here:
http://pandas.pydata.org/pandas-docs/stable/indexing.html#ix-indexer-is-deprecated
 99%|███████████▉| 198/200 [02:41<00:01,  1.22it/s]C:\Anaconda3\lib\site-packages\ipykernel\__main__.py:12: DeprecationWarning: 
.ix is deprecated. Please use
.loc for label based indexing or
.iloc for positional indexing

See the documentation here:
http://pandas.pydata.org/pandas-docs/stable/indexing.html#ix-indexer-is-deprecated
100%|███████████▉| 199/200 [02:42<00:00,  1.22it/s]C:\Anaconda3\lib\site-packages\ipykernel\__main__.py:12: DeprecationWarning: 
.ix is deprecated. Please use
.loc for label based indexing or
.iloc for positional indexing

See the documentation here:
http://pandas.pydata.org/pandas-docs/stable/indexing.html#ix-indexer-is-deprecated
100%|████████████| 200/200 [02:43<00:00,  1.22it/s]
```

```
평균적인 threshold, 0.46 (0.05) [0.38-0.56]
평균적인 auc, 0.81 (0.05) [0.71-0.91]
평균적인 sensitivity, 0.74 (0.07) [0.61-0.87]
평균적인 specificity, 0.74 (0.06) [0.63-0.85]
평균적인 ppv, 0.71 (0.06) [0.6-0.83]
평균적인 npv, 0.77 (0.05) [0.67-0.88]
평균적인 accuracy, 0.74 (0.05) [0.64-0.84]
----------------------------------------------------------------------------------
AdaBoostClassifier(algorithm='SAMME.R', base_estimator=None,
          learning_rate=1.0, n_estimators=50, random_state=None)
```

```
  0%|                      | 0/200 [00:00<?, ?it/s]C:\Anaconda3\lib\site-packages\ipykernel\__main__.py:12: DeprecationWarning: 
.ix is deprecated. Please use
.loc for label based indexing or
.iloc for positional indexing

See the documentation here:
http://pandas.pydata.org/pandas-docs/stable/indexing.html#ix-indexer-is-deprecated
C:\Anaconda3\lib\site-packages\ipykernel\__main__.py:12: DeprecationWarning: 
.ix is deprecated. Please use
.loc for label based indexing or
.iloc for positional indexing

See the documentation here:
http://pandas.pydata.org/pandas-docs/stable/indexing.html#ix-indexer-is-deprecated
  1%|▏             | 2/200 [00:00<00:19,  9.90it/s]C:\Anaconda3\lib\site-packages\ipykernel\__main__.py:12: DeprecationWarning: 
.ix is deprecated. Please use
.loc for label based indexing or
.iloc for positional indexing

See the documentation here:
http://pandas.pydata.org/pandas-docs/stable/indexing.html#ix-indexer-is-deprecated
C:\Anaconda3\lib\site-packages\ipykernel\__main__.py:12: DeprecationWarning: 
.ix is deprecated. Please use
.loc for label based indexing or
.iloc for positional indexing

See the documentation here:
http://pandas.pydata.org/pandas-docs/stable/indexing.html#ix-indexer-is-deprecated
  2%|▎             | 4/200 [00:00<00:19, 10.13it/s]C:\Anaconda3\lib\site-packages\ipykernel\__main__.py:12: DeprecationWarning: 
.ix is deprecated. Please use
.loc for label based indexing or
.iloc for positional indexing

See the documentation here:
http://pandas.pydata.org/pandas-docs/stable/indexing.html#ix-indexer-is-deprecated
C:\Anaconda3\lib\site-packages\ipykernel\__main__.py:12: DeprecationWarning: 
.ix is deprecated. Please use
.loc for label based indexing or
.iloc for positional indexing

See the documentation here:
http://pandas.pydata.org/pandas-docs/stable/indexing.html#ix-indexer-is-deprecated
  3%|▍             | 6/200 [00:00<00:18, 10.22it/s]C:\Anaconda3\lib\site-packages\ipykernel\__main__.py:12: DeprecationWarning: 
.ix is deprecated. Please use
.loc for label based indexing or
.iloc for positional indexing

See the documentation here:
http://pandas.pydata.org/pandas-docs/stable/indexing.html#ix-indexer-is-deprecated
C:\Anaconda3\lib\site-packages\ipykernel\__main__.py:12: DeprecationWarning: 
.ix is deprecated. Please use
.loc for label based indexing or
.iloc for positional indexing

See the documentation here:
http://pandas.pydata.org/pandas-docs/stable/indexing.html#ix-indexer-is-deprecated
  4%|▌             | 8/200 [00:00<00:18, 10.20it/s]C:\Anaconda3\lib\site-packages\ipykernel\__main__.py:12: DeprecationWarning: 
.ix is deprecated. Please use
.loc for label based indexing or
.iloc for positional indexing

See the documentation here:
http://pandas.pydata.org/pandas-docs/stable/indexing.html#ix-indexer-is-deprecated
  4%|▋             | 9/200 [00:00<00:21,  9.08it/s]C:\Anaconda3\lib\site-packages\ipykernel\__main__.py:12: DeprecationWarning: 
.ix is deprecated. Please use
.loc for label based indexing or
.iloc for positional indexing

See the documentation here:
http://pandas.pydata.org/pandas-docs/stable/indexing.html#ix-indexer-is-deprecated
C:\Anaconda3\lib\site-packages\ipykernel\__main__.py:12: DeprecationWarning: 
.ix is deprecated. Please use
.loc for label based indexing or
.iloc for positional indexing

See the documentation here:
http://pandas.pydata.org/pandas-docs/stable/indexing.html#ix-indexer-is-deprecated
  6%|▋            | 11/200 [00:01<00:19,  9.50it/s]C:\Anaconda3\lib\site-packages\ipykernel\__main__.py:12: DeprecationWarning: 
.ix is deprecated. Please use
.loc for label based indexing or
.iloc for positional indexing

See the documentation here:
http://pandas.pydata.org/pandas-docs/stable/indexing.html#ix-indexer-is-deprecated
  6%|▊            | 12/200 [00:01<00:20,  9.23it/s]C:\Anaconda3\lib\site-packages\ipykernel\__main__.py:12: DeprecationWarning: 
.ix is deprecated. Please use
.loc for label based indexing or
.iloc for positional indexing

See the documentation here:
http://pandas.pydata.org/pandas-docs/stable/indexing.html#ix-indexer-is-deprecated
  6%|▊            | 13/200 [00:01<00:20,  9.27it/s]C:\Anaconda3\lib\site-packages\ipykernel\__main__.py:12: DeprecationWarning: 
.ix is deprecated. Please use
.loc for label based indexing or
.iloc for positional indexing

See the documentation here:
http://pandas.pydata.org/pandas-docs/stable/indexing.html#ix-indexer-is-deprecated
  7%|▉            | 14/200 [00:01<00:21,  8.85it/s]C:\Anaconda3\lib\site-packages\ipykernel\__main__.py:12: DeprecationWarning: 
.ix is deprecated. Please use
.loc for label based indexing or
.iloc for positional indexing

See the documentation here:
http://pandas.pydata.org/pandas-docs/stable/indexing.html#ix-indexer-is-deprecated
  8%|▉            | 15/200 [00:01<00:22,  8.28it/s]C:\Anaconda3\lib\site-packages\ipykernel\__main__.py:12: DeprecationWarning: 
.ix is deprecated. Please use
.loc for label based indexing or
.iloc for positional indexing

See the documentation here:
http://pandas.pydata.org/pandas-docs/stable/indexing.html#ix-indexer-is-deprecated
  8%|█            | 16/200 [00:01<00:21,  8.38it/s]C:\Anaconda3\lib\site-packages\ipykernel\__main__.py:12: DeprecationWarning: 
.ix is deprecated. Please use
.loc for label based indexing or
.iloc for positional indexing

See the documentation here:
http://pandas.pydata.org/pandas-docs/stable/indexing.html#ix-indexer-is-deprecated
  8%|█            | 17/200 [00:01<00:22,  8.22it/s]C:\Anaconda3\lib\site-packages\ipykernel\__main__.py:12: DeprecationWarning: 
.ix is deprecated. Please use
.loc for label based indexing or
.iloc for positional indexing

See the documentation here:
http://pandas.pydata.org/pandas-docs/stable/indexing.html#ix-indexer-is-deprecated
  9%|█▏           | 18/200 [00:01<00:21,  8.31it/s]C:\Anaconda3\lib\site-packages\ipykernel\__main__.py:12: DeprecationWarning: 
.ix is deprecated. Please use
.loc for label based indexing or
.iloc for positional indexing

See the documentation here:
http://pandas.pydata.org/pandas-docs/stable/indexing.html#ix-indexer-is-deprecated
 10%|█▏           | 19/200 [00:02<00:21,  8.61it/s]C:\Anaconda3\lib\site-packages\ipykernel\__main__.py:12: DeprecationWarning: 
.ix is deprecated. Please use
.loc for label based indexing or
.iloc for positional indexing

See the documentation here:
http://pandas.pydata.org/pandas-docs/stable/indexing.html#ix-indexer-is-deprecated
 10%|█▎           | 20/200 [00:02<00:20,  8.82it/s]C:\Anaconda3\lib\site-packages\ipykernel\__main__.py:12: DeprecationWarning: 
.ix is deprecated. Please use
.loc for label based indexing or
.iloc for positional indexing

See the documentation here:
http://pandas.pydata.org/pandas-docs/stable/indexing.html#ix-indexer-is-deprecated
 10%|█▎           | 21/200 [00:02<00:20,  8.74it/s]C:\Anaconda3\lib\site-packages\ipykernel\__main__.py:12: DeprecationWarning: 
.ix is deprecated. Please use
.loc for label based indexing or
.iloc for positional indexing

See the documentation here:
http://pandas.pydata.org/pandas-docs/stable/indexing.html#ix-indexer-is-deprecated
 11%|█▍           | 22/200 [00:02<00:21,  8.47it/s]C:\Anaconda3\lib\site-packages\ipykernel\__main__.py:12: DeprecationWarning: 
.ix is deprecated. Please use
.loc for label based indexing or
.iloc for positional indexing

See the documentation here:
http://pandas.pydata.org/pandas-docs/stable/indexing.html#ix-indexer-is-deprecated
 12%|█▍           | 23/200 [00:02<00:20,  8.71it/s]C:\Anaconda3\lib\site-packages\ipykernel\__main__.py:12: DeprecationWarning: 
.ix is deprecated. Please use
.loc for label based indexing or
.iloc for positional indexing

See the documentation here:
http://pandas.pydata.org/pandas-docs/stable/indexing.html#ix-indexer-is-deprecated
 12%|█▌           | 24/200 [00:02<00:19,  8.96it/s]C:\Anaconda3\lib\site-packages\ipykernel\__main__.py:12: DeprecationWarning: 
.ix is deprecated. Please use
.loc for label based indexing or
.iloc for positional indexing

See the documentation here:
http://pandas.pydata.org/pandas-docs/stable/indexing.html#ix-indexer-is-deprecated
C:\Anaconda3\lib\site-packages\ipykernel\__main__.py:12: DeprecationWarning: 
.ix is deprecated. Please use
.loc for label based indexing or
.iloc for positional indexing

See the documentation here:
http://pandas.pydata.org/pandas-docs/stable/indexing.html#ix-indexer-is-deprecated
 13%|█▋           | 26/200 [00:02<00:18,  9.48it/s]C:\Anaconda3\lib\site-packages\ipykernel\__main__.py:12: DeprecationWarning: 
.ix is deprecated. Please use
.loc for label based indexing or
.iloc for positional indexing

See the documentation here:
http://pandas.pydata.org/pandas-docs/stable/indexing.html#ix-indexer-is-deprecated
C:\Anaconda3\lib\site-packages\ipykernel\__main__.py:12: DeprecationWarning: 
.ix is deprecated. Please use
.loc for label based indexing or
.iloc for positional indexing

See the documentation here:
http://pandas.pydata.org/pandas-docs/stable/indexing.html#ix-indexer-is-deprecated
 14%|█▊           | 28/200 [00:02<00:17,  9.87it/s]C:\Anaconda3\lib\site-packages\ipykernel\__main__.py:12: DeprecationWarning: 
.ix is deprecated. Please use
.loc for label based indexing or
.iloc for positional indexing

See the documentation here:
http://pandas.pydata.org/pandas-docs/stable/indexing.html#ix-indexer-is-deprecated
 14%|█▉           | 29/200 [00:03<00:17,  9.91it/s]C:\Anaconda3\lib\site-packages\ipykernel\__main__.py:12: DeprecationWarning: 
.ix is deprecated. Please use
.loc for label based indexing or
.iloc for positional indexing

See the documentation here:
http://pandas.pydata.org/pandas-docs/stable/indexing.html#ix-indexer-is-deprecated
 15%|█▉           | 30/200 [00:03<00:17,  9.91it/s]C:\Anaconda3\lib\site-packages\ipykernel\__main__.py:12: DeprecationWarning: 
.ix is deprecated. Please use
.loc for label based indexing or
.iloc for positional indexing

See the documentation here:
http://pandas.pydata.org/pandas-docs/stable/indexing.html#ix-indexer-is-deprecated
 16%|██           | 31/200 [00:03<00:17,  9.73it/s]C:\Anaconda3\lib\site-packages\ipykernel\__main__.py:12: DeprecationWarning: 
.ix is deprecated. Please use
.loc for label based indexing or
.iloc for positional indexing

See the documentation here:
http://pandas.pydata.org/pandas-docs/stable/indexing.html#ix-indexer-is-deprecated
C:\Anaconda3\lib\site-packages\ipykernel\__main__.py:12: DeprecationWarning: 
.ix is deprecated. Please use
.loc for label based indexing or
.iloc for positional indexing

See the documentation here:
http://pandas.pydata.org/pandas-docs/stable/indexing.html#ix-indexer-is-deprecated
 16%|██▏          | 33/200 [00:03<00:17,  9.73it/s]C:\Anaconda3\lib\site-packages\ipykernel\__main__.py:12: DeprecationWarning: 
.ix is deprecated. Please use
.loc for label based indexing or
.iloc for positional indexing

See the documentation here:
http://pandas.pydata.org/pandas-docs/stable/indexing.html#ix-indexer-is-deprecated
C:\Anaconda3\lib\site-packages\ipykernel\__main__.py:12: DeprecationWarning: 
.ix is deprecated. Please use
.loc for label based indexing or
.iloc for positional indexing

See the documentation here:
http://pandas.pydata.org/pandas-docs/stable/indexing.html#ix-indexer-is-deprecated
 18%|██▎          | 35/200 [00:03<00:16,  9.75it/s]C:\Anaconda3\lib\site-packages\ipykernel\__main__.py:12: DeprecationWarning: 
.ix is deprecated. Please use
.loc for label based indexing or
.iloc for positional indexing

See the documentation here:
http://pandas.pydata.org/pandas-docs/stable/indexing.html#ix-indexer-is-deprecated
 18%|██▎          | 36/200 [00:03<00:16,  9.77it/s]C:\Anaconda3\lib\site-packages\ipykernel\__main__.py:12: DeprecationWarning: 
.ix is deprecated. Please use
.loc for label based indexing or
.iloc for positional indexing

See the documentation here:
http://pandas.pydata.org/pandas-docs/stable/indexing.html#ix-indexer-is-deprecated
 18%|██▍          | 37/200 [00:03<00:16,  9.72it/s]C:\Anaconda3\lib\site-packages\ipykernel\__main__.py:12: DeprecationWarning: 
.ix is deprecated. Please use
.loc for label based indexing or
.iloc for positional indexing

See the documentation here:
http://pandas.pydata.org/pandas-docs/stable/indexing.html#ix-indexer-is-deprecated
 19%|██▍          | 38/200 [00:04<00:16,  9.72it/s]C:\Anaconda3\lib\site-packages\ipykernel\__main__.py:12: DeprecationWarning: 
.ix is deprecated. Please use
.loc for label based indexing or
.iloc for positional indexing

See the documentation here:
http://pandas.pydata.org/pandas-docs/stable/indexing.html#ix-indexer-is-deprecated
C:\Anaconda3\lib\site-packages\ipykernel\__main__.py:12: DeprecationWarning: 
.ix is deprecated. Please use
.loc for label based indexing or
.iloc for positional indexing

See the documentation here:
http://pandas.pydata.org/pandas-docs/stable/indexing.html#ix-indexer-is-deprecated
 20%|██▌          | 40/200 [00:04<00:16,  9.96it/s]C:\Anaconda3\lib\site-packages\ipykernel\__main__.py:12: DeprecationWarning: 
.ix is deprecated. Please use
.loc for label based indexing or
.iloc for positional indexing

See the documentation here:
http://pandas.pydata.org/pandas-docs/stable/indexing.html#ix-indexer-is-deprecated
C:\Anaconda3\lib\site-packages\ipykernel\__main__.py:12: DeprecationWarning: 
.ix is deprecated. Please use
.loc for label based indexing or
.iloc for positional indexing

See the documentation here:
http://pandas.pydata.org/pandas-docs/stable/indexing.html#ix-indexer-is-deprecated
 21%|██▋          | 42/200 [00:04<00:15, 10.18it/s]C:\Anaconda3\lib\site-packages\ipykernel\__main__.py:12: DeprecationWarning: 
.ix is deprecated. Please use
.loc for label based indexing or
.iloc for positional indexing

See the documentation here:
http://pandas.pydata.org/pandas-docs/stable/indexing.html#ix-indexer-is-deprecated
C:\Anaconda3\lib\site-packages\ipykernel\__main__.py:12: DeprecationWarning: 
.ix is deprecated. Please use
.loc for label based indexing or
.iloc for positional indexing

See the documentation here:
http://pandas.pydata.org/pandas-docs/stable/indexing.html#ix-indexer-is-deprecated
 22%|██▊          | 44/200 [00:04<00:15, 10.36it/s]C:\Anaconda3\lib\site-packages\ipykernel\__main__.py:12: DeprecationWarning: 
.ix is deprecated. Please use
.loc for label based indexing or
.iloc for positional indexing

See the documentation here:
http://pandas.pydata.org/pandas-docs/stable/indexing.html#ix-indexer-is-deprecated
C:\Anaconda3\lib\site-packages\ipykernel\__main__.py:12: DeprecationWarning: 
.ix is deprecated. Please use
.loc for label based indexing or
.iloc for positional indexing

See the documentation here:
http://pandas.pydata.org/pandas-docs/stable/indexing.html#ix-indexer-is-deprecated
 23%|██▉          | 46/200 [00:04<00:14, 10.41it/s]C:\Anaconda3\lib\site-packages\ipykernel\__main__.py:12: DeprecationWarning: 
.ix is deprecated. Please use
.loc for label based indexing or
.iloc for positional indexing

See the documentation here:
http://pandas.pydata.org/pandas-docs/stable/indexing.html#ix-indexer-is-deprecated
C:\Anaconda3\lib\site-packages\ipykernel\__main__.py:12: DeprecationWarning: 
.ix is deprecated. Please use
.loc for label based indexing or
.iloc for positional indexing

See the documentation here:
http://pandas.pydata.org/pandas-docs/stable/indexing.html#ix-indexer-is-deprecated
 24%|███          | 48/200 [00:04<00:14, 10.60it/s]C:\Anaconda3\lib\site-packages\ipykernel\__main__.py:12: DeprecationWarning: 
.ix is deprecated. Please use
.loc for label based indexing or
.iloc for positional indexing

See the documentation here:
http://pandas.pydata.org/pandas-docs/stable/indexing.html#ix-indexer-is-deprecated
C:\Anaconda3\lib\site-packages\ipykernel\__main__.py:12: DeprecationWarning: 
.ix is deprecated. Please use
.loc for label based indexing or
.iloc for positional indexing

See the documentation here:
http://pandas.pydata.org/pandas-docs/stable/indexing.html#ix-indexer-is-deprecated
 25%|███▎         | 50/200 [00:05<00:14, 10.28it/s]C:\Anaconda3\lib\site-packages\ipykernel\__main__.py:12: DeprecationWarning: 
.ix is deprecated. Please use
.loc for label based indexing or
.iloc for positional indexing

See the documentation here:
http://pandas.pydata.org/pandas-docs/stable/indexing.html#ix-indexer-is-deprecated
C:\Anaconda3\lib\site-packages\ipykernel\__main__.py:12: DeprecationWarning: 
.ix is deprecated. Please use
.loc for label based indexing or
.iloc for positional indexing

See the documentation here:
http://pandas.pydata.org/pandas-docs/stable/indexing.html#ix-indexer-is-deprecated
 26%|███▍         | 52/200 [00:05<00:14, 10.19it/s]C:\Anaconda3\lib\site-packages\ipykernel\__main__.py:12: DeprecationWarning: 
.ix is deprecated. Please use
.loc for label based indexing or
.iloc for positional indexing

See the documentation here:
http://pandas.pydata.org/pandas-docs/stable/indexing.html#ix-indexer-is-deprecated
C:\Anaconda3\lib\site-packages\ipykernel\__main__.py:12: DeprecationWarning: 
.ix is deprecated. Please use
.loc for label based indexing or
.iloc for positional indexing

See the documentation here:
http://pandas.pydata.org/pandas-docs/stable/indexing.html#ix-indexer-is-deprecated
 27%|███▌         | 54/200 [00:05<00:14, 10.14it/s]C:\Anaconda3\lib\site-packages\ipykernel\__main__.py:12: DeprecationWarning: 
.ix is deprecated. Please use
.loc for label based indexing or
.iloc for positional indexing

See the documentation here:
http://pandas.pydata.org/pandas-docs/stable/indexing.html#ix-indexer-is-deprecated
C:\Anaconda3\lib\site-packages\ipykernel\__main__.py:12: DeprecationWarning: 
.ix is deprecated. Please use
.loc for label based indexing or
.iloc for positional indexing

See the documentation here:
http://pandas.pydata.org/pandas-docs/stable/indexing.html#ix-indexer-is-deprecated
 28%|███▋         | 56/200 [00:05<00:14, 10.25it/s]C:\Anaconda3\lib\site-packages\ipykernel\__main__.py:12: DeprecationWarning: 
.ix is deprecated. Please use
.loc for label based indexing or
.iloc for positional indexing

See the documentation here:
http://pandas.pydata.org/pandas-docs/stable/indexing.html#ix-indexer-is-deprecated
C:\Anaconda3\lib\site-packages\ipykernel\__main__.py:12: DeprecationWarning: 
.ix is deprecated. Please use
.loc for label based indexing or
.iloc for positional indexing

See the documentation here:
http://pandas.pydata.org/pandas-docs/stable/indexing.html#ix-indexer-is-deprecated
 29%|███▊         | 58/200 [00:05<00:13, 10.53it/s]C:\Anaconda3\lib\site-packages\ipykernel\__main__.py:12: DeprecationWarning: 
.ix is deprecated. Please use
.loc for label based indexing or
.iloc for positional indexing

See the documentation here:
http://pandas.pydata.org/pandas-docs/stable/indexing.html#ix-indexer-is-deprecated
C:\Anaconda3\lib\site-packages\ipykernel\__main__.py:12: DeprecationWarning: 
.ix is deprecated. Please use
.loc for label based indexing or
.iloc for positional indexing

See the documentation here:
http://pandas.pydata.org/pandas-docs/stable/indexing.html#ix-indexer-is-deprecated
 30%|███▉         | 60/200 [00:06<00:13, 10.36it/s]C:\Anaconda3\lib\site-packages\ipykernel\__main__.py:12: DeprecationWarning: 
.ix is deprecated. Please use
.loc for label based indexing or
.iloc for positional indexing

See the documentation here:
http://pandas.pydata.org/pandas-docs/stable/indexing.html#ix-indexer-is-deprecated
C:\Anaconda3\lib\site-packages\ipykernel\__main__.py:12: DeprecationWarning: 
.ix is deprecated. Please use
.loc for label based indexing or
.iloc for positional indexing

See the documentation here:
http://pandas.pydata.org/pandas-docs/stable/indexing.html#ix-indexer-is-deprecated
 31%|████         | 62/200 [00:06<00:13, 10.46it/s]C:\Anaconda3\lib\site-packages\ipykernel\__main__.py:12: DeprecationWarning: 
.ix is deprecated. Please use
.loc for label based indexing or
.iloc for positional indexing

See the documentation here:
http://pandas.pydata.org/pandas-docs/stable/indexing.html#ix-indexer-is-deprecated
C:\Anaconda3\lib\site-packages\ipykernel\__main__.py:12: DeprecationWarning: 
.ix is deprecated. Please use
.loc for label based indexing or
.iloc for positional indexing

See the documentation here:
http://pandas.pydata.org/pandas-docs/stable/indexing.html#ix-indexer-is-deprecated
 32%|████▏        | 64/200 [00:06<00:12, 10.64it/s]C:\Anaconda3\lib\site-packages\ipykernel\__main__.py:12: DeprecationWarning: 
.ix is deprecated. Please use
.loc for label based indexing or
.iloc for positional indexing

See the documentation here:
http://pandas.pydata.org/pandas-docs/stable/indexing.html#ix-indexer-is-deprecated
C:\Anaconda3\lib\site-packages\ipykernel\__main__.py:12: DeprecationWarning: 
.ix is deprecated. Please use
.loc for label based indexing or
.iloc for positional indexing

See the documentation here:
http://pandas.pydata.org/pandas-docs/stable/indexing.html#ix-indexer-is-deprecated
 33%|████▎        | 66/200 [00:06<00:12, 10.34it/s]C:\Anaconda3\lib\site-packages\ipykernel\__main__.py:12: DeprecationWarning: 
.ix is deprecated. Please use
.loc for label based indexing or
.iloc for positional indexing

See the documentation here:
http://pandas.pydata.org/pandas-docs/stable/indexing.html#ix-indexer-is-deprecated
C:\Anaconda3\lib\site-packages\ipykernel\__main__.py:12: DeprecationWarning: 
.ix is deprecated. Please use
.loc for label based indexing or
.iloc for positional indexing

See the documentation here:
http://pandas.pydata.org/pandas-docs/stable/indexing.html#ix-indexer-is-deprecated
 34%|████▍        | 68/200 [00:06<00:12, 10.51it/s]C:\Anaconda3\lib\site-packages\ipykernel\__main__.py:12: DeprecationWarning: 
.ix is deprecated. Please use
.loc for label based indexing or
.iloc for positional indexing

See the documentation here:
http://pandas.pydata.org/pandas-docs/stable/indexing.html#ix-indexer-is-deprecated
C:\Anaconda3\lib\site-packages\ipykernel\__main__.py:12: DeprecationWarning: 
.ix is deprecated. Please use
.loc for label based indexing or
.iloc for positional indexing

See the documentation here:
http://pandas.pydata.org/pandas-docs/stable/indexing.html#ix-indexer-is-deprecated
 35%|████▌        | 70/200 [00:07<00:12, 10.50it/s]C:\Anaconda3\lib\site-packages\ipykernel\__main__.py:12: DeprecationWarning: 
.ix is deprecated. Please use
.loc for label based indexing or
.iloc for positional indexing

See the documentation here:
http://pandas.pydata.org/pandas-docs/stable/indexing.html#ix-indexer-is-deprecated
C:\Anaconda3\lib\site-packages\ipykernel\__main__.py:12: DeprecationWarning: 
.ix is deprecated. Please use
.loc for label based indexing or
.iloc for positional indexing

See the documentation here:
http://pandas.pydata.org/pandas-docs/stable/indexing.html#ix-indexer-is-deprecated
 36%|████▋        | 72/200 [00:07<00:12, 10.14it/s]C:\Anaconda3\lib\site-packages\ipykernel\__main__.py:12: DeprecationWarning: 
.ix is deprecated. Please use
.loc for label based indexing or
.iloc for positional indexing

See the documentation here:
http://pandas.pydata.org/pandas-docs/stable/indexing.html#ix-indexer-is-deprecated
C:\Anaconda3\lib\site-packages\ipykernel\__main__.py:12: DeprecationWarning: 
.ix is deprecated. Please use
.loc for label based indexing or
.iloc for positional indexing

See the documentation here:
http://pandas.pydata.org/pandas-docs/stable/indexing.html#ix-indexer-is-deprecated
 37%|████▊        | 74/200 [00:07<00:12, 10.27it/s]C:\Anaconda3\lib\site-packages\ipykernel\__main__.py:12: DeprecationWarning: 
.ix is deprecated. Please use
.loc for label based indexing or
.iloc for positional indexing

See the documentation here:
http://pandas.pydata.org/pandas-docs/stable/indexing.html#ix-indexer-is-deprecated
C:\Anaconda3\lib\site-packages\ipykernel\__main__.py:12: DeprecationWarning: 
.ix is deprecated. Please use
.loc for label based indexing or
.iloc for positional indexing

See the documentation here:
http://pandas.pydata.org/pandas-docs/stable/indexing.html#ix-indexer-is-deprecated
 38%|████▉        | 76/200 [00:07<00:12, 10.19it/s]C:\Anaconda3\lib\site-packages\ipykernel\__main__.py:12: DeprecationWarning: 
.ix is deprecated. Please use
.loc for label based indexing or
.iloc for positional indexing

See the documentation here:
http://pandas.pydata.org/pandas-docs/stable/indexing.html#ix-indexer-is-deprecated
C:\Anaconda3\lib\site-packages\ipykernel\__main__.py:12: DeprecationWarning: 
.ix is deprecated. Please use
.loc for label based indexing or
.iloc for positional indexing

See the documentation here:
http://pandas.pydata.org/pandas-docs/stable/indexing.html#ix-indexer-is-deprecated
 39%|█████        | 78/200 [00:07<00:11, 10.29it/s]C:\Anaconda3\lib\site-packages\ipykernel\__main__.py:12: DeprecationWarning: 
.ix is deprecated. Please use
.loc for label based indexing or
.iloc for positional indexing

See the documentation here:
http://pandas.pydata.org/pandas-docs/stable/indexing.html#ix-indexer-is-deprecated
C:\Anaconda3\lib\site-packages\ipykernel\__main__.py:12: DeprecationWarning: 
.ix is deprecated. Please use
.loc for label based indexing or
.iloc for positional indexing

See the documentation here:
http://pandas.pydata.org/pandas-docs/stable/indexing.html#ix-indexer-is-deprecated
 40%|█████▏       | 80/200 [00:08<00:11, 10.61it/s]C:\Anaconda3\lib\site-packages\ipykernel\__main__.py:12: DeprecationWarning: 
.ix is deprecated. Please use
.loc for label based indexing or
.iloc for positional indexing

See the documentation here:
http://pandas.pydata.org/pandas-docs/stable/indexing.html#ix-indexer-is-deprecated
C:\Anaconda3\lib\site-packages\ipykernel\__main__.py:12: DeprecationWarning: 
.ix is deprecated. Please use
.loc for label based indexing or
.iloc for positional indexing

See the documentation here:
http://pandas.pydata.org/pandas-docs/stable/indexing.html#ix-indexer-is-deprecated
 41%|█████▎       | 82/200 [00:08<00:11, 10.48it/s]C:\Anaconda3\lib\site-packages\ipykernel\__main__.py:12: DeprecationWarning: 
.ix is deprecated. Please use
.loc for label based indexing or
.iloc for positional indexing

See the documentation here:
http://pandas.pydata.org/pandas-docs/stable/indexing.html#ix-indexer-is-deprecated
C:\Anaconda3\lib\site-packages\ipykernel\__main__.py:12: DeprecationWarning: 
.ix is deprecated. Please use
.loc for label based indexing or
.iloc for positional indexing

See the documentation here:
http://pandas.pydata.org/pandas-docs/stable/indexing.html#ix-indexer-is-deprecated
 42%|█████▍       | 84/200 [00:08<00:11, 10.27it/s]C:\Anaconda3\lib\site-packages\ipykernel\__main__.py:12: DeprecationWarning: 
.ix is deprecated. Please use
.loc for label based indexing or
.iloc for positional indexing

See the documentation here:
http://pandas.pydata.org/pandas-docs/stable/indexing.html#ix-indexer-is-deprecated
C:\Anaconda3\lib\site-packages\ipykernel\__main__.py:12: DeprecationWarning: 
.ix is deprecated. Please use
.loc for label based indexing or
.iloc for positional indexing

See the documentation here:
http://pandas.pydata.org/pandas-docs/stable/indexing.html#ix-indexer-is-deprecated
 43%|█████▌       | 86/200 [00:08<00:11, 10.25it/s]C:\Anaconda3\lib\site-packages\ipykernel\__main__.py:12: DeprecationWarning: 
.ix is deprecated. Please use
.loc for label based indexing or
.iloc for positional indexing

See the documentation here:
http://pandas.pydata.org/pandas-docs/stable/indexing.html#ix-indexer-is-deprecated
C:\Anaconda3\lib\site-packages\ipykernel\__main__.py:12: DeprecationWarning: 
.ix is deprecated. Please use
.loc for label based indexing or
.iloc for positional indexing

See the documentation here:
http://pandas.pydata.org/pandas-docs/stable/indexing.html#ix-indexer-is-deprecated
 44%|█████▋       | 88/200 [00:08<00:11,  9.67it/s]C:\Anaconda3\lib\site-packages\ipykernel\__main__.py:12: DeprecationWarning: 
.ix is deprecated. Please use
.loc for label based indexing or
.iloc for positional indexing

See the documentation here:
http://pandas.pydata.org/pandas-docs/stable/indexing.html#ix-indexer-is-deprecated
C:\Anaconda3\lib\site-packages\ipykernel\__main__.py:12: DeprecationWarning: 
.ix is deprecated. Please use
.loc for label based indexing or
.iloc for positional indexing

See the documentation here:
http://pandas.pydata.org/pandas-docs/stable/indexing.html#ix-indexer-is-deprecated
 45%|█████▊       | 90/200 [00:09<00:11,  9.87it/s]C:\Anaconda3\lib\site-packages\ipykernel\__main__.py:12: DeprecationWarning: 
.ix is deprecated. Please use
.loc for label based indexing or
.iloc for positional indexing

See the documentation here:
http://pandas.pydata.org/pandas-docs/stable/indexing.html#ix-indexer-is-deprecated
 46%|█████▉       | 91/200 [00:09<00:11,  9.65it/s]C:\Anaconda3\lib\site-packages\ipykernel\__main__.py:12: DeprecationWarning: 
.ix is deprecated. Please use
.loc for label based indexing or
.iloc for positional indexing

See the documentation here:
http://pandas.pydata.org/pandas-docs/stable/indexing.html#ix-indexer-is-deprecated
 46%|█████▉       | 92/200 [00:09<00:11,  9.47it/s]C:\Anaconda3\lib\site-packages\ipykernel\__main__.py:12: DeprecationWarning: 
.ix is deprecated. Please use
.loc for label based indexing or
.iloc for positional indexing

See the documentation here:
http://pandas.pydata.org/pandas-docs/stable/indexing.html#ix-indexer-is-deprecated
 46%|██████       | 93/200 [00:09<00:11,  9.10it/s]C:\Anaconda3\lib\site-packages\ipykernel\__main__.py:12: DeprecationWarning: 
.ix is deprecated. Please use
.loc for label based indexing or
.iloc for positional indexing

See the documentation here:
http://pandas.pydata.org/pandas-docs/stable/indexing.html#ix-indexer-is-deprecated
 47%|██████       | 94/200 [00:09<00:11,  9.05it/s]C:\Anaconda3\lib\site-packages\ipykernel\__main__.py:12: DeprecationWarning: 
.ix is deprecated. Please use
.loc for label based indexing or
.iloc for positional indexing

See the documentation here:
http://pandas.pydata.org/pandas-docs/stable/indexing.html#ix-indexer-is-deprecated
 48%|██████▏      | 95/200 [00:09<00:11,  9.20it/s]C:\Anaconda3\lib\site-packages\ipykernel\__main__.py:12: DeprecationWarning: 
.ix is deprecated. Please use
.loc for label based indexing or
.iloc for positional indexing

See the documentation here:
http://pandas.pydata.org/pandas-docs/stable/indexing.html#ix-indexer-is-deprecated
C:\Anaconda3\lib\site-packages\ipykernel\__main__.py:12: DeprecationWarning: 
.ix is deprecated. Please use
.loc for label based indexing or
.iloc for positional indexing

See the documentation here:
http://pandas.pydata.org/pandas-docs/stable/indexing.html#ix-indexer-is-deprecated
 48%|██████▎      | 97/200 [00:09<00:10,  9.37it/s]C:\Anaconda3\lib\site-packages\ipykernel\__main__.py:12: DeprecationWarning: 
.ix is deprecated. Please use
.loc for label based indexing or
.iloc for positional indexing

See the documentation here:
http://pandas.pydata.org/pandas-docs/stable/indexing.html#ix-indexer-is-deprecated
 49%|██████▎      | 98/200 [00:09<00:10,  9.42it/s]C:\Anaconda3\lib\site-packages\ipykernel\__main__.py:12: DeprecationWarning: 
.ix is deprecated. Please use
.loc for label based indexing or
.iloc for positional indexing

See the documentation here:
http://pandas.pydata.org/pandas-docs/stable/indexing.html#ix-indexer-is-deprecated
 50%|██████▍      | 99/200 [00:10<00:10,  9.50it/s]C:\Anaconda3\lib\site-packages\ipykernel\__main__.py:12: DeprecationWarning: 
.ix is deprecated. Please use
.loc for label based indexing or
.iloc for positional indexing

See the documentation here:
http://pandas.pydata.org/pandas-docs/stable/indexing.html#ix-indexer-is-deprecated
C:\Anaconda3\lib\site-packages\ipykernel\__main__.py:12: DeprecationWarning: 
.ix is deprecated. Please use
.loc for label based indexing or
.iloc for positional indexing

See the documentation here:
http://pandas.pydata.org/pandas-docs/stable/indexing.html#ix-indexer-is-deprecated
 50%|██████      | 101/200 [00:10<00:10,  9.82it/s]C:\Anaconda3\lib\site-packages\ipykernel\__main__.py:12: DeprecationWarning: 
.ix is deprecated. Please use
.loc for label based indexing or
.iloc for positional indexing

See the documentation here:
http://pandas.pydata.org/pandas-docs/stable/indexing.html#ix-indexer-is-deprecated
 51%|██████      | 102/200 [00:10<00:09,  9.84it/s]C:\Anaconda3\lib\site-packages\ipykernel\__main__.py:12: DeprecationWarning: 
.ix is deprecated. Please use
.loc for label based indexing or
.iloc for positional indexing

See the documentation here:
http://pandas.pydata.org/pandas-docs/stable/indexing.html#ix-indexer-is-deprecated
C:\Anaconda3\lib\site-packages\ipykernel\__main__.py:12: DeprecationWarning: 
.ix is deprecated. Please use
.loc for label based indexing or
.iloc for positional indexing

See the documentation here:
http://pandas.pydata.org/pandas-docs/stable/indexing.html#ix-indexer-is-deprecated
 52%|██████▏     | 104/200 [00:10<00:09, 10.02it/s]C:\Anaconda3\lib\site-packages\ipykernel\__main__.py:12: DeprecationWarning: 
.ix is deprecated. Please use
.loc for label based indexing or
.iloc for positional indexing

See the documentation here:
http://pandas.pydata.org/pandas-docs/stable/indexing.html#ix-indexer-is-deprecated
C:\Anaconda3\lib\site-packages\ipykernel\__main__.py:12: DeprecationWarning: 
.ix is deprecated. Please use
.loc for label based indexing or
.iloc for positional indexing

See the documentation here:
http://pandas.pydata.org/pandas-docs/stable/indexing.html#ix-indexer-is-deprecated
 53%|██████▎     | 106/200 [00:10<00:09, 10.12it/s]C:\Anaconda3\lib\site-packages\ipykernel\__main__.py:12: DeprecationWarning: 
.ix is deprecated. Please use
.loc for label based indexing or
.iloc for positional indexing

See the documentation here:
http://pandas.pydata.org/pandas-docs/stable/indexing.html#ix-indexer-is-deprecated
C:\Anaconda3\lib\site-packages\ipykernel\__main__.py:12: DeprecationWarning: 
.ix is deprecated. Please use
.loc for label based indexing or
.iloc for positional indexing

See the documentation here:
http://pandas.pydata.org/pandas-docs/stable/indexing.html#ix-indexer-is-deprecated
 54%|██████▍     | 108/200 [00:10<00:08, 10.35it/s]C:\Anaconda3\lib\site-packages\ipykernel\__main__.py:12: DeprecationWarning: 
.ix is deprecated. Please use
.loc for label based indexing or
.iloc for positional indexing

See the documentation here:
http://pandas.pydata.org/pandas-docs/stable/indexing.html#ix-indexer-is-deprecated
C:\Anaconda3\lib\site-packages\ipykernel\__main__.py:12: DeprecationWarning: 
.ix is deprecated. Please use
.loc for label based indexing or
.iloc for positional indexing

See the documentation here:
http://pandas.pydata.org/pandas-docs/stable/indexing.html#ix-indexer-is-deprecated
 55%|██████▌     | 110/200 [00:11<00:08, 10.67it/s]C:\Anaconda3\lib\site-packages\ipykernel\__main__.py:12: DeprecationWarning: 
.ix is deprecated. Please use
.loc for label based indexing or
.iloc for positional indexing

See the documentation here:
http://pandas.pydata.org/pandas-docs/stable/indexing.html#ix-indexer-is-deprecated
C:\Anaconda3\lib\site-packages\ipykernel\__main__.py:12: DeprecationWarning: 
.ix is deprecated. Please use
.loc for label based indexing or
.iloc for positional indexing

See the documentation here:
http://pandas.pydata.org/pandas-docs/stable/indexing.html#ix-indexer-is-deprecated
 56%|██████▋     | 112/200 [00:11<00:08, 10.09it/s]C:\Anaconda3\lib\site-packages\ipykernel\__main__.py:12: DeprecationWarning: 
.ix is deprecated. Please use
.loc for label based indexing or
.iloc for positional indexing

See the documentation here:
http://pandas.pydata.org/pandas-docs/stable/indexing.html#ix-indexer-is-deprecated
C:\Anaconda3\lib\site-packages\ipykernel\__main__.py:12: DeprecationWarning: 
.ix is deprecated. Please use
.loc for label based indexing or
.iloc for positional indexing

See the documentation here:
http://pandas.pydata.org/pandas-docs/stable/indexing.html#ix-indexer-is-deprecated
 57%|██████▊     | 114/200 [00:11<00:08,  9.97it/s]C:\Anaconda3\lib\site-packages\ipykernel\__main__.py:12: DeprecationWarning: 
.ix is deprecated. Please use
.loc for label based indexing or
.iloc for positional indexing

See the documentation here:
http://pandas.pydata.org/pandas-docs/stable/indexing.html#ix-indexer-is-deprecated
C:\Anaconda3\lib\site-packages\ipykernel\__main__.py:12: DeprecationWarning: 
.ix is deprecated. Please use
.loc for label based indexing or
.iloc for positional indexing

See the documentation here:
http://pandas.pydata.org/pandas-docs/stable/indexing.html#ix-indexer-is-deprecated
 58%|██████▉     | 116/200 [00:11<00:08, 10.15it/s]C:\Anaconda3\lib\site-packages\ipykernel\__main__.py:12: DeprecationWarning: 
.ix is deprecated. Please use
.loc for label based indexing or
.iloc for positional indexing

See the documentation here:
http://pandas.pydata.org/pandas-docs/stable/indexing.html#ix-indexer-is-deprecated
C:\Anaconda3\lib\site-packages\ipykernel\__main__.py:12: DeprecationWarning: 
.ix is deprecated. Please use
.loc for label based indexing or
.iloc for positional indexing

See the documentation here:
http://pandas.pydata.org/pandas-docs/stable/indexing.html#ix-indexer-is-deprecated
 59%|███████     | 118/200 [00:11<00:08, 10.11it/s]C:\Anaconda3\lib\site-packages\ipykernel\__main__.py:12: DeprecationWarning: 
.ix is deprecated. Please use
.loc for label based indexing or
.iloc for positional indexing

See the documentation here:
http://pandas.pydata.org/pandas-docs/stable/indexing.html#ix-indexer-is-deprecated
C:\Anaconda3\lib\site-packages\ipykernel\__main__.py:12: DeprecationWarning: 
.ix is deprecated. Please use
.loc for label based indexing or
.iloc for positional indexing

See the documentation here:
http://pandas.pydata.org/pandas-docs/stable/indexing.html#ix-indexer-is-deprecated
 60%|███████▏    | 120/200 [00:12<00:07, 10.20it/s]C:\Anaconda3\lib\site-packages\ipykernel\__main__.py:12: DeprecationWarning: 
.ix is deprecated. Please use
.loc for label based indexing or
.iloc for positional indexing

See the documentation here:
http://pandas.pydata.org/pandas-docs/stable/indexing.html#ix-indexer-is-deprecated
C:\Anaconda3\lib\site-packages\ipykernel\__main__.py:12: DeprecationWarning: 
.ix is deprecated. Please use
.loc for label based indexing or
.iloc for positional indexing

See the documentation here:
http://pandas.pydata.org/pandas-docs/stable/indexing.html#ix-indexer-is-deprecated
 61%|███████▎    | 122/200 [00:12<00:07, 10.18it/s]C:\Anaconda3\lib\site-packages\ipykernel\__main__.py:12: DeprecationWarning: 
.ix is deprecated. Please use
.loc for label based indexing or
.iloc for positional indexing

See the documentation here:
http://pandas.pydata.org/pandas-docs/stable/indexing.html#ix-indexer-is-deprecated
C:\Anaconda3\lib\site-packages\ipykernel\__main__.py:12: DeprecationWarning: 
.ix is deprecated. Please use
.loc for label based indexing or
.iloc for positional indexing

See the documentation here:
http://pandas.pydata.org/pandas-docs/stable/indexing.html#ix-indexer-is-deprecated
 62%|███████▍    | 124/200 [00:12<00:07, 10.28it/s]C:\Anaconda3\lib\site-packages\ipykernel\__main__.py:12: DeprecationWarning: 
.ix is deprecated. Please use
.loc for label based indexing or
.iloc for positional indexing

See the documentation here:
http://pandas.pydata.org/pandas-docs/stable/indexing.html#ix-indexer-is-deprecated
C:\Anaconda3\lib\site-packages\ipykernel\__main__.py:12: DeprecationWarning: 
.ix is deprecated. Please use
.loc for label based indexing or
.iloc for positional indexing

See the documentation here:
http://pandas.pydata.org/pandas-docs/stable/indexing.html#ix-indexer-is-deprecated
 63%|███████▌    | 126/200 [00:12<00:07, 10.18it/s]C:\Anaconda3\lib\site-packages\ipykernel\__main__.py:12: DeprecationWarning: 
.ix is deprecated. Please use
.loc for label based indexing or
.iloc for positional indexing

See the documentation here:
http://pandas.pydata.org/pandas-docs/stable/indexing.html#ix-indexer-is-deprecated
C:\Anaconda3\lib\site-packages\ipykernel\__main__.py:12: DeprecationWarning: 
.ix is deprecated. Please use
.loc for label based indexing or
.iloc for positional indexing

See the documentation here:
http://pandas.pydata.org/pandas-docs/stable/indexing.html#ix-indexer-is-deprecated
 64%|███████▋    | 128/200 [00:12<00:07, 10.19it/s]C:\Anaconda3\lib\site-packages\ipykernel\__main__.py:12: DeprecationWarning: 
.ix is deprecated. Please use
.loc for label based indexing or
.iloc for positional indexing

See the documentation here:
http://pandas.pydata.org/pandas-docs/stable/indexing.html#ix-indexer-is-deprecated
C:\Anaconda3\lib\site-packages\ipykernel\__main__.py:12: DeprecationWarning: 
.ix is deprecated. Please use
.loc for label based indexing or
.iloc for positional indexing

See the documentation here:
http://pandas.pydata.org/pandas-docs/stable/indexing.html#ix-indexer-is-deprecated
 65%|███████▊    | 130/200 [00:13<00:06, 10.25it/s]C:\Anaconda3\lib\site-packages\ipykernel\__main__.py:12: DeprecationWarning: 
.ix is deprecated. Please use
.loc for label based indexing or
.iloc for positional indexing

See the documentation here:
http://pandas.pydata.org/pandas-docs/stable/indexing.html#ix-indexer-is-deprecated
C:\Anaconda3\lib\site-packages\ipykernel\__main__.py:12: DeprecationWarning: 
.ix is deprecated. Please use
.loc for label based indexing or
.iloc for positional indexing

See the documentation here:
http://pandas.pydata.org/pandas-docs/stable/indexing.html#ix-indexer-is-deprecated
 66%|███████▉    | 132/200 [00:13<00:06, 10.38it/s]C:\Anaconda3\lib\site-packages\ipykernel\__main__.py:12: DeprecationWarning: 
.ix is deprecated. Please use
.loc for label based indexing or
.iloc for positional indexing

See the documentation here:
http://pandas.pydata.org/pandas-docs/stable/indexing.html#ix-indexer-is-deprecated
C:\Anaconda3\lib\site-packages\ipykernel\__main__.py:12: DeprecationWarning: 
.ix is deprecated. Please use
.loc for label based indexing or
.iloc for positional indexing

See the documentation here:
http://pandas.pydata.org/pandas-docs/stable/indexing.html#ix-indexer-is-deprecated
 67%|████████    | 134/200 [00:13<00:06, 10.04it/s]C:\Anaconda3\lib\site-packages\ipykernel\__main__.py:12: DeprecationWarning: 
.ix is deprecated. Please use
.loc for label based indexing or
.iloc for positional indexing

See the documentation here:
http://pandas.pydata.org/pandas-docs/stable/indexing.html#ix-indexer-is-deprecated
C:\Anaconda3\lib\site-packages\ipykernel\__main__.py:12: DeprecationWarning: 
.ix is deprecated. Please use
.loc for label based indexing or
.iloc for positional indexing

See the documentation here:
http://pandas.pydata.org/pandas-docs/stable/indexing.html#ix-indexer-is-deprecated
 68%|████████▏   | 136/200 [00:13<00:06, 10.09it/s]C:\Anaconda3\lib\site-packages\ipykernel\__main__.py:12: DeprecationWarning: 
.ix is deprecated. Please use
.loc for label based indexing or
.iloc for positional indexing

See the documentation here:
http://pandas.pydata.org/pandas-docs/stable/indexing.html#ix-indexer-is-deprecated
C:\Anaconda3\lib\site-packages\ipykernel\__main__.py:12: DeprecationWarning: 
.ix is deprecated. Please use
.loc for label based indexing or
.iloc for positional indexing

See the documentation here:
http://pandas.pydata.org/pandas-docs/stable/indexing.html#ix-indexer-is-deprecated
 69%|████████▎   | 138/200 [00:13<00:06,  9.82it/s]C:\Anaconda3\lib\site-packages\ipykernel\__main__.py:12: DeprecationWarning: 
.ix is deprecated. Please use
.loc for label based indexing or
.iloc for positional indexing

See the documentation here:
http://pandas.pydata.org/pandas-docs/stable/indexing.html#ix-indexer-is-deprecated
C:\Anaconda3\lib\site-packages\ipykernel\__main__.py:12: DeprecationWarning: 
.ix is deprecated. Please use
.loc for label based indexing or
.iloc for positional indexing

See the documentation here:
http://pandas.pydata.org/pandas-docs/stable/indexing.html#ix-indexer-is-deprecated
 70%|████████▍   | 140/200 [00:14<00:06,  9.63it/s]C:\Anaconda3\lib\site-packages\ipykernel\__main__.py:12: DeprecationWarning: 
.ix is deprecated. Please use
.loc for label based indexing or
.iloc for positional indexing

See the documentation here:
http://pandas.pydata.org/pandas-docs/stable/indexing.html#ix-indexer-is-deprecated
 70%|████████▍   | 141/200 [00:14<00:06,  8.53it/s]C:\Anaconda3\lib\site-packages\ipykernel\__main__.py:12: DeprecationWarning: 
.ix is deprecated. Please use
.loc for label based indexing or
.iloc for positional indexing

See the documentation here:
http://pandas.pydata.org/pandas-docs/stable/indexing.html#ix-indexer-is-deprecated
 71%|████████▌   | 142/200 [00:14<00:07,  7.67it/s]C:\Anaconda3\lib\site-packages\ipykernel\__main__.py:12: DeprecationWarning: 
.ix is deprecated. Please use
.loc for label based indexing or
.iloc for positional indexing

See the documentation here:
http://pandas.pydata.org/pandas-docs/stable/indexing.html#ix-indexer-is-deprecated
 72%|████████▌   | 143/200 [00:14<00:07,  8.10it/s]C:\Anaconda3\lib\site-packages\ipykernel\__main__.py:12: DeprecationWarning: 
.ix is deprecated. Please use
.loc for label based indexing or
.iloc for positional indexing

See the documentation here:
http://pandas.pydata.org/pandas-docs/stable/indexing.html#ix-indexer-is-deprecated
C:\Anaconda3\lib\site-packages\ipykernel\__main__.py:12: DeprecationWarning: 
.ix is deprecated. Please use
.loc for label based indexing or
.iloc for positional indexing

See the documentation here:
http://pandas.pydata.org/pandas-docs/stable/indexing.html#ix-indexer-is-deprecated
 72%|████████▋   | 145/200 [00:14<00:06,  8.69it/s]C:\Anaconda3\lib\site-packages\ipykernel\__main__.py:12: DeprecationWarning: 
.ix is deprecated. Please use
.loc for label based indexing or
.iloc for positional indexing

See the documentation here:
http://pandas.pydata.org/pandas-docs/stable/indexing.html#ix-indexer-is-deprecated
 73%|████████▊   | 146/200 [00:14<00:06,  8.90it/s]C:\Anaconda3\lib\site-packages\ipykernel\__main__.py:12: DeprecationWarning: 
.ix is deprecated. Please use
.loc for label based indexing or
.iloc for positional indexing

See the documentation here:
http://pandas.pydata.org/pandas-docs/stable/indexing.html#ix-indexer-is-deprecated
C:\Anaconda3\lib\site-packages\ipykernel\__main__.py:12: DeprecationWarning: 
.ix is deprecated. Please use
.loc for label based indexing or
.iloc for positional indexing

See the documentation here:
http://pandas.pydata.org/pandas-docs/stable/indexing.html#ix-indexer-is-deprecated
 74%|████████▉   | 148/200 [00:14<00:05,  9.26it/s]C:\Anaconda3\lib\site-packages\ipykernel\__main__.py:12: DeprecationWarning: 
.ix is deprecated. Please use
.loc for label based indexing or
.iloc for positional indexing

See the documentation here:
http://pandas.pydata.org/pandas-docs/stable/indexing.html#ix-indexer-is-deprecated
 74%|████████▉   | 149/200 [00:15<00:05,  9.18it/s]C:\Anaconda3\lib\site-packages\ipykernel\__main__.py:12: DeprecationWarning: 
.ix is deprecated. Please use
.loc for label based indexing or
.iloc for positional indexing

See the documentation here:
http://pandas.pydata.org/pandas-docs/stable/indexing.html#ix-indexer-is-deprecated
C:\Anaconda3\lib\site-packages\ipykernel\__main__.py:12: DeprecationWarning: 
.ix is deprecated. Please use
.loc for label based indexing or
.iloc for positional indexing

See the documentation here:
http://pandas.pydata.org/pandas-docs/stable/indexing.html#ix-indexer-is-deprecated
 76%|█████████   | 151/200 [00:15<00:05,  9.57it/s]C:\Anaconda3\lib\site-packages\ipykernel\__main__.py:12: DeprecationWarning: 
.ix is deprecated. Please use
.loc for label based indexing or
.iloc for positional indexing

See the documentation here:
http://pandas.pydata.org/pandas-docs/stable/indexing.html#ix-indexer-is-deprecated
 76%|█████████   | 152/200 [00:15<00:05,  9.40it/s]C:\Anaconda3\lib\site-packages\ipykernel\__main__.py:12: DeprecationWarning: 
.ix is deprecated. Please use
.loc for label based indexing or
.iloc for positional indexing

See the documentation here:
http://pandas.pydata.org/pandas-docs/stable/indexing.html#ix-indexer-is-deprecated
C:\Anaconda3\lib\site-packages\ipykernel\__main__.py:12: DeprecationWarning: 
.ix is deprecated. Please use
.loc for label based indexing or
.iloc for positional indexing

See the documentation here:
http://pandas.pydata.org/pandas-docs/stable/indexing.html#ix-indexer-is-deprecated
 77%|█████████▏  | 154/200 [00:15<00:04,  9.80it/s]C:\Anaconda3\lib\site-packages\ipykernel\__main__.py:12: DeprecationWarning: 
.ix is deprecated. Please use
.loc for label based indexing or
.iloc for positional indexing

See the documentation here:
http://pandas.pydata.org/pandas-docs/stable/indexing.html#ix-indexer-is-deprecated
C:\Anaconda3\lib\site-packages\ipykernel\__main__.py:12: DeprecationWarning: 
.ix is deprecated. Please use
.loc for label based indexing or
.iloc for positional indexing

See the documentation here:
http://pandas.pydata.org/pandas-docs/stable/indexing.html#ix-indexer-is-deprecated
 78%|█████████▎  | 156/200 [00:15<00:04,  9.99it/s]C:\Anaconda3\lib\site-packages\ipykernel\__main__.py:12: DeprecationWarning: 
.ix is deprecated. Please use
.loc for label based indexing or
.iloc for positional indexing

See the documentation here:
http://pandas.pydata.org/pandas-docs/stable/indexing.html#ix-indexer-is-deprecated
C:\Anaconda3\lib\site-packages\ipykernel\__main__.py:12: DeprecationWarning: 
.ix is deprecated. Please use
.loc for label based indexing or
.iloc for positional indexing

See the documentation here:
http://pandas.pydata.org/pandas-docs/stable/indexing.html#ix-indexer-is-deprecated
 79%|█████████▍  | 158/200 [00:15<00:04, 10.19it/s]C:\Anaconda3\lib\site-packages\ipykernel\__main__.py:12: DeprecationWarning: 
.ix is deprecated. Please use
.loc for label based indexing or
.iloc for positional indexing

See the documentation here:
http://pandas.pydata.org/pandas-docs/stable/indexing.html#ix-indexer-is-deprecated
C:\Anaconda3\lib\site-packages\ipykernel\__main__.py:12: DeprecationWarning: 
.ix is deprecated. Please use
.loc for label based indexing or
.iloc for positional indexing

See the documentation here:
http://pandas.pydata.org/pandas-docs/stable/indexing.html#ix-indexer-is-deprecated
 80%|█████████▌  | 160/200 [00:16<00:03, 10.15it/s]C:\Anaconda3\lib\site-packages\ipykernel\__main__.py:12: DeprecationWarning: 
.ix is deprecated. Please use
.loc for label based indexing or
.iloc for positional indexing

See the documentation here:
http://pandas.pydata.org/pandas-docs/stable/indexing.html#ix-indexer-is-deprecated
C:\Anaconda3\lib\site-packages\ipykernel\__main__.py:12: DeprecationWarning: 
.ix is deprecated. Please use
.loc for label based indexing or
.iloc for positional indexing

See the documentation here:
http://pandas.pydata.org/pandas-docs/stable/indexing.html#ix-indexer-is-deprecated
 81%|█████████▋  | 162/200 [00:16<00:03,  9.91it/s]C:\Anaconda3\lib\site-packages\ipykernel\__main__.py:12: DeprecationWarning: 
.ix is deprecated. Please use
.loc for label based indexing or
.iloc for positional indexing

See the documentation here:
http://pandas.pydata.org/pandas-docs/stable/indexing.html#ix-indexer-is-deprecated
C:\Anaconda3\lib\site-packages\ipykernel\__main__.py:12: DeprecationWarning: 
.ix is deprecated. Please use
.loc for label based indexing or
.iloc for positional indexing

See the documentation here:
http://pandas.pydata.org/pandas-docs/stable/indexing.html#ix-indexer-is-deprecated
 82%|█████████▊  | 164/200 [00:16<00:03, 10.04it/s]C:\Anaconda3\lib\site-packages\ipykernel\__main__.py:12: DeprecationWarning: 
.ix is deprecated. Please use
.loc for label based indexing or
.iloc for positional indexing

See the documentation here:
http://pandas.pydata.org/pandas-docs/stable/indexing.html#ix-indexer-is-deprecated
C:\Anaconda3\lib\site-packages\ipykernel\__main__.py:12: DeprecationWarning: 
.ix is deprecated. Please use
.loc for label based indexing or
.iloc for positional indexing

See the documentation here:
http://pandas.pydata.org/pandas-docs/stable/indexing.html#ix-indexer-is-deprecated
 83%|█████████▉  | 166/200 [00:16<00:03, 10.03it/s]C:\Anaconda3\lib\site-packages\ipykernel\__main__.py:12: DeprecationWarning: 
.ix is deprecated. Please use
.loc for label based indexing or
.iloc for positional indexing

See the documentation here:
http://pandas.pydata.org/pandas-docs/stable/indexing.html#ix-indexer-is-deprecated
C:\Anaconda3\lib\site-packages\ipykernel\__main__.py:12: DeprecationWarning: 
.ix is deprecated. Please use
.loc for label based indexing or
.iloc for positional indexing

See the documentation here:
http://pandas.pydata.org/pandas-docs/stable/indexing.html#ix-indexer-is-deprecated
 84%|██████████  | 168/200 [00:16<00:03, 10.27it/s]C:\Anaconda3\lib\site-packages\ipykernel\__main__.py:12: DeprecationWarning: 
.ix is deprecated. Please use
.loc for label based indexing or
.iloc for positional indexing

See the documentation here:
http://pandas.pydata.org/pandas-docs/stable/indexing.html#ix-indexer-is-deprecated
C:\Anaconda3\lib\site-packages\ipykernel\__main__.py:12: DeprecationWarning: 
.ix is deprecated. Please use
.loc for label based indexing or
.iloc for positional indexing

See the documentation here:
http://pandas.pydata.org/pandas-docs/stable/indexing.html#ix-indexer-is-deprecated
 85%|██████████▏ | 170/200 [00:17<00:02, 10.28it/s]C:\Anaconda3\lib\site-packages\ipykernel\__main__.py:12: DeprecationWarning: 
.ix is deprecated. Please use
.loc for label based indexing or
.iloc for positional indexing

See the documentation here:
http://pandas.pydata.org/pandas-docs/stable/indexing.html#ix-indexer-is-deprecated
C:\Anaconda3\lib\site-packages\ipykernel\__main__.py:12: DeprecationWarning: 
.ix is deprecated. Please use
.loc for label based indexing or
.iloc for positional indexing

See the documentation here:
http://pandas.pydata.org/pandas-docs/stable/indexing.html#ix-indexer-is-deprecated
 86%|██████████▎ | 172/200 [00:17<00:02, 10.13it/s]C:\Anaconda3\lib\site-packages\ipykernel\__main__.py:12: DeprecationWarning: 
.ix is deprecated. Please use
.loc for label based indexing or
.iloc for positional indexing

See the documentation here:
http://pandas.pydata.org/pandas-docs/stable/indexing.html#ix-indexer-is-deprecated
C:\Anaconda3\lib\site-packages\ipykernel\__main__.py:12: DeprecationWarning: 
.ix is deprecated. Please use
.loc for label based indexing or
.iloc for positional indexing

See the documentation here:
http://pandas.pydata.org/pandas-docs/stable/indexing.html#ix-indexer-is-deprecated
 87%|██████████▍ | 174/200 [00:17<00:02, 10.14it/s]C:\Anaconda3\lib\site-packages\ipykernel\__main__.py:12: DeprecationWarning: 
.ix is deprecated. Please use
.loc for label based indexing or
.iloc for positional indexing

See the documentation here:
http://pandas.pydata.org/pandas-docs/stable/indexing.html#ix-indexer-is-deprecated
C:\Anaconda3\lib\site-packages\ipykernel\__main__.py:12: DeprecationWarning: 
.ix is deprecated. Please use
.loc for label based indexing or
.iloc for positional indexing

See the documentation here:
http://pandas.pydata.org/pandas-docs/stable/indexing.html#ix-indexer-is-deprecated
 88%|██████████▌ | 176/200 [00:17<00:02,  9.70it/s]C:\Anaconda3\lib\site-packages\ipykernel\__main__.py:12: DeprecationWarning: 
.ix is deprecated. Please use
.loc for label based indexing or
.iloc for positional indexing

See the documentation here:
http://pandas.pydata.org/pandas-docs/stable/indexing.html#ix-indexer-is-deprecated
C:\Anaconda3\lib\site-packages\ipykernel\__main__.py:12: DeprecationWarning: 
.ix is deprecated. Please use
.loc for label based indexing or
.iloc for positional indexing

See the documentation here:
http://pandas.pydata.org/pandas-docs/stable/indexing.html#ix-indexer-is-deprecated
 89%|██████████▋ | 178/200 [00:17<00:02,  9.65it/s]C:\Anaconda3\lib\site-packages\ipykernel\__main__.py:12: DeprecationWarning: 
.ix is deprecated. Please use
.loc for label based indexing or
.iloc for positional indexing

See the documentation here:
http://pandas.pydata.org/pandas-docs/stable/indexing.html#ix-indexer-is-deprecated
 90%|██████████▋ | 179/200 [00:18<00:02,  9.34it/s]C:\Anaconda3\lib\site-packages\ipykernel\__main__.py:12: DeprecationWarning: 
.ix is deprecated. Please use
.loc for label based indexing or
.iloc for positional indexing

See the documentation here:
http://pandas.pydata.org/pandas-docs/stable/indexing.html#ix-indexer-is-deprecated
 90%|██████████▊ | 180/200 [00:18<00:02,  9.45it/s]C:\Anaconda3\lib\site-packages\ipykernel\__main__.py:12: DeprecationWarning: 
.ix is deprecated. Please use
.loc for label based indexing or
.iloc for positional indexing

See the documentation here:
http://pandas.pydata.org/pandas-docs/stable/indexing.html#ix-indexer-is-deprecated
 90%|██████████▊ | 181/200 [00:18<00:01,  9.55it/s]C:\Anaconda3\lib\site-packages\ipykernel\__main__.py:12: DeprecationWarning: 
.ix is deprecated. Please use
.loc for label based indexing or
.iloc for positional indexing

See the documentation here:
http://pandas.pydata.org/pandas-docs/stable/indexing.html#ix-indexer-is-deprecated
C:\Anaconda3\lib\site-packages\ipykernel\__main__.py:12: DeprecationWarning: 
.ix is deprecated. Please use
.loc for label based indexing or
.iloc for positional indexing

See the documentation here:
http://pandas.pydata.org/pandas-docs/stable/indexing.html#ix-indexer-is-deprecated
 92%|██████████▉ | 183/200 [00:18<00:01,  9.71it/s]C:\Anaconda3\lib\site-packages\ipykernel\__main__.py:12: DeprecationWarning: 
.ix is deprecated. Please use
.loc for label based indexing or
.iloc for positional indexing

See the documentation here:
http://pandas.pydata.org/pandas-docs/stable/indexing.html#ix-indexer-is-deprecated
 92%|███████████ | 184/200 [00:18<00:01,  9.60it/s]C:\Anaconda3\lib\site-packages\ipykernel\__main__.py:12: DeprecationWarning: 
.ix is deprecated. Please use
.loc for label based indexing or
.iloc for positional indexing

See the documentation here:
http://pandas.pydata.org/pandas-docs/stable/indexing.html#ix-indexer-is-deprecated
C:\Anaconda3\lib\site-packages\ipykernel\__main__.py:12: DeprecationWarning: 
.ix is deprecated. Please use
.loc for label based indexing or
.iloc for positional indexing

See the documentation here:
http://pandas.pydata.org/pandas-docs/stable/indexing.html#ix-indexer-is-deprecated
 93%|███████████▏| 186/200 [00:18<00:01,  9.82it/s]C:\Anaconda3\lib\site-packages\ipykernel\__main__.py:12: DeprecationWarning: 
.ix is deprecated. Please use
.loc for label based indexing or
.iloc for positional indexing

See the documentation here:
http://pandas.pydata.org/pandas-docs/stable/indexing.html#ix-indexer-is-deprecated
C:\Anaconda3\lib\site-packages\ipykernel\__main__.py:12: DeprecationWarning: 
.ix is deprecated. Please use
.loc for label based indexing or
.iloc for positional indexing

See the documentation here:
http://pandas.pydata.org/pandas-docs/stable/indexing.html#ix-indexer-is-deprecated
 94%|███████████▎| 188/200 [00:18<00:01,  9.93it/s]C:\Anaconda3\lib\site-packages\ipykernel\__main__.py:12: DeprecationWarning: 
.ix is deprecated. Please use
.loc for label based indexing or
.iloc for positional indexing

See the documentation here:
http://pandas.pydata.org/pandas-docs/stable/indexing.html#ix-indexer-is-deprecated
 94%|███████████▎| 189/200 [00:19<00:01,  9.89it/s]C:\Anaconda3\lib\site-packages\ipykernel\__main__.py:12: DeprecationWarning: 
.ix is deprecated. Please use
.loc for label based indexing or
.iloc for positional indexing

See the documentation here:
http://pandas.pydata.org/pandas-docs/stable/indexing.html#ix-indexer-is-deprecated
C:\Anaconda3\lib\site-packages\ipykernel\__main__.py:12: DeprecationWarning: 
.ix is deprecated. Please use
.loc for label based indexing or
.iloc for positional indexing

See the documentation here:
http://pandas.pydata.org/pandas-docs/stable/indexing.html#ix-indexer-is-deprecated
 96%|███████████▍| 191/200 [00:19<00:00,  9.84it/s]C:\Anaconda3\lib\site-packages\ipykernel\__main__.py:12: DeprecationWarning: 
.ix is deprecated. Please use
.loc for label based indexing or
.iloc for positional indexing

See the documentation here:
http://pandas.pydata.org/pandas-docs/stable/indexing.html#ix-indexer-is-deprecated
C:\Anaconda3\lib\site-packages\ipykernel\__main__.py:12: DeprecationWarning: 
.ix is deprecated. Please use
.loc for label based indexing or
.iloc for positional indexing

See the documentation here:
http://pandas.pydata.org/pandas-docs/stable/indexing.html#ix-indexer-is-deprecated
 96%|███████████▌| 193/200 [00:19<00:00,  9.73it/s]C:\Anaconda3\lib\site-packages\ipykernel\__main__.py:12: DeprecationWarning: 
.ix is deprecated. Please use
.loc for label based indexing or
.iloc for positional indexing

See the documentation here:
http://pandas.pydata.org/pandas-docs/stable/indexing.html#ix-indexer-is-deprecated
 97%|███████████▋| 194/200 [00:19<00:00,  9.66it/s]C:\Anaconda3\lib\site-packages\ipykernel\__main__.py:12: DeprecationWarning: 
.ix is deprecated. Please use
.loc for label based indexing or
.iloc for positional indexing

See the documentation here:
http://pandas.pydata.org/pandas-docs/stable/indexing.html#ix-indexer-is-deprecated
C:\Anaconda3\lib\site-packages\ipykernel\__main__.py:12: DeprecationWarning: 
.ix is deprecated. Please use
.loc for label based indexing or
.iloc for positional indexing

See the documentation here:
http://pandas.pydata.org/pandas-docs/stable/indexing.html#ix-indexer-is-deprecated
 98%|███████████▊| 196/200 [00:19<00:00, 10.06it/s]C:\Anaconda3\lib\site-packages\ipykernel\__main__.py:12: DeprecationWarning: 
.ix is deprecated. Please use
.loc for label based indexing or
.iloc for positional indexing

See the documentation here:
http://pandas.pydata.org/pandas-docs/stable/indexing.html#ix-indexer-is-deprecated
C:\Anaconda3\lib\site-packages\ipykernel\__main__.py:12: DeprecationWarning: 
.ix is deprecated. Please use
.loc for label based indexing or
.iloc for positional indexing

See the documentation here:
http://pandas.pydata.org/pandas-docs/stable/indexing.html#ix-indexer-is-deprecated
 99%|███████████▉| 198/200 [00:19<00:00, 10.19it/s]C:\Anaconda3\lib\site-packages\ipykernel\__main__.py:12: DeprecationWarning: 
.ix is deprecated. Please use
.loc for label based indexing or
.iloc for positional indexing

See the documentation here:
http://pandas.pydata.org/pandas-docs/stable/indexing.html#ix-indexer-is-deprecated
C:\Anaconda3\lib\site-packages\ipykernel\__main__.py:12: DeprecationWarning: 
.ix is deprecated. Please use
.loc for label based indexing or
.iloc for positional indexing

See the documentation here:
http://pandas.pydata.org/pandas-docs/stable/indexing.html#ix-indexer-is-deprecated
100%|████████████| 200/200 [00:20<00:00, 10.17it/s]
```

```
평균적인 threshold, 0.5 (0.01) [0.48-0.52]
평균적인 auc, 0.77 (0.06) [0.65-0.88]
평균적인 sensitivity, 0.71 (0.08) [0.52-0.87]
평균적인 specificity, 0.71 (0.07) [0.56-0.81]
평균적인 ppv, 0.68 (0.07) [0.54-0.8]
평균적인 npv, 0.74 (0.06) [0.62-0.87]
평균적인 accuracy, 0.71 (0.06) [0.58-0.82]
----------------------------------------------------------------------------------
<catboost.core.CatBoostClassifier object at 0x0000021620E03D68>
```

```
100%|██████████| 200/200 [1:43:29<00:00, 31.05s/it]
```

```
평균적인 threshold, 0.46 (0.17) [0.16-0.79]
평균적인 auc, 0.8 (0.05) [0.69-0.89]
평균적인 sensitivity, 0.73 (0.07) [0.57-0.87]
평균적인 specificity, 0.74 (0.07) [0.59-0.85]
평균적인 ppv, 0.7 (0.06) [0.58-0.82]
평균적인 npv, 0.76 (0.06) [0.66-0.88]
평균적인 accuracy, 0.73 (0.06) [0.62-0.84]
----------------------------------------------------------------------------------
```
