# Supplementary material for: Machine learning assessment of myocardial ischemia using angiography: Development and retrospective validation
Source: PLoS Med. 2018 Nov 13;15(11):e1002693. doi: 10.1371/journal.pmed.1002693 (PMC6233920; doi:10.1371/journal.pmed.1002693)
Supplement: S1 Data — (ZIP) [file pmed.1002693.s011.zip › S1_Data/180827_final_900_sp_ffr_unstack-addMLA.html]

180827\_final\_900\_sp\_ffr\_unstack-addMLA


In [1]:

```
import numpy as np
import pandas as pd
from sklearn.metrics import mean_absolute_error,mean_squared_error,median_absolute_error
from sklearn.model_selection import train_test_split, StratifiedKFold, KFold

from sklearn.linear_model import LassoCV, RidgeCV, ElasticNetCV, LinearRegression
from sklearn.ensemble import RandomForestRegressor, ExtraTreesRegressor, GradientBoostingRegressor

from sklearn.metrics import mean_absolute_error,mean_squared_error,median_absolute_error
```

In [2]:

```
!pip install pandas_ml
```

```
Requirement already satisfied: pandas_ml in c:\anaconda3\lib\site-packages (0.5.0)
Requirement already satisfied: pandas>=0.17.0 in c:\anaconda3\lib\site-packages (from pandas_ml) (0.21.0)
Requirement already satisfied: enum34 in c:\anaconda3\lib\site-packages (from pandas_ml) (1.1.6)
Requirement already satisfied: python-dateutil>=2 in c:\anaconda3\lib\site-packages (from pandas>=0.17.0->pandas_ml) (2.6.1)
Requirement already satisfied: numpy>=1.9.0 in c:\anaconda3\lib\site-packages (from pandas>=0.17.0->pandas_ml) (1.14.0)
Requirement already satisfied: pytz>=2011k in c:\anaconda3\lib\site-packages (from pandas>=0.17.0->pandas_ml) (2017.3)
Requirement already satisfied: six>=1.5 in c:\anaconda3\lib\site-packages (from python-dateutil>=2->pandas>=0.17.0->pandas_ml) (1.11.0)
```

```
thrift-sasl 0.2.1 requires sasl>=0.2.1, which is not installed.
pyhs2 0.6.0 requires sasl, which is not installed.
pyhs2 0.6.0 requires thrift, which is not installed.
tensorflow-tensorboard 1.5.1 has requirement bleach==1.5.0, but you'll have bleach 2.1.3 which is incompatible.
tensorflow-tensorboard 1.5.1 has requirement html5lib==0.9999999, but you'll have html5lib 1.0.1 which is incompatible.
crowdai 1.0.10 has requirement Jinja2<2.10,>=2.9.6, but you'll have jinja2 2.10 which is incompatible.
crowdai 1.0.10 has requirement six<1.11,>=1.10, but you'll have six 1.11.0 which is incompatible.
crowdai 1.0.10 has requirement Werkzeug<0.13,>=0.12.1, but you'll have werkzeug 0.14.1 which is incompatible.
docker-py 1.10.3 has requirement requests<2.11,>=2.5.2, but you'll have requests 2.14.2 which is incompatible.
You are using pip version 10.0.1, however version 18.0 is available.
You should consider upgrading via the 'python -m pip install --upgrade pip' command.
```

In [3]:

```
!pip install tqdm
```

```
Requirement already satisfied: tqdm in c:\anaconda3\lib\site-packages (4.11.2)
```

```
thrift-sasl 0.2.1 requires sasl>=0.2.1, which is not installed.
pyhs2 0.6.0 requires sasl, which is not installed.
pyhs2 0.6.0 requires thrift, which is not installed.
tensorflow-tensorboard 1.5.1 has requirement bleach==1.5.0, but you'll have bleach 2.1.3 which is incompatible.
tensorflow-tensorboard 1.5.1 has requirement html5lib==0.9999999, but you'll have html5lib 1.0.1 which is incompatible.
crowdai 1.0.10 has requirement Jinja2<2.10,>=2.9.6, but you'll have jinja2 2.10 which is incompatible.
crowdai 1.0.10 has requirement six<1.11,>=1.10, but you'll have six 1.11.0 which is incompatible.
crowdai 1.0.10 has requirement Werkzeug<0.13,>=0.12.1, but you'll have werkzeug 0.14.1 which is incompatible.
docker-py 1.10.3 has requirement requests<2.11,>=2.5.2, but you'll have requests 2.14.2 which is incompatible.
You are using pip version 10.0.1, however version 18.0 is available.
You should consider upgrading via the 'python -m pip install --upgrade pip' command.
```

In [4]:

```
final_900 = pd.read_csv('subterritory_final_aug28_MLA1.csv',encoding='cp949')
final_900 = pd.concat([pd.get_dummies(final_900['segment']),final_900],axis=1)
final_900.drop('segment',axis=1,inplace=True)

test_200 = pd.read_csv('./test200.csv')
test_200 = pd.concat([pd.get_dummies(test_200['segment']),test_200],axis=1)
test_200.drop('segment',axis=1,inplace=True)
# test set에는 1st OM 더미변수가 없었기 때문에 전부 0으로 단순히 만들어주었습니다.
test_200['1st OM'] = 0
```

In [5]:

```
print(final_900.shape)
print(test_200.shape)
```

```
(630, 62)
(200, 60)
```

In [6]:

```
features = ['IVUS_MLA','diminutive', 'diam_SB3', 'sp_D_pLAD', 'diam_SB2', 'diam_D2', 'calc_sp_RCA', 'calc_sp_LCX','calc_sp_LAD',
 '%DS', 'Sum_D3+D4+S2', 'Mid LAD', '1st OM', 'sp_D_pLCX', 'Proximal LAD', 'sp_D_LM', 'Apex curve',
 'disal_MLD', 'diam_D1', 'Proximal LCX', 'Proximal RCA', 'Distal RCA', 'diam_SB1', 'diam_D4',
 'Sum_D1+D2+S1', 'Sum_SB2+SB3', 'diam_D3', 'diam_S1', 'aver_MLD', 'age', 'Sum_D1+D2',
 'prox_MLD', 'Mid RCA', 'Distal LAD', 'RI presence', 'lesion_L', 'D_mRCA', 'Distal LCX', 'distance_os_MLD', 'MLD', 'gender', 'diam_S2', 'Sum_D3+D4']
```

# 결측값이 있는 변수¶

In [7]:

```
train_features = final_900[features]
train_features['Sum_D1+D2'] = train_features['diam_D1']+train_features['diam_D2']
train_features['Sum_D1+D2+S1'] = train_features['diam_D1']+train_features['diam_D2']+train_features['diam_S1']
train_features['Sum_D3+D4'] = train_features['diam_D3']+train_features['diam_D4']
train_features['Sum_D3+D4+S2'] = train_features['diam_D3']+train_features['diam_D4']+train_features['diam_S2']
train_features['Sum_SB2+SB3'] = train_features['diam_SB2']+train_features['diam_SB3']
```

```
C:\Anaconda3\lib\site-packages\ipykernel\__main__.py:2: SettingWithCopyWarning: 
A value is trying to be set on a copy of a slice from a DataFrame.
Try using .loc[row_indexer,col_indexer] = value instead

See the caveats in the documentation: http://pandas.pydata.org/pandas-docs/stable/indexing.html#indexing-view-versus-copy
  from ipykernel import kernelapp as app
C:\Anaconda3\lib\site-packages\ipykernel\__main__.py:3: SettingWithCopyWarning: 
A value is trying to be set on a copy of a slice from a DataFrame.
Try using .loc[row_indexer,col_indexer] = value instead

See the caveats in the documentation: http://pandas.pydata.org/pandas-docs/stable/indexing.html#indexing-view-versus-copy
  app.launch_new_instance()
C:\Anaconda3\lib\site-packages\ipykernel\__main__.py:4: SettingWithCopyWarning: 
A value is trying to be set on a copy of a slice from a DataFrame.
Try using .loc[row_indexer,col_indexer] = value instead

See the caveats in the documentation: http://pandas.pydata.org/pandas-docs/stable/indexing.html#indexing-view-versus-copy
C:\Anaconda3\lib\site-packages\ipykernel\__main__.py:5: SettingWithCopyWarning: 
A value is trying to be set on a copy of a slice from a DataFrame.
Try using .loc[row_indexer,col_indexer] = value instead

See the caveats in the documentation: http://pandas.pydata.org/pandas-docs/stable/indexing.html#indexing-view-versus-copy
C:\Anaconda3\lib\site-packages\ipykernel\__main__.py:6: SettingWithCopyWarning: 
A value is trying to be set on a copy of a slice from a DataFrame.
Try using .loc[row_indexer,col_indexer] = value instead

See the caveats in the documentation: http://pandas.pydata.org/pandas-docs/stable/indexing.html#indexing-view-versus-copy
```

In [8]:

```
test_features = test_200[features]
test_features['Sum_D1+D2'] = test_features['diam_D1']+test_features['diam_D2']
test_features['Sum_D1+D2+S1'] = test_features['diam_D1']+test_features['diam_D2']+test_features['diam_S1']
test_features['Sum_D3+D4'] = test_features['diam_D3']+test_features['diam_D4']
test_features['Sum_D3+D4+S2'] = test_features['diam_D3']+test_features['diam_D4']+test_features['diam_S2']
test_features['Sum_SB2+SB3'] = test_features['diam_SB2']+test_features['diam_SB3']
```

```
C:\Anaconda3\lib\site-packages\ipykernel\__main__.py:2: SettingWithCopyWarning: 
A value is trying to be set on a copy of a slice from a DataFrame.
Try using .loc[row_indexer,col_indexer] = value instead

See the caveats in the documentation: http://pandas.pydata.org/pandas-docs/stable/indexing.html#indexing-view-versus-copy
  from ipykernel import kernelapp as app
C:\Anaconda3\lib\site-packages\ipykernel\__main__.py:3: SettingWithCopyWarning: 
A value is trying to be set on a copy of a slice from a DataFrame.
Try using .loc[row_indexer,col_indexer] = value instead

See the caveats in the documentation: http://pandas.pydata.org/pandas-docs/stable/indexing.html#indexing-view-versus-copy
  app.launch_new_instance()
C:\Anaconda3\lib\site-packages\ipykernel\__main__.py:4: SettingWithCopyWarning: 
A value is trying to be set on a copy of a slice from a DataFrame.
Try using .loc[row_indexer,col_indexer] = value instead

See the caveats in the documentation: http://pandas.pydata.org/pandas-docs/stable/indexing.html#indexing-view-versus-copy
C:\Anaconda3\lib\site-packages\ipykernel\__main__.py:5: SettingWithCopyWarning: 
A value is trying to be set on a copy of a slice from a DataFrame.
Try using .loc[row_indexer,col_indexer] = value instead

See the caveats in the documentation: http://pandas.pydata.org/pandas-docs/stable/indexing.html#indexing-view-versus-copy
C:\Anaconda3\lib\site-packages\ipykernel\__main__.py:6: SettingWithCopyWarning: 
A value is trying to be set on a copy of a slice from a DataFrame.
Try using .loc[row_indexer,col_indexer] = value instead

See the caveats in the documentation: http://pandas.pydata.org/pandas-docs/stable/indexing.html#indexing-view-versus-copy
```

In [9]:

```
for col in train_features.columns:
    if train_features[col].isna().sum() > 0:
        print('결측값 있는 변수 : \t',col)
```

```
결측값 있는 변수 : 	 diam_SB3
결측값 있는 변수 : 	 diam_SB2
결측값 있는 변수 : 	 diam_D2
결측값 있는 변수 : 	 Sum_D3+D4+S2
결측값 있는 변수 : 	 diam_D1
결측값 있는 변수 : 	 diam_SB1
결측값 있는 변수 : 	 diam_D4
결측값 있는 변수 : 	 Sum_D1+D2+S1
결측값 있는 변수 : 	 Sum_SB2+SB3
결측값 있는 변수 : 	 diam_D3
결측값 있는 변수 : 	 diam_S1
결측값 있는 변수 : 	 Sum_D1+D2
결측값 있는 변수 : 	 diam_S2
결측값 있는 변수 : 	 Sum_D3+D4
```

In [10]:

```
for col in test_features.columns:
    if test_features[col].isna().sum() > 0:
        print('결측값 있는 변수 : \t',col)
```

```
결측값 있는 변수 : 	 diam_SB3
결측값 있는 변수 : 	 diam_SB2
결측값 있는 변수 : 	 diam_D2
결측값 있는 변수 : 	 Sum_D3+D4+S2
결측값 있는 변수 : 	 diam_D1
결측값 있는 변수 : 	 diam_SB1
결측값 있는 변수 : 	 diam_D4
결측값 있는 변수 : 	 Sum_D1+D2+S1
결측값 있는 변수 : 	 Sum_SB2+SB3
결측값 있는 변수 : 	 diam_D3
결측값 있는 변수 : 	 diam_S1
결측값 있는 변수 : 	 Sum_D1+D2
결측값 있는 변수 : 	 diam_S2
결측값 있는 변수 : 	 Sum_D3+D4
```

In [11]:

```
target = (final_900['FFR']<0.8).astype(int)
test_target = (test_200['FFR']<0.8).astype(int)
```

In [12]:

```
from sklearn.linear_model import LogisticRegressionCV
from sklearn.neural_network import MLPClassifier
from sklearn.neighbors import KNeighborsClassifier
from sklearn.svm import SVC
from sklearn.gaussian_process import GaussianProcessClassifier
from sklearn.gaussian_process.kernels import RBF
from sklearn.tree import DecisionTreeClassifier
from sklearn.ensemble import RandomForestClassifier, AdaBoostClassifier, ExtraTreesClassifier
from sklearn.naive_bayes import GaussianNB
from sklearn.discriminant_analysis import QuadraticDiscriminantAnalysis
import catboost as cgb
import lightgbm as lgb
```

In [13]:

```
classifiers = [
    LogisticRegressionCV(refit=False,n_jobs=-1),
    SVC(kernel="linear", C=0.025,probability=True),
    RandomForestClassifier(max_depth=5, n_estimators=500,n_jobs=-1,random_state=4321),
    AdaBoostClassifier(),
    cgb.CatBoostClassifier(verbose=False,random_seed =4321)
    ]
```

In [14]:

```
import sklearn.metrics as metrics
import matplotlib.pyplot as plt
import pandas_ml as pdml
%matplotlib inline
```

결측값 0으로 대체

In [15]:

```
train_features.fillna(0,inplace=True)
test_features.fillna(0,inplace=True)
```

```
C:\Anaconda3\lib\site-packages\pandas\core\frame.py:3035: SettingWithCopyWarning: 
A value is trying to be set on a copy of a slice from a DataFrame

See the caveats in the documentation: http://pandas.pydata.org/pandas-docs/stable/indexing.html#indexing-view-versus-copy
  downcast=downcast, **kwargs)
```

# test set¶

In [16]:

```
for clf in classifiers:
    print(str(clf))
    clf.fit(train_features, target)
    pred_score = clf.predict_proba(test_features)
    
    fpr, tpr, thresholds = metrics.roc_curve(test_target, pred_score[:,1])
    i = np.arange(len(tpr))
    roc = pd.DataFrame({'fpr' : pd.Series(fpr, index=i),'tpr' : pd.Series(tpr, index = i), '1-fpr' : pd.Series(1-fpr, index = i), 'tf' : pd.Series(tpr - (1-fpr), index = i), 'cutoff value' : pd.Series(thresholds, index = i)})
    print()
    print(roc.ix[(roc.tf-0).abs().argsort()[1]])
    threshold = roc.ix[(roc.tf-0).abs().argsort()[1]]['cutoff value']
    print()
    roc_auc = metrics.auc(fpr, tpr)
    pred = np.where(pred_score>=threshold,1,0)
    stats = pdml.ConfusionMatrix(test_target,pred[:,1]).stats()
    print('{} {} {} {} {} {} {}'.format(threshold.round(2), roc_auc.round(2), stats['TPR'].round(2), stats['TNR'].round(2), 
                                        stats['PPV'].round(2), stats['NPV'].round(2), stats['ACC'].round(2)))
    print()
    plt.plot(fpr, tpr, lw=2, label="ROC curve (area = {roc_auc:.2f})".format(roc_auc=roc_auc))
    plt.plot([0, 1], [0, 1], color='navy', lw=2, linestyle='--')
    plt.xlim([0.0, 1.0])
    plt.ylim([0.0, 1.05])
    plt.xlabel('False Positive Rate')
    plt.ylabel('True Positive Rate')
    plt.title('Receiver operating characteristic')
    plt.legend(loc="lower right")
    plt.show()
    print('----------------------------------------------------------------------------------')
```

```
LogisticRegressionCV(Cs=10, class_weight=None, cv=None, dual=False,
           fit_intercept=True, intercept_scaling=1.0, max_iter=100,
           multi_class='ovr', n_jobs=-1, penalty='l2', random_state=None,
           refit=False, scoring=None, solver='lbfgs', tol=0.0001,
           verbose=0)

1-fpr           0.805556
cutoff value    0.404364
fpr             0.194444
tf             -0.033816
tpr             0.771739
Name: 26, dtype: float64

0.4 0.87 0.77 0.81 0.77 0.81 0.79
```

```
C:\Anaconda3\lib\site-packages\ipykernel\__main__.py:10: DeprecationWarning: 
.ix is deprecated. Please use
.loc for label based indexing or
.iloc for positional indexing

See the documentation here:
http://pandas.pydata.org/pandas-docs/stable/indexing.html#ix-indexer-is-deprecated
```

```
----------------------------------------------------------------------------------
SVC(C=0.025, cache_size=200, class_weight=None, coef0=0.0,
  decision_function_shape='ovr', degree=3, gamma='auto', kernel='linear',
  max_iter=-1, probability=True, random_state=None, shrinking=True,
  tol=0.001, verbose=False)

1-fpr           0.787037
cutoff value    0.373111
fpr             0.212963
tf             -0.015298
tpr             0.771739
Name: 36, dtype: float64

0.37 0.86 0.77 0.79 0.76 0.8 0.78
```

```
C:\Anaconda3\lib\site-packages\ipykernel\__main__.py:10: DeprecationWarning: 
.ix is deprecated. Please use
.loc for label based indexing or
.iloc for positional indexing

See the documentation here:
http://pandas.pydata.org/pandas-docs/stable/indexing.html#ix-indexer-is-deprecated
```

```
----------------------------------------------------------------------------------
RandomForestClassifier(bootstrap=True, class_weight=None, criterion='gini',
            max_depth=5, max_features='auto', max_leaf_nodes=None,
            min_impurity_decrease=0.0, min_impurity_split=None,
            min_samples_leaf=1, min_samples_split=2,
            min_weight_fraction_leaf=0.0, n_estimators=500, n_jobs=-1,
            oob_score=False, random_state=4321, verbose=0,
            warm_start=False)

1-fpr           0.750000
cutoff value    0.406774
fpr             0.250000
tf             -0.010870
tpr             0.739130
Name: 34, dtype: float64

0.41 0.83 0.74 0.75 0.72 0.77 0.74
```

```
C:\Anaconda3\lib\site-packages\ipykernel\__main__.py:10: DeprecationWarning: 
.ix is deprecated. Please use
.loc for label based indexing or
.iloc for positional indexing

See the documentation here:
http://pandas.pydata.org/pandas-docs/stable/indexing.html#ix-indexer-is-deprecated
```

```
----------------------------------------------------------------------------------
AdaBoostClassifier(algorithm='SAMME.R', base_estimator=None,
          learning_rate=1.0, n_estimators=50, random_state=None)

1-fpr           0.731481
cutoff value    0.493071
fpr             0.268519
tf              0.007649
tpr             0.739130
Name: 35, dtype: float64

0.49 0.83 0.74 0.73 0.7 0.77 0.74
```

```
C:\Anaconda3\lib\site-packages\ipykernel\__main__.py:10: DeprecationWarning: 
.ix is deprecated. Please use
.loc for label based indexing or
.iloc for positional indexing

See the documentation here:
http://pandas.pydata.org/pandas-docs/stable/indexing.html#ix-indexer-is-deprecated
```

```
----------------------------------------------------------------------------------
<catboost.core.CatBoostClassifier object at 0x000002A4857FE240>

1-fpr           0.768519
cutoff value    0.180407
fpr             0.231481
tf              0.014090
tpr             0.782609
Name: 34, dtype: float64

0.18 0.82 0.78 0.77 0.74 0.81 0.78
```

```
----------------------------------------------------------------------------------
```

In [17]:

```
# catboost feature importance
pd.DataFrame(list(zip(classifiers[4].feature_importances_,features))).sort_values(0,ascending=False)
```

Out[17]:

|  | 0 | 1 |
| --- | --- | --- |
| 0 | 14.454654 | IVUS\_MLA |
| 9 | 5.748526 | %DS |
| 38 | 5.373849 | distance\_os\_MLD |
| 15 | 5.345162 | sp\_D\_LM |
| 29 | 5.024461 | age |
| 39 | 4.862118 | MLD |
| 36 | 4.195955 | D\_mRCA |
| 8 | 4.193352 | calc\_sp\_LAD |
| 35 | 4.018547 | lesion\_L |
| 3 | 3.906466 | sp\_D\_pLAD |
| 7 | 3.855337 | calc\_sp\_LCX |
| 28 | 3.484004 | aver\_MLD |
| 6 | 3.266250 | calc\_sp\_RCA |
| 26 | 3.195383 | diam\_D3 |
| 31 | 3.068841 | prox\_MLD |
| 17 | 3.068118 | disal\_MLD |
| 14 | 3.032536 | Proximal LAD |
| 13 | 2.658398 | sp\_D\_pLCX |
| 10 | 2.201424 | Sum\_D3+D4+S2 |
| 40 | 2.170825 | gender |
| 42 | 1.734493 | Sum\_D3+D4 |
| 24 | 1.493685 | Sum\_D1+D2+S1 |
| 18 | 1.138655 | diam\_D1 |
| 34 | 0.992599 | RI presence |
| 30 | 0.963939 | Sum\_D1+D2 |
| 22 | 0.935163 | diam\_SB1 |
| 27 | 0.872085 | diam\_S1 |
| 41 | 0.859953 | diam\_S2 |
| 32 | 0.835959 | Mid RCA |
| 1 | 0.656763 | diminutive |
| 16 | 0.318210 | Apex curve |
| 11 | 0.262093 | Mid LAD |
| 19 | 0.256135 | Proximal LCX |
| 5 | 0.244419 | diam\_D2 |
| 37 | 0.242711 | Distal LCX |
| 21 | 0.231821 | Distal RCA |
| 20 | 0.227748 | Proximal RCA |
| 23 | 0.216943 | diam\_D4 |
| 25 | 0.208550 | Sum\_SB2+SB3 |
| 4 | 0.102730 | diam\_SB2 |
| 12 | 0.031370 | 1st OM |
| 33 | 0.028618 | Distal LAD |
| 2 | 0.021149 | diam\_SB3 |

# train set cv¶

In [18]:

```
from sklearn.model_selection import StratifiedKFold
skf = StratifiedKFold(n_splits=5,random_state=4321,shuffle=True)
```

In [19]:

```
for clf in classifiers:
    t = 1
    print(str(clf))
    ci = {'accuracy':[],'sensitivity':[],'specificity':[],'PPV':[],'NPV':[],'auc':[],'threshold':[]}
    for train_index, test_index in skf.split(train_features, target):
        print("그룹 %s" % t)
        X_train = train_features.values[train_index]
        X_test = train_features.values[test_index]
        y_train = target.values[train_index]
        y_test = target.values[test_index]
        clf.fit(X_train, y_train)
        pred_score = clf.predict_proba(X_test)
        fpr, tpr, thresholds = metrics.roc_curve(y_test, pred_score[:,1])
        i = np.arange(len(tpr))
        roc = pd.DataFrame({'fpr' : pd.Series(fpr, index=i),'tpr' : pd.Series(tpr, index = i), '1-fpr' : pd.Series(1-fpr, index = i), 'tf' : pd.Series(tpr - (1-fpr), index = i), 'cutoff value' : pd.Series(thresholds, index = i)})
        print()
        print(roc.ix[(roc.tf-0).abs().argsort()[1]])
        threshold = roc.ix[(roc.tf-0).abs().argsort()[1]]['cutoff value']
        ci['threshold'].append(threshold)
        print()
        roc_auc = metrics.auc(fpr, tpr)
        pred = np.where(pred_score>=threshold,1,0)
        ci['auc'].append(roc_auc)
        stats = pdml.ConfusionMatrix(y_test,pred[:,1]).stats()
        ci['sensitivity'].append(stats['TPR'])
        ci['specificity'].append(stats['TNR'])
        ci['PPV'].append(stats['PPV'])
        ci['NPV'].append(stats['NPV'])
        ci['accuracy'].append(stats['ACC'])
        print(pdml.ConfusionMatrix(y_test,pred[:,1]).print_stats())
        print()
        plt.plot(fpr, tpr, lw=2, label="Group {t} ROC curve (area = {roc_auc:.2f})".format(t=t,roc_auc=roc_auc))
        plt.plot([0, 1], [0, 1], color='navy', lw=2, linestyle='--')
        plt.xlim([0.0, 1.0])
        plt.ylim([0.0, 1.05])
        plt.xlabel('False Positive Rate')
        plt.ylabel('True Positive Rate')
        plt.title('Receiver operating characteristic')
        plt.legend(loc="lower right")
        t += 1
    plt.show()
    print("평균적인 threshold, {} ({}) [{}-{}]".format(np.mean(ci['threshold']).round(2), np.std(ci['threshold']).round(2), 
          np.percentile(ci['threshold'],2.5).round(2), np.percentile(ci['threshold'],97.5).round(2)))
    print("평균적인 auc, {} ({}) [{}-{}]".format(np.mean(ci['auc']).round(2), np.std(ci['auc']).round(2), 
          np.percentile(ci['auc'],2.5).round(2), np.percentile(ci['auc'],97.5).round(2)))
    print("평균적인 sensitivity, {} ({}) [{}-{}]".format(np.mean(ci['sensitivity']).round(2), np.std(ci['sensitivity']).round(2), 
          np.percentile(ci['sensitivity'],2.5).round(2), np.percentile(ci['sensitivity'],97.5).round(2)))
    print("평균적인 specificity, {} ({}) [{}-{}]".format(np.mean(ci['specificity']).round(2), np.std(ci['specificity']).round(2), 
          np.percentile(ci['specificity'],2.5).round(2), np.percentile(ci['specificity'],97.5).round(2)))
    print("평균적인 ppv, {} ({}) [{}-{}]".format(np.mean(ci['PPV']).round(2), np.std(ci['PPV']).round(2), 
          np.percentile(ci['PPV'],2.5).round(2), np.percentile(ci['PPV'],97.5).round(2)))
    print("평균적인 npv, {} ({}) [{}-{}]".format(np.mean(ci['NPV']).round(2), np.std(ci['NPV']).round(2), 
          np.percentile(ci['NPV'],2.5).round(2), np.percentile(ci['NPV'],97.5).round(2)))
    print("평균적인 accuracy, {} ({}) [{}-{}]".format(np.mean(ci['accuracy']).round(2), np.std(ci['accuracy']).round(2), 
          np.percentile(ci['accuracy'],2.5).round(2), np.percentile(ci['accuracy'],97.5).round(2)))
    print('----------------------------------------------------------------------------------')
```

```
LogisticRegressionCV(Cs=10, class_weight=None, cv=None, dual=False,
           fit_intercept=True, intercept_scaling=1.0, max_iter=100,
           multi_class='ovr', n_jobs=-1, penalty='l2', random_state=None,
           refit=False, scoring=None, solver='lbfgs', tol=0.0001,
           verbose=0)
그룹 1

1-fpr           0.791045
cutoff value    0.479012
fpr             0.208955
tf             -0.028333
tpr             0.762712
Name: 24, dtype: float64

population: 126
P: 59
N: 67
PositiveTest: 59
NegativeTest: 67
TP: 45
TN: 53
FP: 14
FN: 14
TPR: 0.7627118644067796
TNR: 0.7910447761194029
PPV: 0.7627118644067796
NPV: 0.7910447761194029
FPR: 0.208955223880597
FDR: 0.23728813559322035
FNR: 0.23728813559322035
ACC: 0.7777777777777778
F1_score: 0.7627118644067796
MCC: 0.5537566405261827
informedness: 0.5537566405261827
markedness: 0.5537566405261827
prevalence: 0.46825396825396826
LRP: 3.6501210653753025
LRN: 0.2999680204669012
DOR: 12.168367346938775
FOR: 0.208955223880597
None

그룹 2
```

```
C:\Anaconda3\lib\site-packages\ipykernel\__main__.py:17: DeprecationWarning: 
.ix is deprecated. Please use
.loc for label based indexing or
.iloc for positional indexing

See the documentation here:
http://pandas.pydata.org/pandas-docs/stable/indexing.html#ix-indexer-is-deprecated
```

```
1-fpr           0.820896
cutoff value    0.496154
fpr             0.179104
tf              0.009613
tpr             0.830508
Name: 21, dtype: float64

population: 126
P: 59
N: 67
PositiveTest: 61
NegativeTest: 65
TP: 49
TN: 55
FP: 12
FN: 10
TPR: 0.8305084745762712
TNR: 0.8208955223880597
PPV: 0.8032786885245902
NPV: 0.8461538461538461
FPR: 0.1791044776119403
FDR: 0.19672131147540983
FNR: 0.1694915254237288
ACC: 0.8253968253968254
F1_score: 0.8166666666666667
MCC: 0.6504175188663124
informedness: 0.6514039969643308
markedness: 0.6494325346784362
prevalence: 0.46825396825396826
LRP: 4.637005649717515
LRN: 0.2064714946070878
DOR: 22.45833333333334
FOR: 0.15384615384615385
None

그룹 3

1-fpr           0.791045
cutoff value    0.573941
fpr             0.208955
tf             -0.011384
tpr             0.779661
Name: 20, dtype: float64

population: 126
P: 59
N: 67
PositiveTest: 60
NegativeTest: 66
TP: 46
TN: 53
FP: 14
FN: 13
TPR: 0.7796610169491526
TNR: 0.7910447761194029
PPV: 0.7666666666666667
NPV: 0.803030303030303
FPR: 0.208955223880597
FDR: 0.23333333333333334
FNR: 0.22033898305084745
ACC: 0.7857142857142857
F1_score: 0.773109243697479
MCC: 0.5702011582763243
informedness: 0.5707057930685555
markedness: 0.5696969696969698
prevalence: 0.46825396825396826
LRP: 3.7312348668280872
LRN: 0.278541733290694
DOR: 13.395604395604394
FOR: 0.19696969696969696
None

그룹 4

1-fpr           0.791045
cutoff value    0.389952
fpr             0.208955
tf             -0.028333
tpr             0.762712
Name: 18, dtype: float64

population: 126
P: 59
N: 67
PositiveTest: 59
NegativeTest: 67
TP: 45
TN: 53
FP: 14
FN: 14
TPR: 0.7627118644067796
TNR: 0.7910447761194029
PPV: 0.7627118644067796
NPV: 0.7910447761194029
FPR: 0.208955223880597
FDR: 0.23728813559322035
FNR: 0.23728813559322035
ACC: 0.7777777777777778
F1_score: 0.7627118644067796
MCC: 0.5537566405261827
informedness: 0.5537566405261827
markedness: 0.5537566405261827
prevalence: 0.46825396825396826
LRP: 3.6501210653753025
LRN: 0.2999680204669012
DOR: 12.168367346938775
FOR: 0.208955223880597
None

그룹 5

1-fpr           0.761194
cutoff value    0.379056
fpr             0.238806
tf             -0.015431
tpr             0.745763
Name: 22, dtype: float64

population: 126
P: 59
N: 67
PositiveTest: 60
NegativeTest: 66
TP: 44
TN: 51
FP: 16
FN: 15
TPR: 0.7457627118644068
TNR: 0.7611940298507462
PPV: 0.7333333333333333
NPV: 0.7727272727272727
FPR: 0.23880597014925373
FDR: 0.26666666666666666
FNR: 0.2542372881355932
ACC: 0.753968253968254
F1_score: 0.7394957983193278
MCC: 0.5065084757029051
informedness: 0.506956741715153
markedness: 0.5060606060606059
prevalence: 0.46825396825396826
LRP: 3.1228813559322033
LRN: 0.3339980059820538
DOR: 9.35
FOR: 0.22727272727272727
None
```

```
평균적인 threshold, 0.46 (0.07) [0.38-0.57]
평균적인 auc, 0.87 (0.01) [0.85-0.88]
평균적인 sensitivity, 0.78 (0.03) [0.75-0.83]
평균적인 specificity, 0.79 (0.02) [0.76-0.82]
평균적인 ppv, 0.77 (0.02) [0.74-0.8]
평균적인 npv, 0.8 (0.02) [0.77-0.84]
평균적인 accuracy, 0.78 (0.02) [0.76-0.82]
----------------------------------------------------------------------------------
SVC(C=0.025, cache_size=200, class_weight=None, coef0=0.0,
  decision_function_shape='ovr', degree=3, gamma='auto', kernel='linear',
  max_iter=-1, probability=True, random_state=None, shrinking=True,
  tol=0.001, verbose=False)
그룹 1

1-fpr           0.805970
cutoff value    0.444871
fpr             0.194030
tf             -0.009360
tpr             0.796610
Name: 24, dtype: float64

population: 126
P: 59
N: 67
PositiveTest: 60
NegativeTest: 66
TP: 47
TN: 54
FP: 13
FN: 12
TPR: 0.7966101694915254
TNR: 0.8059701492537313
PPV: 0.7833333333333333
NPV: 0.8181818181818182
FPR: 0.19402985074626866
FDR: 0.21666666666666667
FNR: 0.2033898305084746
ACC: 0.8015873015873016
F1_score: 0.7899159663865546
MCC: 0.6020474995630339
informedness: 0.6025803187452567
markedness: 0.6015151515151516
prevalence: 0.46825396825396826
LRP: 4.105606258148631
LRN: 0.2523540489642185
DOR: 16.26923076923077
FOR: 0.18181818181818182
None

그룹 2

1-fpr           0.776119
cutoff value    0.494361
fpr             0.223881
tf              0.020491
tpr             0.796610
Name: 22, dtype: float64
```

```
C:\Anaconda3\lib\site-packages\ipykernel\__main__.py:17: DeprecationWarning: 
.ix is deprecated. Please use
.loc for label based indexing or
.iloc for positional indexing

See the documentation here:
http://pandas.pydata.org/pandas-docs/stable/indexing.html#ix-indexer-is-deprecated
```

```
population: 126
P: 59
N: 67
PositiveTest: 62
NegativeTest: 64
TP: 47
TN: 52
FP: 15
FN: 12
TPR: 0.7966101694915254
TNR: 0.7761194029850746
PPV: 0.7580645161290323
NPV: 0.8125
FPR: 0.22388059701492538
FDR: 0.24193548387096775
FNR: 0.2033898305084746
ACC: 0.7857142857142857
F1_score: 0.7768595041322314
MCC: 0.5716460193099387
informedness: 0.5727295724766002
markedness: 0.5705645161290323
prevalence: 0.46825396825396826
LRP: 3.55819209039548
LRN: 0.26205997392438074
DOR: 13.577777777777776
FOR: 0.1875
None

그룹 3

1-fpr           0.835821
cutoff value    0.552260
fpr             0.164179
tf             -0.039211
tpr             0.796610
Name: 19, dtype: float64

population: 126
P: 59
N: 67
PositiveTest: 58
NegativeTest: 68
TP: 47
TN: 56
FP: 11
FN: 12
TPR: 0.7966101694915254
TNR: 0.835820895522388
PPV: 0.8103448275862069
NPV: 0.8235294117647058
FPR: 0.16417910447761194
FDR: 0.1896551724137931
FNR: 0.2033898305084746
ACC: 0.8174603174603174
F1_score: 0.8034188034188035
MCC: 0.6331522409954671
informedness: 0.6324310650139133
markedness: 0.6338742393509127
prevalence: 0.46825396825396826
LRP: 4.852080123266564
LRN: 0.24334140435835352
DOR: 19.939393939393938
FOR: 0.17647058823529413
None

그룹 4

1-fpr           0.835821
cutoff value    0.441823
fpr             0.164179
tf              0.011637
tpr             0.847458
Name: 19, dtype: float64

population: 126
P: 59
N: 67
PositiveTest: 61
NegativeTest: 65
TP: 50
TN: 56
FP: 11
FN: 9
TPR: 0.847457627118644
TNR: 0.835820895522388
PPV: 0.819672131147541
NPV: 0.8615384615384616
FPR: 0.16417910447761194
FDR: 0.18032786885245902
FNR: 0.15254237288135594
ACC: 0.8412698412698413
F1_score: 0.8333333333333334
MCC: 0.6822437741584115
informedness: 0.6832785226410321
markedness: 0.6812105926860026
prevalence: 0.46825396825396826
LRP: 5.1617873651771955
LRN: 0.18250605326876515
DOR: 28.28282828282828
FOR: 0.13846153846153847
None

그룹 5

1-fpr           0.746269
cutoff value    0.463732
fpr             0.253731
tf             -0.034404
tpr             0.711864
Name: 19, dtype: float64

population: 126
P: 59
N: 67
PositiveTest: 59
NegativeTest: 67
TP: 42
TN: 50
FP: 17
FN: 17
TPR: 0.711864406779661
TNR: 0.746268656716418
PPV: 0.711864406779661
NPV: 0.746268656716418
FPR: 0.2537313432835821
FDR: 0.288135593220339
FNR: 0.288135593220339
ACC: 0.7301587301587301
F1_score: 0.711864406779661
MCC: 0.4581330634960789
informedness: 0.45813306349607896
markedness: 0.45813306349607896
prevalence: 0.46825396825396826
LRP: 2.805583250249252
LRN: 0.3861016949152542
DOR: 7.266435986159169
FOR: 0.2537313432835821
None
```

```
평균적인 threshold, 0.48 (0.04) [0.44-0.55]
평균적인 auc, 0.87 (0.02) [0.84-0.89]
평균적인 sensitivity, 0.79 (0.04) [0.72-0.84]
평균적인 specificity, 0.8 (0.03) [0.75-0.84]
평균적인 ppv, 0.78 (0.04) [0.72-0.82]
평균적인 npv, 0.81 (0.04) [0.75-0.86]
평균적인 accuracy, 0.8 (0.04) [0.74-0.84]
----------------------------------------------------------------------------------
RandomForestClassifier(bootstrap=True, class_weight=None, criterion='gini',
            max_depth=5, max_features='auto', max_leaf_nodes=None,
            min_impurity_decrease=0.0, min_impurity_split=None,
            min_samples_leaf=1, min_samples_split=2,
            min_weight_fraction_leaf=0.0, n_estimators=500, n_jobs=-1,
            oob_score=False, random_state=4321, verbose=0,
            warm_start=False)
그룹 1

1-fpr           0.776119
cutoff value    0.472019
fpr             0.223881
tf              0.037440
tpr             0.813559
Name: 21, dtype: float64

population: 126
P: 59
N: 67
PositiveTest: 63
NegativeTest: 63
TP: 48
TN: 52
FP: 15
FN: 11
TPR: 0.8135593220338984
TNR: 0.7761194029850746
PPV: 0.7619047619047619
NPV: 0.8253968253968254
FPR: 0.22388059701492538
FDR: 0.23809523809523808
FNR: 0.1864406779661017
ACC: 0.7936507936507936
F1_score: 0.7868852459016393
MCC: 0.5884889558875502
informedness: 0.589678725018973
markedness: 0.5873015873015872
prevalence: 0.46825396825396826
LRP: 3.6338983050847458
LRN: 0.24022164276401564
DOR: 15.127272727272727
FOR: 0.1746031746031746
None

그룹 2
```

```
C:\Anaconda3\lib\site-packages\ipykernel\__main__.py:17: DeprecationWarning: 
.ix is deprecated. Please use
.loc for label based indexing or
.iloc for positional indexing

See the documentation here:
http://pandas.pydata.org/pandas-docs/stable/indexing.html#ix-indexer-is-deprecated
```

```
1-fpr           0.820896
cutoff value    0.498268
fpr             0.179104
tf             -0.024285
tpr             0.796610
Name: 20, dtype: float64

population: 126
P: 59
N: 67
PositiveTest: 59
NegativeTest: 67
TP: 47
TN: 55
FP: 12
FN: 12
TPR: 0.7966101694915254
TNR: 0.8208955223880597
PPV: 0.7966101694915254
NPV: 0.8208955223880597
FPR: 0.1791044776119403
FDR: 0.2033898305084746
FNR: 0.2033898305084746
ACC: 0.8095238095238095
F1_score: 0.7966101694915254
MCC: 0.6175056918795852
informedness: 0.6175056918795852
markedness: 0.6175056918795852
prevalence: 0.46825396825396826
LRP: 4.44774011299435
LRN: 0.2477657935285054
DOR: 17.95138888888889
FOR: 0.1791044776119403
None

그룹 3
```

```
C:\Anaconda3\lib\site-packages\ipykernel\__main__.py:17: DeprecationWarning: 
.ix is deprecated. Please use
.loc for label based indexing or
.iloc for positional indexing

See the documentation here:
http://pandas.pydata.org/pandas-docs/stable/indexing.html#ix-indexer-is-deprecated
```

```
1-fpr           0.805970
cutoff value    0.521686
fpr             0.194030
tf             -0.009360
tpr             0.796610
Name: 20, dtype: float64

population: 126
P: 59
N: 67
PositiveTest: 60
NegativeTest: 66
TP: 47
TN: 54
FP: 13
FN: 12
TPR: 0.7966101694915254
TNR: 0.8059701492537313
PPV: 0.7833333333333333
NPV: 0.8181818181818182
FPR: 0.19402985074626866
FDR: 0.21666666666666667
FNR: 0.2033898305084746
ACC: 0.8015873015873016
F1_score: 0.7899159663865546
MCC: 0.6020474995630339
informedness: 0.6025803187452567
markedness: 0.6015151515151516
prevalence: 0.46825396825396826
LRP: 4.105606258148631
LRN: 0.2523540489642185
DOR: 16.26923076923077
FOR: 0.18181818181818182
None

그룹 4
```

```
C:\Anaconda3\lib\site-packages\ipykernel\__main__.py:17: DeprecationWarning: 
.ix is deprecated. Please use
.loc for label based indexing or
.iloc for positional indexing

See the documentation here:
http://pandas.pydata.org/pandas-docs/stable/indexing.html#ix-indexer-is-deprecated
```

```
1-fpr           0.776119
cutoff value    0.432792
fpr             0.223881
tf              0.037440
tpr             0.813559
Name: 15, dtype: float64

population: 126
P: 59
N: 67
PositiveTest: 63
NegativeTest: 63
TP: 48
TN: 52
FP: 15
FN: 11
TPR: 0.8135593220338984
TNR: 0.7761194029850746
PPV: 0.7619047619047619
NPV: 0.8253968253968254
FPR: 0.22388059701492538
FDR: 0.23809523809523808
FNR: 0.1864406779661017
ACC: 0.7936507936507936
F1_score: 0.7868852459016393
MCC: 0.5884889558875502
informedness: 0.589678725018973
markedness: 0.5873015873015872
prevalence: 0.46825396825396826
LRP: 3.6338983050847458
LRN: 0.24022164276401564
DOR: 15.127272727272727
FOR: 0.1746031746031746
None

그룹 5
```

```
C:\Anaconda3\lib\site-packages\ipykernel\__main__.py:17: DeprecationWarning: 
.ix is deprecated. Please use
.loc for label based indexing or
.iloc for positional indexing

See the documentation here:
http://pandas.pydata.org/pandas-docs/stable/indexing.html#ix-indexer-is-deprecated
```

```
1-fpr           0.761194
cutoff value    0.423091
fpr             0.238806
tf             -0.032380
tpr             0.728814
Name: 17, dtype: float64

population: 126
P: 59
N: 67
PositiveTest: 59
NegativeTest: 67
TP: 43
TN: 51
FP: 16
FN: 16
TPR: 0.7288135593220338
TNR: 0.7611940298507462
PPV: 0.7288135593220338
NPV: 0.7611940298507462
FPR: 0.23880597014925373
FDR: 0.2711864406779661
FNR: 0.2711864406779661
ACC: 0.746031746031746
F1_score: 0.7288135593220338
MCC: 0.49000758917278014
informedness: 0.4900075891727802
markedness: 0.4900075891727802
prevalence: 0.46825396825396826
LRP: 3.051906779661017
LRN: 0.35626453971419075
DOR: 8.56640625
FOR: 0.23880597014925373
None
```

```
C:\Anaconda3\lib\site-packages\ipykernel\__main__.py:17: DeprecationWarning: 
.ix is deprecated. Please use
.loc for label based indexing or
.iloc for positional indexing

See the documentation here:
http://pandas.pydata.org/pandas-docs/stable/indexing.html#ix-indexer-is-deprecated
```

```
평균적인 threshold, 0.47 (0.04) [0.42-0.52]
평균적인 auc, 0.87 (0.02) [0.83-0.89]
평균적인 sensitivity, 0.79 (0.03) [0.74-0.81]
평균적인 specificity, 0.79 (0.02) [0.76-0.82]
평균적인 ppv, 0.77 (0.02) [0.73-0.8]
평균적인 npv, 0.81 (0.02) [0.77-0.83]
평균적인 accuracy, 0.79 (0.02) [0.75-0.81]
----------------------------------------------------------------------------------
AdaBoostClassifier(algorithm='SAMME.R', base_estimator=None,
          learning_rate=1.0, n_estimators=50, random_state=None)
그룹 1

1-fpr           0.746269
cutoff value    0.500451
fpr             0.253731
tf              0.050342
tpr             0.796610
Name: 25, dtype: float64
```

```
C:\Anaconda3\lib\site-packages\ipykernel\__main__.py:17: DeprecationWarning: 
.ix is deprecated. Please use
.loc for label based indexing or
.iloc for positional indexing

See the documentation here:
http://pandas.pydata.org/pandas-docs/stable/indexing.html#ix-indexer-is-deprecated
```

```
population: 126
P: 59
N: 67
PositiveTest: 64
NegativeTest: 62
TP: 47
TN: 50
FP: 17
FN: 12
TPR: 0.7966101694915254
TNR: 0.746268656716418
PPV: 0.734375
NPV: 0.8064516129032258
FPR: 0.2537313432835821
FDR: 0.265625
FNR: 0.2033898305084746
ACC: 0.7698412698412699
F1_score: 0.7642276422764228
MCC: 0.5418517479854807
informedness: 0.5428788262079434
markedness: 0.5408266129032258
prevalence: 0.46825396825396826
LRP: 3.1395812562313057
LRN: 0.2725423728813559
DOR: 11.519607843137255
FOR: 0.1935483870967742
None

그룹 2

1-fpr           0.761194
cutoff value    0.500223
fpr             0.238806
tf             -0.015431
tpr             0.745763
Name: 19, dtype: float64

population: 126
P: 59
N: 67
PositiveTest: 60
NegativeTest: 66
TP: 44
TN: 51
FP: 16
FN: 15
TPR: 0.7457627118644068
TNR: 0.7611940298507462
PPV: 0.7333333333333333
NPV: 0.7727272727272727
FPR: 0.23880597014925373
FDR: 0.26666666666666666
FNR: 0.2542372881355932
ACC: 0.753968253968254
F1_score: 0.7394957983193278
MCC: 0.5065084757029051
informedness: 0.506956741715153
markedness: 0.5060606060606059
prevalence: 0.46825396825396826
LRP: 3.1228813559322033
LRN: 0.3339980059820538
DOR: 9.35
FOR: 0.22727272727272727
None

그룹 3
```

```
C:\Anaconda3\lib\site-packages\ipykernel\__main__.py:17: DeprecationWarning: 
.ix is deprecated. Please use
.loc for label based indexing or
.iloc for positional indexing

See the documentation here:
http://pandas.pydata.org/pandas-docs/stable/indexing.html#ix-indexer-is-deprecated
```

```
1-fpr           0.716418
cutoff value    0.503930
fpr             0.283582
tf              0.063243
tpr             0.779661
Name: 20, dtype: float64

population: 126
P: 59
N: 67
PositiveTest: 65
NegativeTest: 61
TP: 46
TN: 48
FP: 19
FN: 13
TPR: 0.7796610169491526
TNR: 0.7164179104477612
PPV: 0.7076923076923077
NPV: 0.7868852459016393
FPR: 0.2835820895522388
FDR: 0.2923076923076923
FNR: 0.22033898305084745
ACC: 0.746031746031746
F1_score: 0.7419354838709677
MCC: 0.4953276716492577
informedness: 0.4960789273969137
markedness: 0.4945775535939472
prevalence: 0.46825396825396826
LRP: 2.7493309545049067
LRN: 0.3075564971751413
DOR: 8.93927125506073
FOR: 0.21311475409836064
None

그룹 4
```

```
C:\Anaconda3\lib\site-packages\ipykernel\__main__.py:17: DeprecationWarning: 
.ix is deprecated. Please use
.loc for label based indexing or
.iloc for positional indexing

See the documentation here:
http://pandas.pydata.org/pandas-docs/stable/indexing.html#ix-indexer-is-deprecated
```

```
1-fpr           0.761194
cutoff value    0.493299
fpr             0.238806
tf              0.018467
tpr             0.779661
Name: 20, dtype: float64

population: 126
P: 59
N: 67
PositiveTest: 62
NegativeTest: 64
TP: 46
TN: 51
FP: 16
FN: 13
TPR: 0.7796610169491526
TNR: 0.7611940298507462
PPV: 0.7419354838709677
NPV: 0.796875
FPR: 0.23880597014925373
FDR: 0.25806451612903225
FNR: 0.22033898305084745
ACC: 0.7698412698412699
F1_score: 0.7603305785123967
MCC: 0.5398317973872124
informedness: 0.5408550467998987
markedness: 0.5388104838709677
prevalence: 0.46825396825396826
LRP: 3.2648305084745766
LRN: 0.28946493851778
DOR: 11.278846153846155
FOR: 0.203125
None

그룹 5
```

```
C:\Anaconda3\lib\site-packages\ipykernel\__main__.py:17: DeprecationWarning: 
.ix is deprecated. Please use
.loc for label based indexing or
.iloc for positional indexing

See the documentation here:
http://pandas.pydata.org/pandas-docs/stable/indexing.html#ix-indexer-is-deprecated
```

```
1-fpr           0.716418
cutoff value    0.496616
fpr             0.283582
tf              0.063243
tpr             0.779661
Name: 21, dtype: float64

population: 126
P: 59
N: 67
PositiveTest: 65
NegativeTest: 61
TP: 46
TN: 48
FP: 19
FN: 13
TPR: 0.7796610169491526
TNR: 0.7164179104477612
PPV: 0.7076923076923077
NPV: 0.7868852459016393
FPR: 0.2835820895522388
FDR: 0.2923076923076923
FNR: 0.22033898305084745
ACC: 0.746031746031746
F1_score: 0.7419354838709677
MCC: 0.4953276716492577
informedness: 0.4960789273969137
markedness: 0.4945775535939472
prevalence: 0.46825396825396826
LRP: 2.7493309545049067
LRN: 0.3075564971751413
DOR: 8.93927125506073
FOR: 0.21311475409836064
None
```

```
C:\Anaconda3\lib\site-packages\ipykernel\__main__.py:17: DeprecationWarning: 
.ix is deprecated. Please use
.loc for label based indexing or
.iloc for positional indexing

See the documentation here:
http://pandas.pydata.org/pandas-docs/stable/indexing.html#ix-indexer-is-deprecated
```

```
평균적인 threshold, 0.5 (0.0) [0.49-0.5]
평균적인 auc, 0.84 (0.03) [0.8-0.88]
평균적인 sensitivity, 0.78 (0.02) [0.75-0.79]
평균적인 specificity, 0.74 (0.02) [0.72-0.76]
평균적인 ppv, 0.73 (0.01) [0.71-0.74]
평균적인 npv, 0.79 (0.01) [0.77-0.81]
평균적인 accuracy, 0.76 (0.01) [0.75-0.77]
----------------------------------------------------------------------------------
<catboost.core.CatBoostClassifier object at 0x000002A4857FE240>
그룹 1

1-fpr           0.791045
cutoff value    0.364990
fpr             0.208955
tf              0.022515
tpr             0.813559
Name: 23, dtype: float64

population: 126
P: 59
N: 67
PositiveTest: 62
NegativeTest: 64
TP: 48
TN: 53
FP: 14
FN: 11
TPR: 0.8135593220338984
TNR: 0.7910447761194029
PPV: 0.7741935483870968
NPV: 0.828125
FPR: 0.208955223880597
FDR: 0.22580645161290322
FNR: 0.1864406779661017
ACC: 0.8015873015873016
F1_score: 0.7933884297520661
MCC: 0.603460241232665
informedness: 0.6046040981533012
markedness: 0.6023185483870968
prevalence: 0.46825396825396826
LRP: 3.8934624697336564
LRN: 0.23568915893827952
DOR: 16.51948051948052
FOR: 0.171875
None

그룹 2

1-fpr           0.805970
cutoff value    0.527732
fpr             0.194030
tf             -0.026309
tpr             0.779661
Name: 20, dtype: float64

population: 126
P: 59
N: 67
PositiveTest: 59
NegativeTest: 67
TP: 46
TN: 54
FP: 13
FN: 13
TPR: 0.7796610169491526
TNR: 0.8059701492537313
PPV: 0.7796610169491526
NPV: 0.8059701492537313
FPR: 0.19402985074626866
FDR: 0.22033898305084745
FNR: 0.22033898305084745
ACC: 0.7936507936507936
F1_score: 0.7796610169491526
MCC: 0.5856311662028839
informedness: 0.5856311662028839
markedness: 0.5856311662028839
prevalence: 0.46825396825396826
LRP: 4.018252933507171
LRN: 0.27338355304457
DOR: 14.698224852071004
FOR: 0.19402985074626866
None

그룹 3

1-fpr           0.835821
cutoff value    0.757777
fpr             0.164179
tf             -0.073109
tpr             0.762712
Name: 20, dtype: float64

population: 126
P: 59
N: 67
PositiveTest: 56
NegativeTest: 70
TP: 45
TN: 56
FP: 11
FN: 14
TPR: 0.7627118644067796
TNR: 0.835820895522388
PPV: 0.8035714285714286
NPV: 0.8
FPR: 0.16417910447761194
FDR: 0.19642857142857142
FNR: 0.23728813559322035
ACC: 0.8015873015873016
F1_score: 0.782608695652174
MCC: 0.6010468142809241
informedness: 0.5985327599291677
markedness: 0.6035714285714286
prevalence: 0.46825396825396826
LRP: 4.645608628659476
LRN: 0.2838983050847458
DOR: 16.36363636363636
FOR: 0.2
None

그룹 4

1-fpr           0.776119
cutoff value    0.265591
fpr             0.223881
tf              0.037440
tpr             0.813559
Name: 18, dtype: float64

population: 126
P: 59
N: 67
PositiveTest: 63
NegativeTest: 63
TP: 48
TN: 52
FP: 15
FN: 11
TPR: 0.8135593220338984
TNR: 0.7761194029850746
PPV: 0.7619047619047619
NPV: 0.8253968253968254
FPR: 0.22388059701492538
FDR: 0.23809523809523808
FNR: 0.1864406779661017
ACC: 0.7936507936507936
F1_score: 0.7868852459016393
MCC: 0.5884889558875502
informedness: 0.589678725018973
markedness: 0.5873015873015872
prevalence: 0.46825396825396826
LRP: 3.6338983050847458
LRN: 0.24022164276401564
DOR: 15.127272727272727
FOR: 0.1746031746031746
None

그룹 5

1-fpr           0.761194
cutoff value    0.260415
fpr             0.238806
tf              0.035416
tpr             0.796610
Name: 23, dtype: float64

population: 126
P: 59
N: 67
PositiveTest: 63
NegativeTest: 63
TP: 47
TN: 51
FP: 16
FN: 12
TPR: 0.7966101694915254
TNR: 0.7611940298507462
PPV: 0.746031746031746
NPV: 0.8095238095238095
FPR: 0.23880597014925373
FDR: 0.25396825396825395
FNR: 0.2033898305084746
ACC: 0.7777777777777778
F1_score: 0.7704918032786885
MCC: 0.5566787420557908
informedness: 0.5578041993422715
markedness: 0.5555555555555556
prevalence: 0.46825396825396826
LRP: 3.3358050847457625
LRN: 0.2671984047856431
DOR: 12.484374999999998
FOR: 0.19047619047619047
None
```

```
평균적인 threshold, 0.44 (0.19) [0.26-0.73]
평균적인 auc, 0.86 (0.02) [0.85-0.89]
평균적인 sensitivity, 0.79 (0.02) [0.76-0.81]
평균적인 specificity, 0.79 (0.03) [0.76-0.83]
평균적인 ppv, 0.77 (0.02) [0.75-0.8]
평균적인 npv, 0.81 (0.01) [0.8-0.83]
평균적인 accuracy, 0.79 (0.01) [0.78-0.8]
----------------------------------------------------------------------------------
```
